# Ni-catalyzed carbamoylation of unactivated alkenes for stereoselective construction of six-membered lactams

**DOI:** 10.1038/s41467-022-33425-3

**Published:** 2022-10-10

**Authors:** Chenhuan Zhang, Xianqing Wu, Tingting Xia, Jingping Qu, Yifeng Chen

**Affiliations:** Key Laboratory for Advanced Materials and Joint International Research Laboratory of Precision Chemistry and Molecular Engineering, Feringa Nobel Prize Scientist Joint Research Center, Frontiers Science Center for Materiobiology and Dynamic Chemistry, School of Chemistry and Molecular Engineering, East China University of Science and Technology, 130 Meilong Road, Shanghai, 200237 China

**Keywords:** Asymmetric catalysis, Synthetic chemistry methodology

## Abstract

Nitrogen-based heterocycles have aroused widespread interest due to their reoccurrence in many pharmaceuticals. Amongst these motifs, the enantioenriched lactams are the ubiquitous scaffolds found in myriad biologically active natural products and drugs. Recently, the transition metal-catalyzed asymmetric carbamoylation has been widely employed as a straightforward arsenal for chiral lactam architecture synthesis, including β-lactam and γ-lactam. However, despite the extensive efforts, there still remains no protocol to accomplish the related δ-lactam synthesis. In this manuscript, the Ni-catalyzed enantioselective carbamoylation of unactivated alkenes by the leverage of reductive dicarbofunctionalization strategy allows for the expedient access to two types of mostly common six-membered lactams: 3,4-dihydroquinolinones and 2-piperidinone in high yield and enantioselectivity. This protocol features with good functional group tolerance, as well as broad substrate scope. The newly developed chiral 8-Quinox skeleton ligand is the key parameter for this transformation, which significantly enhances the reactivity and enantioselectivity.

## Abstract (web summary)

Six-membered chiral lactams are common structural motifs of pharmaceuticals. Here, the authors describe a nickel-catalyzed reductive carbamoylation of alkenes to form enantioenriched six-membered lactams.

## Introduction

Nitrogen-containing heterocycles constitute the versatile structure motifs in organic and medicinal chemistry^1,2^. Amongst these, chiral lactam is recognized as one of the most privileged skeletons^3–6^, which also serves as a valuable subunit for complex molecular synthesis and drug discovery. Owing to these unique pharmacological properties and synthetic utilities, tremendous efforts have been devoted to the development of facile and robust methodologies for stereoselective synthesis of this architecture^7–15^. However, a particular synthetic challenge in this scenario is the enantioselective synthesis of α-alkylated lactam enabled by asymmetric catalysis, likely due to the lack of general asymmetric α-alkylation of simple lactam^16–19^. Organocatalyst-promoted alkylation and Michael addition were developed to tackle the aforementioned challenge. Nevertheless, this strategy is largely limited to the lactam scope wherein an electron-withdrawing group including ester or aryl group was required at the adjacent position of amide functionality to generate the reactive chiral enolate intermediate^20–23^.

**Fig. 1:**
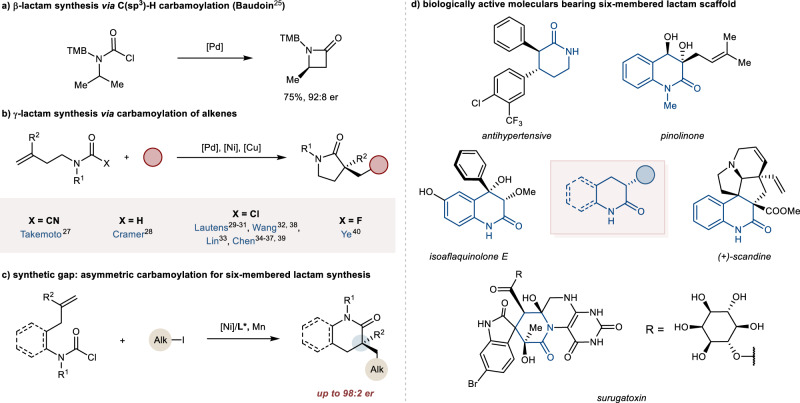
Asymmetric carbamoylation for chiral lactam synthesis. **a** β-Lactam synthesis enabled by the C(sp^3^)-H carbamoylation. **b** γ-Lactam synthesis enabled by the carbamoylation of alkenes. **c** Asymmetric carbamoylation for six-membered-lactam synthesis (this work). **d** Representative six-membered lactams skeleton in myriad biologically active natural products and drugs.

A strategically distinct approach to forge chiral lactam is the cyclization from the acyclic fragment by the synergistic formation of amide functionality and set-up of a new stereogenic center in the formed lactam ring, namely as asymmetric carbamoylation. The representative precursor of carbamoylation is formamide and carbamoyl halide, which possess several advantages, including the one-step synthesis from the prevalent secondary amine and exceptional chemo-stable property^24^. Recently, several transition metal-catalyzed asymmetric transformations involving carbamoyl electrophiles have emerged as a fascinating tool box for synthesis of chiral lactam moiety^25–40^. Baudoin group accomplished the only example to construct the β-lactam by palladium-catalyzed desymmetric C(sp^3^)‒H carbamoylation (Fig. 1a)^25^. The most reliable approaches are the transition metal-catalyzed enantioselective cyclization of carbamoyl electrophile to the pendent alkenes in which the stereogenic center was simultaneously constructed at the α-position of amide functionality with the cyclization to afford the lactam ring. This approach allows for the facile synthesis of five-membered lactam, including oxindole and γ-lactam (Fig. 1b)^27–40^. The seminal Pd-catalyzed asymmetric cyanocarbamoylation of alkenes was developed by Takemoto to access the oxindole^27^. The hydrocarbamoylation of alkene was accomplished by Cramer to allow the expedient synthesis of α-methyl pyrrolidinone^28^. Very recently, a series of asymmetric carbamoylation-initiated difunctionalization of alkenes including borocarbamoylation^29,30^, iodocarbamoylation^31^, acylcarbamoylation^32,33^, alkylcarbamoylation^34–38^ and arylcarbamoylation^39,40^ were independently developed by Lautens, Wang, Lin, Ye and our group. All the above methods could only provide the synthesis of α-alkylated five-membered lactam by the asymmetric *5-exo-trig* cyclization. Despite the apparent similarities to γ-lactam, there is no example of asymmetric α-alkylated six-membered lactams synthesis by enantioselective carbamoylation (Fig. 1c).

Six-membered lactams, including δ-lactam and dihydroquinolinone, are ubiquitous motif found across biologically active molecules (Fig. 1d)^41,42^ We envisaged that the aforesaid synthetic difficulty lies in the lack of rigidity of the longer chain and the larger spatial distances, as supported by theoretical calculations that *6-exo-trig* cyclization of d^10^ transition metal-controlled intramolecular migratory insertion of unsaturated bond was disfavored compared with the *5-exo-trig* cyclization in Baldwin-type rules^43,44^. Recently, the nickel-catalyzed asymmetric reductive cyclized difunctionalization of alkenes strategy has received the considerable attention, which allows the rapid stereoselective formation of five-membered carbo-skeleton or heterocycles^45–66^.

Herein, we report the unprecedented *6-exo-trig* cyclized carbamoylation by the utilization of the nickel-catalyzed asymmetric reductive difunctionalization of alkenes strategy. It should be noted this also represents the only example for the construction of six-membered nitrogen-containing heterocycles in asymmetric reductive cross-coupling reactions^64^. The utilization of newly modified 8-Quinox ligand enables the synthesis of α-alkylated six-membered lactam with satisfied yields and enantioselectivities.

## Results and Discussion

### Reaction optimization for dihydroquinolinone synthesis

**Fig. 2:**
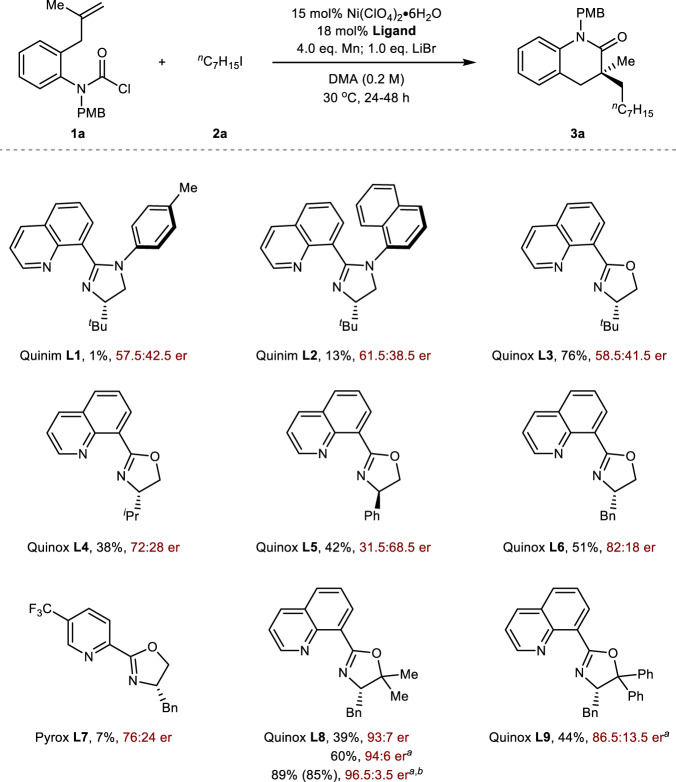
Optimization of the reaction conditions. The reaction was performed with carbamoyl chloride **1a** (1.0 eq.), iodoheptane **2a** (3.0 eq.), Ni(ClO_4_)_2_•6H_2_O (15 mol%), Ligand (18 mol%), Mn (4.0 eq.), LiBr (1.0 eq.), DMA (0.2 M), stirred for 24 h under 30 °C. The yields were reported as corrected GC yield and the er values were determined by chiral HPLC analysis on a chiral stationary phase. ^a^1.0 eq. LiI. ^b^The reaction was performed in DMA/MeCN (v/v = 4/1), isolated yield on 0.2 mmol scale for 48 h was reported in parentheses.

The initial investigation of the carbamoyl-alkylation was commenced with carbamoyl chloride **1a** and iodoheptane **2a** as electrophiles, Ni(ClO_4_)_2_•6H_2_O as catalyst, Mn as reductant, LiBr as additive and DMA as solvent (Fig. 2). The Quinim ligands (**L1** and **L2**) exploited in our lab^34,35^, were first examined, but only trace of target product **3a** was obtained with less than 30% ee. To our delight, the corrected GC yield of **3a** significantly increased to 76% with the utilization of Quinox **L3**^36,37,67^ instead of Quinim, though the er value was only 58.5:41.5. Encouraged by this result, a series of structurally defined Quinox ligands (**L4**‒**L6**) were examined in this transformation. Quinox **L6** provided the desired 3,4-dihydroquinolinone **3a** in 82:18 er with moderate yield, demonstrating that the benzyl group in oxazoline was crucial for this reductive cross-coupling reaction. It was worth noting that the benzyl group substituted Pyrox **L7**, which was widely used in asymmetric reductive reaction, exhibited poor reactivity. With the employment of Quinox **L8** by incorporating dimethyl group at the C5-position of the oxazoline ring, the enantioselectivity of **3a** was significantly increased to 93:7 er. However, replacing the substituent group of oxazoline ring from dimethyl group to diphenyl group (**L9**) decreased the enantioselectivity (44% yield, 86.5:13.5 er). With the optimal ligand (**L8**), reaction efficiency could be improved by treatment of LiI as additive. Finally, the mixed solvent system of DMA/MeCN was found to be beneficial for reactivity without dropping the enantioselectivity, and the product **3a** could be obtained in 85% isolated yield and 96.5:3.5 er.

### Substrate scope of carbamoyl chloride

**Fig. 3:**
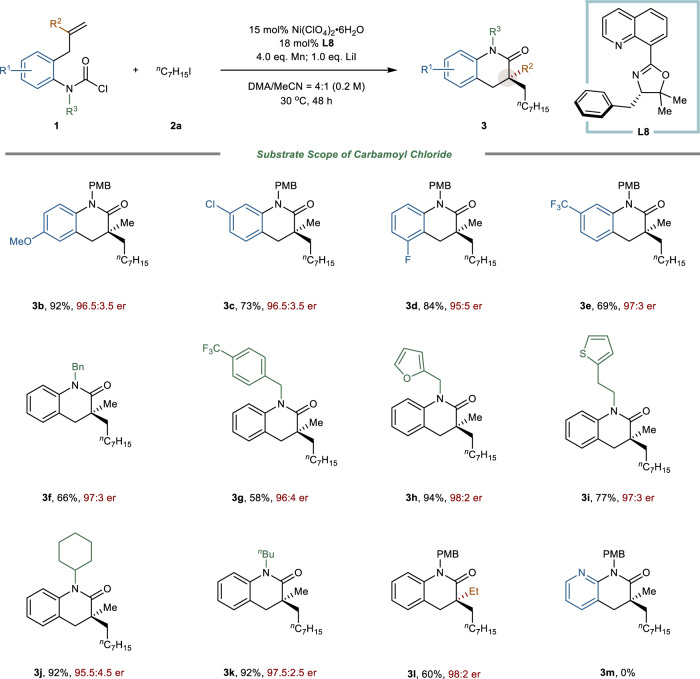
Substrate scope of carbamoyl chloride. The reaction was performed with carbamoyl chloride **1** (1.0 eq.), iodoheptane **2a** (3.0 eq.), Ni(ClO_4_)_2_•6H_2_O (15 mol%), **L8** (18 mol%), Mn (4.0 eq.), LiI (1.0 eq.), DMA/MeCN (v/v = 4/1, 0.2 M), stirred for 48 h under 30 °C. Yields were for isolated products. The er values were determined by chiral HPLC analysis on a chiral stationary phase.

With optimized conditions in hand, we next investigated the scope of the carbamoyl chloride (Fig. 3). Substrates with electron-donating (-OMe) or electron-withdrawing (–F, –Cl, –CF_3_) on the aromatic rings were tolerated, affording corresponding products in excellent reactivity and enantioselectivity (**3b**‒**3e**). Notably, the current procedure could proceed with the carbamoyl chloride averting the direct coupling with aryl chloride, leaving a hand to further transformation. In addition, the carbamoyl chlorides with diverse substituents on N-protecting group could react smoothly to provide **3** **f**‒**3k** with up to 94% yield and up to 98:2 er. Substrates containing heteroaromatic substituents such as furan (**3** **h**), thiophene (**3i**) did not impede the catalytic cycle, which were accommodated with high yield and er. Remarkably, the alkene baring ethyl group delivered the dihydroquinolinone containing all-carbon quaternary center **3** **l** in 60% yield and 98:2 er. Unfortunately, when we switched the aromatic ring from aryl to heteroaryl group such as pyridine, the corresponding products could not be observed during the reaction (**3** **m**). As for thiophene group, we could not obtain the carbamoyl chloride substrate due to its instability.

### Substrate scope of alkyl iodide

**Fig. 4:**
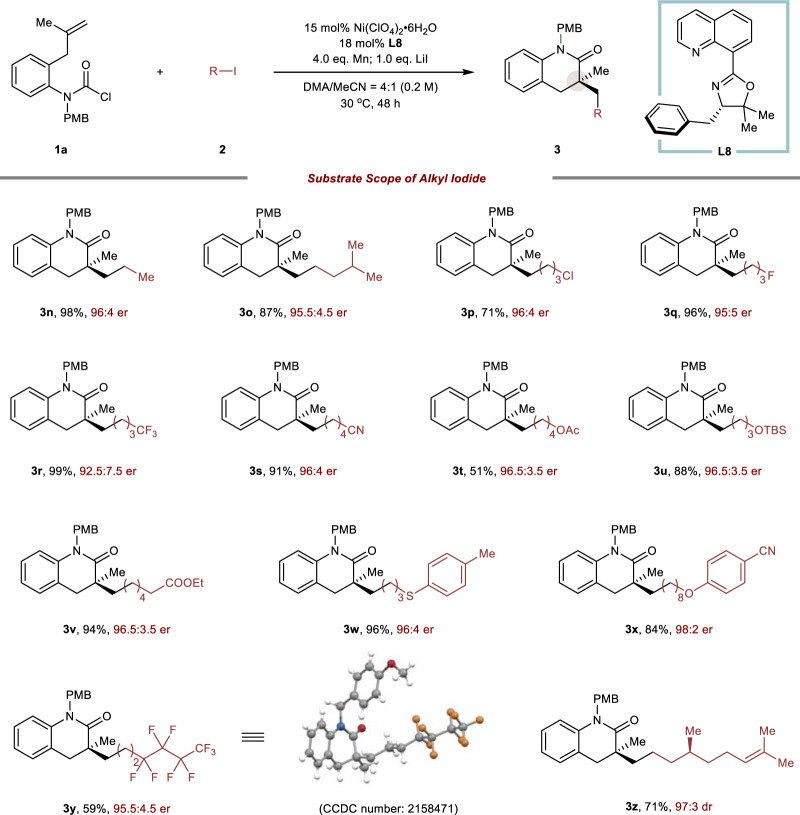
Substrate scope of alkyl iodide. The reaction was performed with carbamoyl chloride **1a** (1.0 eq.), iodide **2** (3.0 eq.), Ni(ClO_4_)_2_•6H_2_O (15 mol%), **L8** (18 mol%), Mn (4.0 eq.), LiI (1.0 eq.), DMA/MeCN (v/v = 4/1, 0.2 M), stirred for 48 h under 30 °C. Yields were for isolated products. The er values were determined by chiral HPLC analysis on a chiral stationary phase.

Inspired by the high functional group tolerance of carbamoyl chloride, we next turned our attention to variations of the alkyl iodide (Fig. 4). Simple alkyl iodide such as ethyl iodide (**3n**) and isoamyl iodide (**3o**) could be compatible in this methodology, which delivered the corresponding 3,4-dihydroquinolinones in high yield and enantioselectivity. Gratifyingly, alkyl iodides bearing –Cl (**3p**), –F (**3q**), –CN (**3** **s**) groups could also be tolerant to give the desired products with up to 96:4 er. However, CF_3_ substituents resulted in a sharp decline in enantioselectivity, albeit with almost quantitative yield (**3r**). Additionally, perfluoride-substituted alkyl electrophile had a slightly negative effect on the reaction, giving product **3** **y** in 59% yield and 95.5:4.5 er, and the absolute configuration was assigned unambiguously by X-ray diffraction. In addition, various functional groups, including esters (**3** **v**), thioether (**3w**), cyano (**3x**) were also harmonious, delivering the corresponding products in moderate to excellent yields, highlighting the synthetic potential of this methodology. The alkyl iodide converted from citronellol could also be tolerant in this reaction, produced **3z** in 71% yield and 97:3 dr. It should be noted that the primary 1-bromoheptane delivered the product **3a** in 83% yield with 90.5:9.5 er. However, the secondary and tertiary alkyl iodides failed to deliver the corresponding products at the standard condition.

### Study on the none aromatic ring tethered carbamoyl chloride

**Fig. 5:**
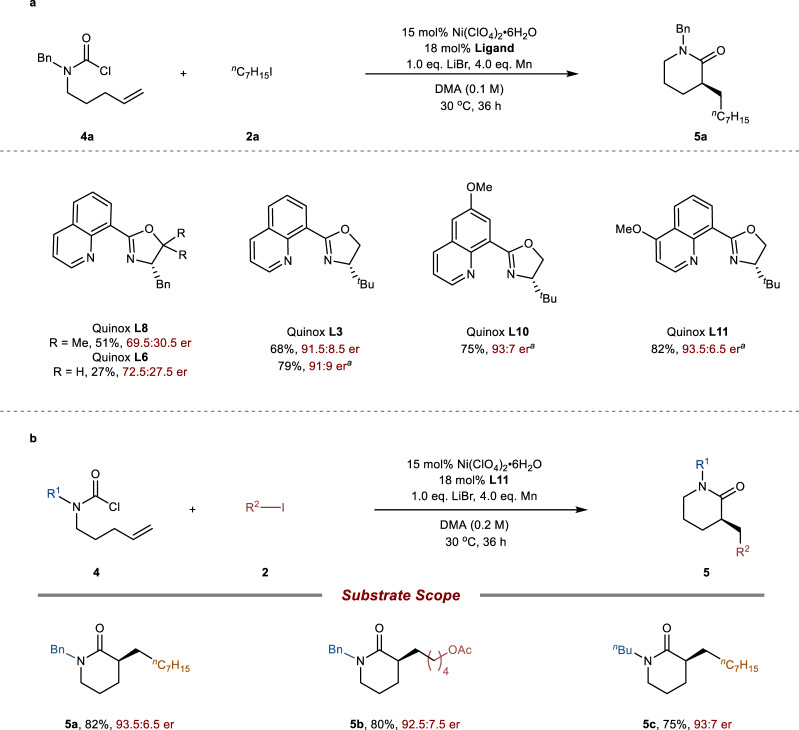
Investigation of none aromatic ring tethered carbamoyl chloride. **a** Optimization of the reaction conditions of none aromatic ring tethered carbamoyl chloride. The reaction was performed with carbamoyl chloride **4a** (1.0 eq.), **2a** (3.0 eq.), Ni(ClO_4_)_2_•6H_2_O (15 mol%), Ligand (18 mol%), Mn (4.0 eq.), LiBr (1.0 eq.), DMA (0.1 M), stirred for 36 h under 30 °C. The yields were reported as corrected GC yield and the er values were determined by HPLC analysis; ^a^0.2 M. **b** Substrate scope for δ-lactam synthesis.

To further investigate this enantioselective tandem cyclization/reductive cross-coupling protocol, we turned our attention to the none aromatic ring tethered carbamoyl chloride which is more flexible, thus causing more challenge for the cyclized carbamoylation (Fig. 5). When readily available carbamoyl chloride **4a** as starting material, Ni(ClO_4_)_2_•6H_2_O as catalyst, Mn as reductant, LiBr as additive and DMA as solvent, we evaluated the ligand effect in the asymmetric reductive carbamoyl-alkylation reaction (Fig. 5a). Unfortunately, the reaction performed with ligand Quinox **L8** used in the above reactions provided moderate yield (51%) but with low enantioselectivity (39% ee). In contrast, Quinox **L6** afford the **5a** in better enantioselectivity (72.5:27.5 er). Then, it was found that Quinox **L3** deliver **5a** in 79% yield and 91:9 er. The modification of the electronics of quinolone revealed that the 6-OMe-Quinox **L10** would further elevate the enantioselectivity into 93:7 er. Finally, switch the electronic rich methoxy group at the C-4 position of quinolone ring, namely Quinox **L11** provided the optimal results, the δ-lactam **5a** was obtained in 82% isolated yield and 93.5:6.5 er. Further screening of additives and solvents did not yield better results (See Supplementary Fig. 6). The exploration of alkyl iodide coupling component and substitution effect on the carbamoyl chloride precursor has little effect on both reaction efficiency and ee (**5b**, **5c**). At current stage, this protocol was not suitable for the construction of β-lactam or seven-membered lactam skeleton.

### Synthetic utility

**Fig. 6:**
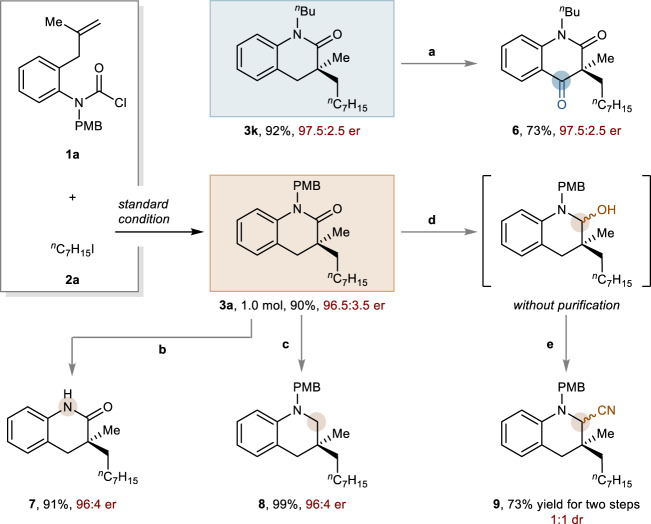
Synthetic transformations of quinolinone products. Reaction conditions: ^a^4.0 eq. CAN, MeCN/H_2_O (0.03 M), r.t.; ^b^TFA (0.1 M), DCM (0.1 M), reflux; ^c^2.0 eq. DIBAL-H, DCM, −78 °C.; ^d^1.0 eq. Red-Al, THF, −2 ~ −5 °C; ^e^1.5 eq. BF_3_•Et_2_O, 1.5 eq. TMSCN.

To demonstrate the synthetic utility of six-membered lactam, products **3a** and **3k** were employed as the building blocks for synthesis of various valuable products (Fig. 6). Firstly, direct oxidation of **3k** by using ceric ammonium nitrate (CAN) delivered **6** with high yield and retention of enantioselectivity. With regards to **3a**, the PMB group of quinolinone **3a** could be removed by treatment with trifluoroacetic acid (TFA) to afford **7** in 91% isolated yield and 96:4 er. Subsequently, the reduction operation of **3a** was carried out using diisobutylaluminum hydride (DIBAL-H) to afford chiral tetrahydroquinoline **8** in almost quantitative yield with enantiopurity maintained. Furthermore, the sequential reduction using sodium bis(2-methoxyethoxy)-aluminiumhydride (Red-Al) and cyanidation by trimethylsilyl cyanide could provide chiral α-cyano tetrahydroquinoline **9** in 73% yield.

### Mechanistic investigation

**Fig. 7:**
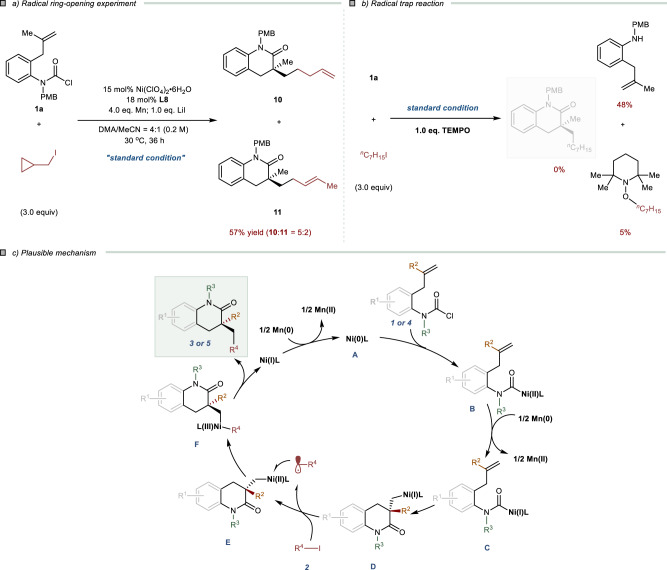
Mechanistic experiment. **a** Radical ring-opening experiment. The yield was reported as isolated yield. **b** Radical trap reaction using TEMPO as radical scavenger. The yields were reported as corrected GC yield with dodecane as an internal standard. **c** Plausible mechanism.

Several preliminary studies have been carried out to elucidate the plausible mechanism of this transformation (Fig. 7). Firstly, a radical ring-opening experiment of (iodomethyl)cyclopropane as electrophile has been performed. The mixture of ring-opening products **10** and **11** were obtained (Fig. 7a). Additionally, the addition of TEMPO (1.0 equiv) completely inhibited the reductive cross-couplings, and only led to decarbonylation by-product (Fig. 7b). Combined with the above experiments and our previous results, a plausible catalytic cycle could be speculated (Fig. 7c):^35^ Firstly, the low-valent nickel species **A** undergoes oxidative addition of carbamoyl chloride to form carbamoyl-Ni(II) species **B**. Then the carbamoyl-Ni(I) **C** is formed by the reduction of Mn, which then proceeds with an enantiodetermining migratory insertion into alkene to forge the intermediate **D**. Next, **D** undergoes the coupling with the alkyl iodide via a SET process to generate the alkyl-Ni(III) species **F**. The six-membered lactams **3** or **5** could obtain by reductive elimination of **F**.

In conclusion, the first enantioselective carbamoylation to access the six-membered lactam by the nickel-catalyzed asymmetric reductive cyclization was reported. The protocol using newly developed Quniox ligand allows the formation of dihydroquinolinones and δ-lactam with good yield and enantioselectivity, which can be further derived to other valuable nitrogen-containing heterocycles. The asymmetric carbamoylation that enables other chiral lactam synthesis is currently underway in our group.

## Methods

### The reaction to produce dihydroquinolinone 3

To a dried 8-mL vial were added Ni(ClO_4_)_2_·6H_2_O (15 mol%), **L8** (18 mol%), Mn (4.0 equiv) and carbamoyl chloride **1** (1.0 equiv) (if solid). Then the vial was transferred into glovebox. LiI (1.0 equiv), DMA, MeCN, carbamoyl chloride **1** (1.0 equiv) (if liquid) and alkyl halide **2** (3.0 equiv) were added in sequence inside the glovebox. The vial was then taken out from the glovebox, sealed with parafilm, put into oil bath (30 °C) and stirred for 48 h. After completion, the reaction mixture was quenched with H_2_O, filtered through a pad of Celite and extracted with EA for three times. The combined organic phase was washed with brine and concentrated under reduced pressure to yield the crude product, which was purified by silica gel flash column chromatography to afford products **3**.

### The reaction to produce δ-lactam 5

To a dried 8-mL vial were added Ni(ClO_4_)_2_·6H_2_O (15 mol%), **L11** (18 mol%), Mn (4.0 equiv). Then the vial was transferred into glovebox. LiBr (1.0 equiv), DMA, carbamoyl chloride **4** (1.0 equiv) and alkyl halide **2** (3.0 equiv) were added in sequence inside the glovebox. The vial was then taken out from the glovebox, sealed with parafilm, put into oil bath (30 °C) and stirred for 36 h. After completion, the reaction mixture was quenched with H_2_O, filtered through a pad of Celite and extracted with EA for three times. The combined organic phase was washed with brine and concentrated under reduced pressure to yield the crude product, which was purified by silica gel flash column chromatography to afford products **5**.

## Supplementary information

Supplementary Information

## Data Availability

The crystallographic data for compound **3** **y** is available from the Cambridge Crystallographic Data Center under deposition numbers CCDC 2158471 respectively (https://www.ccdc.cam.ac.uk/structures/). All other data to support the conclusions are available in the main text or the Supplementary Information. (See Supplementary Method).
