# Supplementary material for: Ni-catalyzed carbamoylation of unactivated alkenes for stereoselective construction of six-membered lactams
Source: Nat Commun. 2022 Oct 10;13:5964. doi: 10.1038/s41467-022-33425-3 (PMC9551058; doi:10.1038/s41467-022-33425-3)
Supplement: Supplementary file 1 — Supplementary Information [file 41467_2022_33425_MOESM1_ESM.pdf]

Supplementary Information

Ni-Catalyzed Carbamoylation of Unactivated Alkenes for Stereoselective Construction of Six-Membered Lactams

Chenhuan Zhang[1], Xianqing Wu[1], Tingting Xia[1], Jingping Qu[1], Yifeng Chen[1]*

[1]Key Laboratory for Advanced Materials and Joint International Research Laboratory of Precision Chemistry and Molecular Engineering, Feringa Nobel Prize Scientist Joint Research Center, Frontiers Science Center for Materiobiology and Dynamic Chemistry, School of Chemistry and Molecular Engineering, East China University of Science & Technology 130 Meilong Road, Shanghai, 200237, China.

E–mail: yifengchen@ecust.edu.cn

Table of Contents

1 Supplementary Methods 2

1.1 General Information..2
1.2 Experimental Procedures for Substrates 3
1.3 Characterization Data for Carbamoyl Chloride...5
1.4 Experimental Procedures and Characterization Data for Ligand Synthesis......................10
1.5 Effects of Additives and Solvents on δ-Lactam Synthesis. 12
1.6 Experimental Procedures and Characterization Data for Products 12
1.7 Experimental Procedures and Characterization Data for Derivatization Experiment.......42
1.8 Mechanistic Experiments..47
1.9 Crystallographic Data for Compound **3y** 49
1.10 NMR Spectroscopic Data...51

2 Supplementary References...104

1 Supplementary Methods

1.1 General Information

All reactions were carried out under nitrogen atmosphere and anhydrous conditions unless otherwise indicated. All manipulations of air-sensitive or moisture-sensitive compounds were performed in a glovebox under an atmosphere of nitrogen. Unless otherwise noted, all catalytic reactions were run in dried glassware. Toluene and THF were distilled from sodium/benzophenone. DCM was distilled over CaH_2. $Ni(ClO_4)_2 \cdot 6H_2O$ (CAS 13520-61-1) was purchased from 9dingchem. Manganese powder (CAS 7439-96-5, 325 mesh) was purchased from Alfa Aesar. LiI (CAS 10377-51-2) was purchased from Bidepharm; LiBr (CAS 7550-35-8) and $C_7H_{15}I$ (CAS 4282-40-0) were purchased from Adamas. DMA (CAS 127-19-5) and MeCN (CAS 75-05-8) were purchased from Adamas (99.8%, SafeDry, with molecular sieves, Water ≤ 50 ppm (by K.F.), SafeSeal); other commercial available reagents were purchased from Energy Chemical, Adamas-beta China and were used as received. Reactions were monitored by thin-layer chromatography (TLC) carried out on 0.20 mm Huanghai silica gel plates (HSGF 254) using UV light as the visualizing agent and iodine and $KMnO_4$ with heat as the developing agents. All new compounds were characterized by means of GC, ^{1}H-NMR, ^{13}C-NMR, and HR-MS. GC analysis was performed on SHIMADZU GC-2030. GC runs were performed with the following method: GC; HP-5 column; inlet temperature 250 °C; column temperature 100 °C for 1 min, then 50 °C/min to 280 °C, then 280 °C for 6 min. HPLC was performed on SHIMADZU LC-2030 Plus Optical rotations were recorded on digital automatic polarimeter (WZZ-2S). NMR spectra were recorded using a Bruker AVANCE Ⅲ 400 MHz NMR spectrometer and can be found at the end of the paper. High-resolution mass spectra (HRMS) were recorded on a Waters Xevo G2 TOF MS; JEOL AccuTOF LG-plus 4G; Waters GCT Premier. All ^{1}H-NMR data were reported in δ units, parts per million (ppm), and were calibrated relative to the signals for residual chloroform (7.26 ppm) in deuterochloroform ($CDCl_3$). All ^{13}C-NMR data were reported in ppm relative to $CDCl_3$ (77.16 ppm) and were obtained with ^{1}H decoupling. The following abbreviations or combinations thereof were used to explain the multiplicities: s = singlet, d = doublet, t = triplet, q = quartet, quint = quintet, sept = septet, m = multiplet.

1.2 Experimental Procedures for Substrates

Compounds **2r**,[1] **2s**,[2] **2w**,[3] **2x**,[4] **4a**,[3,5] **4b**[3,6] were synthesized according to the published procedures.

General procedure A for carbamoyl chlorides synthesis

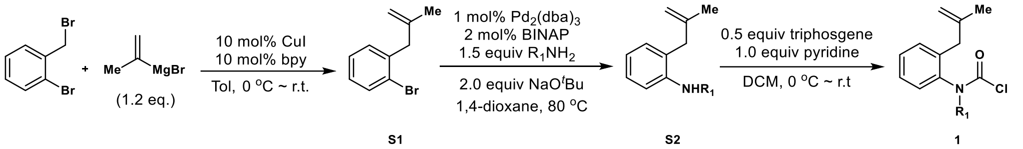

Supplementary Figure 1. Synthesis of carbomoyl chloride

To an oven-dried, round-bottomed flask charged with CuI (10 mol%), 2,2'-bipyridine (10 mol%) in toluene (0.5 M), 2-bromobenzyl bromide (1.0 equiv) was added under nitrogen flow. Then the Grignard reagent (1.2 equiv) in THF was added *via* syringe at 0 °C. The reaction was allowed to warm to room temperature and stirred for 4 h. After completion, the reaction was quenched by *sat. aq.* NH_4Cl solution, extracted with ethyl acetate (three times). The combined organic layer was washed with brine, dried over anhydrous Na_2SO_4, filtered, and concentrated under reduced pressure. The residue was purified by flash chromatography on silica gel (eluent: PE) to afford **S1** (R_f = 0.80, SiO_2, PE) as a colorless liquid (935.6 mg, 5.0 mmol, 89%).[7]

An over-dried resealable Schlenk flask was charged with $Pd_2(dba)_3$ (1 mol%), BIANP (2 mol%), sodium *tert*-butoxide (2.0 equiv), evacuated and backfilled with nitrogen for three times. Then 1,4-dioxane (0.3 M) and **S1** (1.0 equiv) were added in sequence, followed by the corresponding amine (1.5 equiv). The reaction mixture was heated to 80 °C with vigorous stirring overnight until consumption of starting material was observed by TLC. Upon completion the reaction was diluted with ethyl acetate, filtered through a pad of Celite and concentrated *in vacuo*. The crude product was purified by flash chromatography on silica gel (eluent: PE–PE/EA = 20/1) to afford **S2**.

To a solution of **S2** (1.0 equiv) in DCM (0.5 M) was added pyridine (1.0 equiv) and triphosgene (0.5 equiv) in sequence at 0 ℃. The mixture was then allowed to warm to room temperature for enough time until consumption of starting material was observed by TLC, and then the mixture was quenched with 1 M HCl, extracted with DCM for three times, dried over anhydrous Na_2SO_4, filtered. After removing the solvent, the residue was purified by column chromatography (eluent: PE/EA = 50/1–20/1) to give compound **1**.

General procedure B for carbamoyl chlorides synthesis

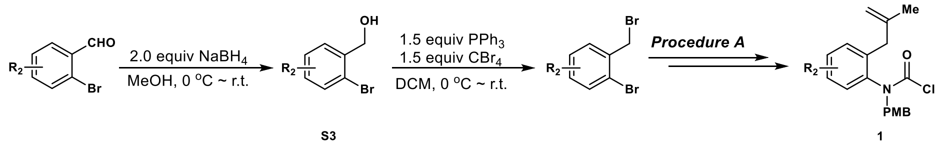

Supplementary Figure 2. Synthesis of carbomoyl chloride

To an oven-dried, round-bottomed flask charged with corresponding aldehyde (1.0 equiv) in MeOH (0.5 M) was added $NaBH_4$ (2.0 equiv) in portions slowly at 0 °C. Until

the gas released completely, the reaction mixture was allowed to warm to room temperature and stirred overnight till consumption of starting material was observed by TLC. Then the reaction mixture was concentrated. Water was added to the residue and the product was extracted with ethyl acetate for three times, dried over anhydrous Na_2SO_4, filtered. After removing the solvent, the residue was purified by column chromatography (eluent: PE/EA = 20/1–5/1) to afford **S3**.

To a mixture of CBr_4 (1.5 equiv) in DCM (0.5 M) was added PPh_3 (1.5 equiv) at 0 ^oC, then the substituted 2-bromobenzyl alcohol **S3** (1.0 equiv) was added *via* syringe. The reaction mixture was stirred at room temperature overnight. The reaction mixture was concentrated *in vacuo* and diluted with PE, the suspension was filtered through Celite and the filtrate was concentrated on a rotary evaporator. The crude product was purified by flash chromatography on silica gel (eluent: PE) to afford substituted 2-bromobenzyl bromide.

Carbamoyl chloride was then synthesized according to the general procedure A.

General procedure C for carbamoyl chlorides synthesis

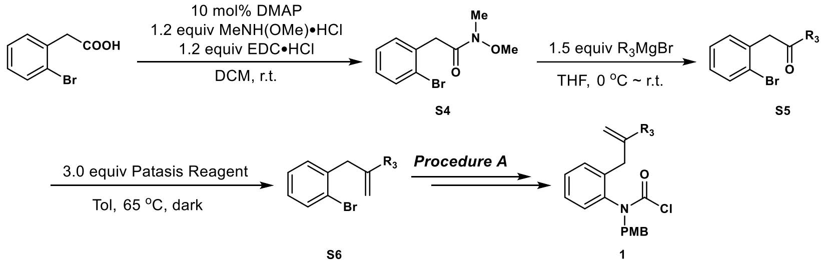

Supplementary Figure 3. Synthesis of carbomoyl chloride

A mixture of 2-bromophenylacetic acid (1.0 equiv), EDC • HCl (1.2 equiv), DMAP (10 mol%) and *N*,*O*-dimethylhydroxylamine hydrochloride (1.2 equiv) in DCM (0.5 M) was stirred at room temperature for 16 h. The reaction mixture was concentrated under vacuum, and the residue was diluted with ethyl acetate. The organic layer was washed with *sat. aq.* $NaHCO_3$ and brine, dried over Na_2SO_4, and concentrated under vacuum. The crude product was purified by column chromatography (eluent: PE/EA = 5/1) to afford 2-(2-bromophenyl)-*N*-methoxy-*N*-methylacetamide **S4** as a yellow oil.

To an oven-dried, round-bottomed flask charged with the 2-(2-bromophenyl)-*N*-methoxy-*N*-methylacetamide **S4** (1.0 equiv) in THF (1.0 M), the corresponding Grignard reagent (1.5 equiv) in THF was added dropwise *via* syringe at 0 ℃. The reaction mixture was warmed to room temperature. When the starting material was consumed as monitored by TLC, the reaction mixture was quenched by *sat. aq.* NH_4Cl solution, extracted with ethyl acetate (three times) and the combined organic layer was washed with brine, dried over anhydrous Na_2SO_4, filtered, and concentrated under reduced pressure. The residue was purified by flash chromatography on silica gel (eluent: PE/EA = 2/1) to afford **S5**.[8]

To a round-bottomed flask charged with newly prepared Cp_2TiMe (Petasis reagent) (3.0 equiv) in toluene (1.0 M), **S5** was added slowly *via* syringe at 0 ^oC. The reaction mixture was heated to 65 ^oC and stirred overnight in the dark. Until consumption of starting material was observed by TLC, the mixture was cooled to room temperature

and filtered through Celite and the filtrate was concentrated on a rotary evaporator. The crude product was purified by flash chromatography on silica gel (eluent: PE) to afford alkene **S6**.

Carbamoyl chloride was then synthesized according to the general procedure A.

1.3 Characterization Data for Carbamoyl Chloride

(4-Methoxybenzyl)(2-(2-methylallyl)phenyl)carbamic chloride (1a)

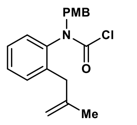

General procedure A was followed starting with 1-bromo-2-(2-methylallyl)benzene on 5.0 mmol and purification by flash column chromatography on silica gel (PE/EA = 20/1) afforded **1a** as yellow solid (1.1 g, 67% over 2 steps); **R*f***= 0.45 (PE/EA = 20/1);

^{1}H NMR (400 MHz, $CDCl_3$): δ 7.33–7.28 (m, 2H), 7.16–7.10 (m, 3H), 6.81 (d, *J* = 8.4 Hz, 2H), 6.72 (d, *J* = 8.0 Hz, 1H), 5.18 (1/2abq, *J* = 14.0 Hz, 1H), 4.93 (s, 1H), 4.73 (s, 1H), 4.24 (1/2abq, *J* = 14.0 Hz, 1H), 3.80 (s, 3H), 3.29 (1/2abq, *J* = 15.6 Hz, 1H), 3.17 (1/2abq, *J* = 15.6 Hz, 1H), 1.67 (s, 3H).

^{13}C{^{1}H} NMR (100 MHz, $CDCl_3$): δ 159.7, 150.3, 143.3, 140.4, 137.3, 131.1, 130.8, 130.0, 129.0, 127.8, 127.3, 114.1, 113.9, 55.5, 55.4, 39.4, 22.6.

HRMS (ESI): $[M+H]^+$ Calcd for $C_{19}H_{21}ClNO_2^+$: 330.1255; found: 330.1248.

(4-Methoxy-2-(2-methylallyl)phenyl)(4-methoxybenzyl)carbamic chloride (1b)

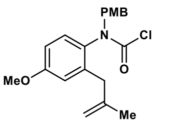

General procedure B was followed starting with 1-bromo-4-methoxy-2-(2-methylallyl)benzene on 2.0 mmol and purification by flash column chromatography on silica gel (PE/EA = 20/1) afforded **1b** as yellow oil (332.0 mg, 46% over 2 steps, contain 15% isomer); **R*f***= 0.39 (PE/EA = 20/1);

^{1}H NMR (400 MHz, $CDCl_3$): δ 7.11 (d, *J* = 8.4 Hz, 2H), 6.82–6.80 (m, 3H), 6.66–6.59 (m, 2H), 5.16 (1/2abq, *J* = 13.6 Hz, 1H), 4.94 (s, 1H), 4.75 (s, 1H), 4.21 (1/2abq, *J* = 14.0 Hz, 1H), 3.80 (s, 3H), 3.79 (s, 3H), 3.24 (1/2abq, *J* = 16.0 Hz, 1H), 3.11 (1/2abq, *J* = 15.6 Hz, 1H), 1.68 (s, 3H).

^{13}C{^{1}H} NMR (100 MHz, $CDCl_3$): δ 159.7, 150.8, 143.2, 138.7, 133.3, 131.11, 131.05, 130.9, 127.9, 115.7, 114.0, 113.9, 112.2, 55.6, 55.5, 55.4, 39.6, 22.5.

HRMS (ESI): $[M+H]^+$ Calcd for $C_{20}H_{23}ClNO_3^+$: 360.1361; found: 360.1353.

(5-Chloro-2-(2-methylallyl)phenyl)(4-methoxybenzyl)carbamic chloride (1c)

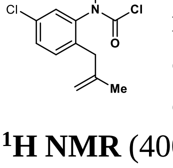

General procedure B was followed starting with 2-bromo-4-chloro-1-(2-methylallyl)benzene on 1.0 mmol and purification by flash column chromatography on silica gel (PE/EA = 50/1 – 20/1) afforded **1c** as yellow oil (163.6 mg, 45% over 2 steps); **R*f***= 0.42 (PE/EA = 20/1);

^{1}H NMR (400 MHz, $CDCl_3$): δ 7.30 (dd, *J* = 8.4, 2.0 Hz, 1H), 7.23 (d, *J* = 8.4 Hz, 1H), 7.11 (d, *J* = 8.4 Hz, 2H), 6.83 (d, *J* = 8.8 Hz, 2H), 6.77 (d, *J* = 1.2 Hz, 1H), 5.10 (1/2abq, *J* = 14.0 Hz, 1H), 4.93 (s, 1H), 4.71 (s, 1H), 4.30 (1/2abq, *J* = 14.0 Hz, 1H), 3.80 (s, 3H), 3.22 (1/2abq, *J* = 16.0 Hz, 1H), 3.07 (1/2abq, *J* = 16.0 Hz, 1H), 1.64 (s, 3H).

$^{13}C\{^1H\}$ NMR (100 MHz, $CDCl_3$): δ 159.9, 149.9, 142.8, 141.2, 136.2, 132.4, 131.8, 131.1, 129.9, 129.3, 127.3, 114.3, 114.2, 55.6, 55.5, 38.9, 22.5.
HRMS (ESI): $[M+H]^+$ Calcd for $C_{19}H_{20}Cl_2NO_2^+$: 364.0866; found: 364.0858.

(3-Fluoro-2-(2-methylallyl)phenyl)(4-methoxybenzyl)carbamic chloride (1d)

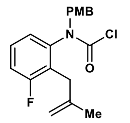

General procedure B was followed starting with 1-bromo-3-fluoro-2-(2-methylallyl)benzene on 2.0 mmol and purification by flash column chromatography on silica gel (PE/EA = 20/1) afforded **1d** as yellow oil (365.5 mg, 53% over 2 steps); **R*f*** = 0.33 (PE/EA = 20/1);
^1H NMR (400 MHz, $CDCl_3$): δ 7.12–7.08 (m, 4H), 6.82 (d, *J* = 8.4 Hz, 2H), 6.46 (dd, *J* = 5.6, 2.4 Hz, 1H), 5.32 (1/2 abq, *J* = 14.4 Hz, 1H), 4.86 (s, 1H), 4.56 (s, 1H), 4.07 (1/2abq, *J* = 14.4 Hz, 1H), 3.80 (s, 3H), 3.39 (1/2abq, *J* = 16.0 Hz, 1H), 3.30 (1/2abq, *J* = 16.0 Hz, 1H), 1.82 (s, 3H).
$^{13}C\{^1H\}$ NMR (100 MHz, $CDCl_3$): δ 161.9 (d, $J_{C–F}$ = 245.8 Hz), 159.7, 150.1, 142.5, 141.6 (d, $J_{C–F}$ = 5.8 Hz), 131.0, 127.9 (d, $J_{C–F}$ = 9.8 Hz), 127.7, 126.4 (d, $J_{C–F}$ = 3.3 Hz), 125.6 (d, $J_{C–F}$ = 17.2 Hz), 116.1 (d, $J_{C–F}$ = 22.7 Hz), 114.1, 111.9, 55.4, 55.3, 33.0 (d, $J_{C–F}$ = 2.9 Hz), 23.0.
^{19}F NMR (376 MHz, $CDCl_3$): -112.8.
HRMS (ESI): $[M+H]^+$ Calcd for $C_{19}H_{20}ClFNO_2^+$: 348.1161; found: 348.1155.

(4-Methoxybenzyl)(2-(2-methylallyl)-5-(trifluoromethyl)phenyl)carbamic chloride (1e)

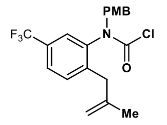

General procedure B was followed starting with 2-bromo-1-(2-methylallyl)-4-(trifluoromethyl)benzene on 0.5 mmol and purification by flash col umn chromatography on silica gel (PE/EA = 20/1) afforded **1e** as yellow oil (137.7 mg, 69% over 2 steps); **R*f*** = 0.60 (PE/EA = 20/1);
^1H NMR (400 MHz, $CDCl_3$): δ 7.57 (d, *J* = 8.0 Hz, 1H), 7.43 (d, *J* = 8.0 Hz, 1H), 7.08 (d, *J* = 8.4 Hz, 2H), 6.95 (s, 1H), 6.82 (d, *J* = 8.4 Hz, 2H), 5.16 (1/2 abq, *J* = 14.0 Hz, 1H), 4.98 (s, 1H), 4.73 (s, 1H), 4.29 (1/2abq, *J* = 14.0 Hz, 1H), 3.79 (s, 3H), 3.32 (1/2abq, *J* = 15.6 Hz, 1H), 3.18 (1/2abq, *J* = 16.0 Hz, 1H), 1.66 (s, 3H).
$^{13}C\{^1H\}$ NMR (100 MHz, $CDCl_3$): δ 159.9, 149.6, 142.2, 141.9, 140.4, 131.2, 131.0, 129.7 (q, $J_{C–F}$ = 33.0 Hz), 127.1 (q, $J_{C–F}$ = 3.7 Hz), 126.9, 125.6 (q, $J_{C–F}$ = 3.6 Hz), 123.3 (q, $J_{C–F}$ = 270.6 Hz), 114.6, 114.1, 55.4, 55.3, 39.1, 22.4.
^{19}F NMR (376 MHz, $CDCl_3$): -62.6.
HRMS (EI): $[M]^+$ Calcd for $C_{20}H_{19}ClF_3NO_2$: 397.1056; found: 397.1054.

Benzyl(2-(2-methylallyl)phenyl)carbamic chloride (1f)

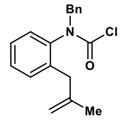

General procedure A was followed starting with 1-bromo-2-(2-methylallyl)benzene on 2.0 mmol and purificati on by flash column chromatography on silica gel (PE/EA = 20/1 – 10/1) afforded **1f** as yellow oil (183.5 mg, 31% over 2 steps); **R*f*** = 0.59 (PE/EA = 10/1);
^1H NMR (400 MHz, $CDCl_3$): δ 7.34–7.28 (m, 5H), 7.23–7.20 (m, 2H), 7.13 (td, *J* = 8.0, 2.8 Hz, 1H), 6.75 (d, *J* = 8.0 Hz, 1H), 5.25 (1/2abq, *J* = 14.0 Hz, 1H), 4.94 (s, 1H), 4.73 (s, 1H), 4.32 (1/2abq, *J* = 14.0 Hz, 1H), 3.25 (1/2abq, *J* = 15.6 Hz, 1H), 3.18

(1/2abq, J = 15.6 Hz, 1H), 1.68 (s, 3H).
^{13}C{^{1}H} NMR (100 MHz, $CDCl_3$): δ 150.4, 143.3, 140.4, 137.3, 135.6, 130.9, 129.9, 129.7, 129.1, 128.8, 128.4, 127.3, 113.9, 56.1, 39.4, 22.5.
HRMS (ESI): $[M+H]^+$ Calcd for $C_{18}H_{19}ClNO^+$: 300.1150; found: 300.1143.

Benzyl(2-(2-methylallyl)phenyl)carbamic chloride (1g)

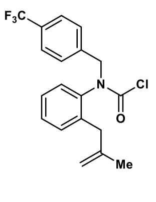

General procedure A was followed starting with 1-bromo-2-(2-methylallyl)benzene on 1.0 mmol and purification by flash column chromatography on silica gel (PE/EA = 20/1) afforded **1g** as yellow oil (184.1 mg, 50% over 2 steps); **R*f***= 0.32 (PE/EA = 20/1);
^{1}H NMR (400 MHz, $CDCl_3$): δ 7.57 (d, J = 8.0 Hz, 2H), 7.40–7.29 (m, 4H), 7.16 (td, J = 8.0, 2.4 Hz, 1H), 6.75 (d, J = 8.0 Hz, 1H), 5.29 (1/2 abq, J = 14.0 Hz, 1H), 4.94 (s, 1H), 4.70 (s, 1H), 4.35 (1/2abq, J = 14.4 Hz, 1H), 3.31 (1/2abq, J = 15.6 Hz, 1H), 3.20 (1/2abq, J = 16.0 Hz, 1H), 1.68 (s, 3H).
^{13}C{^{1}H} NMR (100 MHz, $CDCl_3$): δ 150.7, 143.2, 140.3, 139.5, 137.2, 131.3, 130.7 (q, $J_{C\text{–}F}$ = 32.1 Hz), 129.9, 129.7, 129.4, 127.6, 125.8 (q, $J_{C\text{–}F}$ = 3.7 Hz), 124.1 (q, $J_{C\text{–}F}$ = 270.6 Hz), 113.9, 55.5, 39.5, 22.6.
^{19}F NMR (376 MHz, $CDCl_3$): -62.6.
HRMS (ESI): $[M+H]^+$ Calcd for $C_{19}H_{18}ClF_3NO^+$: 368.1024; found: 368.1018.

(Furan-2-ylmethyl)(2-(2-methylallyl)phenyl)carbamic chloride (1h)

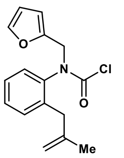

General procedure A was followed tarting with 1-bromo-2-(2-methylallyl)benzene on 2.0 mmol and purification by flash column chromatography on silica gel (PE/EA = 20/1) afforded **1h** as yellow oil (273.5 mg, 47% over 2 steps, contain 6% isomer); **R*f***= 0.53 (PE/EA = 20/1);
^{1}H NMR (400 MHz, $CDCl_3$): δ 7.38 (d, J = 1.2 Hz, 1H), 7.36–7.29 (m, 2H), 7.20 (td, J = 7.6, 2.0 Hz, 1H), 6.89 (d, J = 8.8 Hz, 1H), 6.31 (dd, J = 3.2, 2.0 Hz, 1H), 6.21 (d, J = 3.2 Hz, 1H), 5.13 (1/2 abq, J = 15.2 Hz, 1H), 4.93 (s, 1H), 4.72 (s, 1H), 4.40 (1/2abq, J = 15.2 Hz, 1H), 3.25 (abq, J = 15.6 Hz, 2H), 1.70 (s, 3H).
^{13}C{^{1}H} NMR (100 MHz, $CDCl_3$): δ 150.1, 148.9, 143.3, 142.9, 140.3, 137.5, 130.8, 129.5, 129.2, 127.5, 113.8, 110.8, 110.6, 48.3, 39.4, 22.6.
HRMS (ESI): $[M+H]^+$ Calcd for $C_{16}H_{17}ClNO_2^+$: 290.0942; found: 290.0936.

(2-(2-Methylallyl)phenyl)(2-(thiophen-2-yl)ethyl)carbamic chloride (1i)

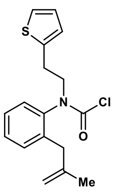

General procedure A was followed starting with 1-bromo-2-(2-methylallyl)benzene on 1.0 mmol and purification by flash column chromatography on silica gel (PE/EA = 40/1) afforded **1i** as pink solid (143.1 mg, 45% over 2 steps); **R*f***= 0.72 (PE/EA = 20/1);
^{1}H NMR (400 MHz, $CDCl_3$): δ 7.39–7.31 (m, 2H), 7.27 (td, J = 8.0, 2.0 Hz, 1H), 7.16 (dd, J = 4.8, 0.8 Hz, 1H), 6.99 (d, J = 7.6 Hz, 1H), 6.94 (dd, J = 5.2, 3.6 Hz, 1H), 6.85 (d, J = 2.8 Hz, 1H), 4.94 (s, 1H), 4.72 (s, 1H), 4.29 (dt, J = 13.2, 7.6 Hz, 1H), 3.45 (m, 1H), 3.31 (abq, J = 16.0 Hz, 2H), 3.22 (t, J = 8.0 Hz, 2H), 1.72 (s, 3H).
^{13}C{^{1}H} NMR (100 MHz, $CDCl_3$): δ 149.7, 143.2, 140.9, 139.9, 137.1, 131.2, 129.4, 129.2, 127.6, 127.2, 125.8, 124.2, 113.8, 53.9, 39.5, 27.8, 22.6.

HRMS (ESI): [M+H]$^+$ Calcd for $C_{17}H_{19}ClNOS^+$: 320.0870; found: 320.0863.

Cyclohexyl(2-(2-methylallyl)phenyl)carbamic chloride (1j)

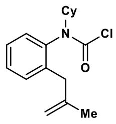

General procedure A was followed starting with 1-bromo-2-(2-methylallyl)benzene on 2.0 mmol and purification by flash column chromatography on silica gel (PE/EA = 20/1) afforded **1j** as yellow oil (402.9 mg, 69% over 2 steps); **R*f***= 0.51 (PE/EA = 20/1);

^{1}H NMR (400 MHz, $CDCl_3$): δ 7.38–7.31 (m, 2H),7.28–7.24 (m, 1H), 7.11 (d, *J* = 8.0 Hz, 1H), 4.97 (s, 1H), 4.78 (s, 1H), 4.05 (tt, *J* = 12.0, 3.2 Hz, 1H), 3.35 (abq, *J* = 16.0 Hz, 2H), 2.12 (d, *J* = 12.0 Hz, 1H), 1.91–1.78 (m, 2H), 1.71 (s, 3H), 1.60–1.52(m, 3H), 1.37–1.23 (m, 2H), 1.21–1.11 (m, 1H), 1.00 (qt, *J* = 12.8, 3.6 Hz, 1H).

^{13}C{^{1}H} NMR (100 MHz, $CDCl_3$): δ 149.1, 143.2, 139.4, 138.4, 130.4, 129.9, 128.9, 127.1, 114.3, 62.4, 39.6, 31.5, 30.2, 25.94, 25.90, 25.3, 22.6.

HRMS (ESI): [M+H]$^+$ Calcd for $C_{17}H_{23}ClNO^+$: 292.1463; found: 292.1456.

Butyl(2-(2-methylallyl)phenyl)carbamic chloride (1k)

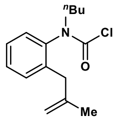

General procedure A was followed on 2.0 mmol starting with 1-bromo-2-(2-methylallyl)benzene and purification by flash column chromatography on silica gel (PE/EA = 50/1) afforded **1k** as yellow oil (322.0 mg, 61% over 2 steps); **R*f***= 0.59 (PE/EA = 20/1);

^{1}H NMR (400 MHz, $CDCl_3$): δ 7.38–7.27 (m, 3H), 7.14 (dd, *J* = 8.0, 0.8 Hz, 1H), 4.93 (s, 1H), 4.73 (s, 1H), 4.00 (ddd, *J* = 16.4, 10.4, 6.4 Hz, 1H), 3.28 (abq, *J* = 15.6 Hz, 2H), 3.19 (ddd, *J* = 15.2, 10.0, 5.2 Hz, 1H), 1.71 (s, 3H), 1.70–1.51 (m, 2H), 1.40–1.25 (m, 2H), 0.91 (t, *J* = 7.6 Hz, 3H).

^{13}C{^{1}H} NMR (100 MHz, $CDCl_3$): δ 149.6, 143.2, 140.9, 137.2, 131.0, 129.6, 129.0, 127.5, 113.8, 52.6, 39.4, 29.6, 22.6, 20.1, 13.8.

HRMS (ESI): [M+H]$^+$ Calcd for $C_{15}H_{21}ClNO$ $^+$: 266.1306; found: 266.1300.

(4-Methoxybenzyl)(2-(2-methylenebutyl)phenyl)carbamic chloride (1l)

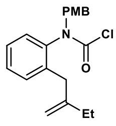

General procedure C was followed on 1.0 mmol and purification by flash column chromatography on silica gel (PE/EA = 20/1 – 10/1) afforded **1l** as yellow oil (74.4 mg, 20% over 5 steps); **R*f***= 0.55 (PE/EA = 10/1);

^{1}H NMR (400 MHz, $CDCl_3$): δ 7.33–7.28 (m, 2H), 7.15–7.11 (m, 3H), 6.82 (d, *J* = 8.8 Hz, 2H), 6.70 (d, *J* = 8.0 Hz, 1H), 5.20 (1/2 abq, *J* = 14.0 Hz, 1H), 4.95 (s, 1H), 4.71 (s, 1H), 4.21 (1/2abq, *J* = 14.0 Hz, 1H), 3.80 (s, 3H), 3.33 (1/2abq, *J* = 15.6 Hz, 1H), 3.20 (1/2abq, *J* = 16.0 Hz, 1H), 1.98 (q, *J* = 7.6 Hz, 2H), 1.05 (t, *J* = 7.6 Hz, 3H).

^{13}C{^{1}H} NMR (100 MHz, $CDCl_3$): δ 159.7, 150.3, 149.0, 140.3, 137.5, 131.1, 131.07, 130.04, 129.0, 127.8, 127.2, 114.0, 111.5, 55.42, 55.38, 38.2, 28.8, 12.4.

HRMS (ESI): [M+Na]$^+$ Calcd for $C_{20}H_{22}ClNNaO_2^+$: 366.1231; found: 366.1223.

(4-Methoxybenzyl)(3-(2-methylallyl)pyridin-2-yl)carbamic chloride (1m)

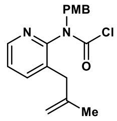

General procedure B was followed on 0.3 mmol and purification by flash column chromatography on silica gel (PE/EA = 20/1 – 5/1) afforded **1m** as yellow oil (54.7 mg, 55% over 2 steps); **R*f*** = 0.42 (PE/EA = 5/1);

^{1}H NMR (400 MHz, $CDCl_3$): δ 8.44 (d, *J* = 3.6 Hz, 1H), 7.58 (d, *J* = 7.6 Hz, 1H), 7.30–7.28 (m, 1H), 7.12 (d, *J* = 8.4 Hz, 2H), 6.77 (d, *J* = 8.8 Hz, 2H), 5.09 (1/2 abq, *J* = 14.0 Hz, 1H), 4.89 (s, 1H), 4.74 (1/2abq, *J* = 14.4 Hz, 1H), 4.64 (s, 1H), 3.76 (s, 3H), 3.18 (1/2abq, *J* = 16.4 Hz, 1H), 2.79 (1/2abq, *J* = 16.0 Hz, 1H), 1.38 (s, 3H).

^{13}C{^{1}H} NMR (100 MHz, $CDCl_3$): δ 159.7, 152.7, 149.0, 147.6, 141.8, 139.0, 133.9, 131.0, 127.3, 124.3, 115.1, 114.1, 55.4, 54.8, 38.6, 22.0.

HRMS (ESI): $[M+H]^+$ Calcd for $C_{18}H_{20}ClN_2O_2^+$: 331.1208; found: 331.1197.

1.4 Experimental Procedures and Characterization Data for Ligand Synthesis

General procedure for synthesis of amino alcohol S7

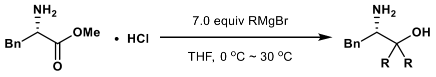

Supplementary Figure 4. Synthesis of amino alcohol

To an oven-dried, round-bottomed flask charged with *L*-phenylalanine methyl ester hydrochloride in THF (1.0 M), the corresponding Grignard reagent (7.0 equiv) in THF was added dropwise *via* syringe at 0 ℃. The mixture was then heated to 30 °C and stirred overnight. When TLC indicates that the reaction is completed, the reaction was quenched by *sat. aq.* NH_4Cl solution, extracted with ethyl acetate (three times) and the combined organic layer was washed with brine, dried over anhydrous Na_2SO_4, filtered, and concentrated under reduced pressure to give amino alcohol **S7** as a yellow oil. The crude produce was used without further purification.

General procedure for synthesis of chiral quinoline-oxazoline ligands

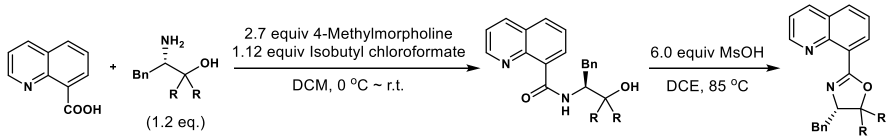

Supplementary Figure 5. Synthesis of chiral quinoline-oxazoline ligands

To a round-bottomed flask charged with 8-carboxyquinoline in DCM (0.2 M), 4-methylmorpholine (2.7 equiv) was added slowly, followed by the addition of isobutyl chloroformate (1.12 equiv) in a dropwise manner at 0 °C (DCM can be added appropriately if the 8-carboxyquinoline was insoluble). Then the amino alcohol **S7** (1.2 equiv) was added *via* syringe. The mixture was warmed to room temperature and stirred overnight till consumption of starting material was observed by TLC. The reaction mixture was quenched by H_2O, extracted with DCM (three times) and the combined organic layer was washed with brine, dried over anhydrous Na_2SO_4, filtered, and concentrated under reduced pressure. The crude product was purified by flash chromatography on silica gel (PE/EA = 5/1–2/1) to afford amide **S8** as white solid.

To a solution of amide **S8** in fresh distilled DCE was added MsOH (6.0 equiv) *via* syringe, the mixture was then heated to 85 °C for required time. Upon completion, the reaction was quenched by *sat. aq.* $NaHCO_3$ solution, and diluted with DCM immediately, extracted with DCM for three times. The combined organic layer was washed with brine, dried over anhydrous Na_2SO_4, filtered, and concentrated under reduced pressure. The yellow oil was purified by flash chromatography on Al_2O_3 (PE/Acetone = 20/1–5/1) to afford a white solid (*Note: 1. The quinoline-oxazoline ligands are acid-sensitive; 2. Further washing by PE could be taken up to obtain a fluffy white solid if the character is not very well*).

(*S*)-4-benzyl-5,5-dimethyl-2-(quinolin-8-yl)-4,5-dihydrooxazole (L8)

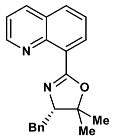

General procedure was followed starting with (*S*)-*N*-(3-hydroxy-3-methyl-1-phenylbutan-2-yl)quinoline-8-carboxamide on 3.0 mmol and purification flash chromatography on Al_2O_3 (PE/Acetone = 20/1–5/1) to afford **L8** as white solid (602.4 mg, 64%); **R*f*** = 0.43 (PE/EA = 5/1); $[\alpha]_D^{25}$ = - 70.59 (c = 0.17, $CHCl_3$).

^{1}H NMR (400 MHz, $CDCl_3$ with K_2CO_3): δ 9.06 (dd, *J* = 4.0, 1.6 Hz, 1H), 8.16 (dd, *J* = 8.4, 1.2 Hz, 1H), 8.07 (dd, *J* = 7.2, 1.2 Hz, 1H), 7.90 (d, *J* = 7.2 Hz, 1H), 7.56 (t, *J* = 7.6 Hz, 1H), 7.43 (dd, *J* = 8.0, 4.0 Hz, 1H), 7.38 (d, *J* = 7.2 Hz, 2H), 7.32 (t, *J* = 7.6 Hz, 2H), 7.21 (t, *J* = 7.2 Hz, 1H), 4.44 (dd, *J* = 8.4, 7.2 Hz, 1H), 3.45 (dd, *J* = 14.4, 6.8 Hz, 1H), 2.92 (dd, *J* = 14.4, 8.4 Hz, 1H), 1.50 (s, 3H), 1.46 (s, 3H).

^{13}C{^{1}H} NMR (100 MHz, $CDCl_3$ with K_2CO_3): δ 162.6, 151.5, 146.5, 139.4, 136.3, 131.4 130.8, 129.3, 128.8, 128.54, 128.52, 126.3, 125.8, 121.5, 86.9, 75.8, 37.6 28.5, 22.1.

HRMS (ESI): $[M+H]^+$ Calcd for $C_{21}H_{21}N_2O^+$: 317.1648; found: 317.1641.

(*S*)-4-benzyl-5,5-diphenyl-2-(quinolin-8-yl)-4,5-dihydrooxazole (L9)

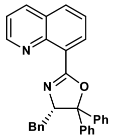

General procedure was followed on starting with (*S*)-*N*-(1-hydroxy-1,1,3-triphenylpropan-2-yl)quinoline-8-carboxamide 1.0 mmol and purification flash chromatography on Al_2O_3 (PE/Acetone = 20/1–5/1) to afford **L9** as white solid (189.8 mg, 43%); **R*f*** = 0.38 (PE/EA = 5/1); $[\alpha]_D^{25}$ = - 208.33 (c = 0.24, $CHCl_3$).

^{1}H NMR (400 MHz, $CDCl_3$ with K_2CO_3): δ 9.13 (d, *J* = 2.8 Hz, 1H), 8.18 (t, *J* = 8.8 Hz, 2H), 7.94 (d, *J* = 8.0 Hz, 1H), 7.61–7.52 (m, 5H), 7.48 (dd, *J* = 8.1, 4.4 Hz, 1H), 7.41–7.32 (m, 6H), 7.25–7.15 (m, 3H), 7.10 (d, *J* = 7.2 Hz, 1H), 5.37 (t, *J* = 6.8 Hz, 1H), 2.93 (dd, *J* = 14.0, 8.0 Hz, 1H), 2.73 (dd, *J* = 14.0, 6.0 Hz, 1H).

^{13}C{^{1}H} NMR (100 MHz, $CDCl_3$ with K_2CO_3): δ 162.3, 151.3, 146.5, 144.5, 141.0, 139.2, 136.2, 131.6, 131.0, 129.4, 128.5, 128.4, 128.2, 127.91, 127.86, 127.8, 127.6, 126.8, 126.1, 125.9, 121.6, 93.1, 77.6, 40.7.

HRMS (ESI): $[M+H]^+$ Calcd for $C_{31}H_{25}N_2O^+$: 441.1961; found: 441.1951.

1.5 Effects of Additives and Solvents on δ-Lactam Synthesis.

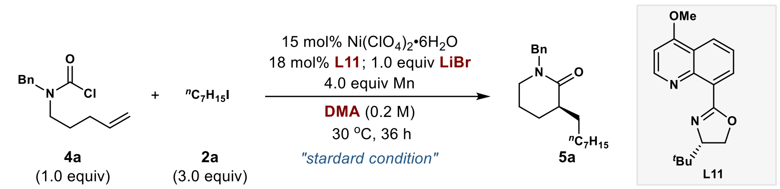

Entry	Modification	TM (%)	er
1	standard condition	82	93.5:6.5
2	DMA/MeCN = 4:1	70	93.5:6.5
3	LiI instead of LiBr	81	93.5:6.5
4	DMA/MeCN = 4:1<br>LiI instead of LiBr	81	92.5:7.5

Supplementary Figure 6. Effects of additives and solvents

1.6 Experimental Procedures and Characterization Data for Products

General procedure D for asymmetric synthesis of 3,4-Dihydroquinolinones *via* Ni-catalyzed reductive cross coupling

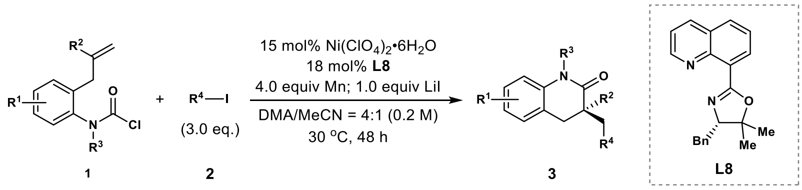

Supplementary Figure 7. Synthesis of dihydroquinolinones

To a dried 8-mL vial were added $Ni(ClO_4)_2 \cdot 6H_2O$ (15 mol%), **L8** (18 mol%), Mn (4.0 equiv) and carbamoyl chloride **1** (1.0 equiv) (if solid). Then the vial was transferred into glovebox. LiI (1.0 equiv), DMA, MeCN, carbamoyl chloride **1** (1.0 equiv) and alkyl halide (3.0 equiv) were added in sequence inside the glovebox. The vial was then taken out from the glovebox, sealed with parafilm, put into oil bath (30 °C) and stirred for 48 h. After completion, the reaction mixture was quenched with H_2O, filtered through a pad of Celite and extracted with EA for three times. The combined organic phase was washed with brine and concentrated under reduced pressure to yield the crude product, which was purified by silica gel flash column chromatography to afford products **3**.

General procedure E for asymmetric synthesis of *δ*-lactam *via* Ni-catalyzed reductive cross coupling

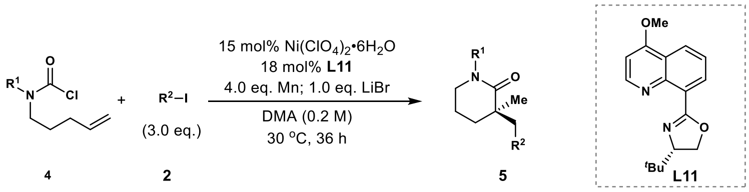

Supplementary Figure 8. Synthesis of δ-lactam

To a dried 8-mL vial were added $Ni(ClO_4)_2 \cdot 6H_2O$ (15 mol%), **L11** (18 mol%), Mn (4.0 equiv) and carbamoyl chloride **4** (1.0 equiv) (if solid). Then the vial was transferred into glovebox. LiBr (1.0 equiv), DMA, carbamoyl chloride **4** (1.0 equiv) (if liquid) and alkyl halide (3.0 equiv) were added in sequence inside the glovebox. The vial was then taken out from the glovebox, sealed with parafilm, put into oil bath (30 ^{o}C) and stirred for 36 h. After completion, the reaction mixture was quenched with H_2O, filtered through a pad of Celite and extracted with EA for three times. The combined organic phase was washed with brine and concentrated under reduced pressure to yield the crude product, which was purified by silica gel flash column chromatography to afford products **5**.

(*S*)-1-(4-methoxybenzyl)-3-methyl-3-octyl-3,4-dihydroquinolin-2(1H)-one (3a)

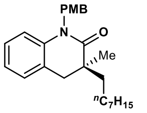

General procedure D was followed on 0.2 mmol and purification flash chromatography on silica gel (PE/EA = 40/1–20/1) to afford **3a** as yellow oil (67.1 mg, 85%); **R*f*** = 0.45 (PE/EA = 10/1); $[\alpha]_D^{25}$ = + 9.07 (c = 0.25, $CHCl_3$).

^{1}H NMR (400 MHz, $CDCl_3$): δ 7.14–7.10 (m, 4H), 6.96 (t, *J* = 8.4 Hz, 1H), 6.87 (d, *J* = 7.6 Hz, 1H), 6.83 (d, *J* = 8.8 Hz, 2H), 5.20 (1/2abq, *J* = 15.6 Hz, 1H), 4.97 (1/2abq, *J* = 16.0 Hz, 1H), 3.77 (s, 2H), 2.83 (abq, *J* = 15.6 Hz, 2H), 1.58–1.45 (m, 2H), 1.39–1.24 (m, 15H), 0.87 (t, *J* = 6.4 Hz, 3H).

^{13}C{^{1}H} NMR (100 MHz, $CDCl_3$): δ 175.1, 158.7, 139.7, 129.7, 128.5, 127.7, 127.3, 125.1, 122.8, 115.0, 114.2, 55.4, 46.1, 40.7, 38.2, 36.6, 32.0, 30.3, 29.6, 29.4, 24.1, 22.8, 22.6, 14.2.

HRMS (ESI): $[M+H]^+$ Calcd for $C_{26}H_{36}NO_2^+$: 394.2741; found: 394.2732.

HPLC (Chiralpak AD-H): *n*-Hexane/*i*-PrOH = 90/10, flow rate 1.0 mL/min, λ = 220 nm, t_R= 6.915 min (major), t_R= 7.532 min (minor); 96.5:3.5 e.r.

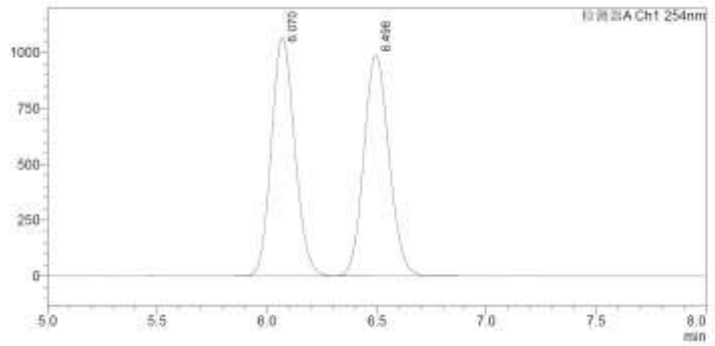

检测器A Ch1 254nm

Peak#	Ret. Time	Area	Height	Conc.	Unit	Mark
1	6.070	7869452	1063078	50.067		M
2	6.496	7848508	986921	49.933		M

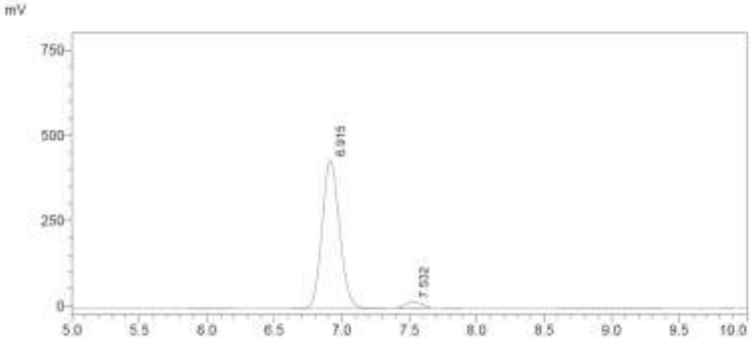

检测器A Ch1 220nm

Peak#	Ret. Time	Area	Height	Conc.	Unit	Mark
1	6.915	3909051	435131	96.688		M
2	7.532	133887	17052	3.312		M

Supplementary Figure 9. HPLC data of **3a**

(*S*)-6-Methoxy-1-(4-methoxybenzyl)-3-methyl-3-octyl-3,4-dihydroquinolin-2(*1H*)-one (3b)

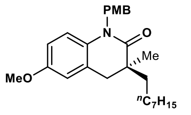

General procedure D was followed on 0.2 mmol and purification flash chromatography on silica gel (PE/EA = 50/1–20/1) to afford **3b** as yellow oil (77.6 mg, 92%); **R*f*** = 0.47 (PE/EA = 10/1); $[\alpha]_D^{25}$ = - 1.90 (c = 6.15, $CHCl_3$).

^{1}H NMR (400 MHz, $CDCl_3$): δ 7.12 (d, *J* = 8.8 Hz, 2H), 6.83 (d, *J* = 8.4 Hz, 2H), 6.78 (d, *J* = 8.8 Hz, 1H), 6.70 (d, *J* = 3.2 Hz, 1H), 6.63 (dd, *J* = 8.8, 3.2 Hz, 1H), 5.17 (1/2 abq, *J* = 15.6 Hz, 1H), 4.94 (1/2 abq, *J* = 16.0 Hz, 1H), 3.76 (s, 3H), 3.75 (s, 3H), 2.79 (s, 2H), 1.58–1.44 (m, 2H), 1.43–1.24 (m, 15H), 0.87 (t, *J* = 6.4 Hz, 3H).

^{13}C{^{1}H} NMR (100 MHz, $CDCl_3$): δ 174.5, 158.6, 155.3, 133.2, 129.8, 127.6, 126.6, 115.8, 114.6, 114.2, 111.5, 55.5, 55.3, 46.1, 40.6, 38.4, 36.6, 32.0, 30.3, 29.6, 29.4, 24.0 ,22.7, 22.5, 14.2.

HRMS (ESI): $[M+H]^+$ Calcd for $C_{27}H_{38}NO_3^+$: 424.2846; found: 424.2834.

HPLC (Chiralpak AD-H): *n*-Hexane/*i*-PrOH = 90/10, flow rate 1.0 mL/min, λ = 254 nm, t_R= 10.137 min (major), t_R= 15.184 min (minor); 96.5:3.5 e.r.

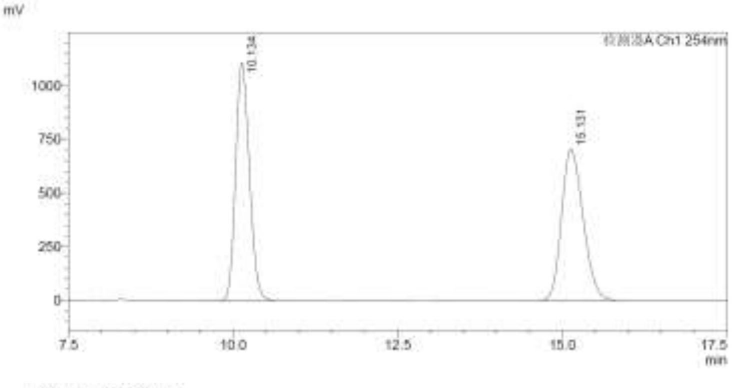

检测器A Ch1 254nm

Peak#	Ret. Time	Area	Height	Conc.	Unit	Mark	Name
1	10.134	15976449	1108600	49.974		M	
2	15.131	15992834	705335	50.026		M	
总计		31969282	1813935				

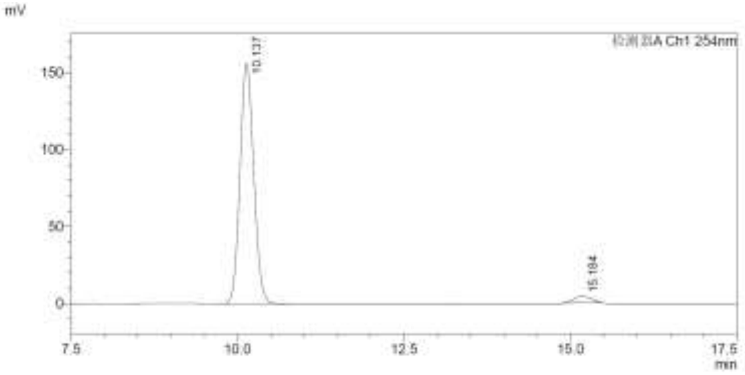

检测器A Ch1 254nm

Peak#	Ret. Time	Area	Height	Conc.	Unit	Mark	Name
1	10.137	2232045	156021	96.581		M	
2	15.184	79015	4255	3.419		M	
总计		2311060	160277				

Supplementary Figure 10. HPLC data of **3b**

(*S*)-7-chloro-1-(4-methoxybenzyl)-3-methyl-3-octyl-3,4-dihydroquinolin-2(*1H*)-one (3c)

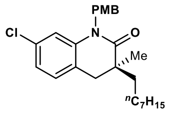

General procedure D was followed on 0.2 mmol and purification flash chromatography on silica gel (PE/EA = 40/1–20/1) to afford **3c** as yellow oil (62.7 mg, 73%); **R*f***= 0.52 (PE/EA = 10/1) ; $[\alpha]_D^{25}$ = +9.95 (c = 0.69, $CHCl_3$).

^{1}H NMR (400 MHz, $CDCl_3$): δ 7.11 (d, *J* = 8.8 Hz, 2H), 7.04 (d, *J* = 8.0 Hz, 1H), 6.93 (dd, *J* = 8.0, 2.0 Hz, 1H), 6.87–6.83 (m, 3H), 5.15 (1/2abq, *J* = 16.0 Hz, 1H), 4.92 (1/2 abq, *J* = 16.0 Hz, 1H), 3.78 (s, 3H), 2.79 (abq, *J* = 15.6 Hz, 2H), 1.55–1.42 (m, 2H), 1.33–1.23 (m, 15H), 0.87 (t, *J* = 6.4 Hz, 3H).

^{13}C{^{1}H} NMR (100 MHz, $CDCl_3$): δ 174.9, 158.9, 140.8, 132.9, 129.4, 129.0, 127.7, 123.5, 122.7, 115.3, 114.4, 55.4, 46.2, 40.6, 37.6, 36.6, 32.0, 30.3, 29.6, 29.4, 24.0, 22.8, 22.5, 14.2.

HRMS (ESI): $[M+H]^+$ Calcd for $C_{26}H_{35}ClNO_2^+$: 428.2351; found: 428.2343.

HPLC (Chiralpak AD-H): *n*-Hexane/*i*-PrOH = 90/10, flow rate 1.0 mL/min, λ = 220 nm, t_R= 5.765 min (major), t_R= 6.814 min (minor); 96.5:3.5 e.r.

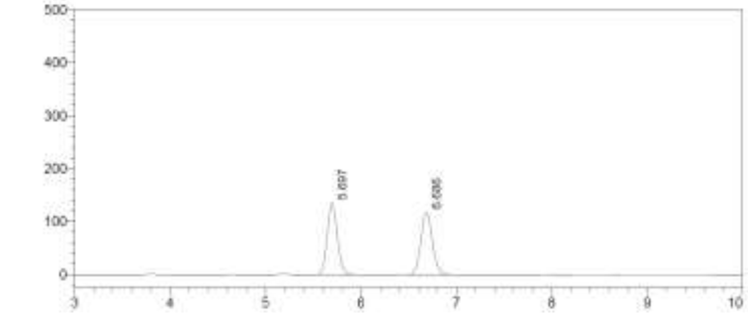

检测器A Ch1 254nm

Peak#	Ret. Time	Area	Height	Conc.	Unit	Mark
1	5.697	981766	134412	49.968		M
2	6.686	983021	117283	50.032		M

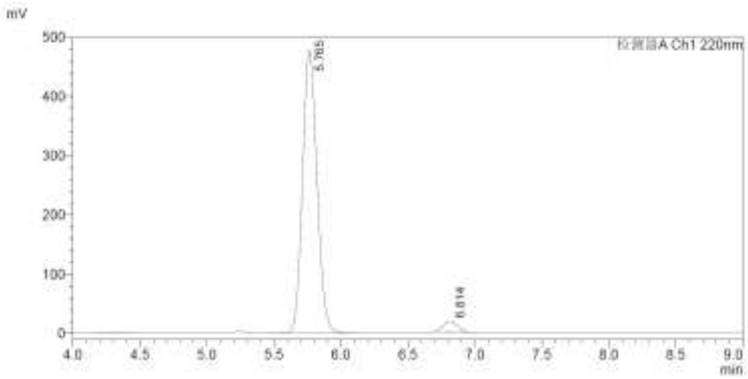

Peak#	Ret. Time	Area	Height	Conc.	Unit	Mark	Name
1	5.765	3584660	478401	96.402		M	
2	6.814	133808	17460	3.598		M	
总计		3718468	495860				

Supplementary Figure 11. HPLC data of **3c**

(*S*)-5-fluoro-1-(4-methoxybenzyl)-3-methyl-3-octyl-3,4-dihydroquinolin-2(*1H*)-one (3d)

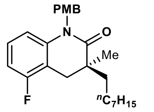

General procedure D was followed on 0.2 mmol and purification flash chromatography on silica gel (PE/EA = 40/1–10/1) to afford **3d** as yellow oil (68.8 mg, 84%); **R*f*** = 0.45 (PE/EA = 10/1); $[\alpha]_D^{25}$ = + 10.83 (c = 0.46, $CHCl_3$).

^{1}H NMR (400 MHz, $CDCl_3$): δ 7.11 (d, *J* = 8.4 Hz, 2H), 7.06 (m, 1H), 6.83 (d, *J* = 8.4 Hz, 2H), 6.74 (t, *J* = 8.4 Hz, 1H), 6.67 (d, *J* = 8.4 Hz, 1H), 5.19 (1/2abq, *J* = 16.0 Hz, 1H), 4.96 (1/2 abq, *J* = 16.0 Hz, 1H), 3.77 (s, 3H), 2.97 (1/2abq, *J* = 16.0 Hz, 1H), 2.75 (1/2 abq, *J* = 16.0 Hz, 1H), 1.58–1.46 (m, 2H), 1.38–1.24 (m, 15H), 0.87 (t, *J* = 6.4 Hz, 3H).

^{13}C{^{1}H} NMR (100 MHz, $CDCl_3$): δ 174.7, 160.4 (d, $J_{C\text{–}F}$ = 241.5 Hz), 158.8, 141.4 (d, $J_{C\text{–}F}$ = 6.8 Hz), 129.3, 127.9 (d, $J_{C\text{–}F}$ = 9.4 Hz). 127.6, 114.2, 112.2 (d, $J_{C\text{–}F}$ = 21.5 Hz), 110.7 (d, $J_{C\text{–}F}$ = 3.2 Hz), 109.9 (d, $J_{C\text{–}F}$ = 22.2 Hz), 55.3, 46.3, 40.1, 36.8, 32.0, 30.2, 30.0 (d, $J_{C\text{–}F}$ = 3.0 Hz), 29.5, 29.3, 24.0, 22.75, 22.67, 14.2.

^{19}F NMR (376 MHz, $CDCl_3$): -119.0.

HRMS (ESI): $[M+H]^+$ Calcd for $C_{26}H_{35}FNO_2^+$: 412.2646; found: 412.2637.

HPLC (Chiralpak AD-H): *n*-Hexane/*i*-PrOH = 90/10, flow rate 1.0 mL/min, λ = 220 nm, t_R= 6.785 min (major), t_R= 8.526 min (minor); 95:5 e.r.

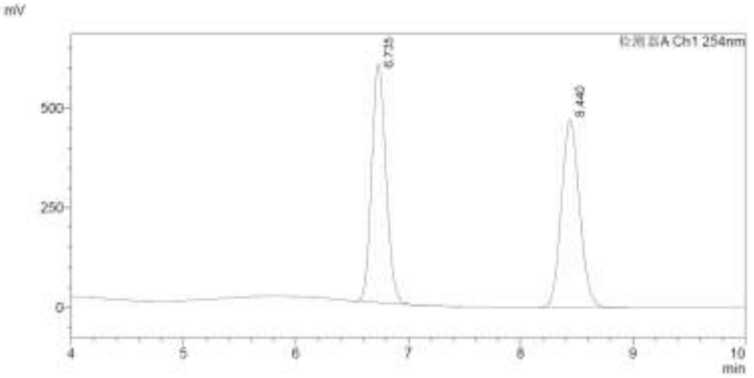

检测器A Ch1 254nm

Peak#	Ret. Time	Area	Height	Conc.	Unit	Mark	Name
1	6.735	5248341	598322	49.875		M	
2	8.440	5274579	468992	50.125		M	
总计		10522920	1067314				

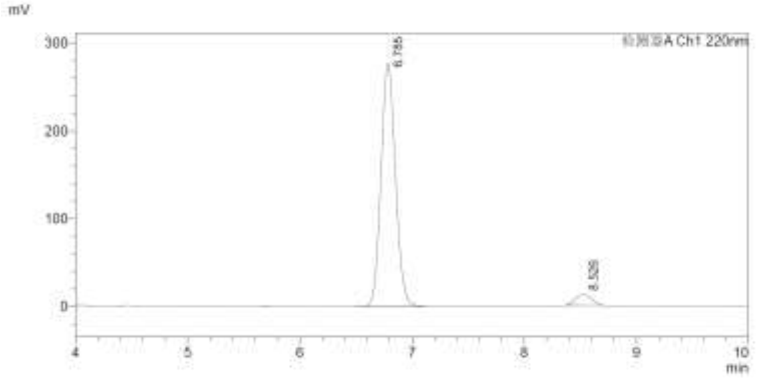

检测器A Ch1 220nm

Peak#	Ret. Time	Area	Height	Conc.	Unit	Mark	Name
1	6.785	2461820	276511	95.096		M	
2	8.526	126945	12648	4.904		M	
总计		2588765	289159				

Supplementary Figure 12. HPLC data of **3d**

(*S*)-1-(4-methoxybenzyl)-3-methyl-3-octyl-7-(trifluoromethyl)-3,4-dihydroquinolin-2(1H)-one (3e)

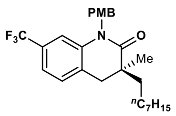

General procedure D was followed on 0.1 mmol and purification flash chromatography on silica gel (PE/EA = 50/1–10/1) to afford **3e** as pale yellow oil (24.9 mg, 69%); **R*f***= 0.45 (PE/EA = 10/1); $[\alpha]_D^{25}$ = + 22.80 (c = 0.50, $CHCl_3$).

^{1}H NMR (400 MHz, $CDCl_3$): δ 7.24–7.20 (m, 2H), 7.15–7.13 (m 3H), 6.84 (d, *J* = 8.8 Hz, 2H), 5.19 (1/2abq, *J* = 15.6 Hz, 1H), 5.00 (1/2abq, *J* = 15.6 Hz, 1H), 3.77 (s, 2H), 2.87 (abq, *J* = 16.0 Hz, 2H), 1.55–1.45 (m, 2H), 1.33–1.23 (m, 15H), 0.86 (t, *J* = 6.4 Hz, 3H).

^{13}C{^{1}H} NMR (100 MHz, $CDCl_3$): δ174.8, 158.9, 140.0, 129.8 (q, $J_{C–F}$ = 32.2 Hz), 129.0, 128.90, 128.86, 127.9, 123.8 (q, $J_{C–F}$ = 270.5 Hz), 119.6 (q, $J_{C–F}$ = 3.8 Hz), 114.4, 111.6 (q, $J_{C–F}$ = 3.8 Hz), 55.4, 46.1, 40.5, 38.0, 36.6, 32.0, 30.2, 29.6, 29.4, 24.0, 22.8, 22.5, 14.2.

^{19}F NMR (376 MHz, $CDCl_3$): -62.5.

HRMS (ESI): $[M+Na]^+$ Calcd for $C_{27}H_{34}F_3NNaO_2^+$: 484.2434; found: 484.2436.

HPLC (Chiralpak AD-H): *n*-Hexane/*i*-PrOH = 90/10, flow rate 1.0 mL/min, λ = 254 nm, t_R= 4.893 min (major), t_R= 5.414 min (minor); 97:3 e.r.

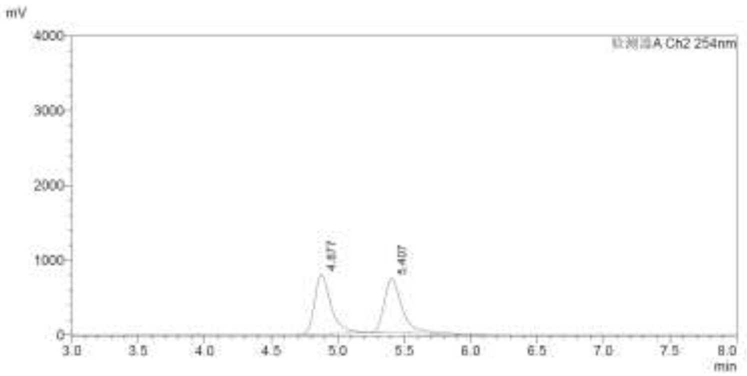

检测器A Ch2 254nm

Peak#	Ret. Time	Area	Height	Conc.	Unit	Mark	Name
1	4.877	6794009	797183	49.612		M	
2	5.407	6900159	725709	50.388		M	
总计		13694168	1522892				

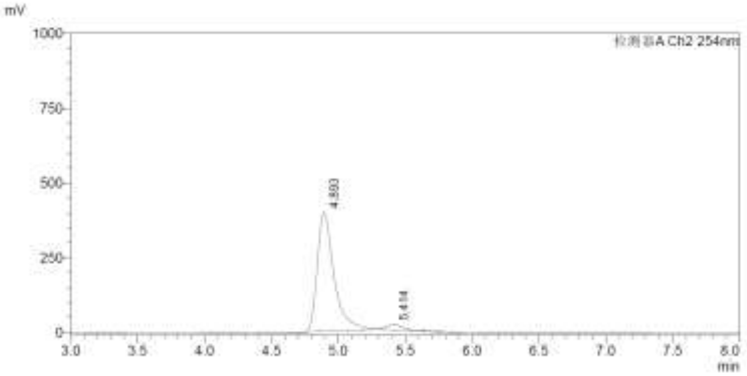

检测器A Ch2 254nm

Peak#	Ret. Time	Area	Height	Conc.	Unit	Mark	Name
1	4.893	3466528	398811	96.834		M	
2	5.414	113333	18363	3.166		M	
总计		3579861	417174				

Supplementary Figure 13. HPLC data of **3e**

(*S*)-1-benzyl-3-methyl-3-octyl-3,4-dihydroquinolin-2(1H)-one (3f)

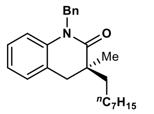

General procedure D was followed on 0.2 mmol and purification flash chromatography on silica gel (PE/EA = 40/1–10/1) to afford **3f** as pale yellow oil (47.9 mg, 66%); $\mathbf{R_f}$= 0.4 (PE/EA = 10/1); $[\alpha]_D^{25}$ = + 3.04 (c = 0.69, $CHCl_3$).

^{1}H NMR (400 MHz, $CDCl_3$): δ 7.31 (m, 2H), 7.25 (m 1H), 7.19 (d, *J* = 7.2 Hz, 2H), 7.16–7.10 (m, 2H), 6.98 (t, *J* = 7.6 Hz, 1H), 6.84 (d, *J* = 8.0 Hz, 1H), 5.30 (1/2abq, *J* = 16.0 Hz, 1H), 5.04 (1/2abq, *J* = 16.4 Hz, 1H), 2.87 (s, 2H), 1.61–1.47 (m, 2H), 1.43–1.25 (m, 15H), 0.89 (t, *J* = 6.8 Hz, 3H).

^{13}C{^{1}H} NMR (100 MHz, $CDCl_3$): δ 175.1, 139.6, 137.6, 128.8, 128.5, 127.3, 127.0, 126.3, 125.1, 122.9, 115.0, 46.7, 40.7, 38.2, 36.6, 32.0, 30.3, 29.6, 29.4, 24.1, 22.8, 22.6, 14.2.

HRMS (ESI): $[M+H]^+$ Calcd for $C_{25}H_{34}NO^+$: 364.2635; found: 364.2626.

HPLC (Chiralpak AD-H): *n*-Hexane/*i*-PrOH = 90/10, flow rate 1.0 mL/min, λ = 254 nm, t_R= 5.358 min (major), t_R= 5.775 min (minor); 97:3 e.r.

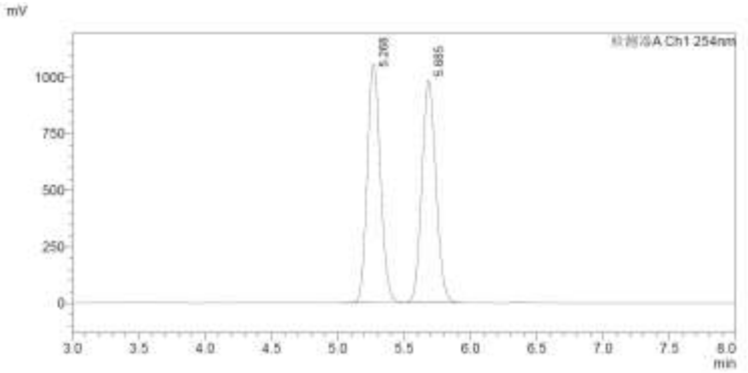

检测器A Ch1 254nm

Peak#	Ret. Time	Area	Height	Conc.	Unit	Mark	Name
1	5.268	7231418	1060039	49.970		M	
2	5.685	7240003	985672	50.030		M	
总计		14471421	2045711				

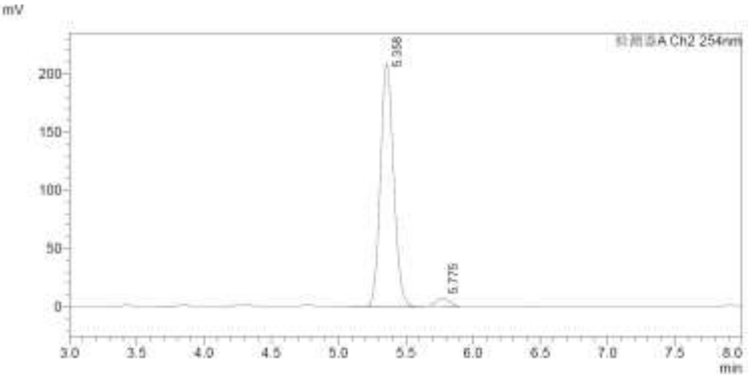

检测器A Ch2 254nm

Peak#	Ret. Time	Area	Height	Conc.	Unit	Mark	Name
1	5.358	1425836	208647	96.863		M	
2	5.775	46175	6975	3.137		M	
总计		1472011	215622				

Supplementary Figure 14. HPLC data of **3f**

(*S*)-3-methyl-3-octyl-1-(4-(trifluoromethyl)benzyl)-3,4-dihydroquinolin-2(1H)-one (3g)

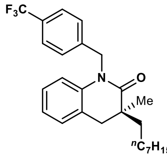

General procedure D was followedon 0.2 mmol and purification flash chromatography on silica gel (PE/EA = 40/1–10/1) to afford **3g** as yellow oil (50.4 mg, 58%); **R*f*** = 0.57 (PE/EA = 10/1); $[\alpha]_D^{25}$ = + 15.83 (c = 0.16, $CHCl_3$).

^{1}H NMR (400 MHz, $CDCl_3$): δ 7.56 (d, *J* = 8.4 Hz, 2H), 7.30 (d, *J* = 8.0 Hz, 2H), 7.16 (d, *J* = 7.2 Hz, 1H), 7.12 (t, *J* = 8.0 Hz, 1H), 7.00 (t, *J* = 7.6 Hz, 1H), 6.75 (d, *J* = 8.0 Hz, 1H), 5.31 (1/2abq, *J* = 16.4 Hz, 1H), 5.07 (1/2abq, 16.8 Hz, 1H), 2.87 (abq, *J* = 15.6 Hz, 2H), 1.59–1.47 (m, 2H), 1.42–1.24 (m, 15H), 0.87 (t, *J* = 6.4 Hz, 3H).

^{13}C{^{1}H} NMR (100 MHz, $CDCl_3$): δ 175.2, 141.8, 139.3, 129.5 (q, J_{C-F} = 32.3 Hz), 128.7, 127.5, 126.6, 125.8 (q, J_{C-F} = 3.7 Hz), 125.1, 124.2 (q, J_{C-F} = 270.3 Hz), 123.2, 114.6, 46.4, 40.8, 38.0, 36.6, 32.0, 30.3, 29.6, 29.4, 24.1, 22.8, 22.5, 14.2.

^{19}F NMR (376 MHz, $CDCl_3$): -62.5.

HRMS (ESI): $[M+H]^+$ Calcd for $C_{26}H_{33}F_3NO^+$: 432.2509; found: 432.2499.

HPLC (Chiralpak OD-H): *n*-Hexane/*i*-PrOH = 97/3, flow rate 1.0 mL/min, λ = 220 nm, t_R= 7.201 min (major), t_R= 11.529 min (minor); 96:4 e.r.

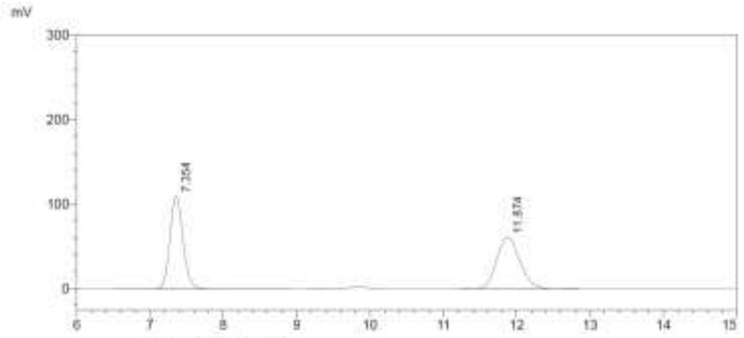

检测器A Ch1 220nm

Peak#	Ret. Time	Area	Height	Conc.	Unit	Mark
1	7.354	1338587	109501	50.025		M
2	11.874	1337260	60965	49.975		M

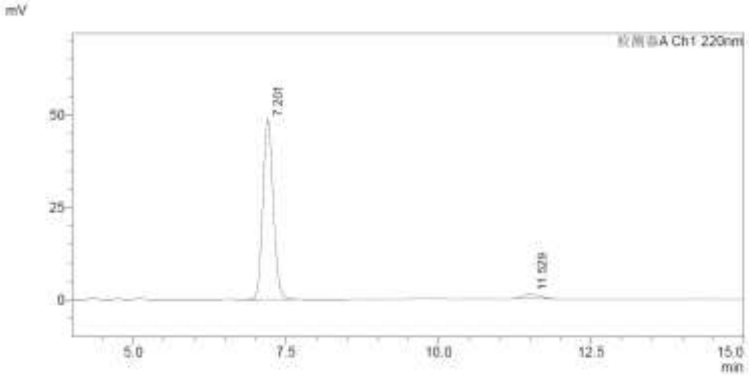

检测器A Ch1 220nm

Peak#	Ret. Time	Area	Height	Conc.	Unit	Mark	Name
1	7.201	576351	48531	96.130		M	
2	11.529	23204	1226	3.870		M	
总计		599555	49758				

Supplementary Figure 15. HPLC data of **3g**

(*S*)-1-(furan-2-ylmethyl)-3-methyl-3-octyl-3,4-dihydroquinolin-2(1H)-one (3h)

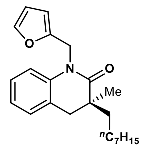

General procedure D was followed on 0.2 mmol and purification flash chromatography on silica gel (PE/EA = 40/1–20/1) to afford **3h** as yellow oil (66.6 mg, 94%); **R*f*** = 0.58 (PE/EA = 10/1); $[\alpha]_D^{25}$ = + 22.22 (c = 0.12, $CHCl_3$).

^{1}H NMR (400 MHz, $CDCl_3$): δ 7.32 (dd, *J* = 2.0, 0.8 Hz, 1H), 7.21 (td, *J* = 8.4, 1.6 Hz, 1H), 7.13–7.11 (m, 2H), 6.99 (td, *J* = 7.2, 0.8 Hz, 1H), 6.29 (dd, *J* = 3.2, 2.0 Hz, 1H), 6.19 (dd, *J* = 3.2, 0.4 Hz, 1H), 5.22 (1/2abq, *J* = 16.0 Hz, 1H), 4.94 (1/2abq, 16.0 Hz, 1H), 2.80 (s, *J* = 2H), 1.49–1.39 (m, 2H), 1.35–1.20 (m, 15H), 0.86 (t, *J* = 7.2 Hz, 3H).

^{13}C{^{1}H} NMR (100 MHz, $CDCl_3$): δ 174.8, 151.2, 141.7, 139.5, 128.5, 127.4, 125.1, 123.0, 114.7, 110.6, 107.7, 40.6, 40.3, 38.1, 36.4, 32.0, 30.2, 29.6, 29.3, 24.0, 22.8, 22.4, 14.2.

HRMS (ESI): $[M+H]^+$ Calcd for $C_{23}H_{32}NO_2^+$: 354.2428; found: 354.2419.

HPLC (Chiralpak AD-H): *n*-Hexane/EtOH = 98/2, flow rate 1.0 mL/min, λ = 254 nm, t_R= 5.134 min (major), t_R= 5.758 min (minor); 98:2 e.r.

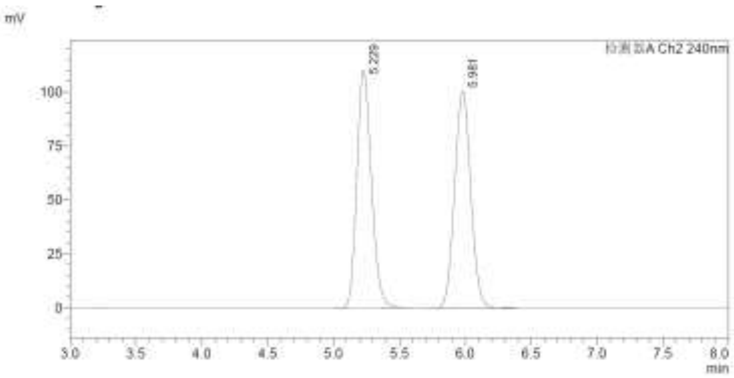

检测器A Ch1 220nm

Peak#	Ret. Time	Area	Height	Conc.	Unit	Mark	Name
1	5.230	1504676	195520	49.714		M	
2	5.981	1522017	179057	50.286		M	
总计		3026693	374576				

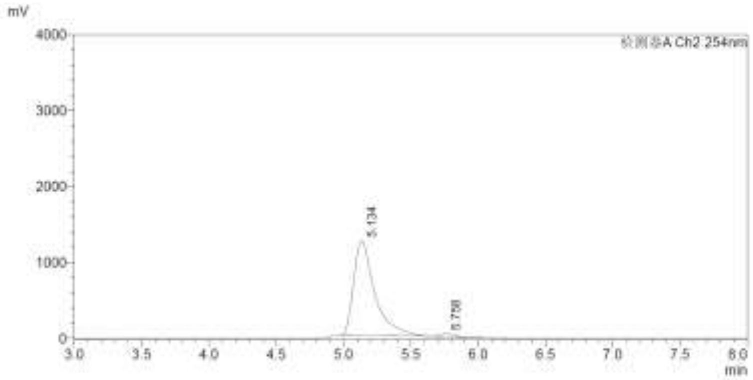

检测器A Ch2 254nm

Peak#	Ret. Time	Area	Height	Conc.	Unit	Mark	Name
1	5.134	13478148	1244193	97.774		M	
2	5.758	306907	37738	2.226		M	
总计		13785055	1281931				

Supplementary Figure 16. HPLC data of **3h**

(*S*)-3-methyl-3-octyl-1-(2-(thiophen-2-yl)ethyl)-3,4-dihydroquinolin-2(1H)-one (3i)

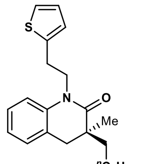

General procedure D was followed on 0.2 mmol and purification flash chromatography on silica gel (PE/EA = 40/1–20/1) to afford **3i** as yellow oil (58.6 mg, 77%); **R*f***= 0.73 (PE/EA = 10/1); $[\alpha]_D^{25}$ = + 21.75 (c = 0.19, $CHCl_3$).

^{1}H NMR (400 MHz, $CDCl_3$): δ 7.24 (t, *J* = 8.4 Hz, 1H), 7.15 (t, *J* = 4.0 Hz, 2H), 7.00 (t, *J* = 7.6 Hz, 1H), 6.96–6.88 (m, 3H), 4.27–4.20 (m, 1H), 4.15–4.08 (m, 1H), 3.21–3.10 (m, 2H), 2.75 (abq, *J* = 15.6 Hz, 2H), 1.48–1.37 (m, 2H), 1.30–1.22 (m, 12H), 1.17 (s, 3H), 0.87 (t, *J* = 6.4 Hz, 3H).

^{13}C{^{1}H} NMR (100 MHz, $CDCl_3$): δ 174.8, 140.9, 139.0, 128.8, 127.4, 127.1, 125.42, 125.39, 123.9, 122.8, 114.0, 44.4, 40.5, 38.0, 36.5, 32.0, 30.2, 29.5, 29.3, 27.6, 24.0, 22.8, 22.5, 14.2.

HRMS (ESI): $[M+H]^+$ Calcd for $C_{24}H_{34}NOS^+$: 384.2356; found: 384.2346.

HPLC (Chiralpak AD-H): *n*-Hexane/*i*-PrOH = 90/10, flow rate 1.0 mL/min, λ = 254 nm, t_R= 4.439 min (major), t_R= 4.929 min (minor); 97:3 e.r.

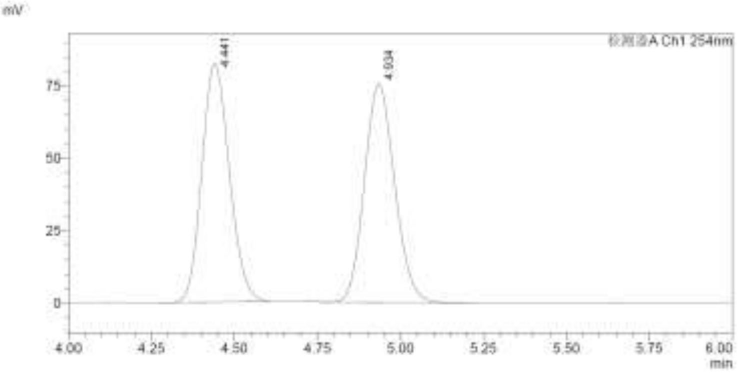

检测器A Ch1 254nm

Peak#	Ret. Time	Area	Height	Conc.	Unit	Mark	Name
1	4.441	476589	82553	49.946		M	
2	4.934	477615	75502	50.054		M	
总计		954204	158055				

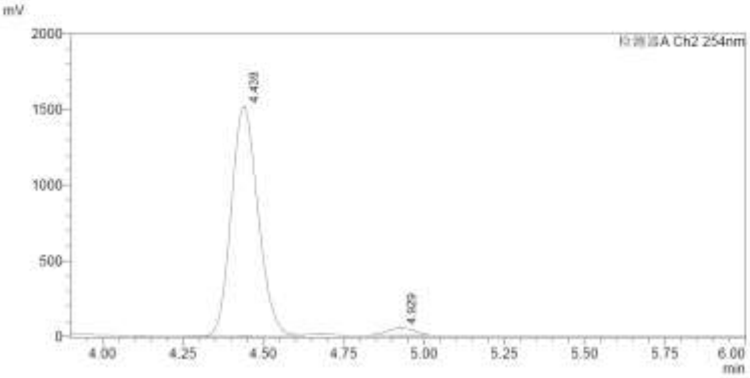

检测器A Ch2 254nm

Peak#	Ret. Time	Area	Height	Conc.	Unit	Mark	Name
1	4.439	8723609	1514045	96.975		M	
2	4.929	272091	47316	3.025		M	
总计		8995700	1561361				

Supplementary Figure 17. HPLC data of **3i**

(*S*)-1-cyclohexyl-3-methyl-3-octyl-3,4-dihydroquinolin-2(1H)-one (3j)

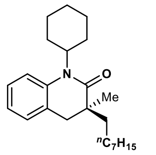

General procedure D was followed on 0.2 mmol and purification flash chromatography on silica gel (PE/EA = 40/1–20/1) to afford **3j** as yellow oil (65.0 mg, 92%); **R*f***= 0.78 (PE/EA = 10/1); $[\alpha]_D^{25}$ = + 30.51 (c = 0.26, $CHCl_3$).

^{1}H NMR (400 MHz, $CDCl_3$): δ 7.21 (t, *J* = 7.2 Hz, 1H), 7.09 (d, *J* = 8.0 Hz, 2H), 6.97 (t, *J* = 7.2 Hz, 1H), 4.09 (tt, *J* = 12.0, 3.6 Hz, 1H), 2.68 (abq, *J* = 14.8 Hz, 2H), 2.42 (quintd, *J* = 12.4, 3.2 Hz, 2H), 1.87–1.67 (m, 5H), 1.42–1.20 (m, 17H), 1.09 (s, 3H), 0.86 (t, *J* = 6.8 Hz, 3H).

^{13}C{^{1}H} NMR (100 MHz, $CDCl_3$): δ 175.6, 140.3, 128.4, 127.0, 126.9, 122.7, 115.6, 58.4, 41.4, 38.0, 36.1, 31.9, 30.2, 30.0, 29.50, 29.47, 29.3, 26.8, 26.7, 25.7, 24.0, 22.7, 22.4, 14.2.

HRMS (ESI): $[M+H]^+$ Calcd for $C_{24}H_{38}NO^+$: 356.2948; found: 356.2940.

HPLC (Chiralpak AD-H): *n*-Hexane/*i*-PrOH = 97/3, flow rate 0.5 mL/min, λ = 254 nm, t_R= 7.307 min (minor), t_R= 7.645 min (major); 95.5:4.5 e.r.

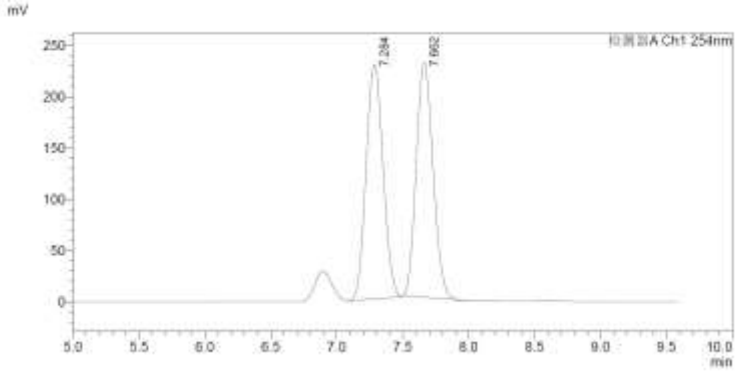

检测器A Ch1 254nm

Peak#	Ret. Time	Area	Height	Conc.	Unit	Mark	Name
1	7.284	2045089	228474	50.161		M	
2	7.662	2031959	228494	49.839		M	
总计		4077048	456968				

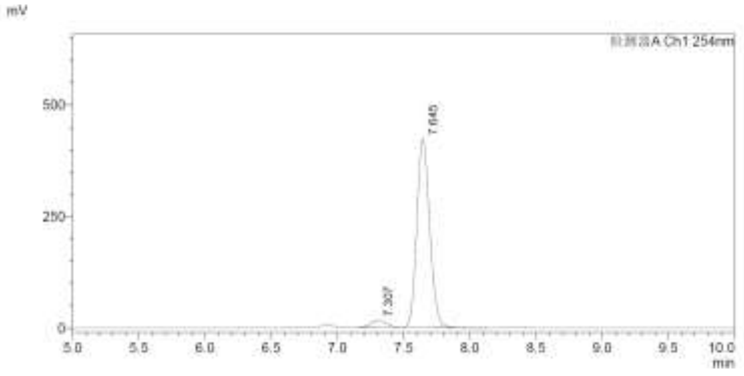

检测器A Ch1 254nm

Peak#	Ret. Time	Area	Height	Conc.	Unit	Mark	Name
1	7.307	140722	16514	4.681		M	
2	7.645	2865740	423510	95.319		M	
总计		3006461	440024				

Supplementary Figure 18. HPLC data of **3j**

(*S*)-1-butyl-3-methyl-3-octyl-3,4-dihydroquinolin-2(1H)-one (3k)

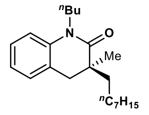

General procedure D was followed on 0.2 mmol and purification flash chromatography on silica gel (PE/EA = 40/1) to afford **3k** as yellow oil (60.3 mg, 92%); **R*f*** = 0.47 (PE/EA = 20/1); $[\alpha]_D^{25}$ = + 8.42 (c = 1.94, $CHCl_3$).

^{1}H NMR (400 MHz, $CDCl_3$): δ 7.22 (t, *J* = 7.2 Hz, 1H), 7.11 (d, *J* = 6.8 Hz, 1H), 6.97 (t, *J* = 7.2 Hz, 1H), 6.94 (d, *J* = 8.0 Hz, 1H), 3.98–3.91 (m, 1H), 3.88–3.81 (m, 1H), 2.74 (abq, *J* = 15.6 Hz, 2H), 1.61 (quint, *J* = 8.0 Hz, 2H), 1.46–1.35 (m, 4H), 1.34–1.21 (m, 12H), 1.15 (s, 3H), 0.96 (t, *J* = 7.2 Hz, 3H), 0.86 (t, *J* = 6.8 Hz, 3H).

^{13}C{^{1}H} NMR (100 MHz, $CDCl_3$): δ 174.7, 139.3, 128.6, 127.3, 125.3, 122.5, 114.1, 42.5, 40.4, 38.1, 36.4, 31.9, 30.2, 29.52, 29.47, 29.3, 24.0, 22.7, 22.5, 20.3, 14.2, 14.0.

HRMS (ESI): $[M+H]^+$ Calcd for $C_{22}H_{36}NO^+$: 330.2791; found: 330.2784.

HPLC (Chiralpak OD-H): *n*-Hexane/*i*-PrOH = 95/5, flow rate 1.0 mL/min, λ = 254 nm, t_R= 3.880 min (major), t_R= 4.136 min (minor); 97.5:2.5 e.r.

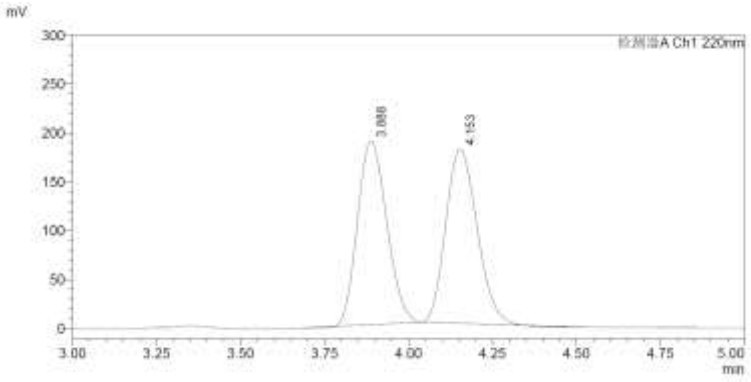

检测器A Ch1 220nm

Peak#	Ret. Time	Area	Height	Conc.	Unit	Mark	Name
1	3.888	1139365	187938	49.672		M	
2	4.153	1154417	178427	50.328		M	
总计		2293782	366365				

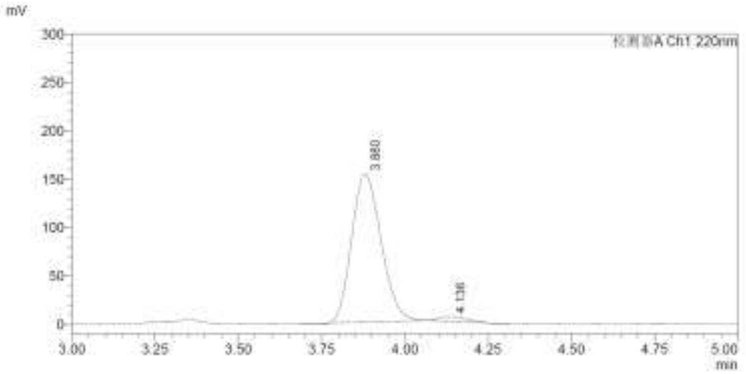

检测器A Ch1 220nm

Peak#	Ret. Time	Area	Height	Conc.	Unit	Mark	Name
1	3.880	937463	152840	97.302		M	
2	4.136	25998	4686	2.698		M	
总计		963461	157526				

Supplementary Figure 19. HPLC data of **3k**

(*S*)-3-ethyl-1-(4-methoxybenzyl)-3-octyl-3,4-dihydroquinolin-2(1H)-one (3l)

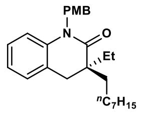

General procedure D was followed on 0.1 mmol and purification flash chromatography on silica gel (PE/EA = 40/1–20/1) to afford **3l** as yellow oil (24.9 mg, 60%); **R*f***= 0.50 (PE/EA = 10/1); $[\alpha]_D^{25}$ = + 23.19 (c = 0.23, $CHCl_3$).

^{1}H NMR (400 MHz, $CDCl_3$): δ 7.13–7.08 (m, 4H), 6.95 (t, *J* = 7.6 Hz, 1H). 6.86–6.81 (m, 3H), 5.08 (s, 2H), 3.77 (s, 3H), 2.84 (s, 2H) 1.69–1.63 (m, 2H), 1.55 (t, *J* = 8.4 Hz, 2H), 1.33–1.24 (m, 12H), 0.91–0.85 (m, 6H).

^{13}C{^{1}H} NMR (100 MHz, $CDCl_3$): δ 174.2, 158.7, 139.4, 129.8, 128.6, 127.7, 127.2, 125.1, 122.8, 114.8, 114.2, 55.4, 46.0, 43.9, 35.3, 34.0, 32.0, 30.4, 29.7, 29.4, 27.1, 24.0, 22.8, 14.3, 8.4.

HRMS (ESI): $[M+H]^+$ Calcd for $C_{27}H_{38}NO_2^+$: 408.2897; found: 408.2889.

HPLC (Chiralpak AD-H): *n*-Hexane/EtOH = 97/3, flow rate 1.0 mL/min, λ = 254 nm, t_R= 5.889 min (major), t_R= 6.915 min (minor); 98:2 e.r.

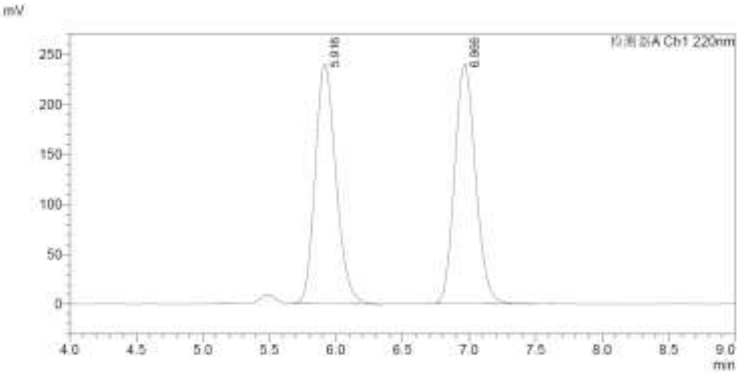

检测器A Ch1 220nm

Peak#	Ret. Time	Area	Height	Conc.	Unit	Mark	Name
1	5.916	2610006	240074	50.097		M	
2	6.966	2599905	239061	49.903		M	
总计		5209910	479134				

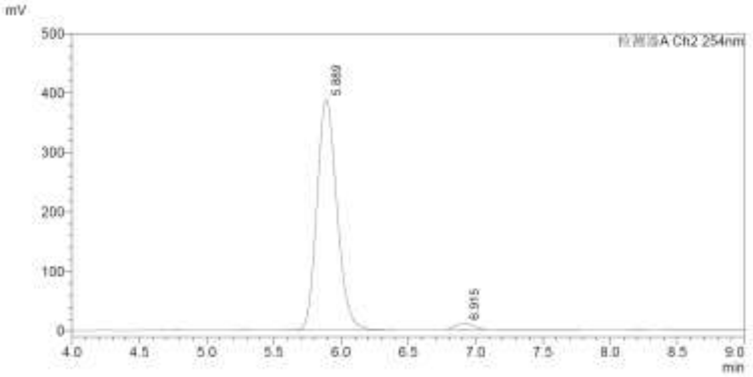

检测器A Ch2 254nm

Peak#	Ret. Time	Area	Height	Conc.	Unit	Mark	Name
1	5.889	3999660	388188	97.832		M	
2	6.915	88627	9975	2.168		M	
总计		4088287	398163				

Supplementary Figure 20. HPLC data of **3l**

(*S*)-1-(4-methoxybenzyl)-3-methyl-3-propyl-3,4-dihydroquinolin-2(1H)-one (3n)

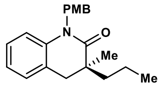

General procedure D was followed on 0.2 mmol and purification flash chromatography on silica gel (PE/EA = 40/1–10/1) to afford **3n** as yellow oil (63.1 mg, 98%); **R*f***= 0.33 (PE/EA = 10/1); $[\alpha]_D^{25}$ = + 20.48 (c = 0.37, $CHCl_3$).

^{1}H NMR (400 MHz, $CDCl_3$): δ 7.14–7.10 (m, 4H), 6.96 (t, *J* = 7.2 Hz, 1H), 6.88 (d, *J* = 8.0 Hz, 1H), 6.84 (d, *J* = 8.4 Hz, 2H), 5.20 (1/2 abq, *J* = 15.6 Hz, 1H), 4.99 (1/2 abq, *J* = 15.6 Hz, 1H), 3.77 (s, 3H), 2.84 (abq, *J* = 16.0 Hz, 2H), 1.58–1.45 (m, 2H), 1.43–1.28 (m, 2H), 1.25 (s, 3H), 0.88 (t, *J* = 7.2 Hz, 3H).

^{13}C{^{1}H} NMR (100 MHz, $CDCl_3$): δ 175.0, 158.7, 139.6, 129.7, 128.5, 127.6, 127.3, 125.1, 122.8, 114.9, 114.2, 55.3, 46.0, 40.7, 38.9, 38.1, 22.6, 17.4, 14.7.

HRMS (ESI): $[M+H]^+$ Calcd for $C_{21}H_{26}NO_2^+$: 324.1958; found: 324.1950.

HPLC (Chiralpak AD-H): *n*-Hexane/*i*-PrOH = 90/10, flow rate 1.0 mL/min, λ = 220 nm, t_R= 8.792 min (major), t_R= 9.556 min (minor); 96:4 e.r.

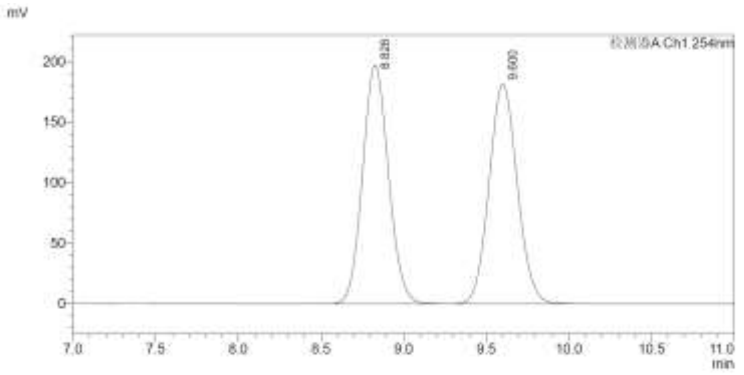

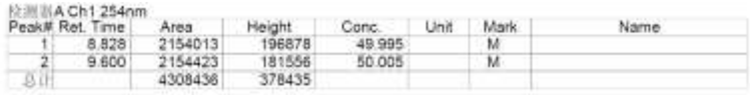

检测器A Ch1 254nm

Peak#	Ret. Time	Area	Height	Conc.	Unit	Mark	Name
1	8.828	2154013	196878	49.995		M	
2	9.600	2154423	181556	50.005		M	
总计		4308436	378435				

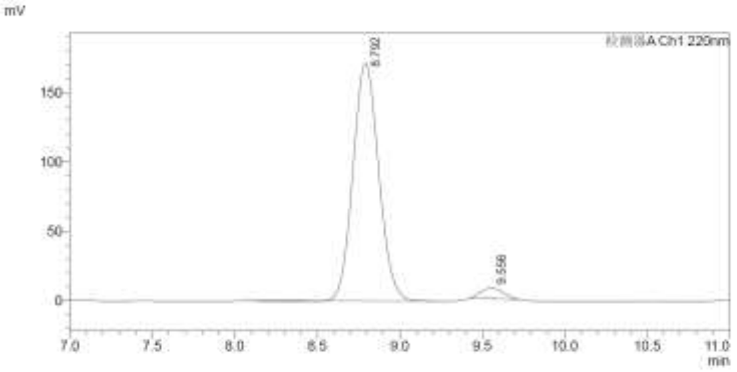

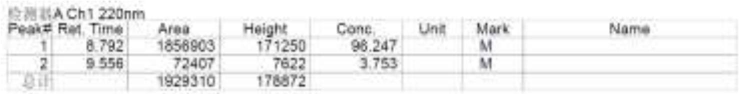

检测器A Ch1 220nm

Peak#	Ret. Time	Area	Height	Conc.	Unit	Mark	Name
1	8.792	1856903	171250	96.247		M	
2	9.556	72407	7622	3.753		M	
总计		1929310	178872				

Supplementary Figure 21. HPLC data of **3n**

(*S*)-1-(4-methoxybenzyl)-3-methyl-3-(4-methylpentyl)-3,4-dihydroquinolin-2(1H)-one (3o)

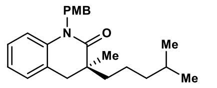

General procedure D was followed on 0.2 mmol and purification flash chromatography on silica gel (PE/EA = 30/1–2/1) to afford **3o** as yellow oil (63.3 mg, 87%); **R*f***= 0.42 (PE/EA = 10/1); $[\alpha]_D^{25}$ = + 17.59 (c = 0.36, $CHCl_3$).

^{1}H NMR (400 MHz, $CDCl_3$): δ 7.14–7.10 (m, 4H), 6.96 (t, *J* = 7.6 Hz, 1H), 6.88 (d, *J* = 8.0 Hz, 1H), 6.83 (d, *J* = 8.8 Hz, 2H), 5.23 (1/2 abq, *J* = 16.0 Hz, 1H), 4.95 (1/2 abq, *J* = 16.0 Hz, 1H), 3.77 (s, 3H), 2.84 (s, 2H), 1.57–1.42 (m, 3H), 1.39–1.28 (m, 2H), 1.26 (s, 3H), 1.16–1.06 (m, 2H), 0.84 (dd, *J* = 6.4, 1.2 Hz, 6H)

^{13}C{^{1}H} NMR (100 MHz, $CDCl_3$): δ 175.0, 158.7, 139.7, 129.7, 128.5, 127.6, 127.3, 125.1, 122.8, 115.0, 114.2, 55.3, 46.1, 40.7, 39.6, 38.3, 36.8, 27.9, 22.8, 22.64, 22.59, 21.9.

HRMS (ESI): $[M+H]^+$ Calcd for $C_{24}H_{32}NO_2^+$: 366.2428; found: 366.2422.

HPLC (Chiralpak AD-H): *n*-Hexane/EtOH = 98/2, flow rate 1.0 mL/min, λ = 240 nm, t_R= 10.174 min (major), t_R= 12.833 min (minor); 95.5:4.5 e.r.

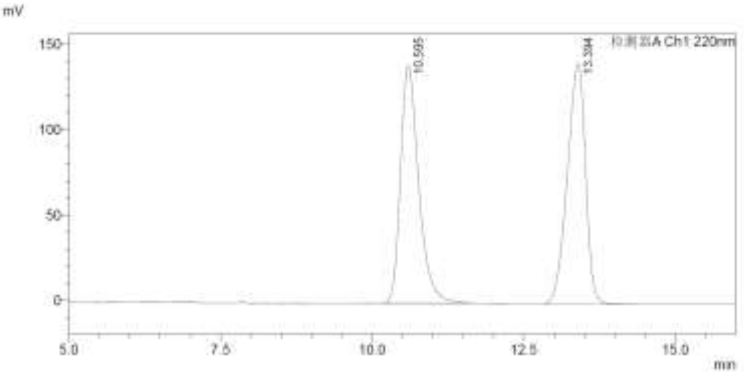

检测器A Ch1 220nm

Peak#	Ret. Time	Area	Height	Conc.	Unit	Mark	Name
1	10.595	2886039	138937	49.702		M	
2	13.394	2920604	140448	50.298		M	
总计		5806642	279385				

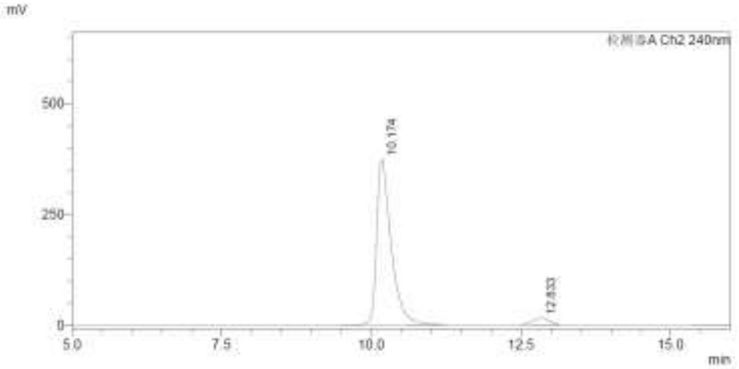

检测器A Ch2 240nm

Peak#	Ret. Time	Area	Height	Conc.	Unit	Mark	Name
1	10.174	6258006	374002	95.644		M	
2	12.833	285021	15239	4.356		M	
总计		6543027	389240				

Supplementary Figure 22. HPLC data of **3o**

(*S*)-3-(4-chlorobutyl)-1-(4-methoxybenzyl)-3-methyl-3,4-dihydroquinolin-2(1H)-one (3p)

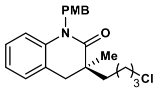

General procedure D was followed on 0.2 mmol and purification flash chromatography on silica gel (PE/EA = 40/1–20/1) to afford **3p** as yellow oil (52.5 mg, 71%); **R*f*** = 0.72 (PE/EA = 3/1); $[\alpha]_D^{25}$ = + 5.24 (c = 4.4, $CHCl_3$).

^{1}H NMR (400 MHz, $CDCl_3$): δ 7.14–7.11 (m, 4H), 6.97 (t, *J* = 7.2 Hz, 1H), 6.89 (d, *J* = 8.0 Hz, 1H), 6.84 (d, *J* = 8.8 Hz, 2H), 5.15 (1/2abq, *J* = 16.0 Hz, 1H), 5.03 (1/2abq, *J* = 16.0 Hz, 1H), 3.76 (s, 3H), 3.55–3.45 (m, 2H), 2.85 (abq, *J* = 15.6 Hz, 2H), 1.73 (quint, *J* = 6.8 Hz, 2H), 1.60–1.47 (m, 4H), 1.25 (s, 3H).

^{13}C{^{1}H} NMR (100 MHz, $CDCl_3$): δ 174.7, 158.7, 139.5, 129.5, 128.5, 127.6, 127.4, 124.8, 122.9, 115.0, 114.2, 55.3, 46.1, 44.9, 40.6, 37.9, 35.9, 33.1, 22.5, 21.5.

HRMS (ESI): $[M+H]^+$ Calcd for $C_{22}H_{27}ClNO_2^+$: 372.1725; found: 372.1715.

HPLC (Chiralpak OD-H): *n*-Hexane/EtOH = 90/10, flow rate 1.0 mL/min, λ = 220 nm, t_R= 7.682 min (major), t_R= 9.335 min (minor); 96:4 e.r.

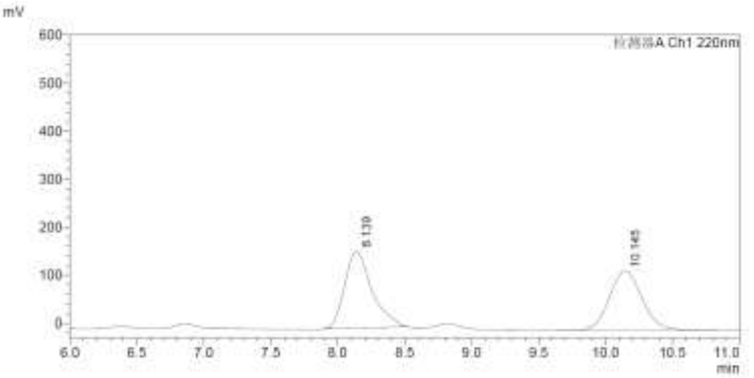

A Ch1 220nm

Peak#	Ret. Time	Area	Height	Conc.	Unit	Mark	Name
1	8.139	2269780	160884	52.361		M	
2	10.145	2065128	123087	47.639		M	
总计		4334908	283971				

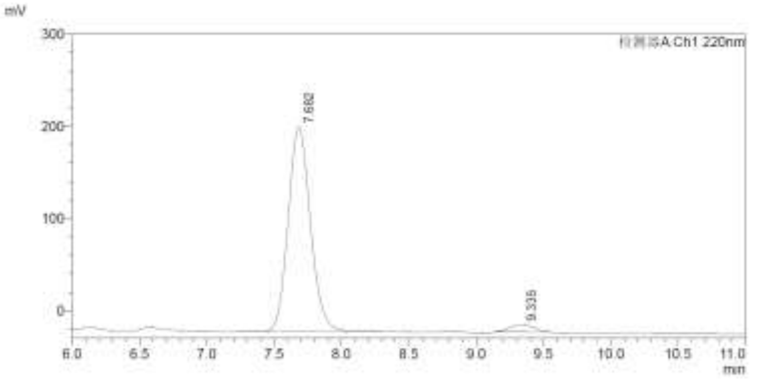

A Ch1 220nm

Peak#	Ret. Time	Area	Height	Conc.	Unit	Mark	Name
1	7.682	2538775	221638	96.195		M	
2	9.335	100418	7995	3.805		M	
总计		2639193	229633				

Supplementary Figure 23. HPLC data of **3p**

(*S*)-3-(4-fluorobutyl)-1-(4-methoxybenzyl)-3-methyl-3,4-dihydroquinolin-2(1H)-one (3q)

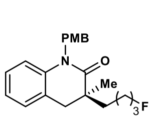

General procedure D was followed on 0.2 mmol and purification flash chromatography on silica gel (PE/EA = 30/1–10/1) to afford **3q** as yellow oil (68.0 mg, 96%); **R*f*** = 0.26 (PE/EA = 10/1); $[\alpha]_D^{25}$ = + 11.11 (c = 0.27, $CHCl_3$).

^{1}H NMR (400 MHz, $CDCl_3$): δ 7.15–7.11 (m, 4H), 6.97 (t, *J* = 7.6 Hz, 1H), 6.90 (d, *J* = 8.0 Hz, 1H), 6.84 (d, *J* = 8.8 Hz, 2H), 5.16 (1/2 abq, *J* = 16.0 Hz, 1H), 5.03 (1/2abq, *J* = 16.0 Hz, 1H), 4.47 (t, *J* = 6.0 Hz, 1H), 4.35 (t, *J* = 6.0 Hz, 1H), 3.76 (s, 3H), 2.85 (abq, *J* = 15.6 Hz, 2H), 1.72–1.58 (m, 4H), 1.56–1.42 (m, 2H), 1.25 (s, 3H).

^{13}C{^{1}H} NMR (100 MHz, $CDCl_3$): δ 174.7, 158.7, 139.4, 129.5, 128.5, 127.6, 127.4, 124.8, 122.9, 115.0, 114.2, 83.9 (d, $J_{C\text{–}F}$ = 163.6 Hz), 55.3, 46.0, 40.6, 37.9, 36.3, 30.9 (d, $J_{C\text{–}F}$ = 19.5 Hz), 22.5, 19.9 (d, $J_{C\text{–}F}$ = 5.3 Hz).

^{19}F NMR (376 MHz, $CDCl_3$): -218.1 (sept, *J* = 25.2 Hz).

HRMS (ESI): $[M+H]^+$ Calcd for $C_{22}H_{27}FNO_2^+$: 356.2020; found: 356.2010.

HPLC (Chiralpak OD-H): *n*-Hexane/EtOH = 90/10, flow rate 1.0 mL/min, λ = 254 nm,

t_R= 8.055 min (major), t_R= 8.982 min (minor); 95:5 e.r.

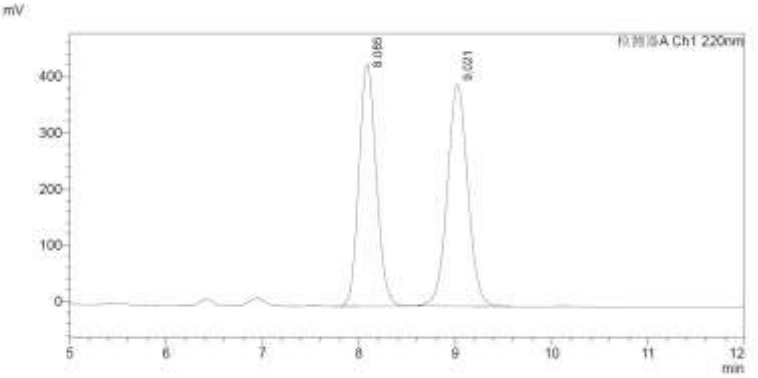

检测器A Ch1 220nm

Peak#	Ret. Time	Area	Height	Conc.	Unit	Mark	Name
1	8.085	5392870	430844	48.163		M	
2	9.021	5804302	393544	51.837		M	
总计		11197172	824388				

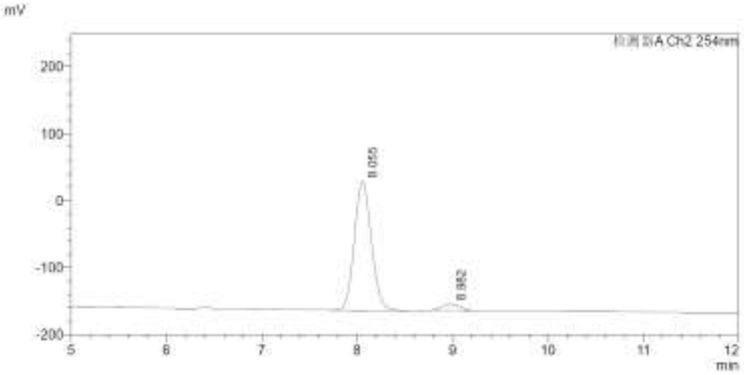

检测器A Ch2 254nm

Peak#	Ret. Time	Area	Height	Conc.	Unit	Mark	Name
1	8.055	2386538	192529	95.233		M	
2	8.982	119450	9403	4.767		M	
总计		2505988	201932				

Supplementary Figure 24. HPLC data of **3q**

(*S*)-1-(4-methoxybenzyl)-3-methyl-3-(5,5,5-trifluoropentyl)-3,4-dihydroquinolin-2(1H)-one (3r)

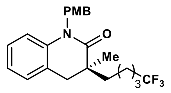

General procedure D was followed on 0.2 mmol and purification flash chromatography on silica gel (PE/EA = 20/1–5/1) to afford **3r** as pale white solid (80.4 mg, 99%); **R*f***= 0.24 (PE/EA = 10/1); $[\alpha]_D^{25}$ = + 15.00 (c = 0.48, $CHCl_3$).

^{1}H NMR (400 MHz, $CDCl_3$): δ 7.14–7.12 (m, 4H), 6.98 (t, *J* = 7.2 Hz, 1H), 6.91 (d, *J* = 8.4 Hz, 1H), 6.84 (d, *J* = 8.4 Hz, 2H), 5.16 (1/2 abq, *J* = 16.0 Hz, 1H), 5.03 (1/2 abq, *J* = 16.0 Hz, 1H), 3.77 (s, 3H), 2.85 (abq, *J* = 15.6 Hz, 2H), 2.09–2.01 (m, 2H), 1.59–1.37 (m, 6H), 1.25 (s, 3H).

^{13}C{^{1}H} NMR (100 MHz, $CDCl_3$): δ 174.5, 158.7, 139.4, 129.5, 128.5, 127.6, 127.4, 127.2 (q, $J_{C\text{–}F}$ = 274.7 Hz), 124.7, 123.0, 115.0, 114.2, 55.3, 46.0, 40.5, 38.1, 36.3, 33.7 (q, $J_{C\text{–}F}$ = 28.3 Hz, 2H), 23.4, 22.5, 22.4 (q, $J_{C\text{–}F}$ = 2.9 Hz).

^{19}F NMR (376 MHz, $CDCl_3$): -66.3.

HRMS (ESI): $[M+H]^+$ Calcd for $C_{23}H_{27}F_3NO_2^+$: 406.1988; found: 406.1979.

HPLC (Chiralpak AD-H): *n*-Hexane/*i*-PrOH = 90/10, flow rate 1.0 mL/min, λ = 220

nm, t_R= 9.062 min (major), t_R= 9.633 min (minor); 92.5:7.5 e.r.

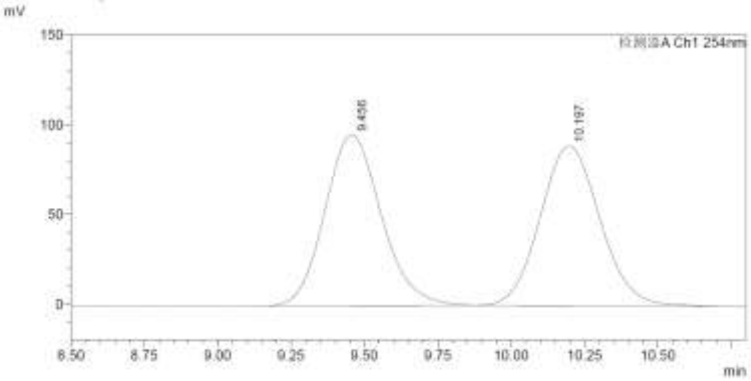

检测器A Ch1 254nm

Peak#	Ret. Time	Area	Height	Conc.	Unit	Mark	Name
1	9.456	1267628	95089	50.234		M	
2	10.197	1255838	88927	49.766		M	
总计		2523466	184016				

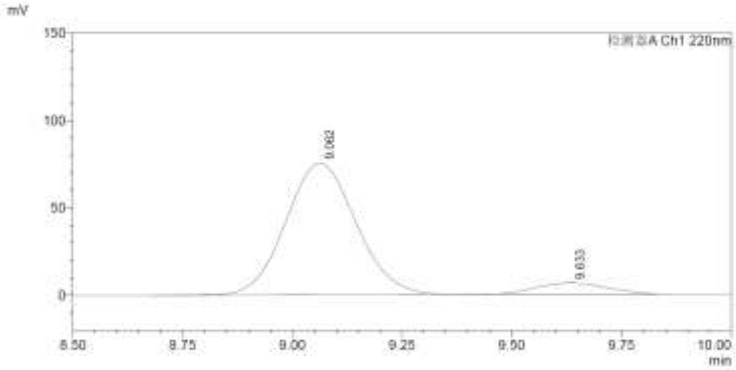

检测器A Ch1 220nm

Peak#	Ret. Time	Area	Height	Conc.	Unit	Mark	Name
1	9.062	839754	75412	92.394		M	
2	9.633	69130	6234	7.606		M	
总计		908884	81646				

Supplementary Figure 25. HPLC data of **3r**

(*S*)-6-(1-(4-methoxybenzyl)-3-methyl-2-oxo-1,2,3,4-tetrahydroquinolin-3-yl)hexanenitrile (3s)

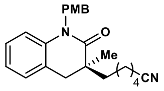

General procedure D was followed on 0.2 mmol and purification flash chromatography on silica gel (PE/EA = 10/1–3/1) to afford **3s** as colorless oil (68.1 mg, 91%); **R*f***= 0.37 (PE/EA = 3/1); $[\alpha]_D^{25}$ = + 26.04 (c = 0.32, $CHCl_3$).

^{1}H NMR (400 MHz, C_6D_6): δ 7.08 (d, *J* = 8.8 Hz, 2H), 6.94–6.90 (m, 2H), 6.83–6.79 (m, 2H), 6.77 (d, *J* = 8.8 Hz, 2H), 5.19 (1/2abq, *J* = 15.6 Hz, 1H), 4.91 (1/2abq, *J* = 15.6 Hz, 1H), 3.31 (s, 3H), 2.48 (abq, *J* = 15.6 Hz, 2H), 1.41–1.28 (m, 4H), 1.17 (s, 3H), 1.12–0.99 (m, 2H), 0.93–0.79 (m, 4H).

^{13}C{^{1}H} NMR (100 MHz, C_6D_6): δ 174.4, 159.8, 140.6, 130.5, 129.0, 128.4, 128.0, 125.5, 123.2, 119.9, 115.6, 115.0, 55.3, 46.5, 41.0, 38.9, 37.1, 29.5, 25.7, 24.0, 23.3, 16.9.

HRMS (ESI): $[M+H]^+$ Calcd for $C_{24}H_{29}N_2O_2^+$: 377.2224; found: 377.2215.

HPLC (Chiralpak AD-H): *n*-Hexane/*i*-PrOH = 80/20, flow rate 1.0 mL/min, λ = 254 nm, t_R= 15.714 min (major), t_R= 18.345 min (minor); 96:4 e.r.

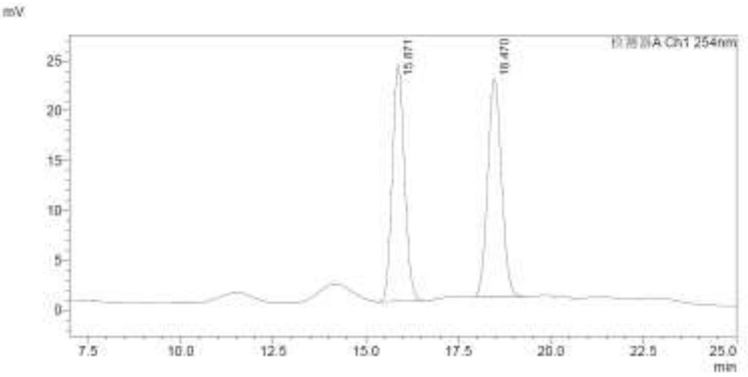

检测器A Ch1 254nm							
Peak#	Ret. Time	Area	Height	Conc.	Unit	Mark	Name
1	15.671	542195	23743	49.144		M	
2	18.470	561090	21853	50.856		M	
总计		1103285	45595				

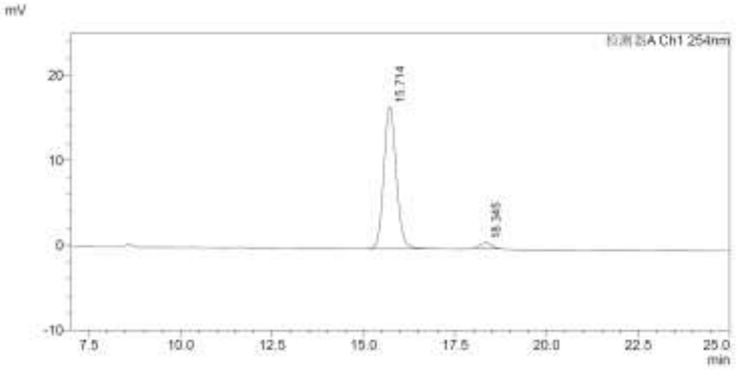

检测器A Ch1 254nm							
Peak#	Ret. Time	Area	Height	Conc.	Unit	Mark	Name
1	15.714	391984	16734	96.153		M	
2	18.345	15684	694	3.847		M	
总计		407668	17428				

Supplementary Figure 26. HPLC data of **3s**

(*S*)-5-(1-(4-methoxybenzyl)-3-methyl-2-oxo-1,2,3,4-tetrahydroquinolin-3-yl)pentyl acetate (3t)

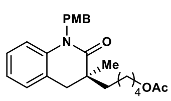

General procedure D was followed on 0.2 mmol and purification flash chromatography on silica gel (PE/EA = 40/1–10/1) to afford **3t** as yellow oil (41.6 mg, 51%); **R*f*** = 0.45 (PE/EA = 20/3); $[\alpha]_D^{25}$ = + 10.91 (c = 0.33, $CHCl_3$)

^{1}H NMR (400 MHz, $CDCl_3$): δ 7.13–7.10 (m, 4H), 6.96 (t, *J* = 7.2 Hz, 1H), 6.88 (d, *J* = 8.0 Hz, 1H), 6.83 (d, *J* = 8.8 Hz, 2H), 5.16 (1/2abq, *J* = 16.0 Hz, 1H), 4.99 (1/2abq, *J* = 16.0 Hz, 1H), 4.02 (t, *J* = 6.8 Hz, 2H), 3.76 (s, 3H), 2.83 (abq, *J* = 15.6 Hz, 2H), 2.03 (s, 3H), 1.63–1.51 (m, 4H), 1.49–1.26 (m, 4H), 1.23 (s, 3H).

^{13}C{^{1}H} NMR (100 MHz, $CDCl_3$): δ 174.9, 171.3, 158.7, 139.5, 129.6, 128.5, 127.6, 127.4, 124.9, 122.9, 115.0, 114.2, 64.6, 55.3, 46.1, 40.6, 38.1, 36.5, 28.6, 26.6, 23.8, 22.6, 21.1.

HRMS (ESI): $[M+H]^+$ Calcd for $C_{25}H_{32}NO_4^+$: 410.2326; found: 410.2316.

HPLC (Chiralpak AD-H): *n*-Hexane/*i*-PrOH = 90/10, flow rate 1.0 mL/min, λ = 220 nm, t_R= 18.071 min (major), t_R= 22.083 min (minor); 96.5:3.5 e.r.

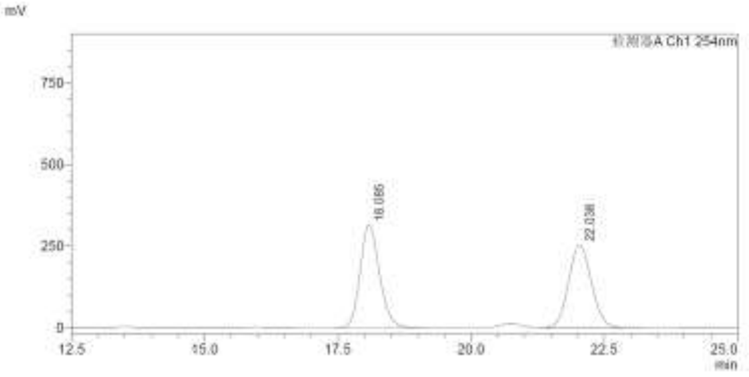

检测器A Ch1 254nm

Peak#	Ret. Time	Area	Height	Conc.	Unit	Mark	Name
1	18.085	7900759	314261	51.177		M	
2	22.038	7537218	253346	48.823		M	
总计		15437977	567607				

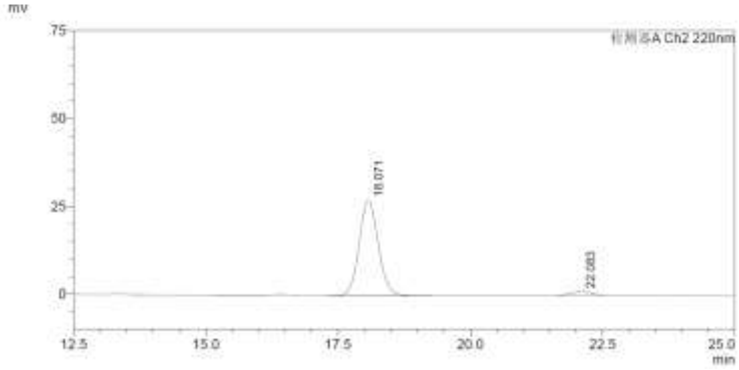

检测器A Ch2 220nm

Peak#	Ret. Time	Area	Height	Conc.	Unit	Mark	Name
1	18.071	675144	27003	96.382		M	
2	22.083	25347	962	3.618		M	
总计		700491	27965				

Supplementary Figure 27. HPLC data of **3t**

(*S*)-3-(4-((*tert*-butyldimethylsilyl)oxy)butyl)-1-(4-methoxybenzyl)-3-methyl-3,4-dihydroquinolin-2(1H)-one (3u)

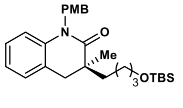

General procedure D was followed on 0.2 mmol and purification flash chromatography on silica gel (PE/EA = 40/1–10/1) to afford **3u** as yellow oil (82.5 mg, 88%); **R*f*** = 0.46 (PE/EA = 10/1); $[\alpha]_D^{25}$ = + 9.79 (c = 0.32, $CHCl_3$).

^{1}H NMR (400 MHz, $CDCl_3$): δ 7.14–7.10 (m, 4H), 6.96 (t, *J* = 7.6 Hz, 1H), 6.88 (d, *J* = 8.0 Hz, 1H), 6.84 (d, *J* = 8.4 Hz, 2H), 5.18 (1/2abq, *J* = 15.6 Hz, 1H), 5.01 (1/2abq, *J* = 16.0 Hz, 1H), 3.76 (s, 3H), 3.59 (t, *J* = 6.4 Hz, 2H), 2.84 (abq, *J* = 15.6 Hz, 2H), 1.63–1.54 (m, 2H), 1.52–1.45 (m, 2H), 1.43–1.31 (m, 2H), 1.25 (s, 3H), 0.90 (s, 9H), 0.04 (s, 6H).

^{13}C{^{1}H} NMR (100 MHz, $CDCl_3$): δ 175.0, 158.7, 139.5, 129.6, 128.4, 127.6, 127.3, 125.0, 122.8, 114.9, 114.2, 63.0, 55.3, 46.0, 40.6, 37.9, 36.3, 33.4, 26.1, 22.5, 20.3, 18.4, -5.18.

HRMS (ESI): $[M+H]^+$ Calcd for $C_{28}H_{42}NO_3Si^+$: 468.2928; found: 468.2917.

HPLC (Chiralpak AD-H): *n*-Hexane/*i*-PrOH = 95/5, flow rate 1.0 mL/min, λ = 254 nm, t_R= 6.171 min (major), t_R= 6.704 min (minor); 96.5:3.5 e.r.

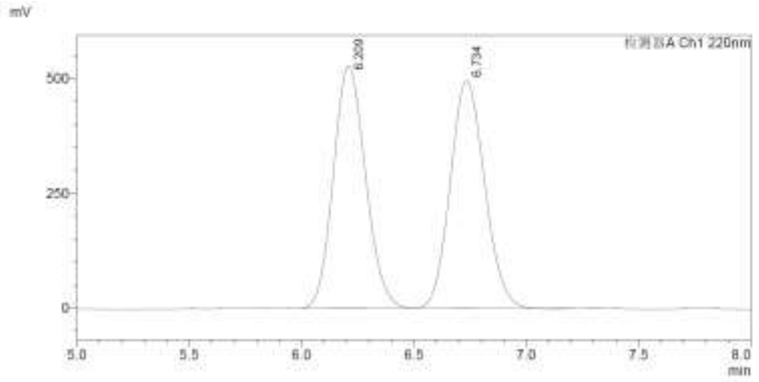

检测器A Ch1 220nm

Peak#	Ret. Time	Area	Height	Conc.	Unit	Mark	Name
1	6.209	5253675	527769	49.967		M	
2	6.734	5260625	495907	50.033		M	
总计		10514299	1023675				

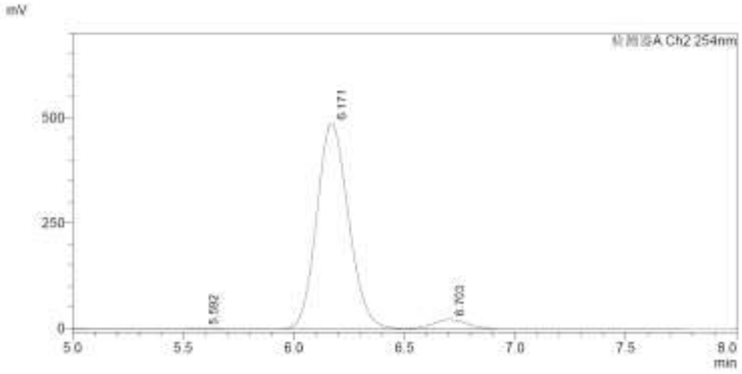

检测器A Ch1 220nm

Peak#	Ret. Time	Area	Height	Conc.	Unit	Mark	Name
1	6.172	6737856	674341	96.676		M	
2	6.704	231656	26206	3.324		M	
总计		6969511	700547				

Supplementary Figure 28. HPLC data of **3u**

Ethyl (*S*)-7-(1-(4-methoxybenzyl)-3-methyl-2-oxo-1,2,3,4-tetrahydroquinolin-3-yl)heptanoate (3v)

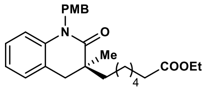

General procedure D was followed on 0.2 mmol and purification flash chromatography on silica gel (PE/EA = 20/1–5/1) to afford **3v** as white solid (82.5 mg, 94%); **R*f*** = 0.59 (PE/EA = 3/1); $[\alpha]_D^{25}$ = + 12.95 (c = 0.32, $CHCl_3$).

^{1}H NMR (400 MHz, $CDCl_3$): δ 7.12–7.08 (m, 4H), 6.94 (t, *J* = 7.6 Hz, 1H), 6.87 (d, *J* = 8.0 Hz, 1H), 6.82 (d, *J* = 8.8 Hz, 2H), 5.17 (1/2 abq, *J* = 15.6 Hz, 1H), 4.98 (1/2 abq, *J* = 16.0 Hz, 1H), 4.10 (q, *J* = 7.2 Hz, 2H), 3.75 (s, 3H), 2.82 (abq, *J* = 15.6 Hz, 2H), 2.25 (t, *J* = 7.2 Hz, 2H), 1.62–1.45 (m, 4H), 1.40–1.21 (m, 12H).

^{13}C{^{1}H} NMR (100 MHz, $CDCl_3$): δ 174.9, 173.8, 158.6, 139.5, 129.6, 128.4, 127.6, 127.3, 124.9, 122.8, 114.9, 114.1, 60.2, 55.3, 46.0, 40.6, 38.0, 36.5, 34.3, 29.8, 29.0, 24.9, 23.8, 22.5, 14.3.

HRMS (ESI): $[M+H]^+$ Calcd for $C_{27}H_{36}NO_4^+$: 438.2639; found: 438.2630.

HPLC (Chiralpak AD-H): *n*-Hexane/*i*-PrOH = 90/10, flow rate 1.0 mL/min, λ = 254 nm, t_R= 15.334 min (major), t_R= 17.714 min (minor); 96.5:3.5 e.r.

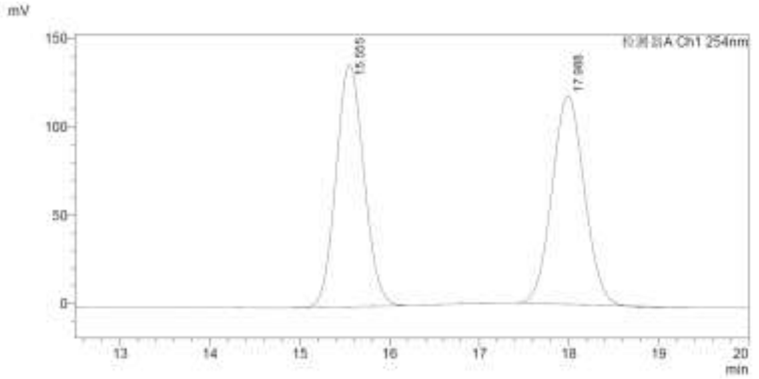

检测器A Ch1 254nm

Peak#	Ret. Time	Area	Height	Conc.	Unit	Mark	Name
1	15.555	2951967	136753	50.010		M	
2	17.988	2950733	117924	49.990		M	
总计		5902699	254677				

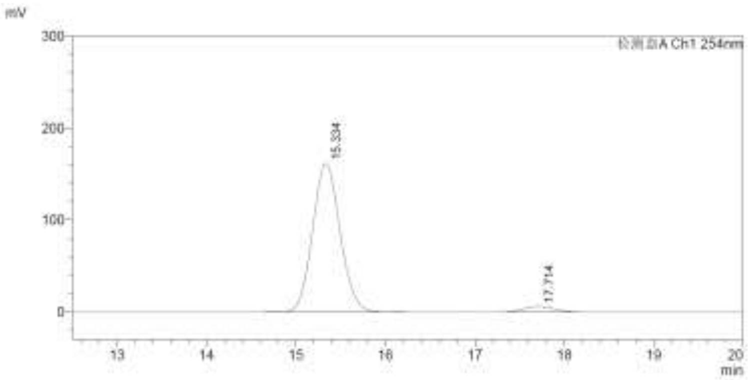

检测器A Ch1 254nm

Peak#	Ret. Time	Area	Height	Conc.	Unit	Mark	Name
1	15.334	3455625	161608	96.443		M	
2	17.714	127461	5756	3.557		M	
总计		3583086	167364				

Supplementary Figure 29. HPLC data of **3v**

(*S*)-1-(4-methoxybenzyl)-3-methyl-3-(4-(p-tolylthio)butyl)-3,4-dihydroquinolin-2(1H)-one (3w)

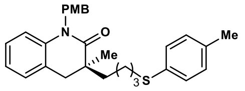

General procedure D was followed on 0.2 mmol and purification flash chromatography on silica gel (PE/EA = 40/1–10/1) to afford **3w** as white solid (88.2 mg, 96%); **R*f***= 0.43 (PE/EA = 10/1); $[\alpha]_D^{25}$ = + 13.51 (c = 0.38, $CHCl_3$).

^{1}H NMR (400 MHz, $CDCl_3$): δ 7.23 (d, *J* = 8.0 Hz, 2H), 7.14–7.08 (m, 6H), 6.97 (t, *J* = 7.2 Hz, 1H), 6.89 (d, *J* = 8.4 Hz, 1H), 6.84 (d, *J* = 8.8 Hz, 2H), 5.17 (1/2abq, *J* = 16.0 Hz, 1H), 5.01 (1/2abq, *J* = 16.0 Hz, 1H), 3.76 (s, 3H), 2.89–2.78 (m, 4H), 2.32 (s, 3H), 1.61–1.37 (m, 6H), 1.24 (s, 3H).

^{13}C{^{1}H} NMR (100 MHz, $CDCl_3$): δ 174.8, 158.7, 139.5, 136.0, 132.9, 130.1, 129.7, 129.5, 128.5, 127.6, 127.4, 124.9, 122.9, 115.0, 114.2, 55.3, 46.1, 40.6, 37.9, 36.1, 34.3, 29.8, 23.3, 22.5, 21.1.

HRMS (ESI): $[M+H]^+$ Calcd for $C_{29}H_{34}NO_2S^+$: 460.2305; found: 460.2293.

HPLC (Chiralpak AD-H): *n*-Hexane/*i*-PrOH = 90/10, flow rate 1.0 mL/min, λ = 254 nm, t_R= 17.796 min (major), t_R= 21.529 min (minor); 96:4 e.r.

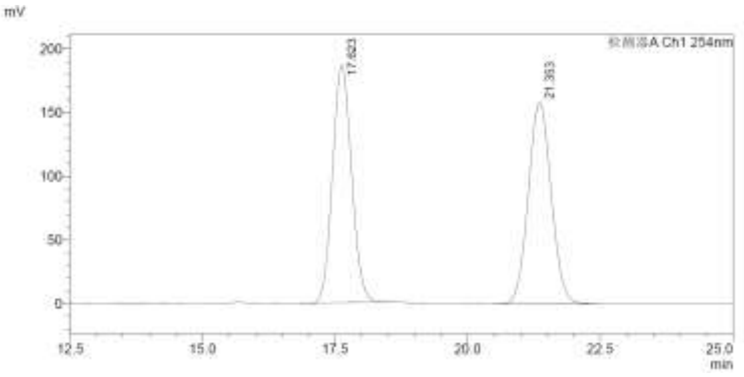

检测器A Ch1 254nm

Peak#	Ret. Time	Area	Height	Conc.	Unit	Mark	Name
1	17.623	4644740	186541	49.475		M	
2	21.353	4743408	158053	50.525		M	
总计		9388148	344593				

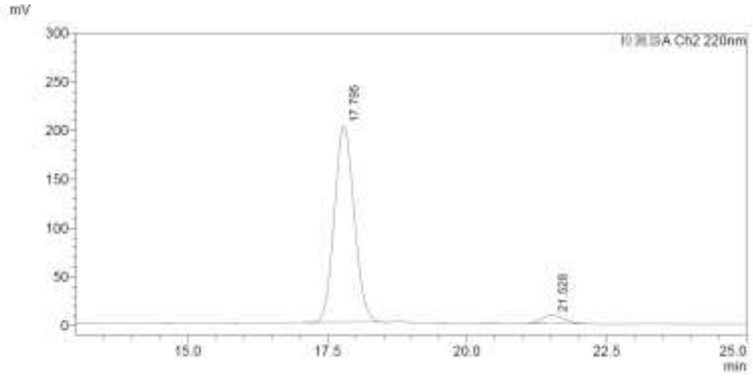

检测器A Ch1 254nm

Peak#	Ret. Time	Area	Height	Conc.	Unit	Mark	Name
1	17.796	3968085	160562	95.961		M	
2	21.529	167001	6378	4.039		M	
总计		4135086	166941				

Supplementary Figure 30. HPLC data of **3w**

(*S*)-4-((9-(1-(4-methoxybenzyl)-3-methyl-2-oxo-1,2,3,4-tetrahydroquinolin-3-yl)nonyl)oxy)benzonitrile (3x)

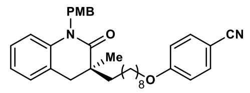

General procedure D was followed on 0.2 mmol and purification flash chromatography on silica gel (PE/EA = 40/1–20/1) to afford **3x** as white solid (85.4 mg, 84%); **R*f***= 0.33 (PE/EA = 10/1); $[\alpha]_D^{25}$ = +13.79 (c = 0.29, $CHCl_3$).

^{1}H NMR (400 MHz, $CDCl_3$): δ 7.56 (d, *J* = 8.8 Hz, 2H), 7.13–7.09 (m, 4H), 6.96 (t, *J* = 7.6 Hz, 1H), 6.92 (d, *J* = 8.8 Hz, 2H), 6.87 (d, *J* = 8.0 Hz, 1H), 6.83 (d, *J* = 8.8 Hz, 2H), 5.19 (1/2abq, *J* = 16.0 Hz, 1H), 4.97 (1/2abq, *J* = 16.0 Hz, 1H), 3.98 (t, *J* = 6.4 Hz, 2H), 3.76 (s, 3H), 2.83 (abq, *J* = 15.6 Hz, 2H), 1.77 (quint, *J* = 6.8 Hz, 2H), 1.57–1.48 (m, 2H), 1.46–1.39 (m, 2H), 1.37–1.24 (m, 13H).

^{13}C{^{1}H} NMR (100 MHz, $CDCl_3$): δ 175.0, 162.5, 158.6, 139.5, 134.0, 129.6, 128.4, 127.6, 127.3, 125.0, 122.8, 119.4, 115.2, 114.9, 114.2, 103.6, 68.5, 55.3, 46.0, 40.6, 38.1, 36.6, 30.2, 29.49, 29.46, 29.3, 29.0, 26.0, 24.0, 22.5.

HRMS (ESI): $[M+H]^+$ Calcd for $C_{34}H_{41}N_2O_3^+$: 525.3112; found: 525.3101.

HPLC (Chiralpak AD-H): *n*-Hexane/EtOH = 90/10, flow rate 1.0 mL/min, 40 ºC, λ = 210 nm, t_R= 28.109 min (major), t_R= 33.229 min (minor); 98:2 e.r.

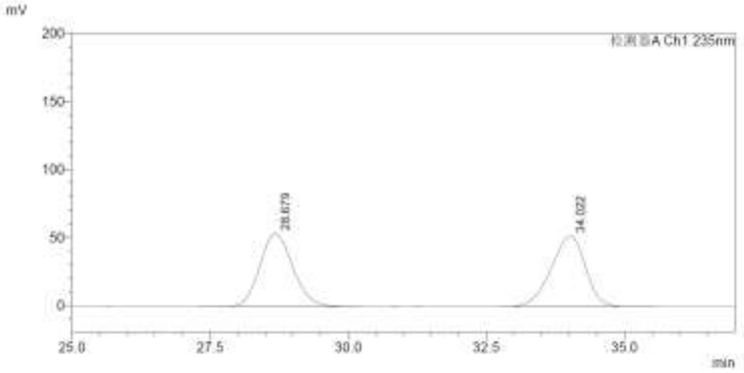

检测器A Ch1 235nm

Peak#	Ret. Time	Area	Height	Conc.	Unit	Mark	Name
1	28.679	2225025	53567	50.014		M	
2	34.022	2223808	51672	49.986		M	
总计		4448833	105238				

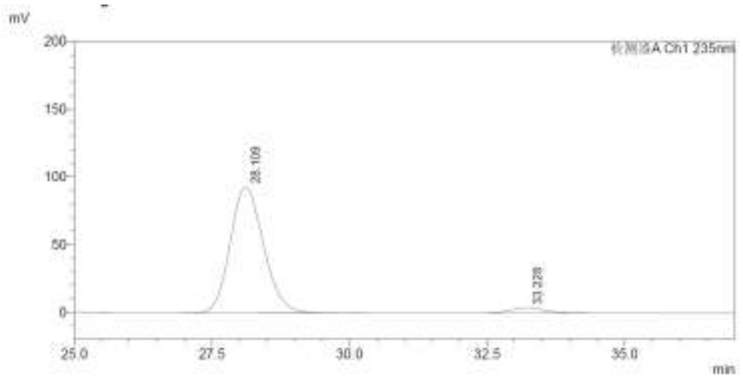

检测器A Ch2 210nm

Peak#	Ret. Time	Area	Height	Conc.	Unit	Mark	Name
1	28.109	7091309	168592	97.760		M	
2	33.229	162510	5173	2.240		M	
总计		7253819	173765				

Supplementary Figure 31. HPLC data of **3x**

(*S*)-1-(4-methoxybenzyl)-3-methyl-3-(4,4,5,5,6,6,7,7,7-nonafluoroheptyl)-3,4-dihydroquinolin-2(1H)-one (3y)

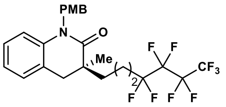

General procedure D was followed on 0.2 mmol and purification flash chromatography on silica gel (PE/EA = 40/1–10/1) to afford **3y** as white solid (63.3 mg, 59%); **R*f*** = 0.35 (PE/EA = 10/1); $[\alpha]_D^{25}$ = + 44.00 (c = 0.05, $CHCl_3$).

^{1}H NMR (400 MHz, $CDCl_3$): δ 7.16–7.12 (m, 4H), 6.99 (t, *J* = 7.6 Hz, 1H), 6.91 (d, *J* = 8.4 Hz, 1H), 6.84 (d, *J* = 8.8 Hz, 2H), 5.09 (abq, *J* = 16.0 Hz, 2H), 3.77 (s, 3H), 2.86 (abq, *J* = 15.6 Hz, 2H), 2.11–1.97 (m, 2H), 1.76–1.62 (m, 4H), 1.26 (s, 3H).

^{13}C{^{1}H} NMR (100 MHz, $CDCl_3$): δ 174.3, 158.8, 139.4, 129.5, 128.6, 127.7, 127.6, 124.6, 123.1, 115.1, 114.3, 55.3 (t, $J_{C\text{–}F}$ = 1.5 Hz), 46.2 (t, $J_{C\text{–}F}$ = 2.5 Hz), 40.7, 38.0, 36.3, 31.3 (t, $J_{C\text{–}F}$ = 23.4 Hz), 22.5, 15.4 (t, $J_{C\text{–}F}$ = 4.1 Hz).

^{19}F NMR (376 MHz, $CDCl_3$): -81.0–-81.1 (m, 3F), -114.4–-114.5 (m, 2F), -124.5–-124.5 (m, 2F), -126.0–-126.1 (m, 2F).

HRMS (ESI): $[M+H]^+$ Calcd for $C_{25}H_{25}F_9NO_2^+$: 542.1736; found: 542.1724.

HPLC (Chiralpak AD-H): *n*-Hexane/*i*-PrOH = 95/5, flow rate 1.0 mL/min, λ = 254 nm,

t_R= 9.800 min (minor), t_R= 10.315 min (major); 95.5:4.5 e.r.

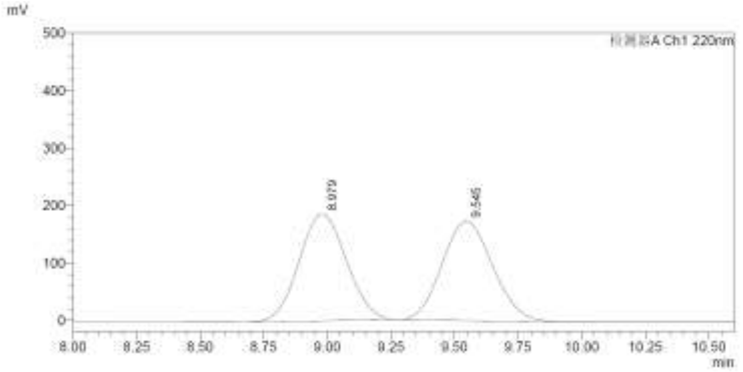

检测器A Ch1 220nm

Peak#	Ret. Time	Area	Height	Conc.	Unit	Mark	Name
1	8.979	2410589	185746	50.140		M	
2	9.545	2397153	172910	49.860		M	
总计		4807741	358656				

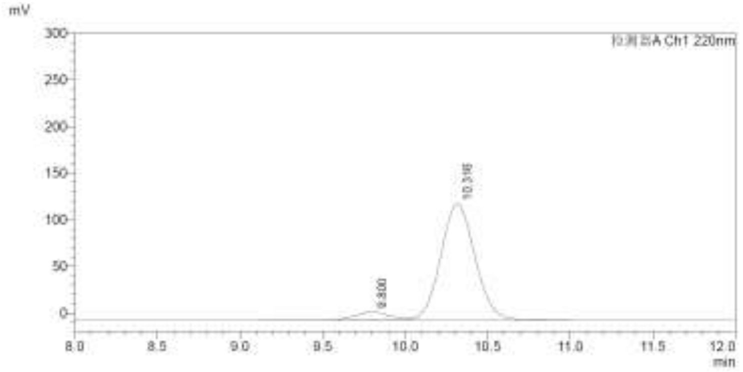

检测器A Ch2 254nm

Peak#	Ret. Time	Area	Height	Conc.	Unit	Mark	Name
1	9.800	61048	5194	4.630		M	
2	10.315	1257435	88956	95.370		M	
总计		1318483	94150				

Supplementary Figure 32. HPLC data of **3y**

(*S*)-3-((*R*)-4,8-dimethylnon-7-en-1-yl)-1-(4-methoxybenzyl)-3-methyl-3,4-dihydroquinolin-2(1H)-one (3z)

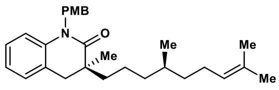

General procedure D was followed on 0.2 mmol and purification flash chromatography on silica gel (PE/EA = 40/1–10/1) to afford **3z** as colorless oil (61.7 mg, 71%); **R*f*** = 0.42 (PE/EA = 10/1); $[\alpha]_D^{25}$ = +8.43 (c = 0.34, $CHCl_3$).

^{1}H NMR (400 MHz, $CDCl_3$): δ 7.14 – 7.10 (m, 4H), 6.96 (t, *J* = 7.6 Hz, 1H), 6.88 (d, *J* = 8.0 Hz, 1H), 6.84 (d, *J* = 8.4 Hz, 2H), 5.24 (1/2 abq, *J* = 16.0 Hz, 1H), 5.08 (t, *J* = 7.2 Hz, 1H), 4.94 (1/2 abq, *J* = 16.0 Hz, 1H), 3.77 (s, 3H), 2.85 (s, 2H), 2.02–1.84 (m, 2H), 1.68 (s, 3H), 1.59 (s, 3H), 1.56–1.43 (m, 2H), 1.43–1.20 (m, 8H), 1.15–1.00 (m, 2H), 0.84 (d, *J* = 6.8 Hz, 3H).

^{13}C{^{1}H} NMR (100 MHz, $CDCl_3$): δ 175.0, 158.6, 139.6, 131.1, 129.6, 128.4, 127.6, 127.3, 125.1, 122.8, 114.9, 114.2, 55.3, 46.1, 40.7, 38.3, 37.6, 37.3, 36.8, 32.4, 25.8, 25.6, 22.6, 21.5, 19.6, 17.7.

HRMS (ESI): $[M+H]^+$ Calcd for $C_{29}H_{40}NO_2^+$: 434.3054; found: 434.3044.

HPLC (Chiralpak AD-H): *n*-Hexane/EtOH = 98/2, flow rate 1.0 mL/min, λ = 240 nm,

t_R= 7.200 min (major), t_R= 8.616 min (minor); 97:3 d.r.

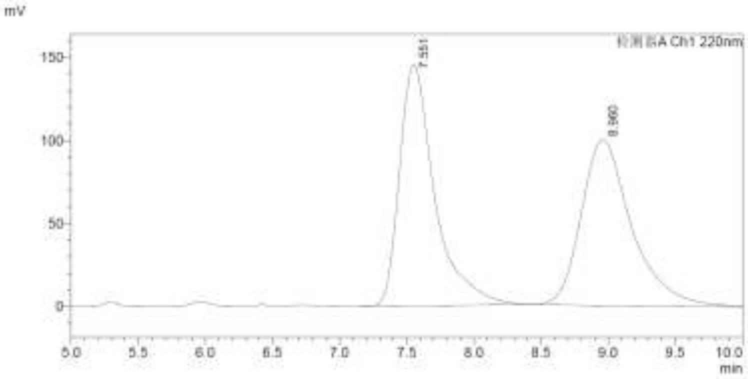

检测器A Ch1 220nm

Peak#	Ret. Time	Area	Height	Conc.	Unit	Mark	Name
1	7.551	2644977	145028	50.119		M	
2	8.960	2632372	99673	49.881		M	
总计		5277349	244700				

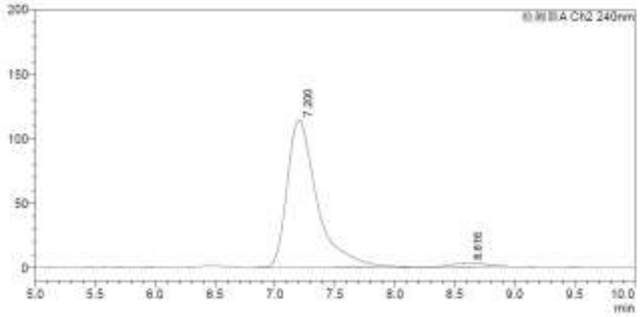

检测器A Ch2 240nm

Peak#	Ret. Time	Area	Height	Conc.	Unit	Mark	Name
1	7.200	1996350	114546	97.171		M	
2	8.616	58131	2989	2.829		M	
总计		2054482	117535				

Supplementary Figure 33. HPLC data of **3z**

(*S*)-1-benzyl-3-octylpiperidin-2-one (5a)

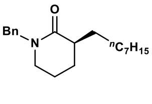

General procedure E was followed on 0.2 mmol and purification flash chromatography on silica gel (PE/EA = 30/1–10/1) to afford **5a** as colorless oil (48.5 mg, 82%); **R*f***= 0.55 (PE/EA = 5/1); $[\alpha]_D^{25}$ = +21.67 (c = 0.12, $CHCl_3$).

^{1}H NMR (400 MHz, $CDCl_3$): δ 7.33–7.29 (m, 2H), 7.27–7.22 (m, 3H), 4.58 (abq, *J* = 14.8 Hz, 2H), 3.18 (dd, *J* = 7.2, 5.2 Hz, 2H), 2.39–2.32 (m, 1H), 2.00–1.90 (m, 2H), 1.87–1.79 (m, 1H), 1.73–1.63 (m, 1H), 1.58–1.46 (m, 2H), 1.33–1.25 (m, 12H), 0.88 (t, *J* = 6.4 Hz, 3H).

^{13}C{^{1}H} NMR (100 MHz, $CDCl_3$): δ 173.1, 137.7, 128.7, 128.1, 127.3, 50.4, 47.5, 41.8, 32.2, 32.0, 29.8, 29.7, 29.4, 27.3, 26.5, 22.8, 21.7, 14.3.

HRMS (ESI): $[M+H]^+$ Calcd for $C_{20}H_{32}NO^+$: 302.2478; found: 302.2471.

HPLC (Chiralpak AD-H): *n*-Hexane/*i*-PrOH = 90/10, flow rate 1.0 mL/min, λ = 220 nm, t_R= 5.524 min (major), t_R= 6.281 min (minor); 93.5:6.5 e.r.

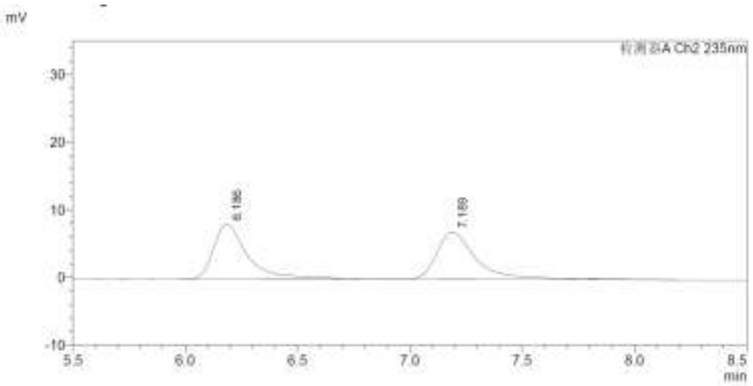

检测器A Ch2 235nm

Peak#	Ret. Time	Area	Height	Conc.	Unit	Mark	Name
1	6.186	87257	8136	50.505		M	
2	7.189	85511	6986	49.495		M	
总计		172767	15122				

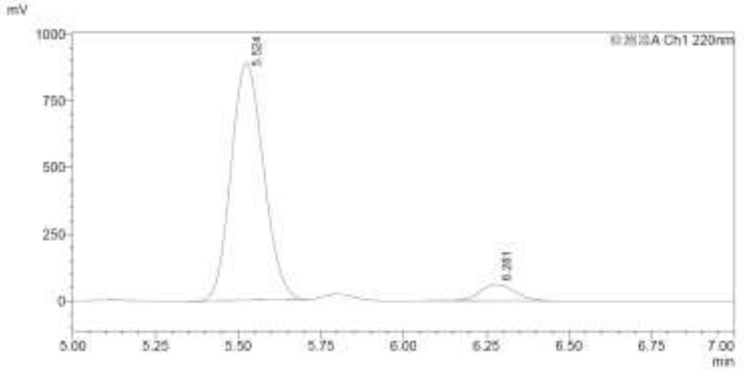

检测器A Ch1 220nm

Peak#	Ret. Time	Area	Height	Conc.	Unit	Mark	Name
1	5.524	6085453	891137	93.325		M	
2	6.281	435234	60991	6.675		M	
总计		6520687	952128				

Supplementary Figure 34. HPLC data of **5a**

5-(1-benzyl-2-oxopiperidin-3-yl)pentyl acetate (5b)

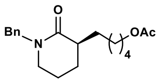

General procedure E was followed on 0.1 mmol and purification flash chromatography on silica gel (PE/EA = 20/1–3/1) to afford **5b** as colorless oil (25.5mg, 80%); **R***f*= 0.29 (PE/EA = 3/1); $[\alpha]_D^{25}$ = +22.22 (c = 0.09, $CHCl_3$).

^{1}H NMR (400 MHz, $CDCl_3$): δ 7.24 (m, 2H), 7.20–7.15 (m, 3H), 4.51 (abq, *J* = 14.4 Hz, 2H), 3.99 (t, *J* = 6.8 Hz, 2H), 3.12 (dd, *J* = 7.2, 5.2 Hz, 2H), 2.32–2.25 (m, 1H), 1.87 (s, 3H), 1.95–1.83 (m, 2H), 1.81–1.73 (m, 1H), 1.67–1.55 (m, 3H), 1.51–1.42 (m, 2H), 1.39–1.26 (m, 4H).

^{13}C{^{1}H} NMR (100 MHz, $CDCl_3$): δ 172.8, 171.3, 137.6, 128.6, 128.1, 127.3, 64.6, 50.4, 47.5, 41.6, 32.0, 28.6, 26.8, 26.5, 26.1, 21.7, 21.1.

HRMS (ESI): $[M+H]^+$ Calcd for $C_{19}H_{28}NO_3^+$: 318.2064; found: 318.2057.

HPLC (Chiralpak AD-H): *n*-Hexane/ *i*-PrOH = 80/20, flow rate 1.0 mL/min, λ = 254 nm, t_R= 6.867 min (major), t_R= 7.914 min (minor); 92.5:7.5 e.r

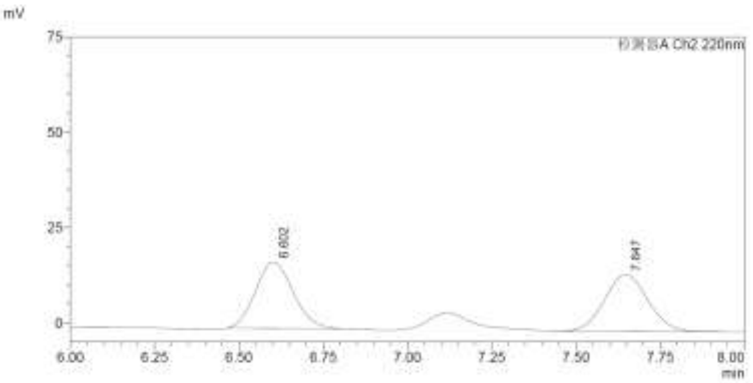

检测器A Ch2 220nm

Peak#	Ret. Time	Area	Height	Conc.	Unit	Mark	Name
1	6.602	134581	17405	50.334		M	
2	7.647	132797	14869	49.666		M	
总计		267378	32274				

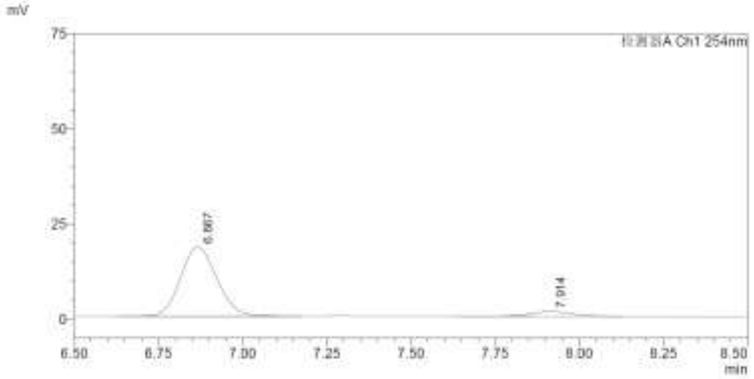

检测器A Ch1 254nm

Peak#	Ret. Time	Area	Height	Conc.	Unit	Mark	Name
1	6.867	136342	18207	92.741		M	
2	7.914	10672	1259	7.259		M	
总计		147014	19466				

Supplementary Figure 35. HPLC data of **5b**

1-butyl-3-octylpiperidin-2-one (5c)

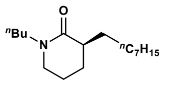

General procedure E was followed on 0.1 mmol and purification flash chromatography on silica gel (PE/EA = 20/1–5/1) to afford **5c** as colorless oil (20.1 mg, 75%); $\mathbf{R}_f$= 0.34 (PE/EA = 5/1); $[\alpha]_D^{25}$ = +25.88 (c = 0.17, $CHCl_3$).

^{1}H NMR (400 MHz, $CDCl_3$): δ 3.39–3.23 (m, 4H), 2.27–2.20 (m, 1H), 1.92–1.81 (m, 3H), 1.76–1.65 (m, 1H), 1.53–1.38 (m, 4H), 1.34–1.25 (m, 14H), 0.91 (t, J = 7.6 Hz, 3H), 0.86 (t, J = 6.8 Hz, 3H).

^{13}C{^{1}H} NMR (100 MHz, $CDCl_3$): δ 172.6, 48.1, 47.2, 41.6, 32.2, 32.0, 29.8, 29.7, 29.43, 29.37, 27.2, 26.6, 22.8, 22.0, 20.3, 14.2, 14.0.

HRMS (ESI): $[M+H]^+$ Calcd for $C_{17}H_{34}NO^+$: 268.2635; found: 268.2628.

HPLC (Chiralpak AD-H): *n*-Hexane/*i*-PrOH = 95/5, flow rate 1.0 mL/min, λ = 220 nm, t_R= 5.290 min (major), t_R= 5.606 min (minor); 93:7 e.r

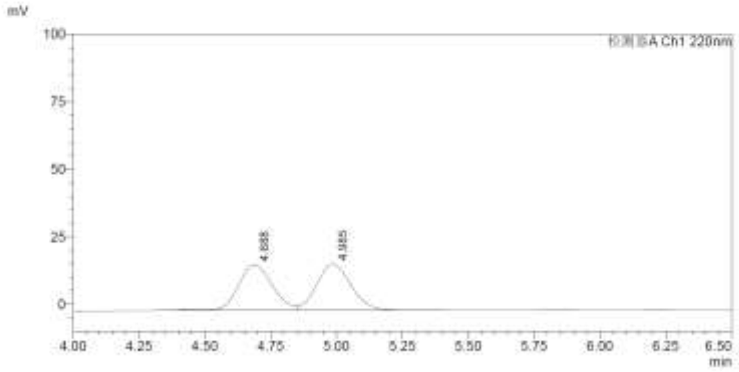

检测器A Ch1 220nm

Peak#	Ret. Time	Area	Height	Conc.	Unit	Mark	Name
1	4.688	130625	15894	51.259		M	
2	4.985	124210	15380	48.741		M	
总计		254835	31274				

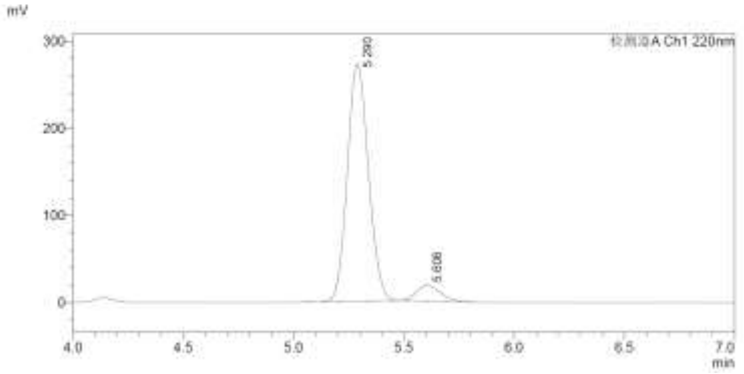

检测器A Ch1 220nm

Peak#	Ret. Time	Area	Height	Conc.	Unit	Mark	Name
1	5.290	1811678	272370	93.074		M	
2	5.606	134812	17811	6.926		M	
总计		1946491	290180				

Supplementary Figure 36. HPLC data of **5c**

1.7 Experimental Procedures and Characterization Data for Derivatization Experiment

a) Procedure for oxidation of compound 3k

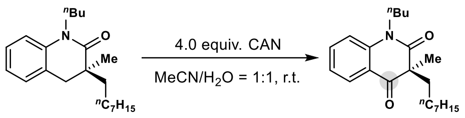

3k, 92%, **95% ee** **6**, 73%, **95% ee**

Supplementary Figure 37. Oxidation of **3k**

To a round-bottomed vial charged with **3k** (1.0 equiv, 0.1 mmol, 32.9 mg) in MeCN/H_2O = 1:1 (0.1 mL, 1.0 M) was added CAN (4.0 equiv, 0.4 mmol, 212.1 mg). The mixture was stirred at room temperature for 3 h. Until consumption of starting material was observed by TLC, the reaction mixture was filtered through a pad of Celite and the filtrate was washed with water and brine. The combined organic layer was dried over anhydrous Na_2SO_4, filtered, and concentrated under reduced pressure. The crude product was purified by flash chromatography to afford **6** as a colorless oil (23.1 mg, 73%, 95% ee). **R*f***= 0.56 (PE/EA = 10/1); $[\alpha]_D^{25}$ = +15.56 (c = 0.15, $CHCl_3$).

^{1}H NMR (400 MHz, $CDCl_3$): δ 8.00 (dd, *J* = 8.0, 1.6 Hz, 1H), 7.60 (dt, *J* = 7.2, 1.6, 1H), 7.16–7.11 (m, 2H), 4.14–4.04 (m, 1H), 3.99–3.92 (m, 1H), 1.97–1.84 (m, 2H), 1.65 (quint, *J* = 7.2 Hz, 2H), 1.48–1.39 (m, 5H), 1.27–1.09 (m, 12H), 0.98 (t, *J* = 7.2 Hz, 3H), 0.83 (t, *J* = 6.8 Hz, 3H).

^{13}C{^{1}H} NMR (100 MHz, $CDCl_3$): δ 197.9, 173.8, 142.6, 136.0, 128.4, 122.9, 120.6, 114.9, 57.3, 42.3, 39.7, 31.9, 29.9, 29.32, 29.25, 29.2, 25.1, 22.72, 22.66, 20.3, 14.2, 13.9.

HRMS (ESI): $[M+H]^+$ Calcd for $C_{22}H_{34}NO_2^+$: 344.2584; found: 344.2575.

HPLC (Chiralpak IC): *n*-Hexane/*i*-PrOH = 95/5, flow rate 1.0 mL/min, λ = 208 nm, t_R= 6.064 min (major), t_R= 6.765 min (minor); 97.5:2.5 e.r.

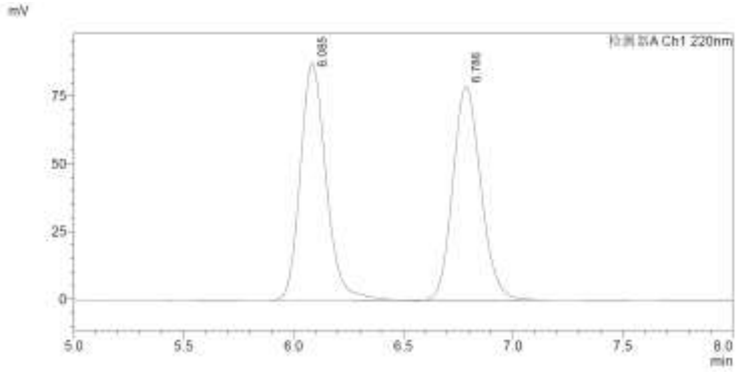

检测器A Ch1 220nm

Peak#	Ret. Time	Area	Height	Conc.	Unit	Mark	Name
1	6.085	710989	87545	50.457		M	
2	6.786	698103	78955	49.543		M	
总计		1409092	166499				

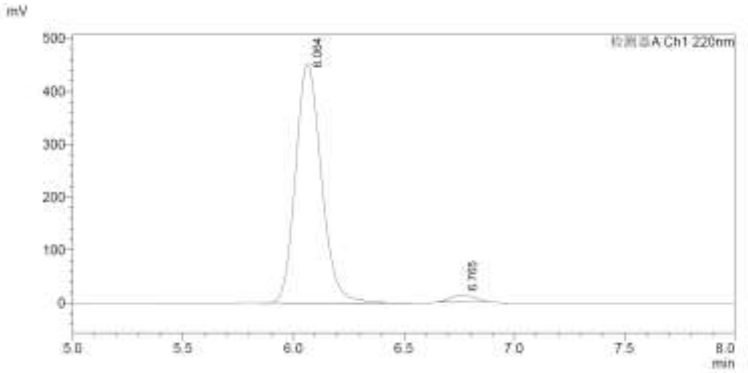

检测器A Ch1 220nm

Peak#	Ret. Time	Area	Height	Conc.	Unit	Mark	Name
1	6.064	3682494	451486	97.462		M	
2	6.765	95907	13195	2.538		M	
总计		3778401	464681				

Supplementary Figure 38. HPLC data of **6**

b) Procedure for deprotection of compound 3a

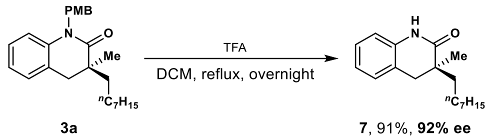

Supplementary Figure 39. Deprotection of **3a**

To a vial charged with **3a** (1.0 equiv, 0.1 mmol, 39.3 mg) in DCM (1.0 mL, 0.1 M) was added TFA (1.0 mL). The reaction mixture was refluxed for overnight until consumption of starting material was observed by TLC. The reaction mixture was concentrated under reduced pressure directly and purified by flash chromatography on silica gel to afford **7** as a yellow oil (24.8 mg, 91%, 92% ee). **R*f*** = 0.42 (PE/EA = 1/1); $[\alpha]_D^{25}$ = +25.93 (c = 0.09, $CHCl_3$).

^{1}H NMR (400 MHz, $CDCl_3$): δ 7.68 (s, 1H), 7.18–7.12 (m, 2H), 6.97 (dt, *J* = 7.4, 0.8 Hz, 1H), 6.70 (d, *J* = 8.0 Hz, 1H), 2.85 (1/2abq, *J* = 15.6 Hz, 1H), 2.76 (1/2abq, *J* = 16.0 Hz, 1H), 1.52–1.47 (m, 2H), 1.37–1.23 (m, 12H), 1.19 (s, 3H), 0.86 (t, *J* = 6.8 Hz, 3H).

^{13}C{^{1}H} NMR (100 MHz, $CDCl_3$): δ 176.1, 136.9, 128.6, 127.4, 123.4, 123.0, 114.4, 40.7, 38.0, 36.6, 31.9, 30.2, 29.5, 29.3, 24.0, 22.7, 22.3, 14.2.

HRMS (ESI): $[M+H]^+$ Calcd for $C_{18}H_{28}NO^+$: 274.2165; found: 274.2160.

HPLC (Chiralpak AD-H): *n*-Hexane/*i*-PrOH = 90/10, flow rate 1.0 mL/min, λ = 208 nm, t_R= 6.644 min (major), t_R= 6.993 min (minor); 96:4 e.r.

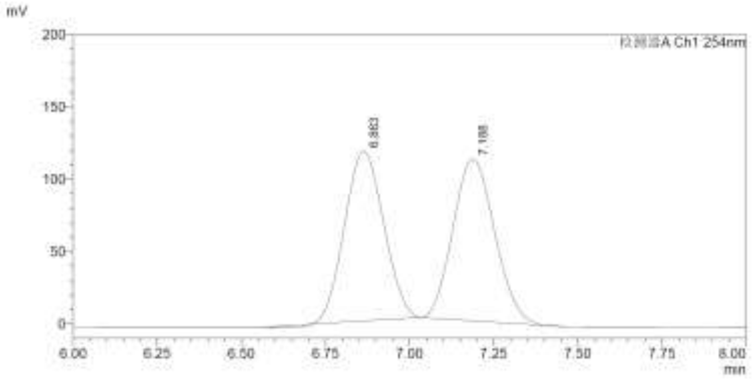

检测器A Ch1 254nm

Peak#	Ret. Time	Area	Height	Conc.	Unit	Mark	Name
1	6.863	947171	117288	49.557		M	
2	7.188	964112	111945	50.443		M	
总计		1911283	229233				

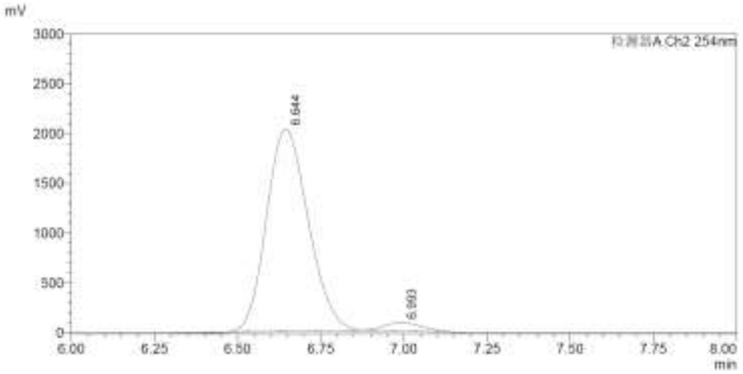

检测器A Ch2 254nm

Peak#	Ret. Time	Area	Height	Conc.	Unit	Mark	Name
1	6.644	17668780	2031161	96.480		M	
2	6.993	644660	82827	3.520		M	
总计		18313440	2113988				

Supplementary Figure 40. HPLC data of **7**

c) Procedure for reduction of Compound 3a

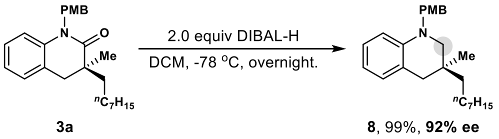

Supplementary Figure 41. Reduction of **3a**

To an oven-dried vial charged with **3a** (1.0 equiv, 0.1 mmol, 39.3 mg) in DCM (1.0 mL, 0.1 M) was added DIBAL-H (1.0 M in toluene, 2.0 equiv, 0.2 mmol) under nitrogen atmosphere at -78 °C. The reaction mixture was stirred for overnight at the same temperature until consumption of starting material was observed by TLC. The reaction mixture was quenched with 1.0 M NaOH solution carefully. The resulting solution was stirred at -78 °C for 30 min and then filtered through a pad of Celite. The filtrate was extracted three times with ethyl acetate and the combined organic layer was washed with brine, dried over anhydrous Na_2SO_4, filtered, and concentrated under reduced pressure. The residue was purified by flash chromatography on silica gel to afford **8** as a colorless oil (37.8 mg, 99%, 92% ee). $\mathbf{R_f}$= 0.71 (PE/EA = 20/1); $[\alpha]_D^{25}$ =

+14.76 (c = 0.14, $CHCl_3$).

^{1}H NMR (400 MHz, $CDCl_3$): δ 7.21 (d, *J* = 8.4 Hz, 2H), 7.03–6.98 (m, 2H), 6.87 (d, *J* = 8.4 Hz, 2H), 6.61 (t, *J* = 7.6 Hz, 1H), 6.57 (d, *J* = 8.0 Hz, 1H), 4.44 (s, 2H), 3.81 (s, 3H), 3.07 (1/2abq *J* = 11.2 Hz, 1H), 2.98 (1/2abq, *J* = 11.2 Hz, 1H), 2.59 (abq, *J* = 16.0 Hz, 2H), 1.32–1.28 (m, 14H), 0.99 (s, 3H), 0.91 (t, *J* = 6.4 Hz, 3H).

^{13}C{^{1}H} NMR (100 MHz, $CDCl_3$): δ 158.6, 144.9, 131.3, 129.8, 127.9, 127.1, 121.2, 116.0, 114.0, 110.7, 60.0, 55.4, 54.9, 41.1, 39.5, 32.1, 31.2, 30.6, 29.8, 29.5, 23.7, 23.6, 22.8, 14.3.

HRMS (ESI): $[M+H]^+$ Calcd for $C_{26}H_{38}NO^+$: 380.2948; found: 380.2938.

HPLC (Chiralpak OD-H): *n*-Hexane/*i*-PrOH = 99/1, flow rate 0.5 mL/min, λ = 220 nm, t_R= 13.959 min (major), t_R= 17.502 min (minor); 96:4 e.r.

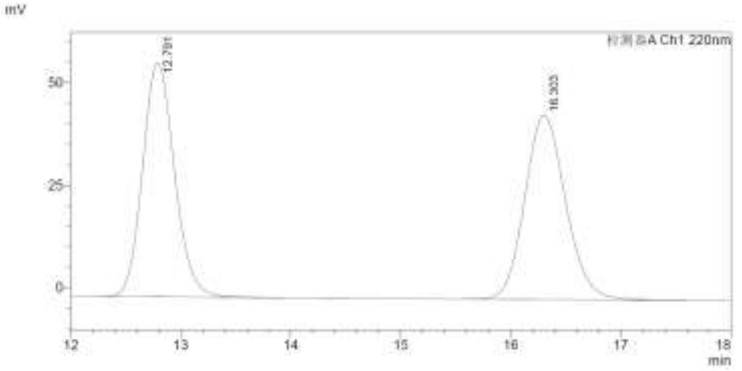

检测器A Ch1 220nm

Peak#	Ret. Time	Area	Height	Conc.	Unit	Mark	Name
1	12.791	1156066	57201	49.897			
2	16.303	1160832	44911	50.103			
总计		2316898	102112				

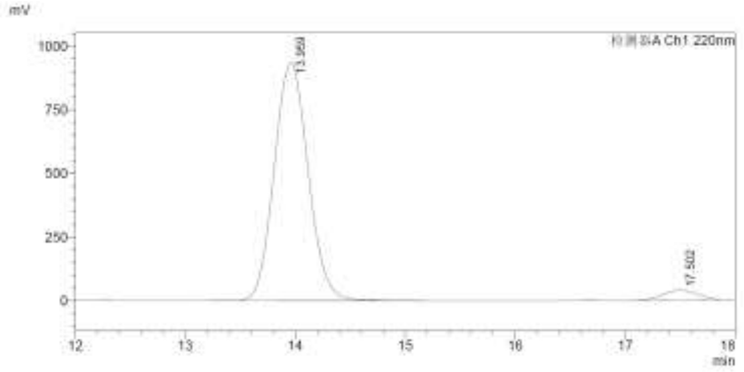

检测器A Ch1 220nm

Peak#	Ret. Time	Area	Height	Conc.	Unit	Mark	Name
1	13.959	20057447	934114	95.898		M	
2	17.502	857911	38653	4.102		M	
总计		20915358	972767				

Supplementary Figure 42. HPLC data of **8**

d) Procedure for reduction/cyanation of compound 3a

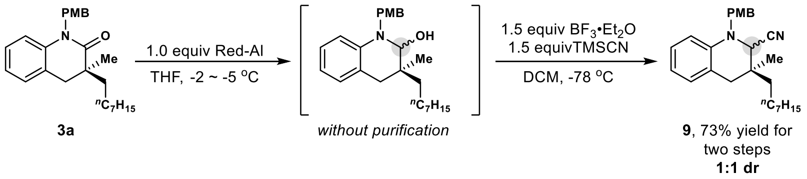

Supplementary Figure 43. Reduction/cyanation of **3a**

To a solution of **3a** (1.0 equiv, 0.1 mmol, 39.3 mg) in THF (1.0 mL, 0.1 M) was added Red-Al (1.0 equiv, 0.1 mmol) dropwise under N_2 atmosphere at -2 ^{o}C ~ -5 ^{o}C. The reaction mixture was stirred for overnight at the same temperature until consumption of starting material was observed by TLC. The reaction mixture was quenched with H_2O carefully, the resulting solution was extracted three times with ethyl acetate. The combined organic layer was washed with brine, dried over anhydrous Na_2SO_4, filtered, and concentrated under reduced pressure affording crude product as a yellow oil, which was used directly without further purification.

The crude product described above and DCM (1.0 mL) were added to an oven-dried vial under N_2 atmosphere. The mixture was cooled to -78 ^{o}C. Then, TMSCN (1.5 equiv, 0.15 mmol, 19 μL) was added followed by the addition of BF_3 • Et_2O (1.5 equiv, 0.15 mmol, 19 μL). The resulting solution was stirred until complete conversion of starting material. The residue was concentrated under reduced pressure directly and purified by flash chromatography on silica gel to afford **9** as a yellow oil (29.5 mg, 73%, 1:1 dr).
R*f* = 0.77 (PE/EA = 5/1);
^{1}H NMR (400 MHz, $CDCl_3$): δ 7.27–7.23 (m, 2H), 7.10 (t, *J* = 7.6 Hz, 1H), 7.04 (t, *J* = 6.4 Hz, 1H), 6.92–6.87 (m, 2H), 6.80–6.75 (m, 2H), 4.77 (1/2abq+1/2abq, *J* = 15.6 Hz, 1H), 4.22 (1/2abq + 1/2abq, *J* = 15.6 Hz, 1H), 3.82 (s+s, 3H), 3.80 (d, *J* = 2.0 Hz, 0.5H), 3.74 (d, *J* = 2.4 Hz, 0.5H), 3.00 (1/2abq + 1/2abq, *J* = 16.8 Hz, 1H), 2.53 (1/2abqd +1/2abqd, *J* = 16.4, 1.6 Hz, 1H), 1.49–1.17 (m, 15.5H), 1.02 (s, 1.5H), 0.91–0.86 (m, 3H).
^{13}C{^{1}H} NMR (100 MHz, $CDCl_3$): δ 159.3, 159.2, 142.13, 142.08, 130.2, 130.0, 129.1, 129.0, 128.9, 128.7, 127.51, 127.49, 121.1, 120.9, 119.2, 119.1, 118.3, 118.0, 114.4, 114.3, 112.6, 59.2, 58.1, 55.42, 55.39, 53.8, 53.5, 40.4, 38.2, 37.9, 36.4, 34.3, 34.0, 31.99, 31.97, 30.3, 30.2, 29.7, 29.6, 29.39, 29.37, 24.0, 23.6, 23.1, 22.78, 22.76, 21.3, 14.2.
HRMS (ESI): $[M+H]^+$ Calcd for $C_{27}H_{37}N_2O^+$: 405.2900; found: 405.2891.

1.8 Mechanistic Experiments

a) Radical ring-opening experiment

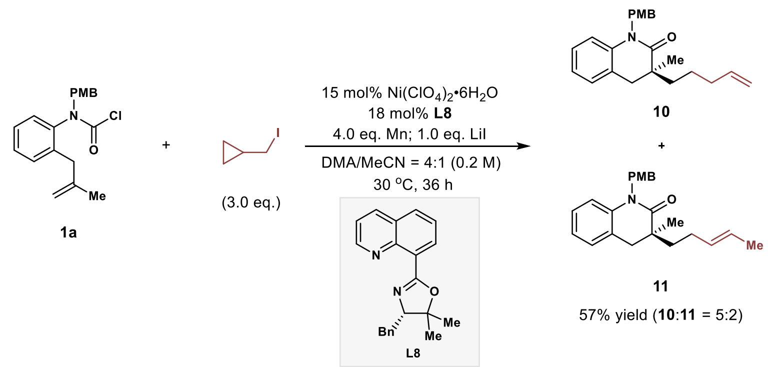

Supplementary Figure 44. Radical ring-opening experiment of **1a**

To a dried 8-mL vial were added $Ni(ClO_4)_2$ • $6H_2O$ (15 mol%, 0.015 mmol, 5.5 mg), **L8** (18 mol%, 0.018 mmol, 5.7 mg), Mn (4.0 equiv, 0.4 mmol, 22.0 mg) and carbamoyl chloride **1a** (1.0 equiv, 0.1 mmol, 32.9 mg). Then the vial was transferred into glovebox. LiI (1.0 equiv, 0.1 mmol, 13.4 mg), DMA (0.4 mL), MeCN (0.1 mL) and (iodomethyl)cyclopropane (3.0 equiv, 0.3 mmol, 28 μL) were added in sequence inside the glovebox. The vial was then taken out from the glovebox, sealed with parafilm, put into oil bath (30 °C) and stirred for 36 h. After completion, the reaction mixture was quenched with H_2O, filtered through a pad of Celite and extracted with EA for three times. The combined organic phase was washed with brine and concentrated under reduced pressure to yield the crude product, which was purified by silica gel flash column chromatography to afford ring-openning products **10** and **11** as an inseperable mixture (19.9 mg, 57%, **10**:**11** = 5:2). The product **11** was transformation from product **10** by olefin isomerization under standard condition. $^{13}C\{^1H\}$ data listed is for the major product **10**.

R*f* = 0.52 (PE/EA = 10/1);

^{1}H NMR (400 MHz, $CDCl_3$): δ 7.13–7.10 (m, 5.6H), 6.96 (t, *J* = 7.2 Hz, 1.4H), 6.87 (d, *J* = 8.0 Hz, 1.4H), 6.83 (d, *J* = 8.8 Hz, 2.8H), 5.76 (ddt, *J* = 16.8, 10.0, 6.4 Hz, 1H), 5.44–5.29 (m, 0.8H), 5.25–5.14 (m, 1.4H), 5.02–4.91 (m, 3.4H), 3.77 (s, 4.2H), 2.89–2.78 (m, 2.8H), 2.05–1.98 (m, 2.8H), 1.61 (d, *J* = 4.8 Hz, 1.2H), 1.58–1.33 (m, 4.8H), 1.26 (s, 1.2H), 1.24 (s, 3H).

$^{13}C\{^1H\}$ NMR (100 MHz, $CDCl_3$): δ175.0, 158.7, 139.6, 138.6, 129.6, 128.5, 127.6, 127.4, 125.0, 122.9, 115.0, 114.8, 114.2, 55.4, 46.1, 40.6, 38.0, 36.1, 34.2, 23.5, 22.6.

b) Radical trap reaction

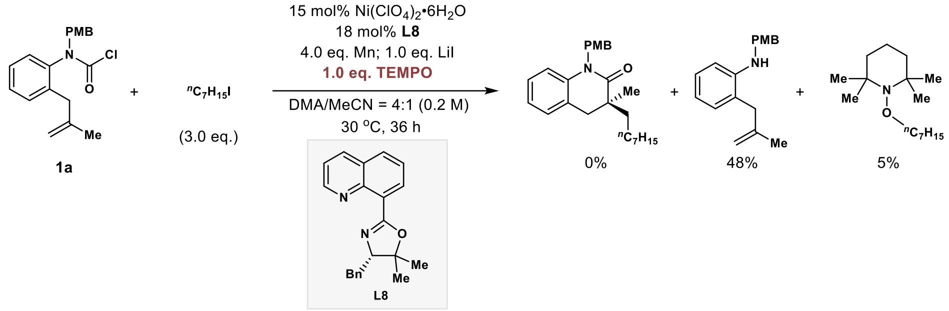

Supplementary Figure 45. Radical trap reaction of **1a**

To a dried 8-mL vial were added $Ni(ClO_4)_2 \cdot 6H_2O$ (15 mol%, 0.015 mmol, 5.5 mg), **L8** (18 mol%, 0.018 mmol, 5.7 mg), Mn (4.0 equiv, 0.4 mmol, 22.0 mg), carbamoyl chloride **1a** (1.0 equiv, 0.1 mmol, 32.9 mg) and TEMPO (1.0 equiv, 0.1 mmol, 156.3 mg). Then the vial was transferred into glovebox. LiI (1.0 equiv, 0.1 mmol, 13.4 mg), DMA (0.4 mL), MeCN (0.1 mL) and 1-iodoheptane (3.0 equiv, 0.3 mmol, 49 μL) were added in sequence inside the glovebox. The vial was then taken out from the glovebox, sealed with parafilm, put into oil bath (30 °C) and stirred for 36 h. The reaction mixture was quenched with H_2O, filtered through a pad of Celite and extracted with EA, and GC analysis was conducted by using an aliquot of the resulting organic phase, dodecane was used as the internal standard. No product was observed on TLC and GC, only decarbonylation by-product could be obtained in 48% GC yield. Additionally, trace amount of TEMPO-adduct product could be detected by GC and confirmed by GC-MS.

1.9 Crystallographic Data for Compound 3y

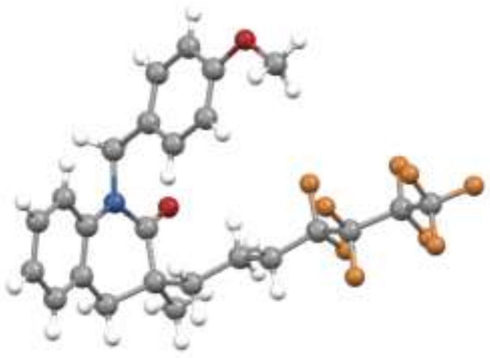

Supplementary Figure 46. Crystal data and structure refinement for **3y** (CCDC 2158471)

Supplementary Table 1. Crystal data and structure refinement for mo_d8v21423_0m.

Identification code	mo_d8v21423_0m	
Empirical formula	C25 H24 F9 N O2	
Formula weight	541.45	
Temperature	293(2) K	
Wavelength	0.71073 Å	
Crystal system	Monoclinic	
Space group	P 21	
Unit cell dimensions	a = 5.86790(10) Å	α= 90 °.
	b = 11.7219(3) Å	β= 92.2610(10) °.
	c = 36.2596(10) Å	γ = 90 °.
Volume	2492.10(10) Å^3	
Z	4	
Density (calculated)	1.443 Mg/m^3	
Absorption coefficient	0.137 mm^{-1}	
F(000)	1112	
Crystal size	0.200 x 0.150 x 0.110 mm^3	
Theta range for data collection	2.811 to 25.996 °.	
Index ranges	–7<=h<=7, –14<=k<=14, –39<=l<=44	
Reflections collected	35253	
Independent reflections	9732 [R(int) = 0.0312]	
Completeness to theta = 25.242 °	99.1 %	
Absorption correction	Semi–empirical from equivalents	
Max. and min. transmission	0.7456 and 0.6418	
Refinement method	Full–matrix least–squares on F^2	
Data / restraints / parameters	9732 / 1 / 672	

Goodness–of–fit on F^2	1.029
Final R indices [I>2sigma(I)]	R1 = 0.0492, wR2 = 0.1295
R indices (all data)	R1 = 0.0635, wR2 = 0.1428
Absolute structure parameter	–0.01(18)
Extinction coefficient	0.037(5)
Largest diff. peak and hole	0.257 and –0.202 e.$Å^{-3}$

1.10 NMR Spectroscopic Data

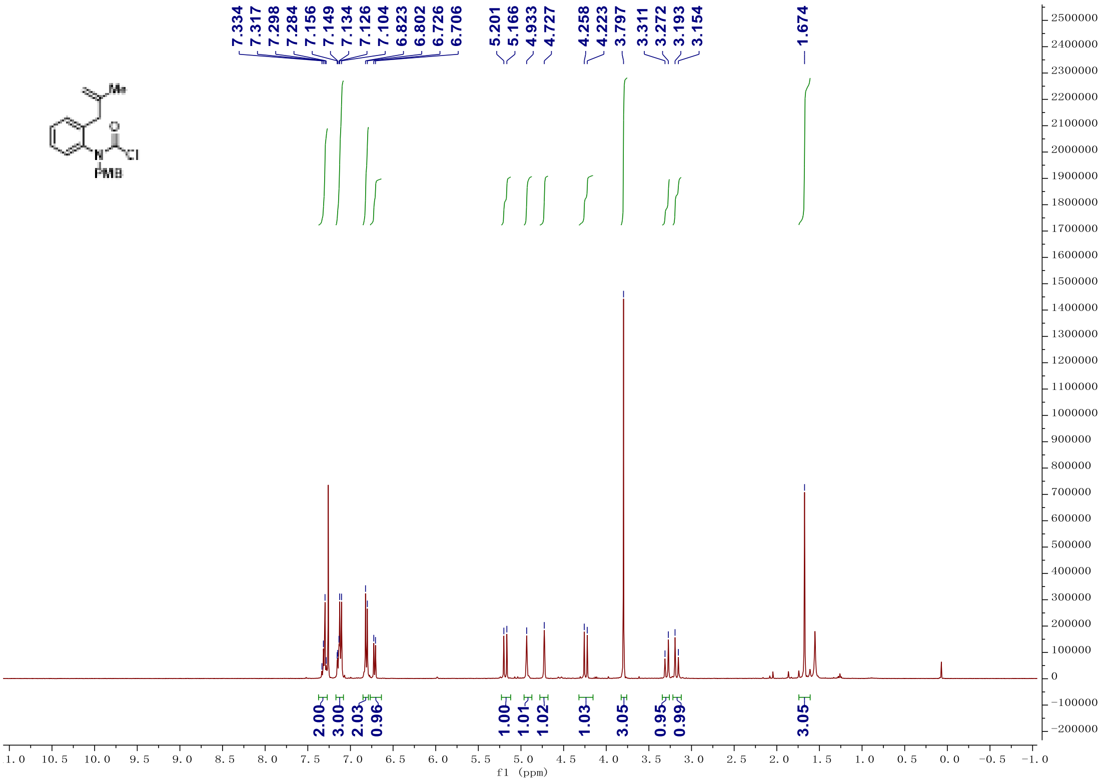

Supplementary Figure 47. ^{1}H NMR-spectrum (400 MHz, $CDCl_3$) of **1a**

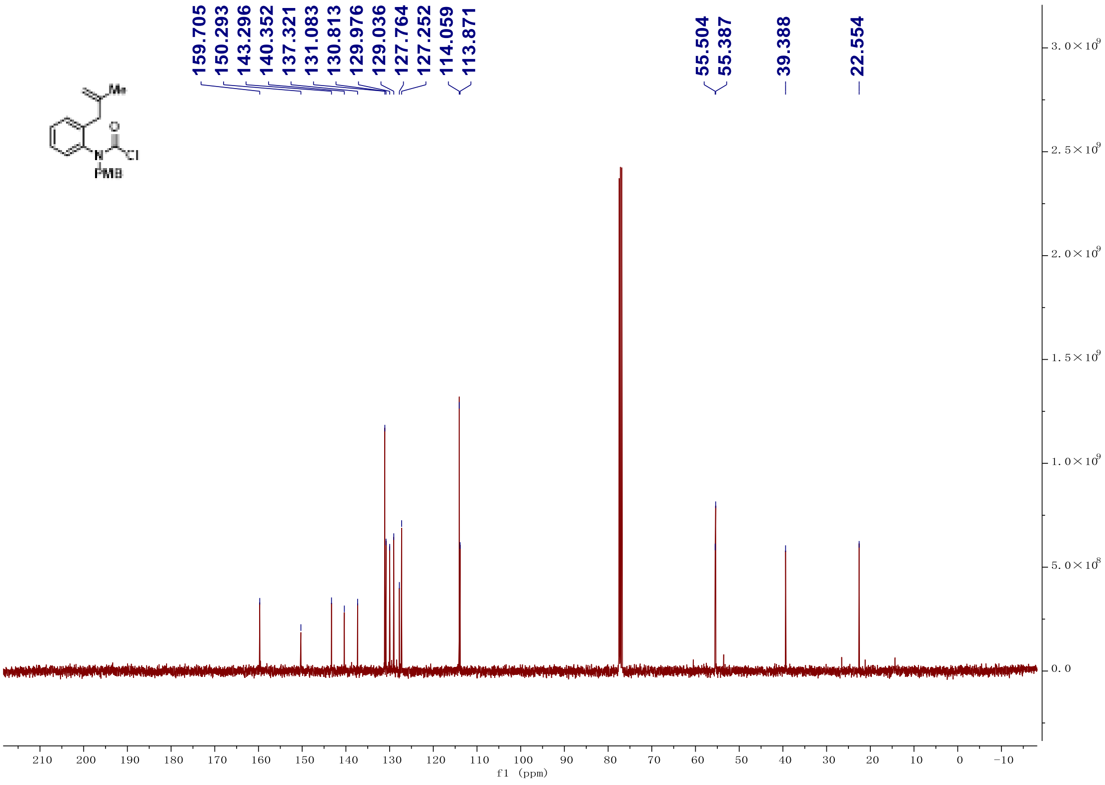

Supplementary Figure 48. ^{13}C NMR-spectrum (100 MHz, $CDCl_3$) of **1a**

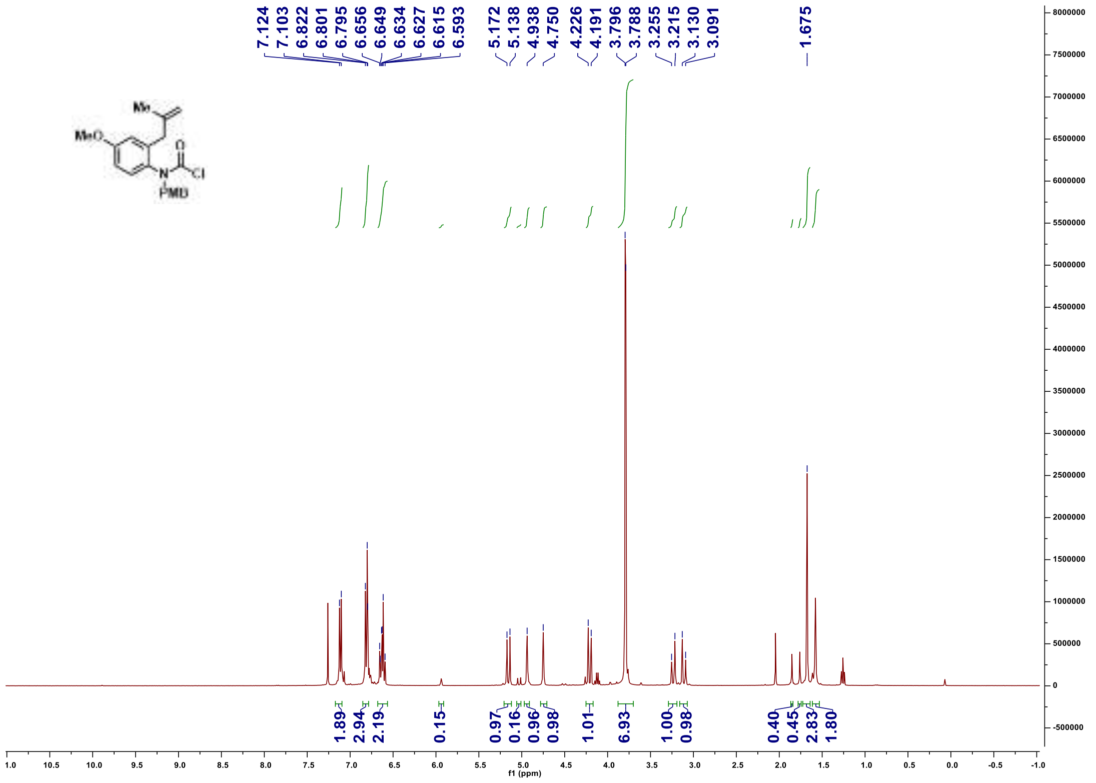

Supplementary Figure 49. ^{1}H NMR-spectrum (400 MHz, $CDCl_3$) of **1b**

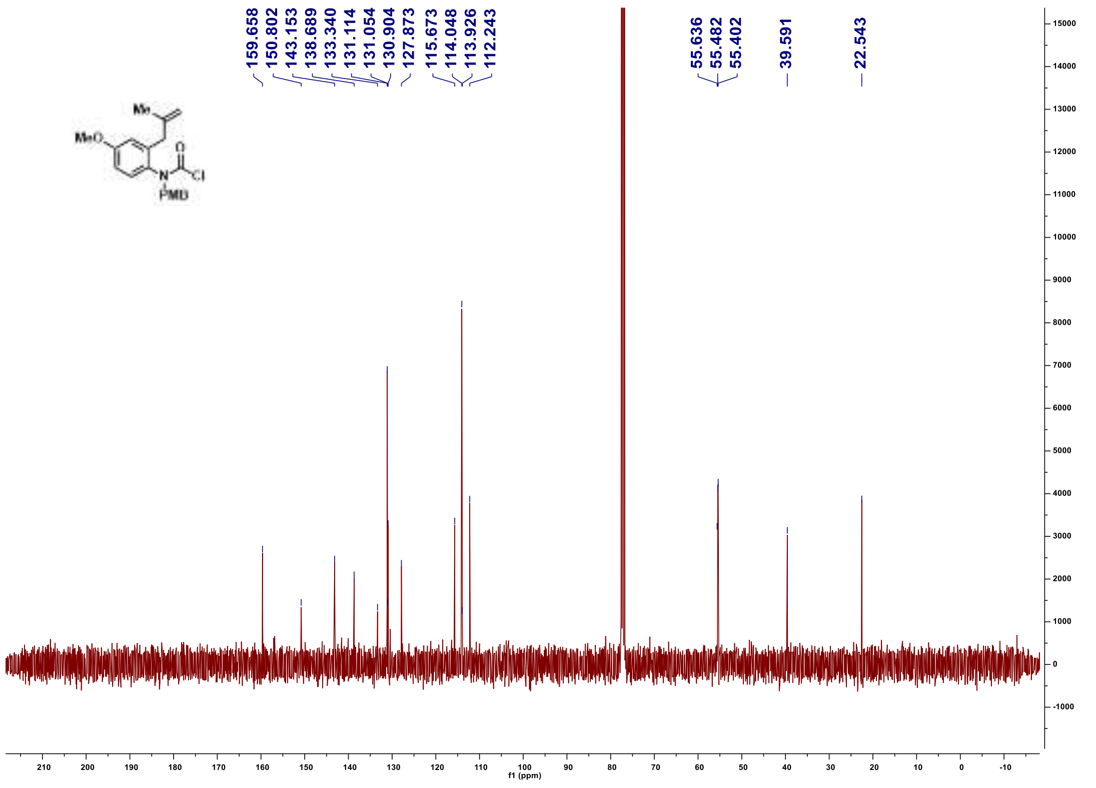

Supplementary Figure 50. ^{13}C NMR-spectrum (100 MHz, $CDCl_3$) of **1b**

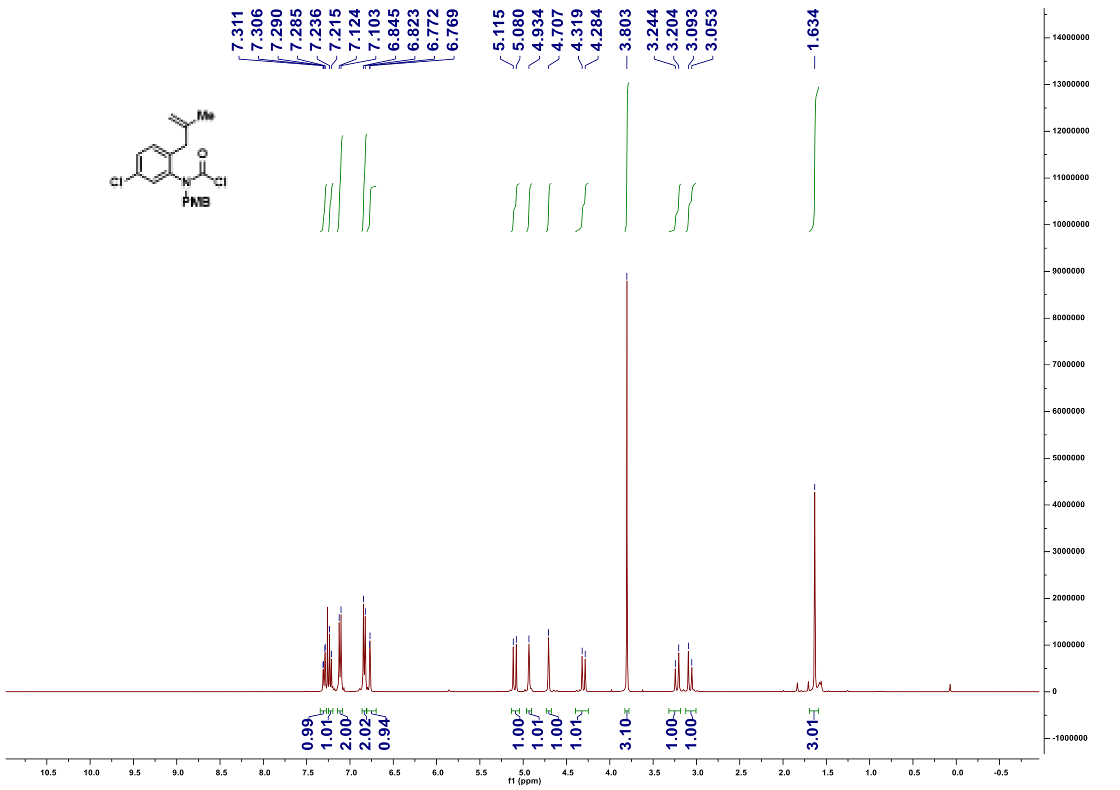

Supplementary Figure 51. ^{1}H NMR-spectrum (400 MHz, $CDCl_3$) of **1c**

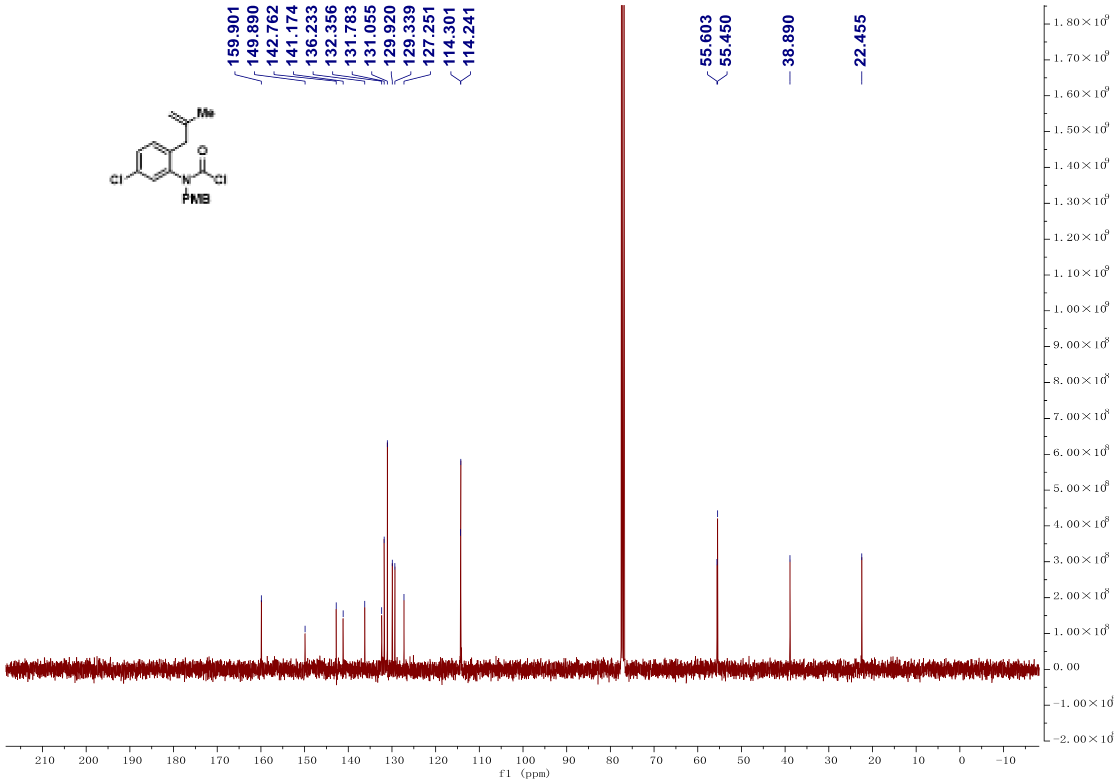

Supplementary Figure 52. ^{13}C NMR-spectrum (100 MHz, $CDCl_3$) of **1c**

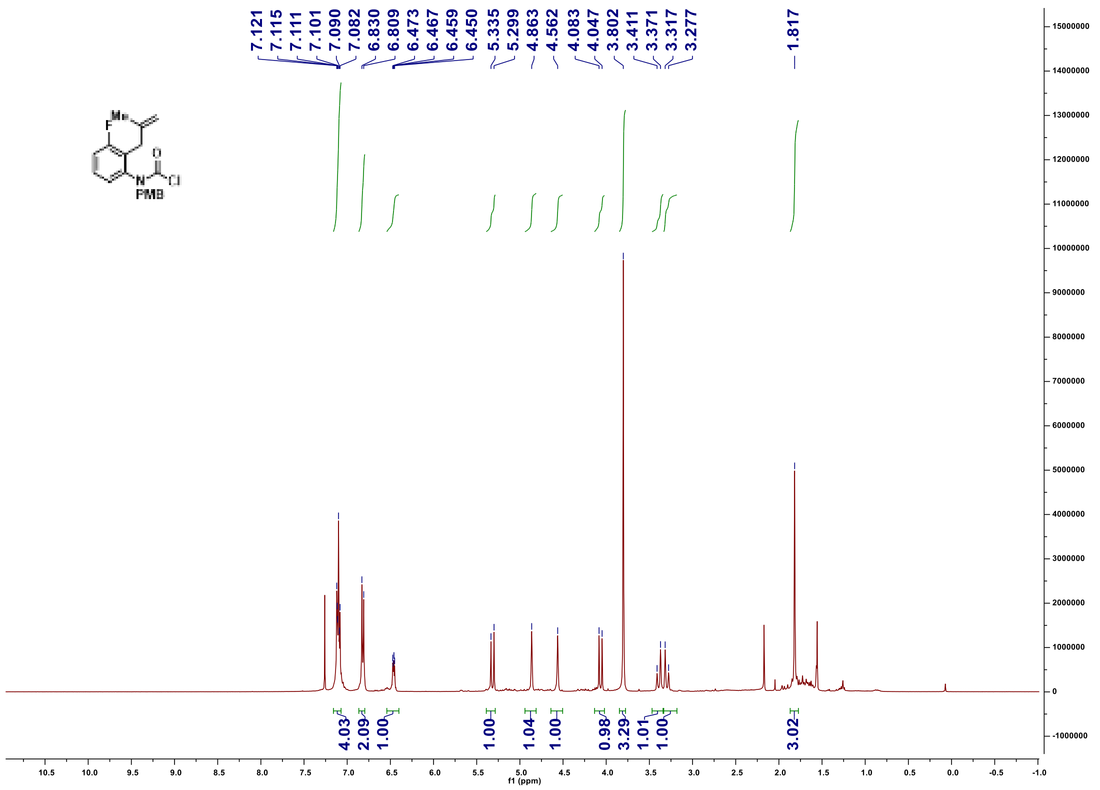

Supplementary Figure 53. ^{1}H NMR-spectrum (400 MHz, $CDCl_3$) of **1d**

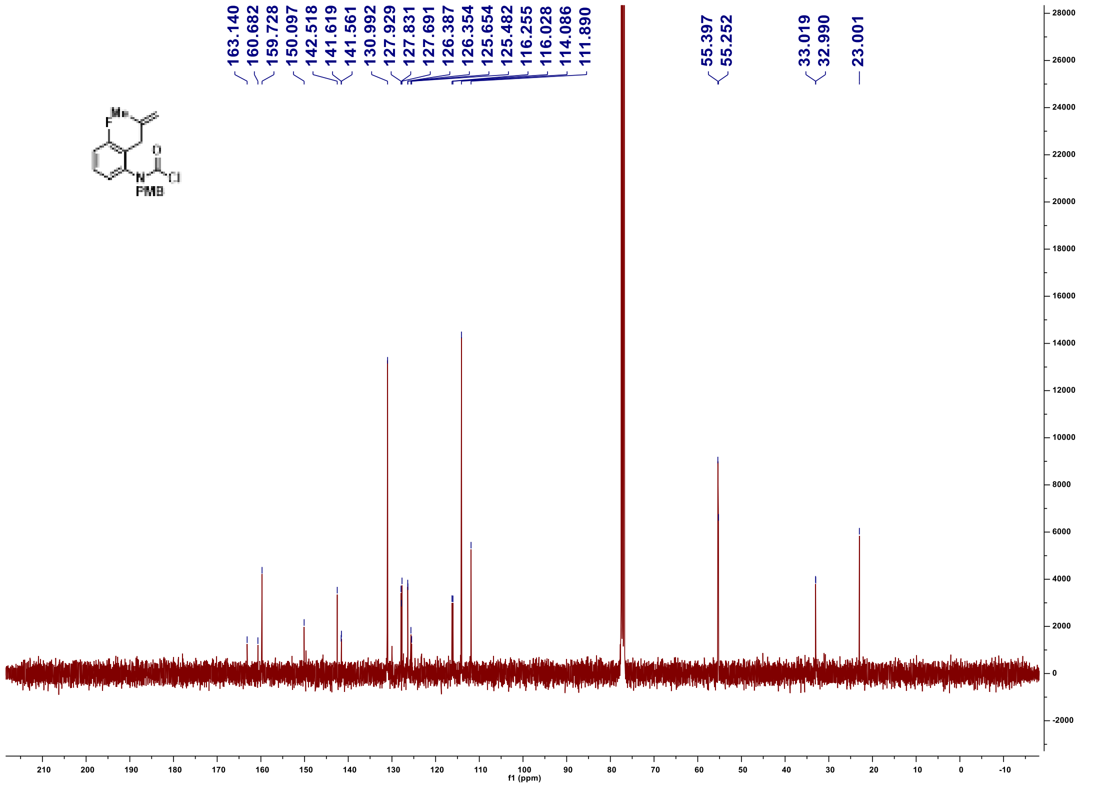

Supplementary Figure 54. ^{13}C NMR-spectrum (100 MHz, $CDCl_3$) of **1d**

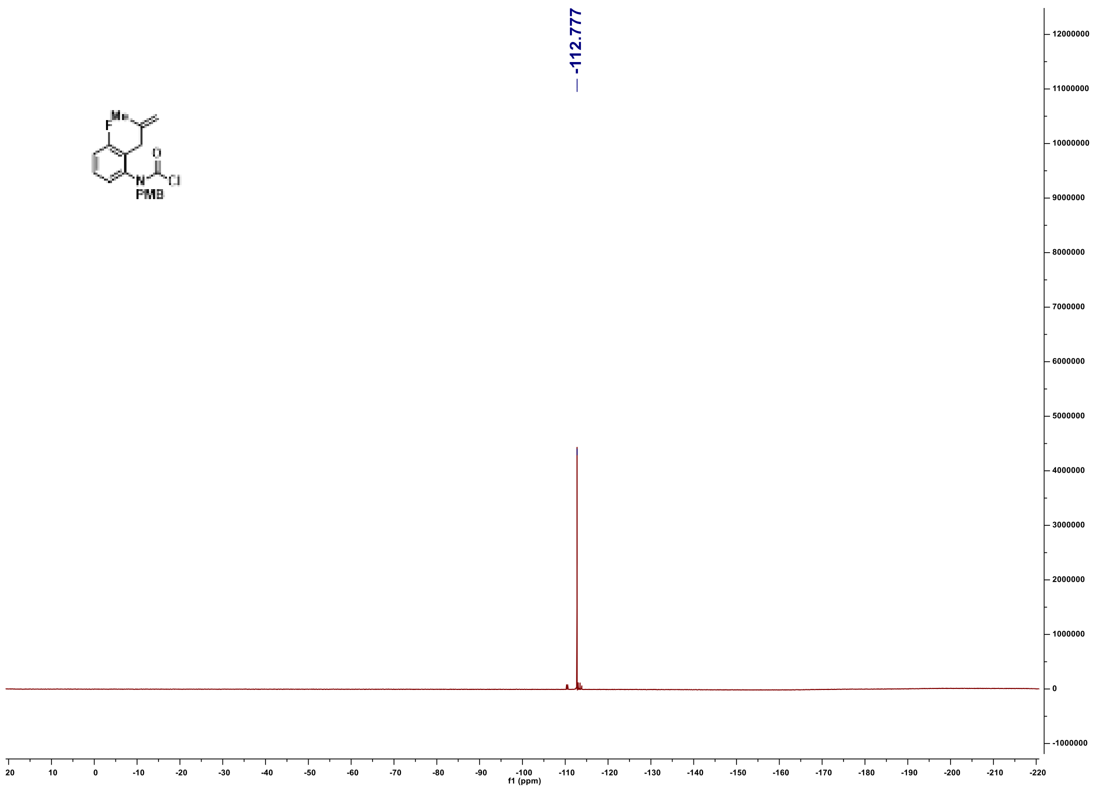

Supplementary Figure 55. ^{19}F NMR-spectrum (376 MHz, $CDCl_3$) of **1d**

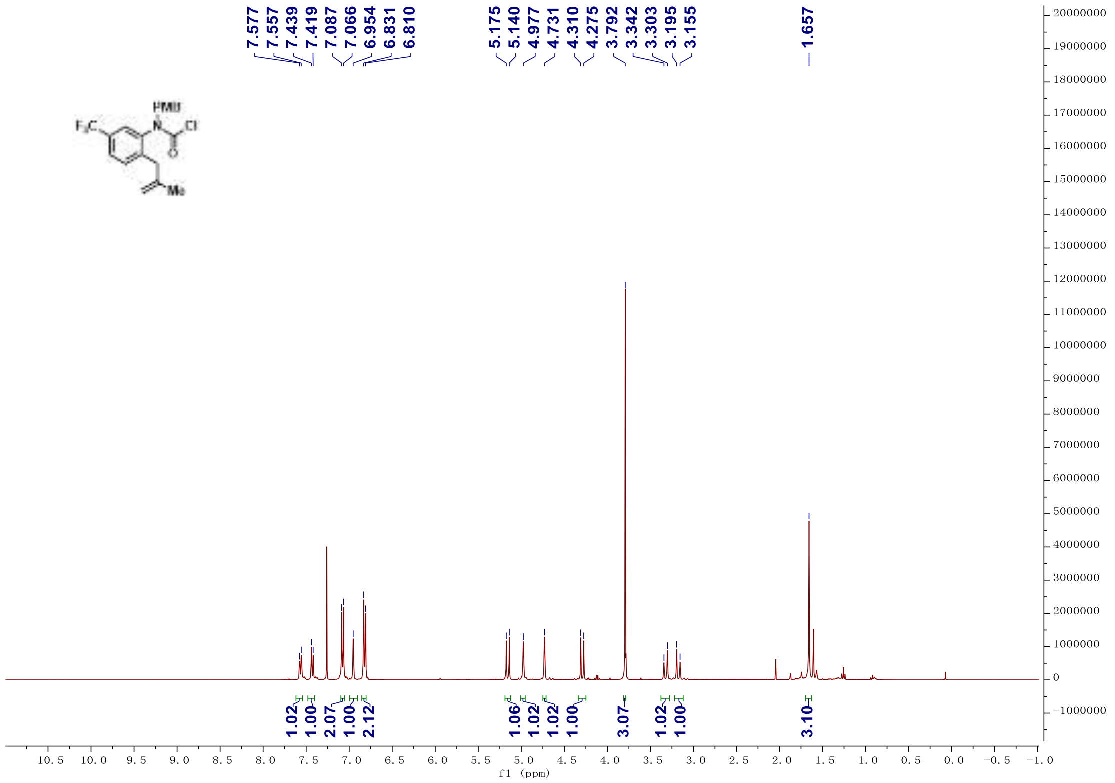

Supplementary Figure 56. ^{1}H NMR-spectrum (400 MHz, $CDCl_3$) of **1e**

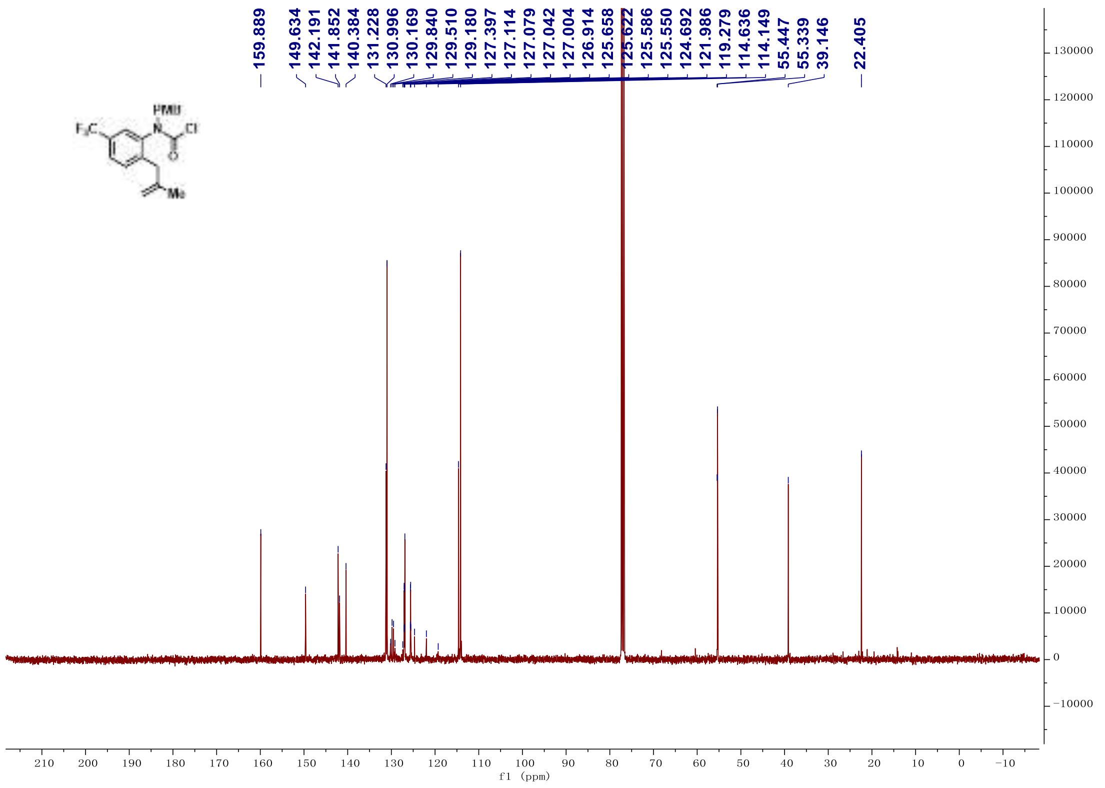

Supplementary Figure 57. ^{13}C NMR-spectrum (100 MHz, $CDCl_3$) of **1e**

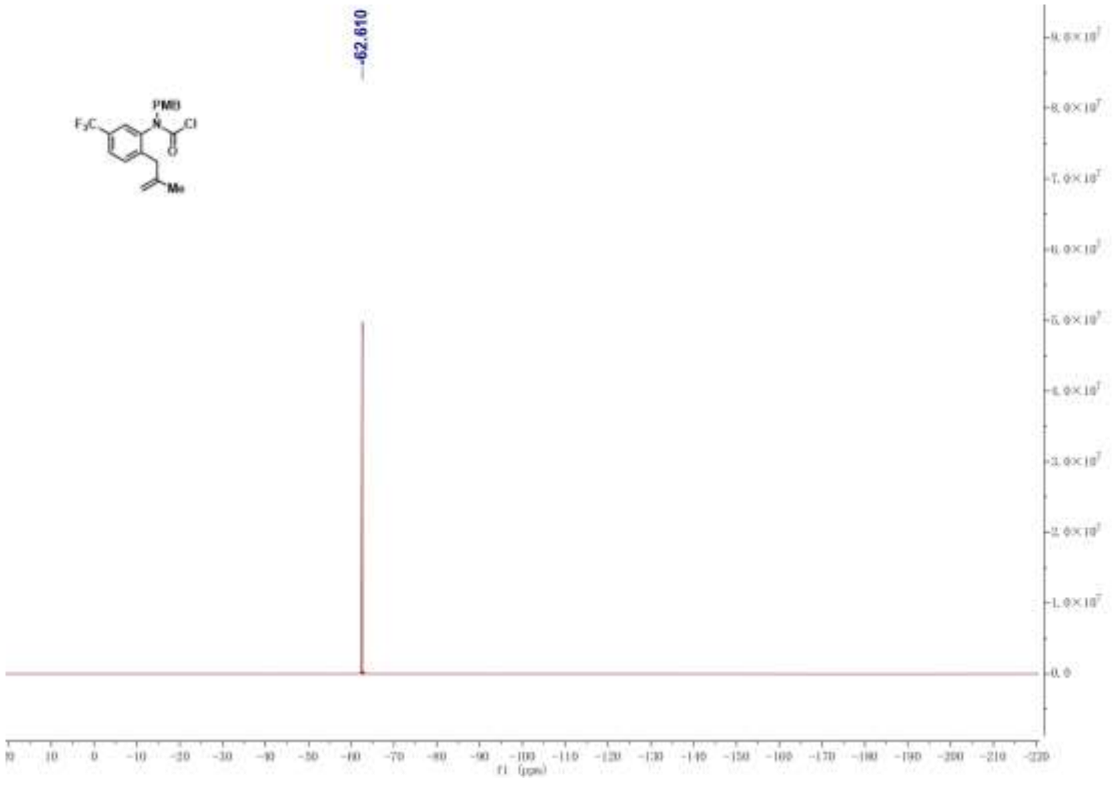

Supplementary Figure 58. ^{19}F NMR-spectrum (376 MHz, $CDCl_3$) of **1e**

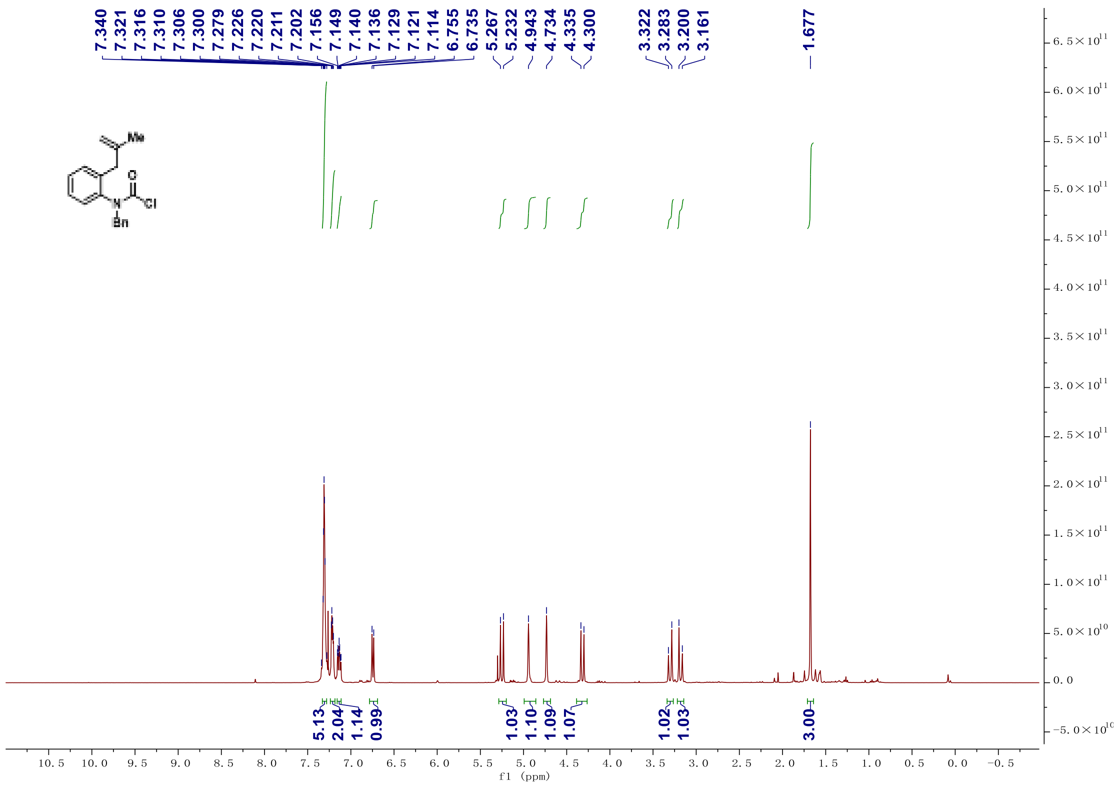

Supplementary Figure 59. ^{1}H NMR-spectrum (400 MHz, $CDCl_3$) of **1f**

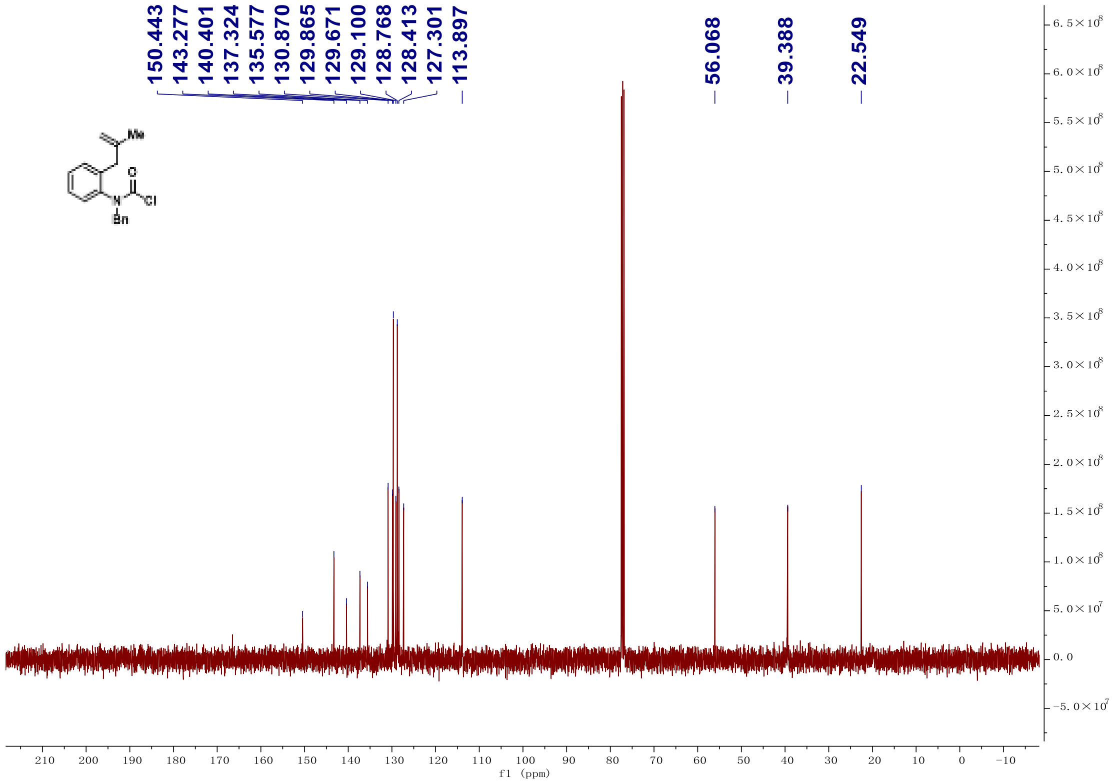

Supplementary Figure 60. ^{13}C NMR-spectrum (100 MHz, $CDCl_3$) of **1f**

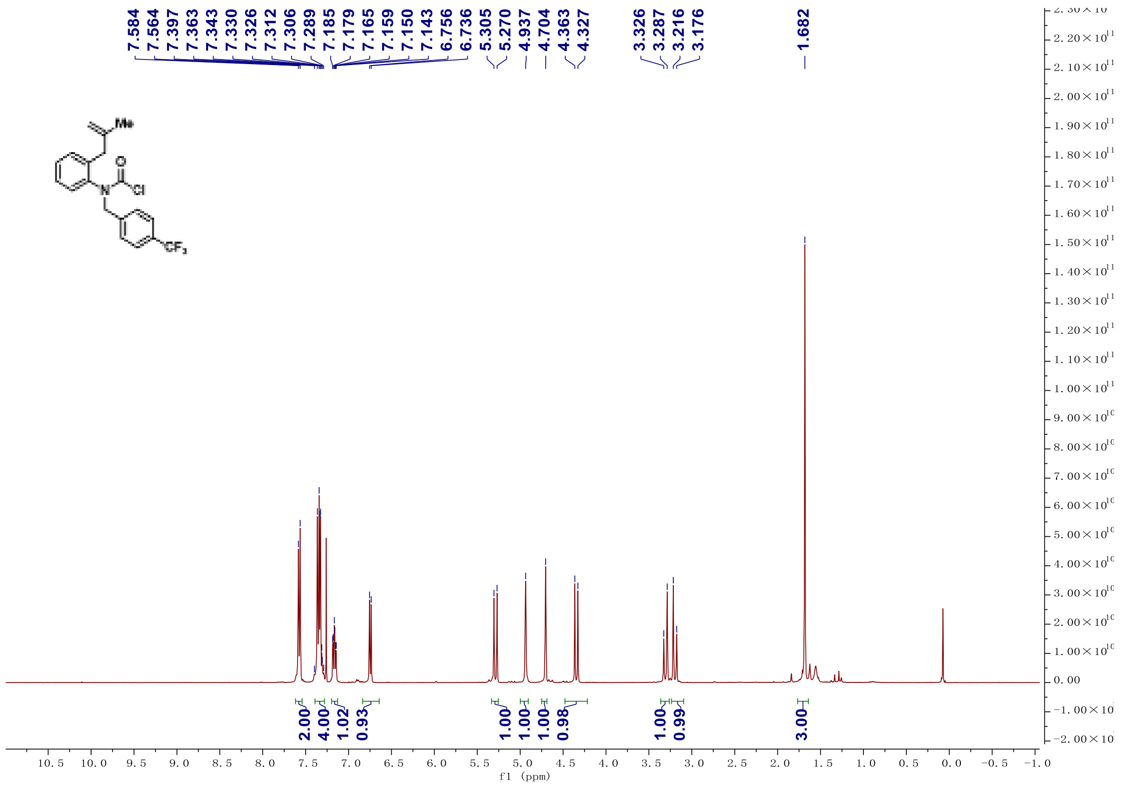

Supplementary Figure 61. ^{1}H NMR-spectrum (400 MHz, $CDCl_3$) of **1g**

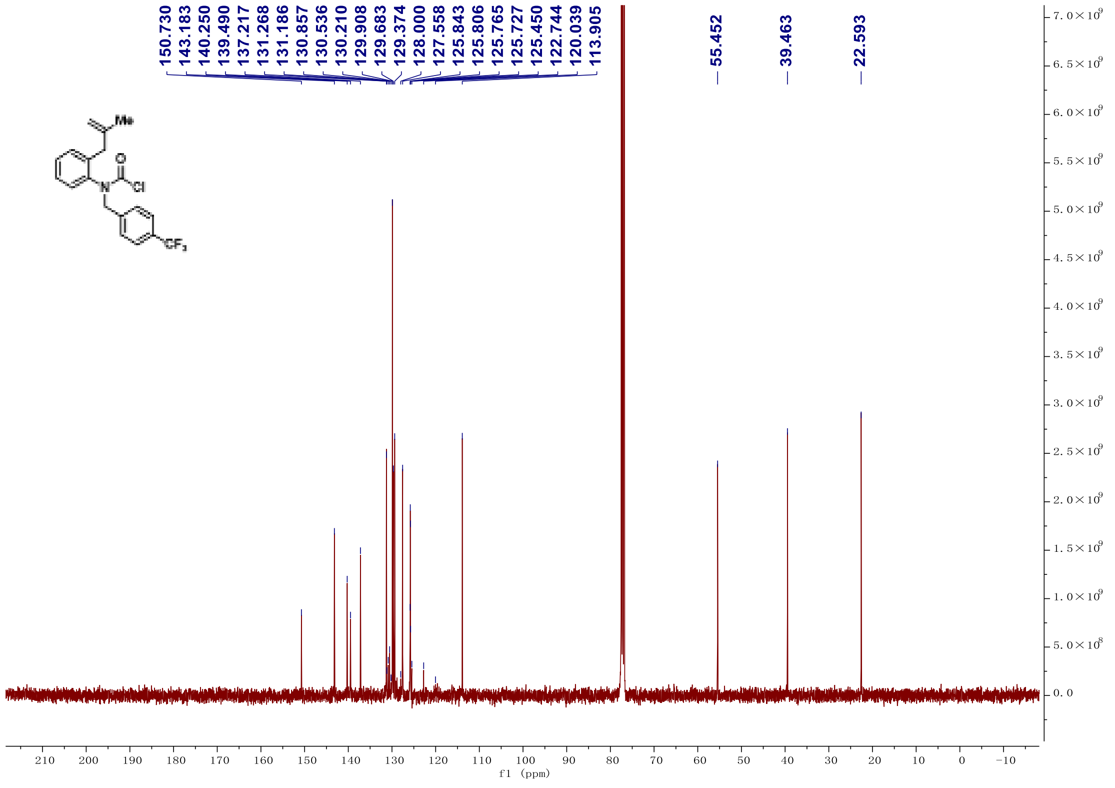

Supplementary Figure 62. ^{13}C NMR-spectrum (100 MHz, $CDCl_3$) of **1g**

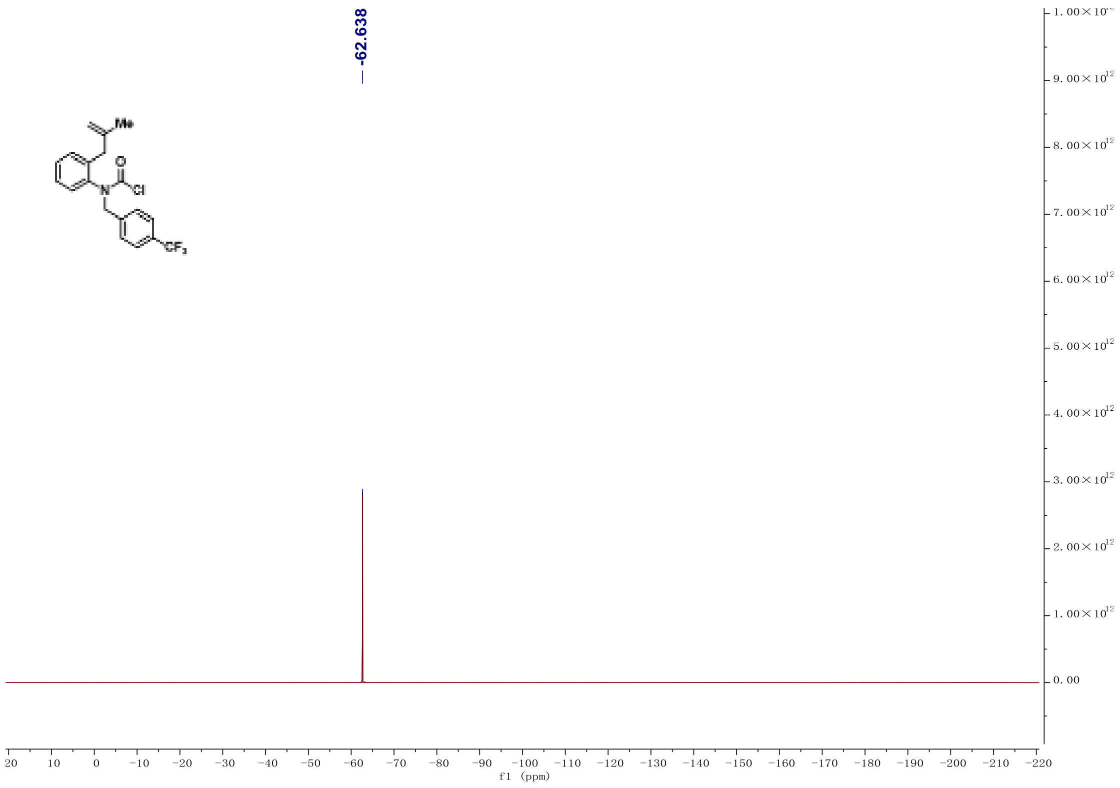

Supplementary Figure 63. ^{19}F NMR-spectrum (376 MHz, $CDCl_3$) of **1g**

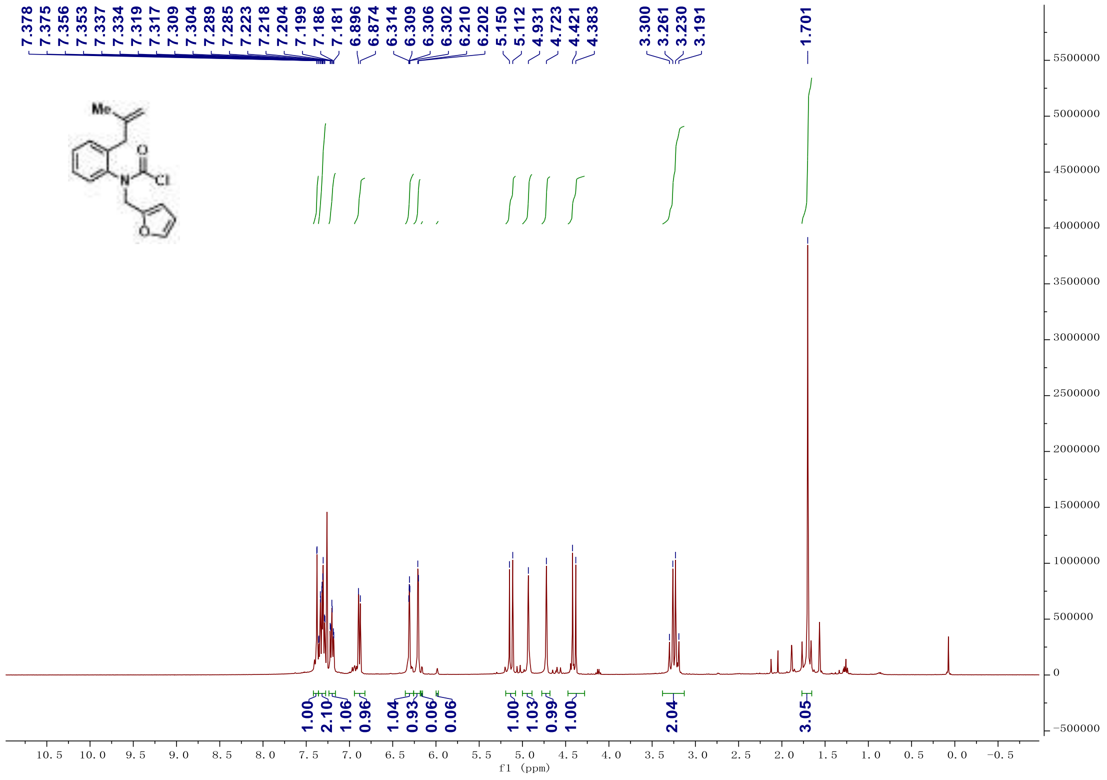

Supplementary Figure 64. ^{1}H NMR-spectrum (400 MHz, $CDCl_3$) of **1h**

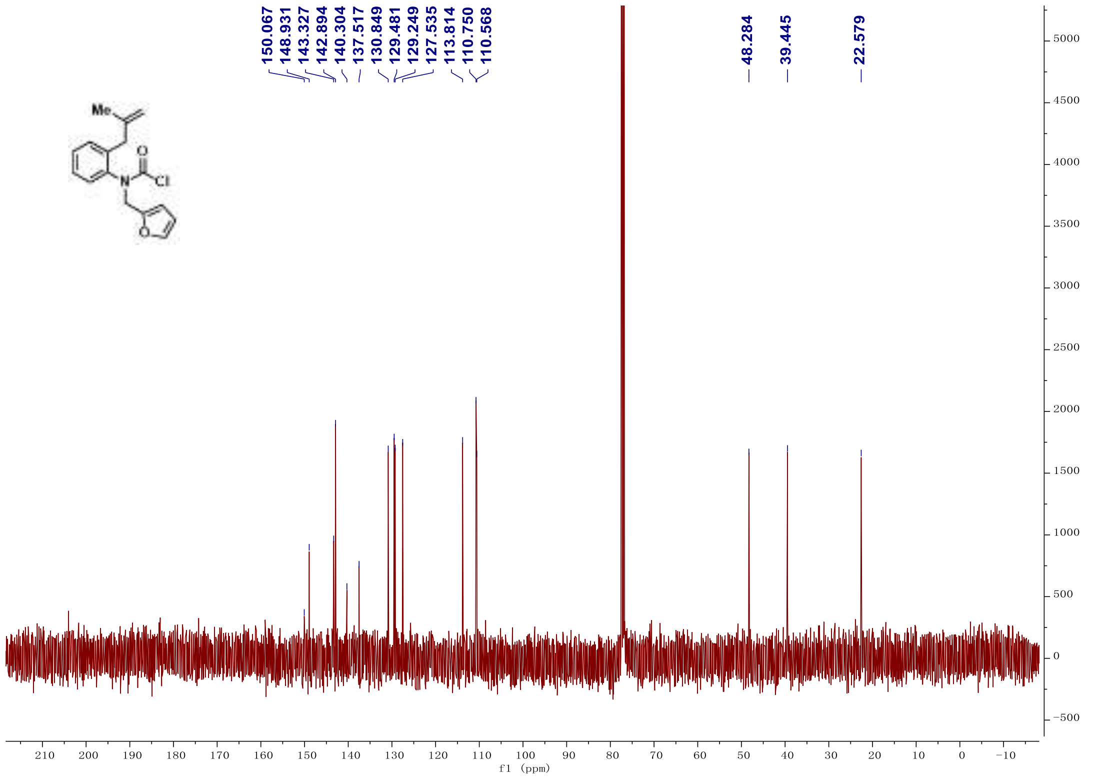

Supplementary Figure 65. ^{13}C NMR-spectrum (100 MHz, $CDCl_3$) of **1h**

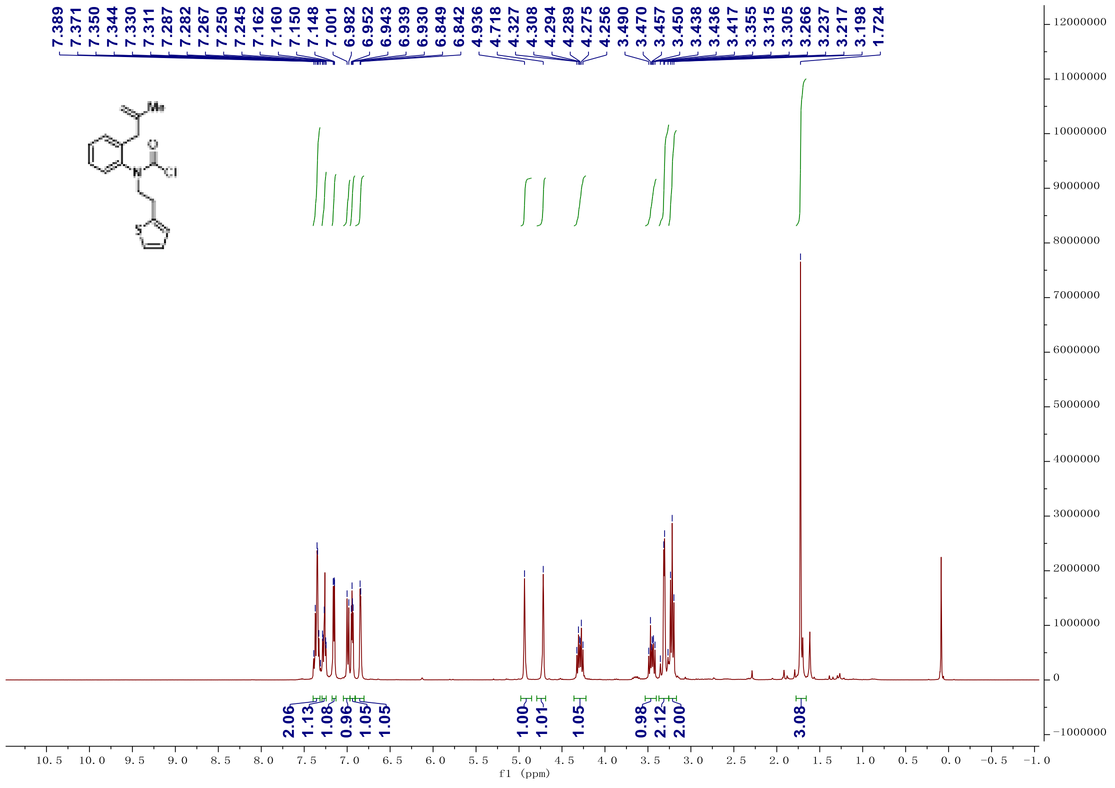

Supplementary Figure 66. ^{1}H NMR-spectrum (400 MHz, $CDCl_3$) of **1i**

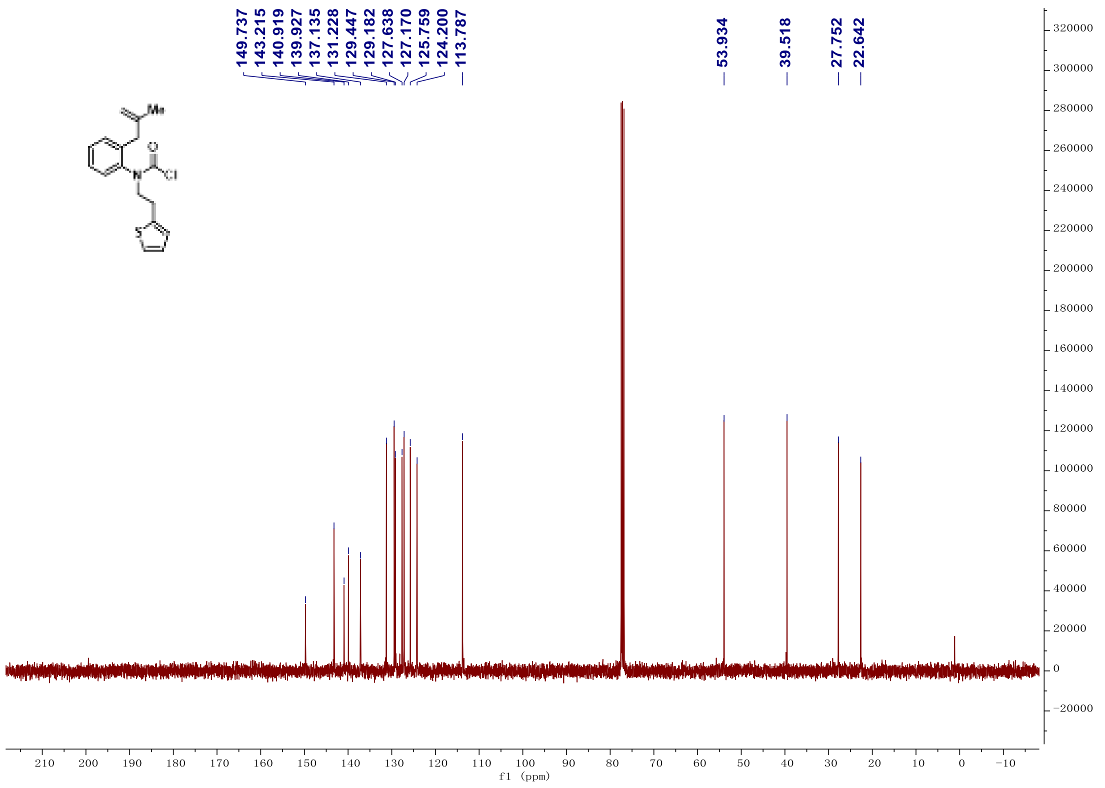

Supplementary Figure 67. ^{13}C NMR-spectrum (100 MHz, $CDCl_3$) of **1i**

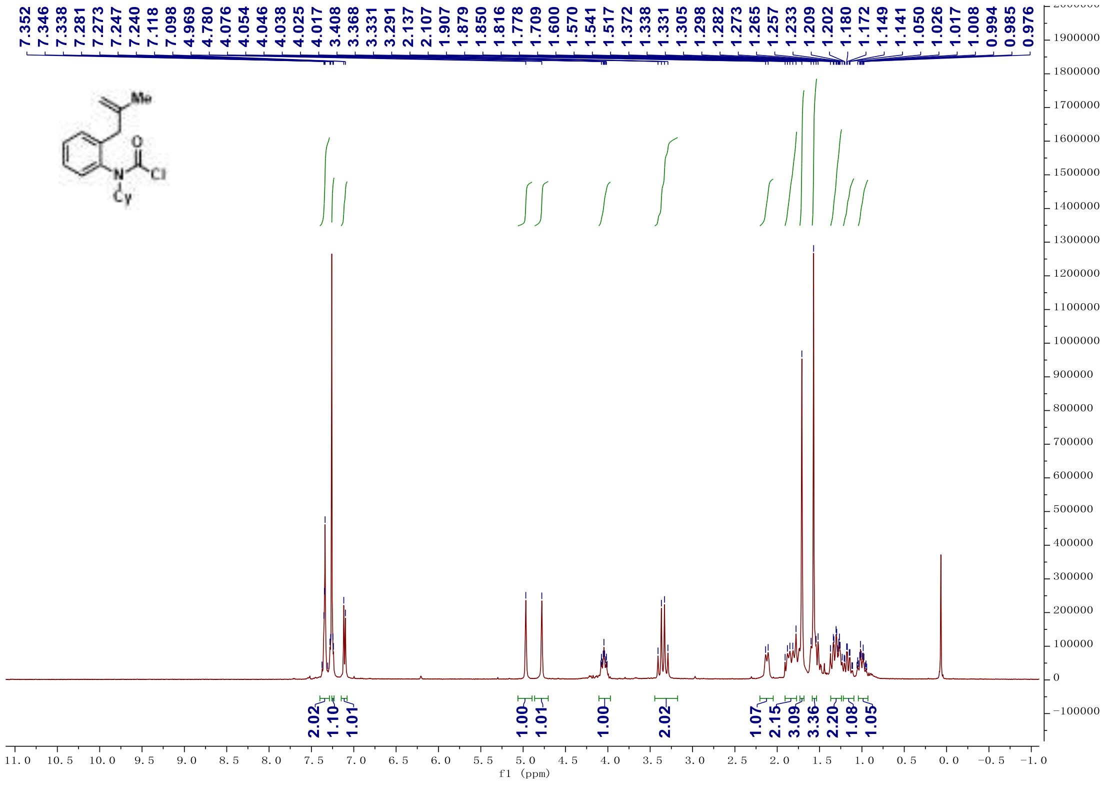

Supplementary Figure 68. ^{1}**H NMR**-spectrum (400 MHz, $CDCl_3$) of **1j**

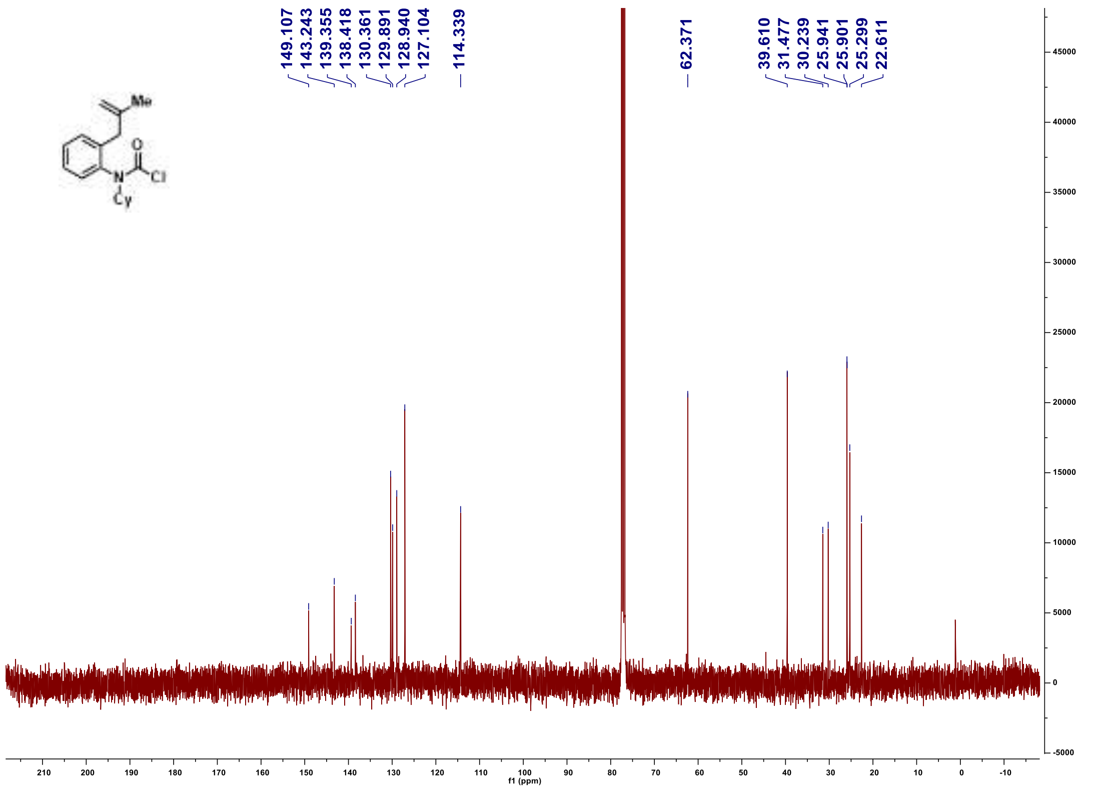

Supplementary Figure 69. ^{13}C NMR-spectrum (100 MHz, $CDCl_3$) of **1j**

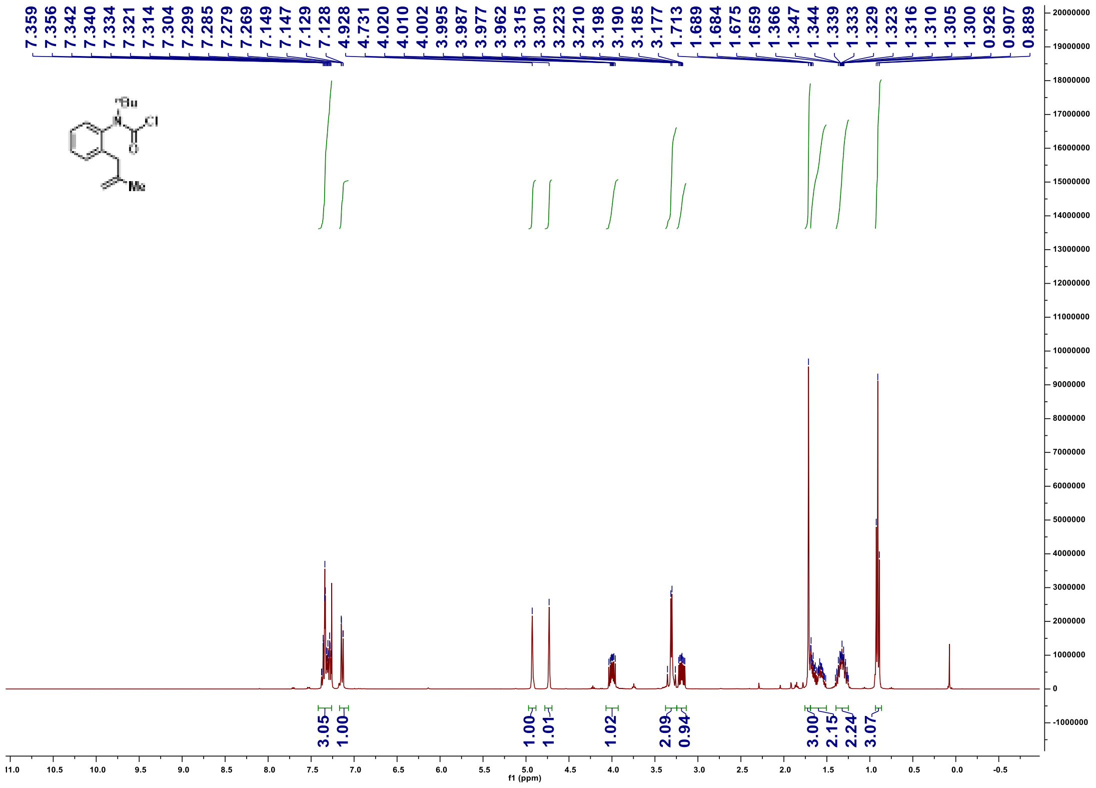

Supplementary Figure 70. ^{1}H NMR-spectrum (400 MHz, $CDCl_3$) of **1k**

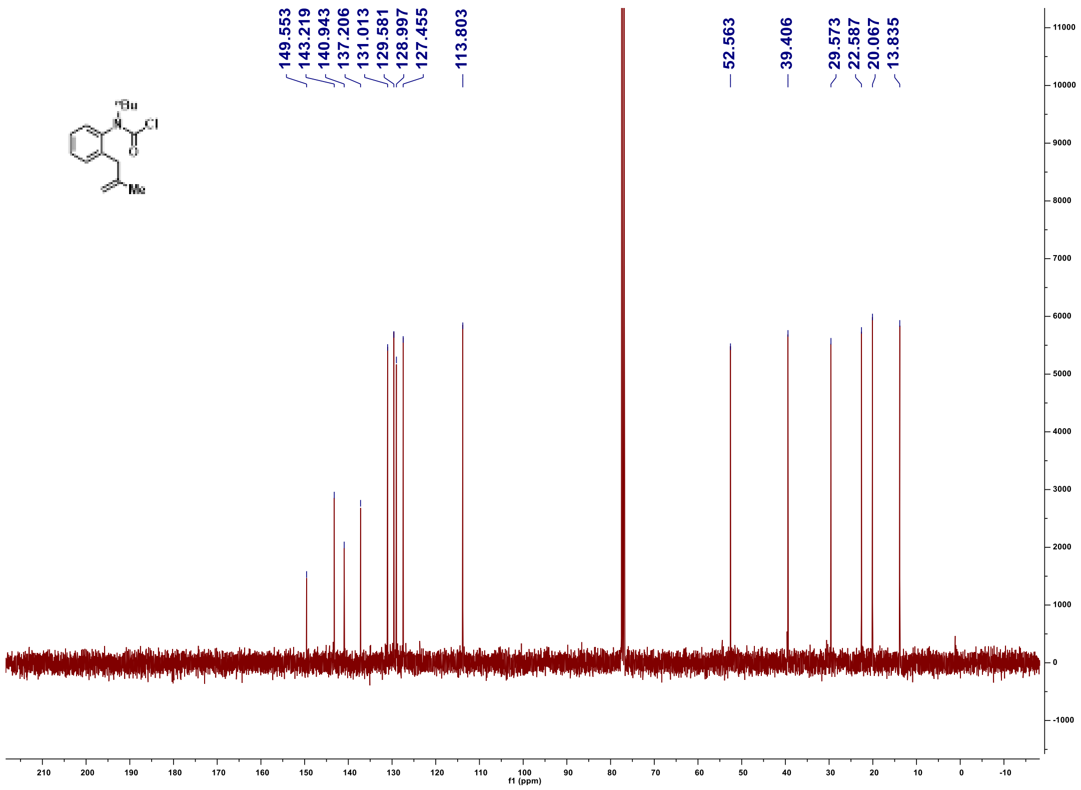

Supplementary Figure 71. ^{13}C NMR-spectrum (100 MHz, $CDCl_3$) of **1k**

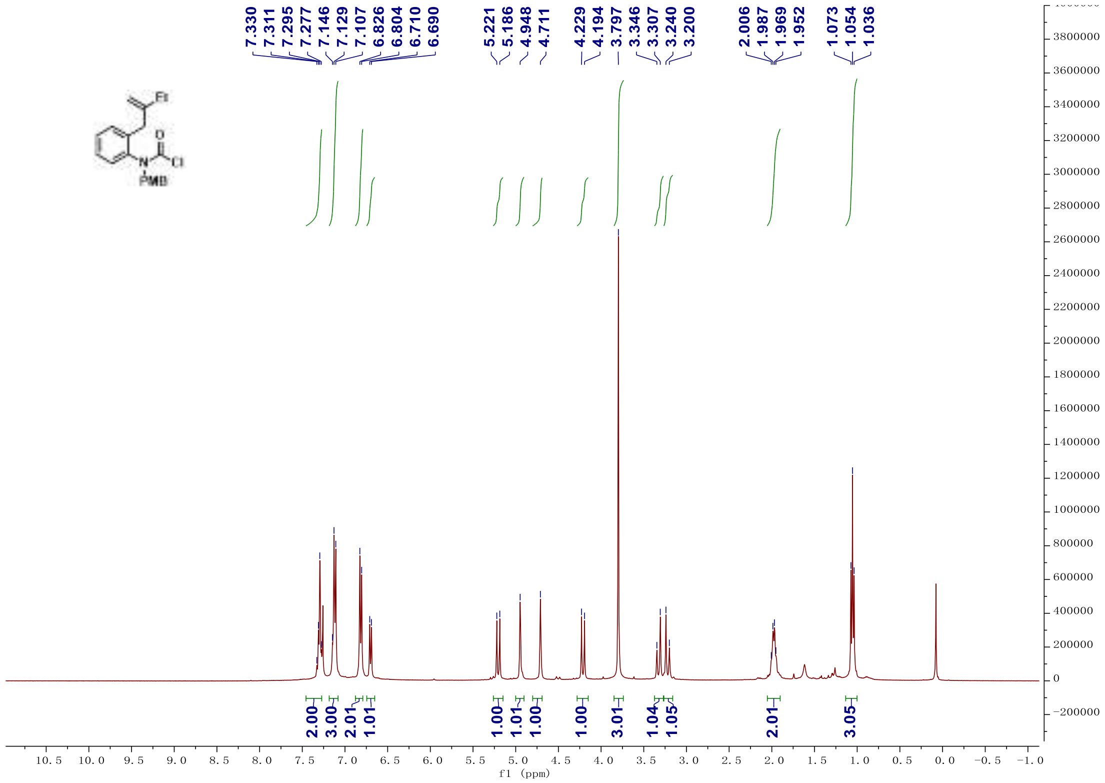

Supplementary Figure 72. ^{1}H NMR-spectrum (400 MHz, $CDCl_3$) of **1l**

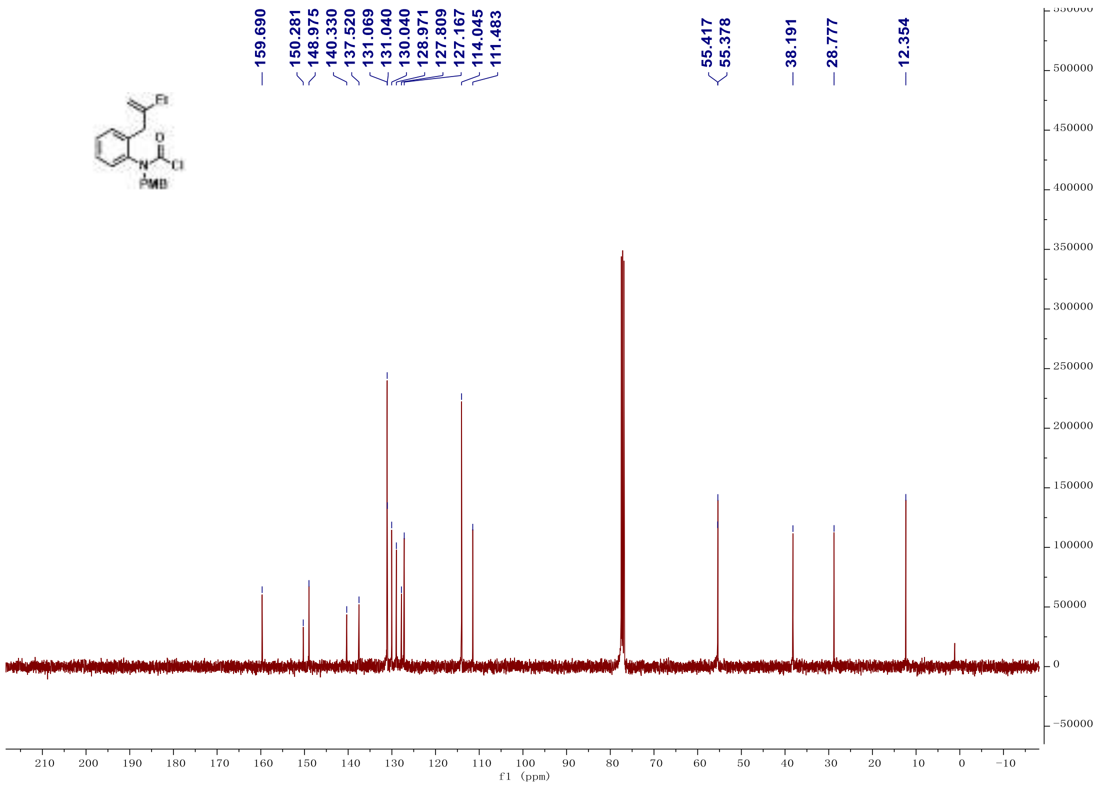

Supplementary Figure 73. ^{13}C NMR-spectrum (100 MHz, $CDCl_3$) of **1l**

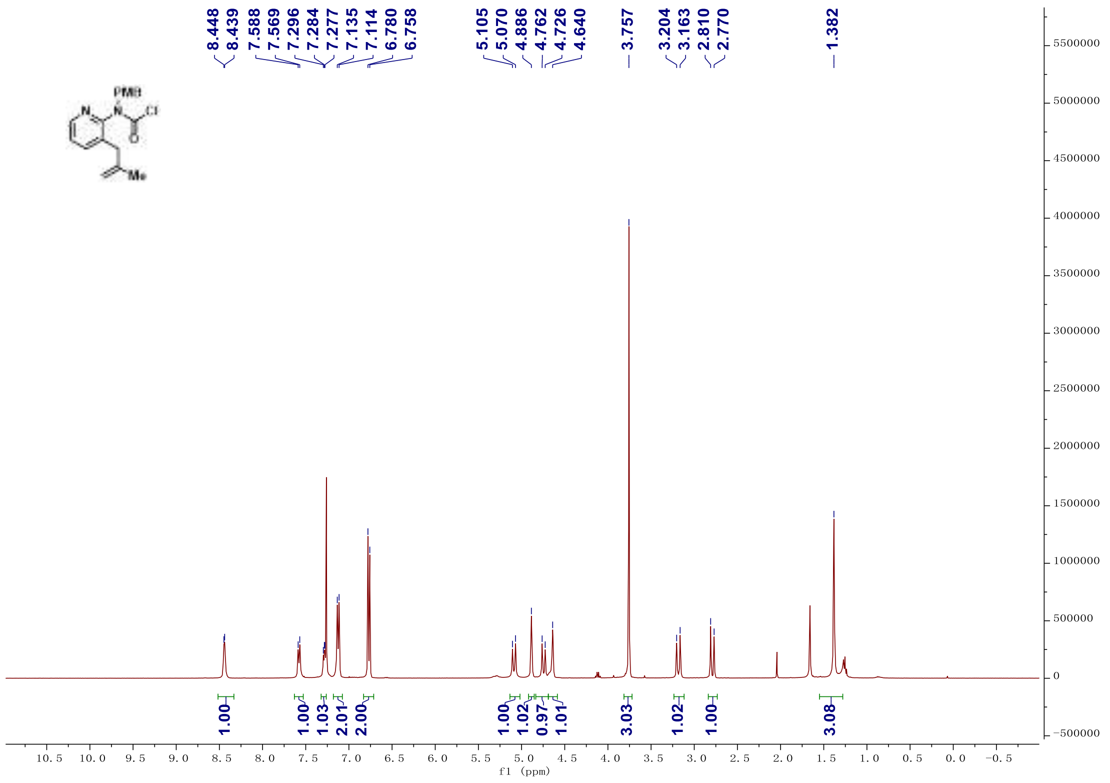

Supplementary Figure 74. ^{1}H NMR-spectrum (400 MHz, $CDCl_3$) of **1m**

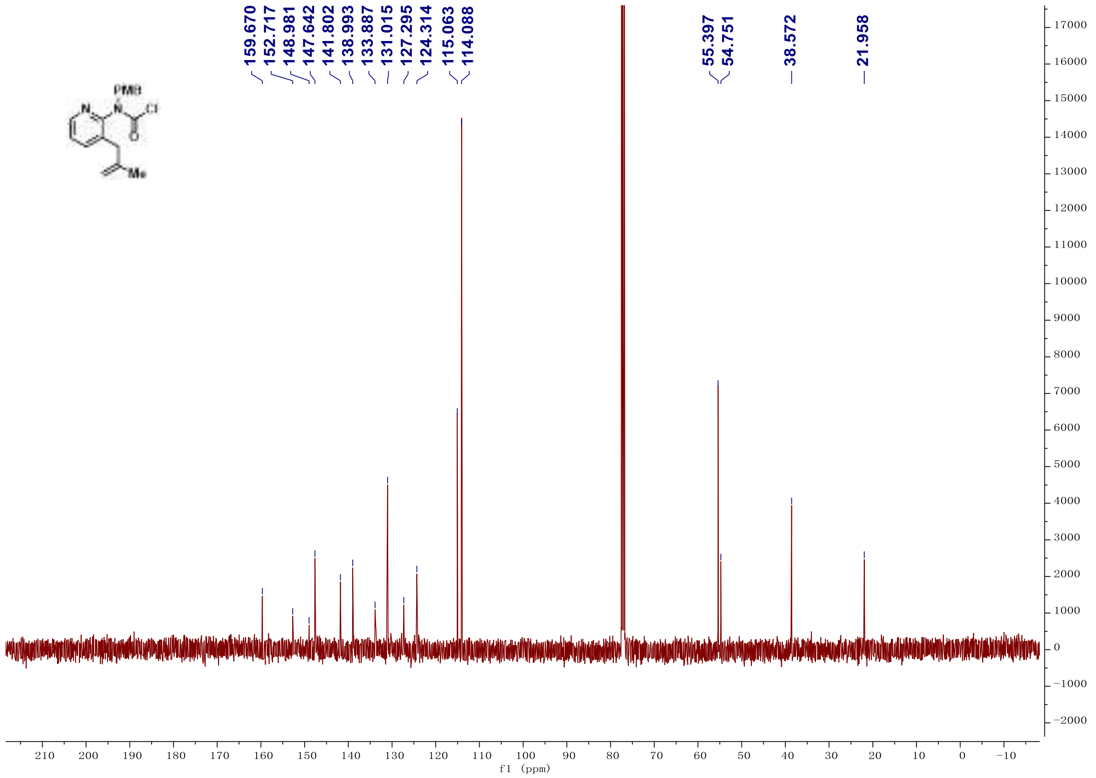

Supplementary Figure 75. ^{13}C NMR-spectrum (100 MHz, $CDCl_3$) of **1m**

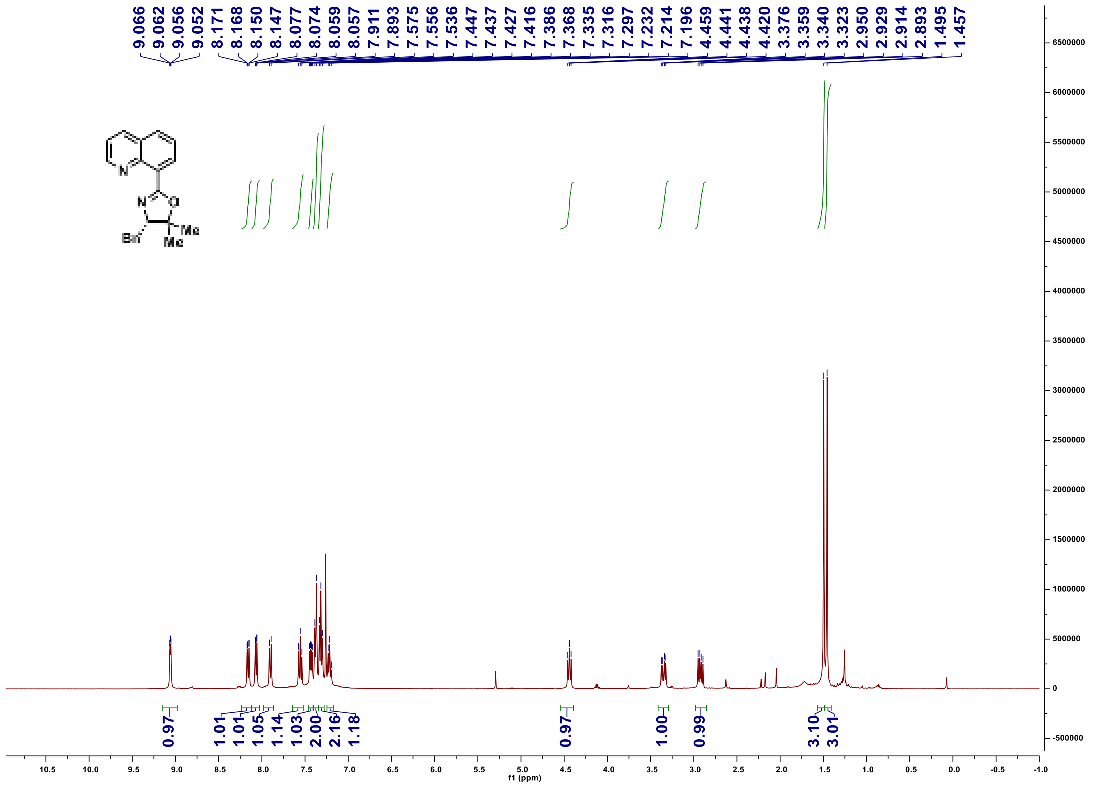

Supplementary Figure 76. ^{1}H NMR-spectrum (400 MHz, $CDCl_3$ with K_2CO_3) of **L8**

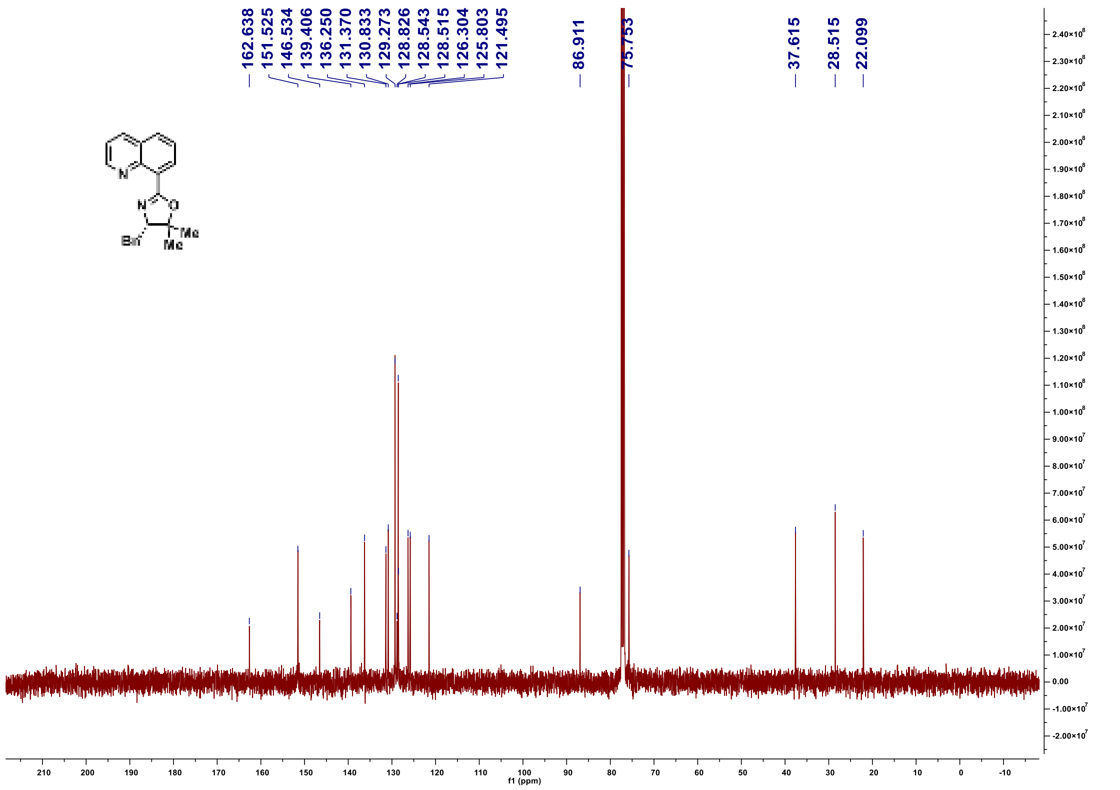

Supplementary Figure 77. ^{13}C NMR-spectrum (100 MHz, $CDCl_3$ with K_2CO_3) of **L8**

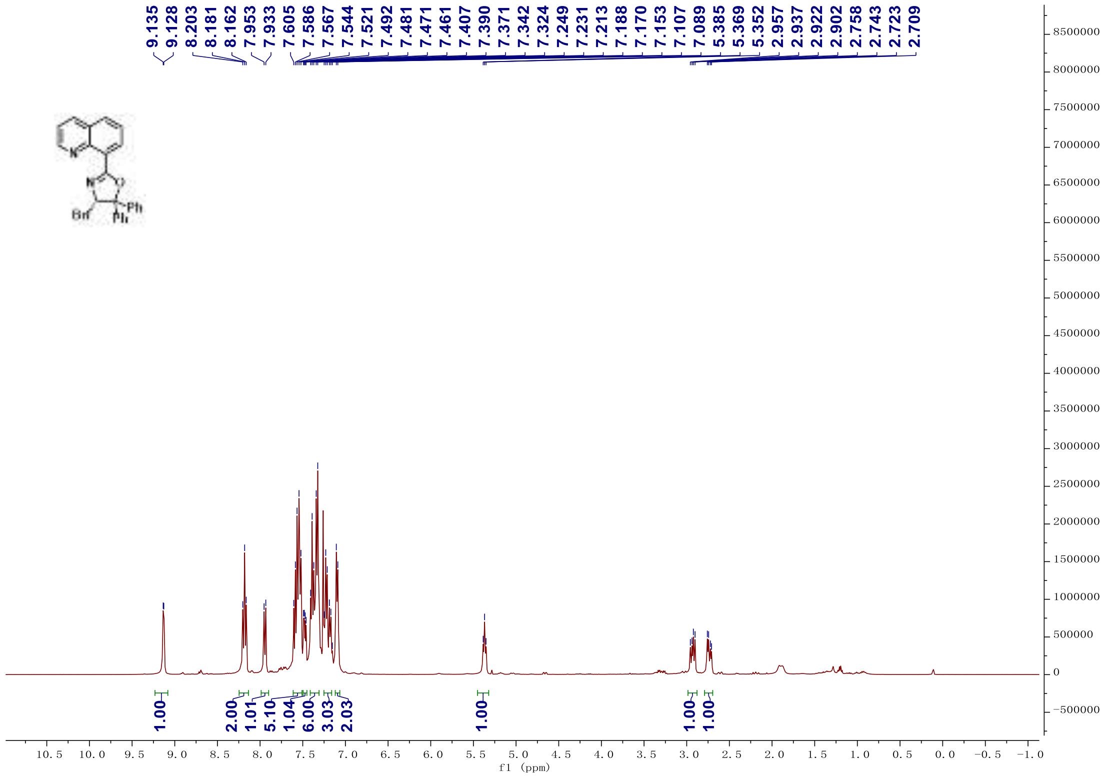

Supplementary Figure 78. ^{1}H NMR-spectrum (400 MHz, $CDCl_3$ with K_2CO_3) of **L9**

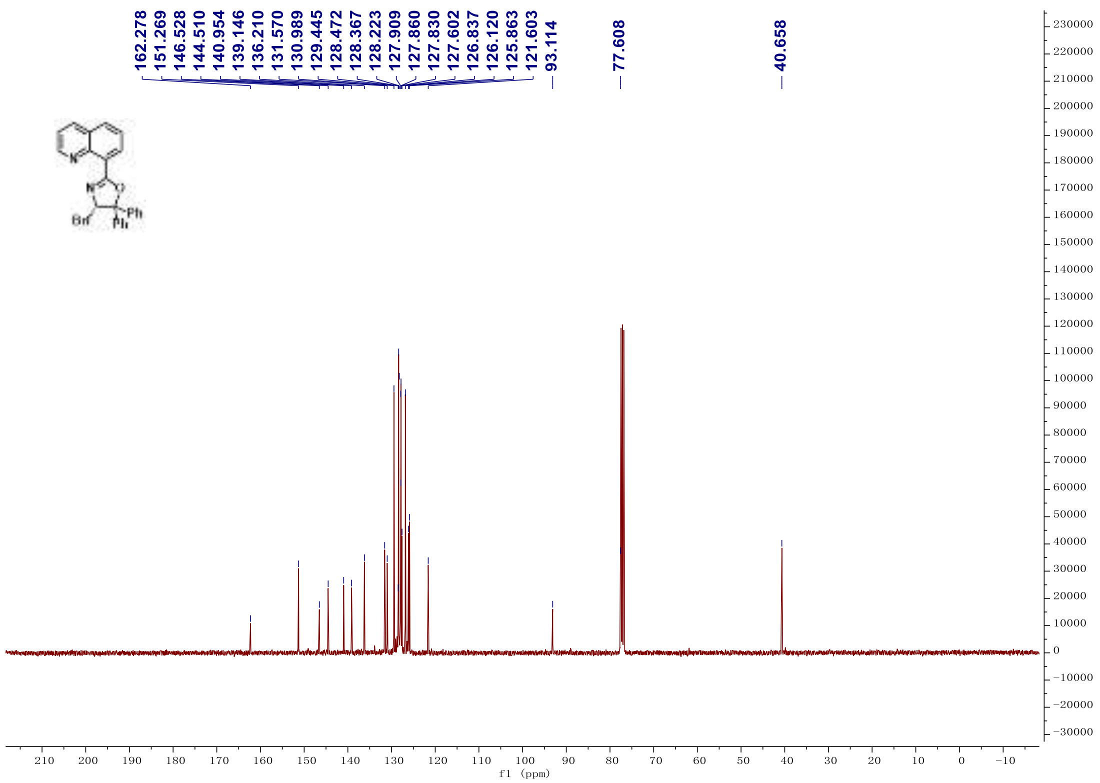

Supplementary Figure 79. ^{13}C NMR-spectrum (100 MHz, $CDCl_3$ with K_2CO_3) of **L9**

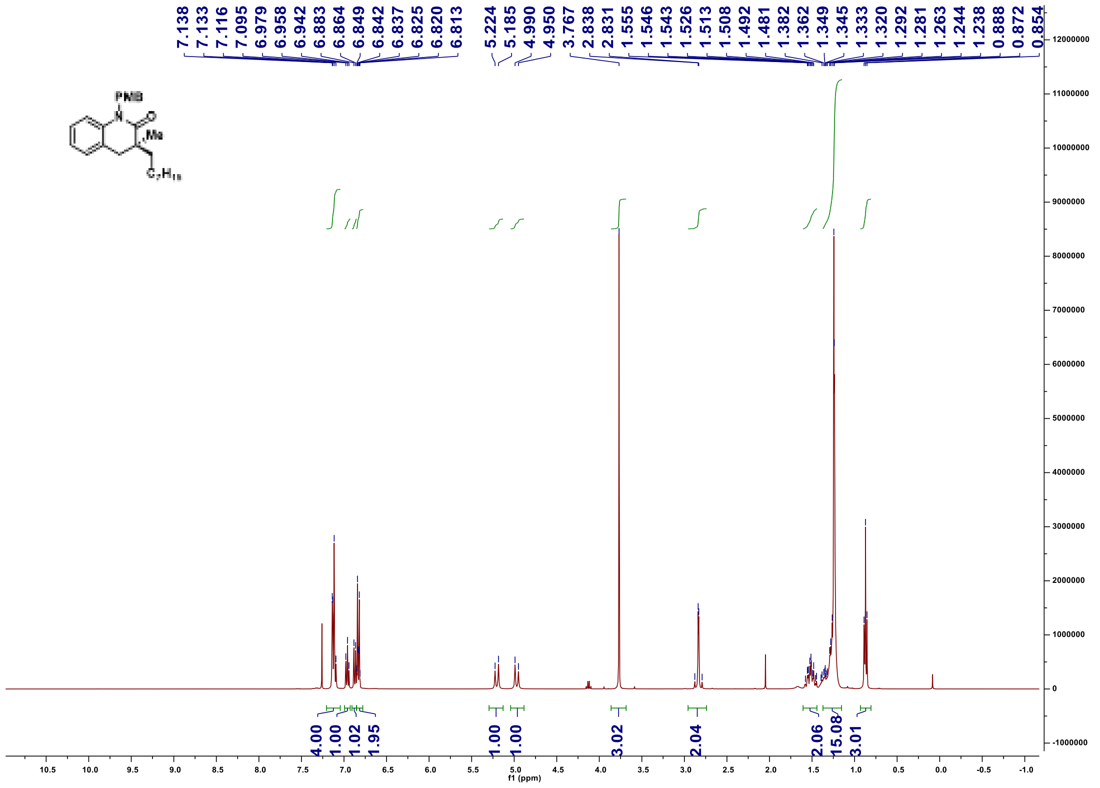

Supplementary Figure 80. ^{1}H NMR-spectrum (400 MHz, $CDCl_3$) of **3a**

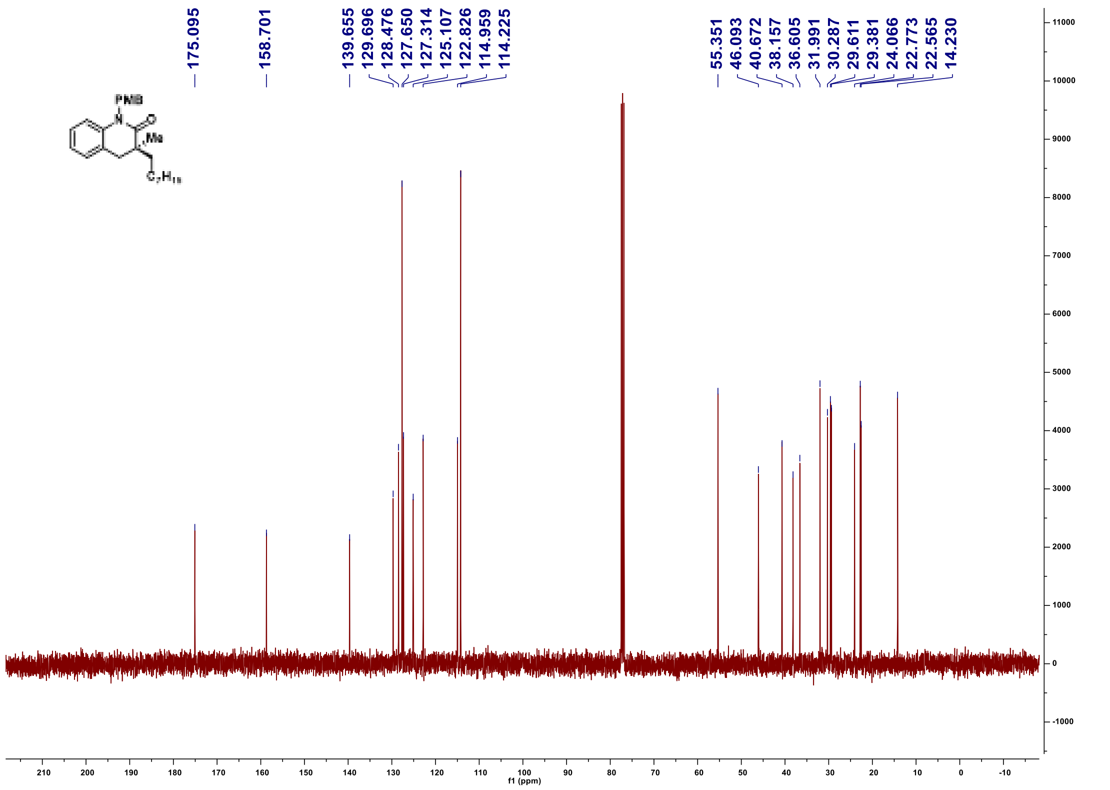

Supplementary Figure 81. ^{13}C NMR-spectrum (100 MHz, $CDCl_3$) of **3a**

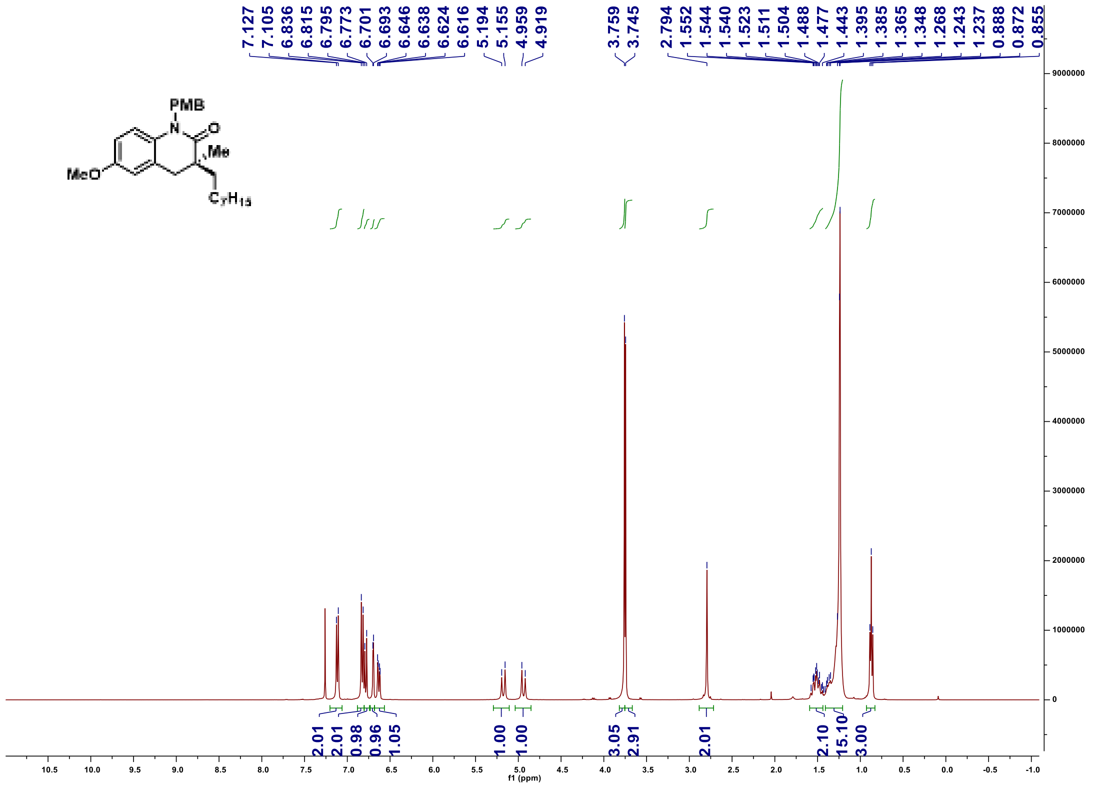

Supplementary Figure 82. ^{1}H NMR-spectrum (400 MHz, $CDCl_3$) of **3b**

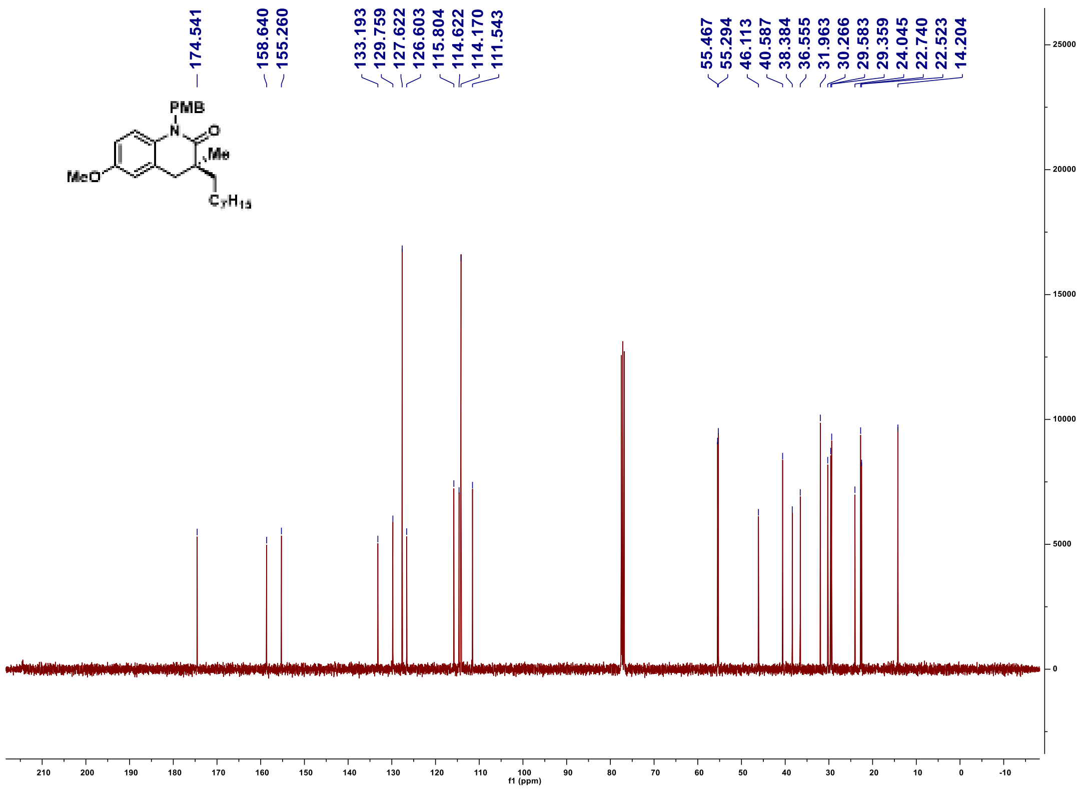

Supplementary Figure 83. ^{13}C NMR-spectrum (100 MHz, $CDCl_3$) of **3b**

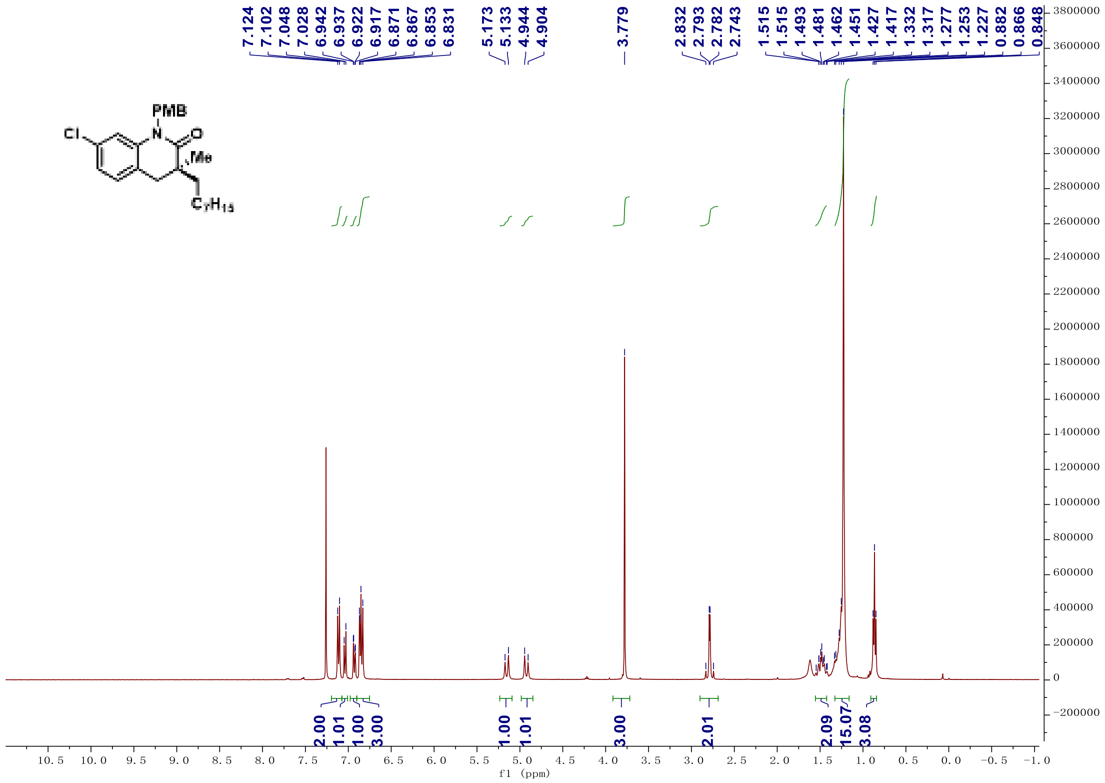

Supplementary Figure 84. ^{1}H NMR-spectrum (400 MHz, $CDCl_3$) of **3c**

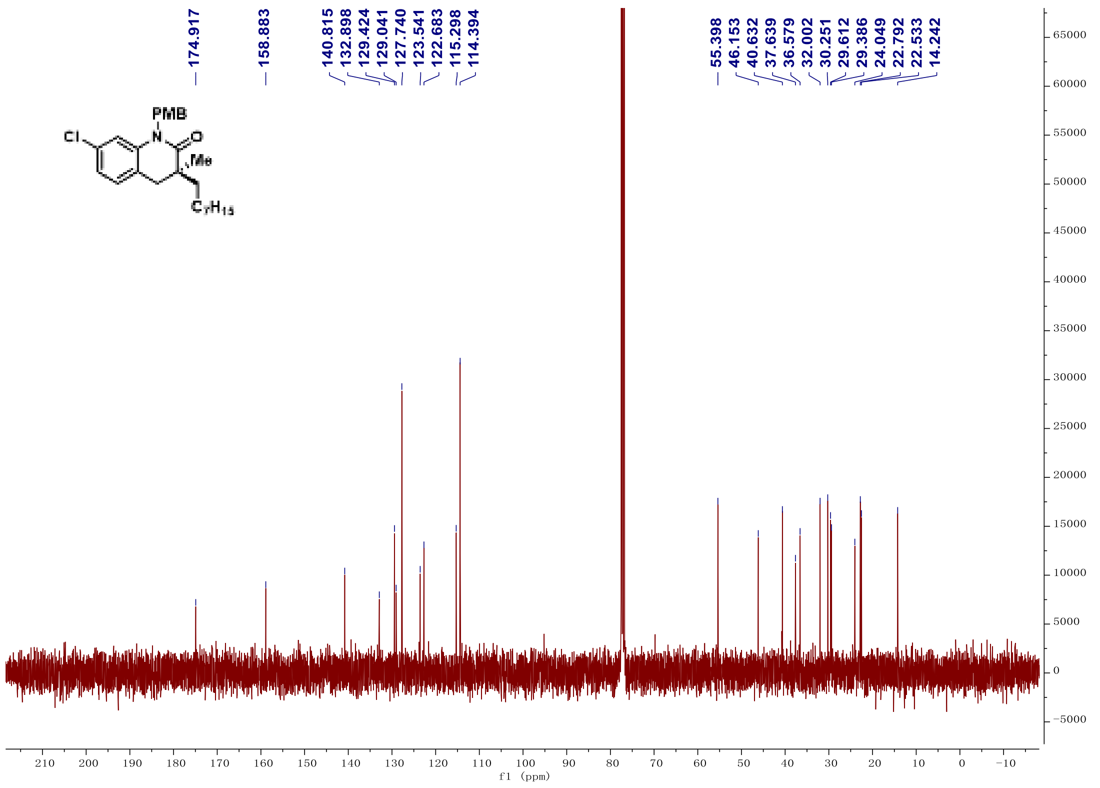

Supplementary Figure 85. ^{13}C NMR-spectrum (100 MHz, $CDCl_3$) of **3c**

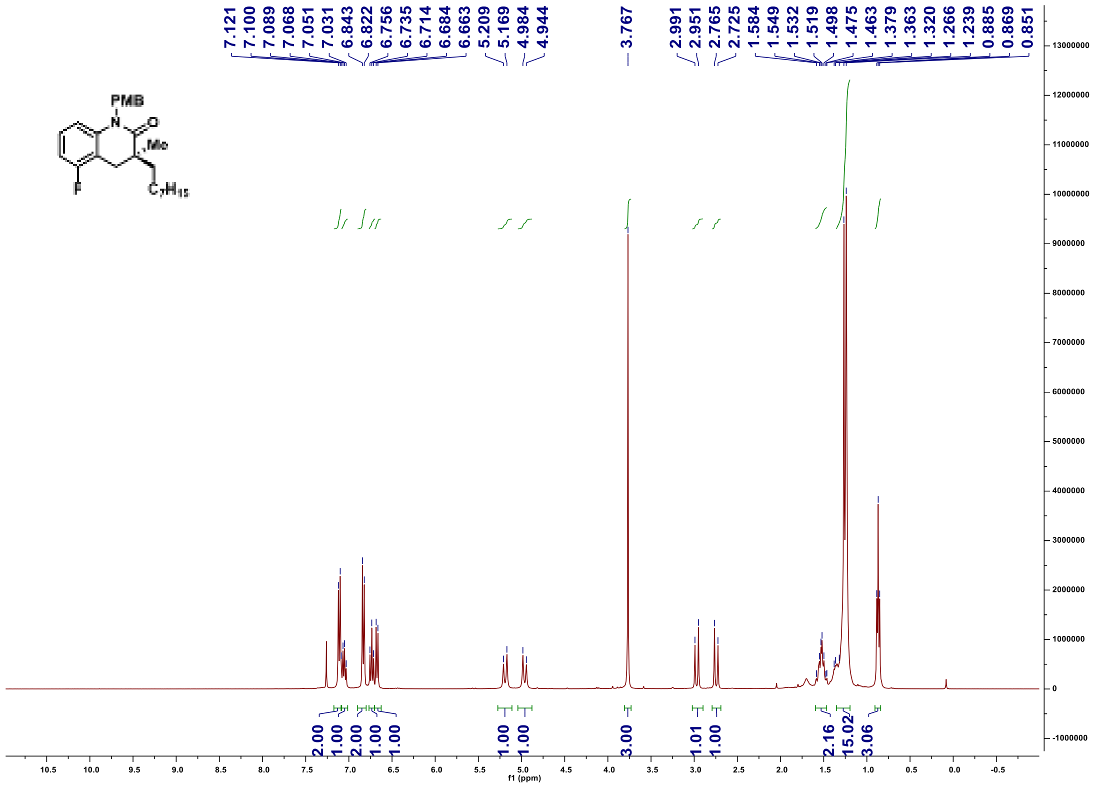

Supplementary Figure 86. ^{1}H NMR-spectrum (400 MHz, $CDCl_3$) of **3d**

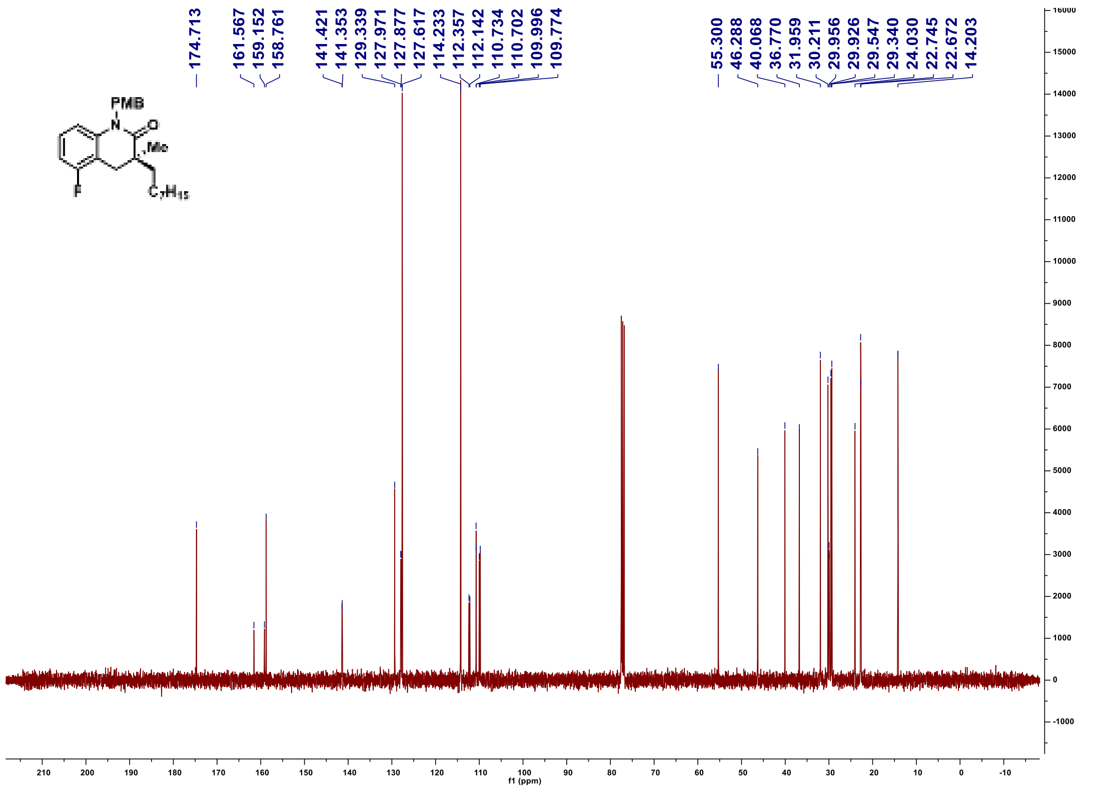

Supplementary Figure 87. ^{13}C NMR-spectrum (100 MHz, $CDCl_3$) of **3d**

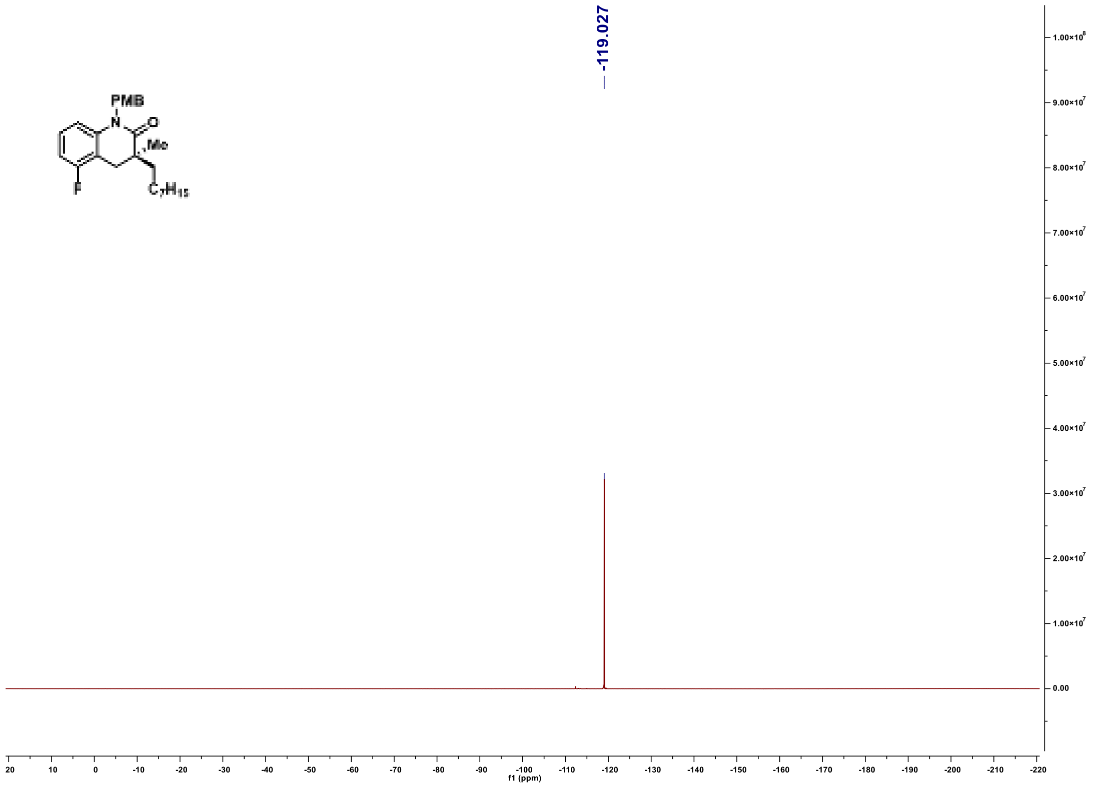

Supplementary Figure 88. ^{19}F NMR-spectrum (376 MHz, $CDCl_3$) of **3d**

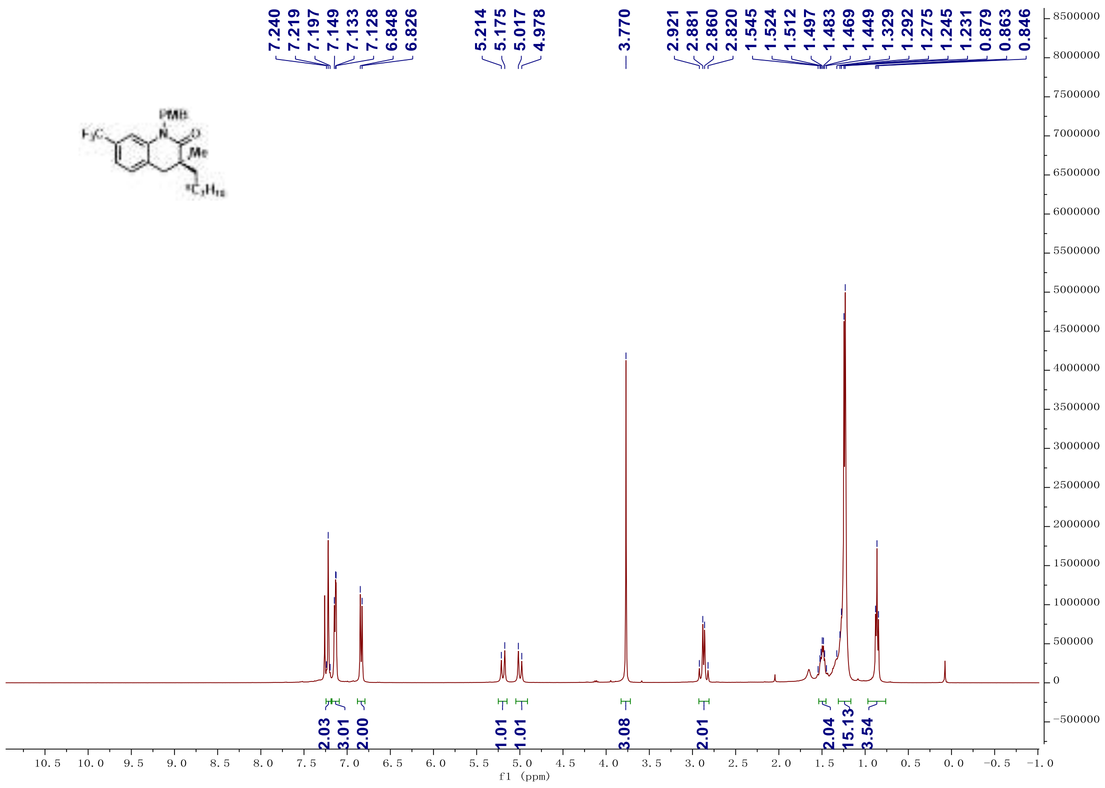

Supplementary Figure 89. ^{1}H NMR-spectrum (400 MHz, $CDCl_3$) of **3e**

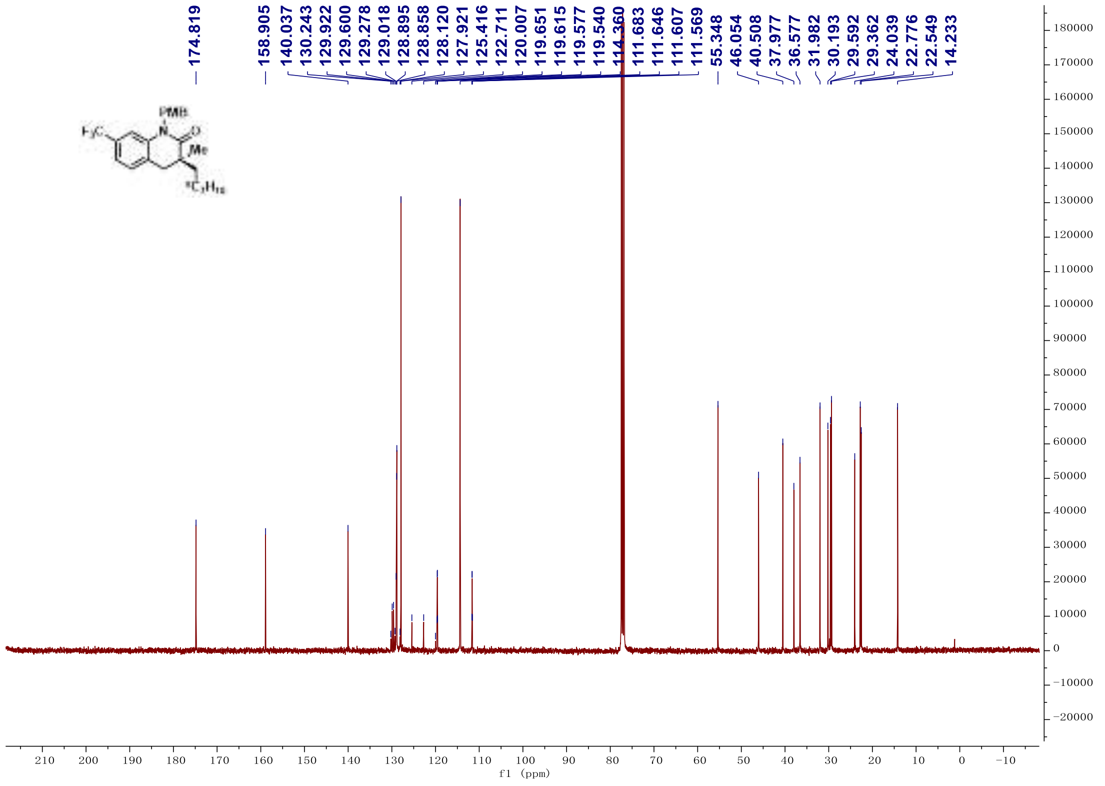

Supplementary Figure 90. ^{13}C NMR-spectrum (100 MHz, $CDCl_3$) of **3e**

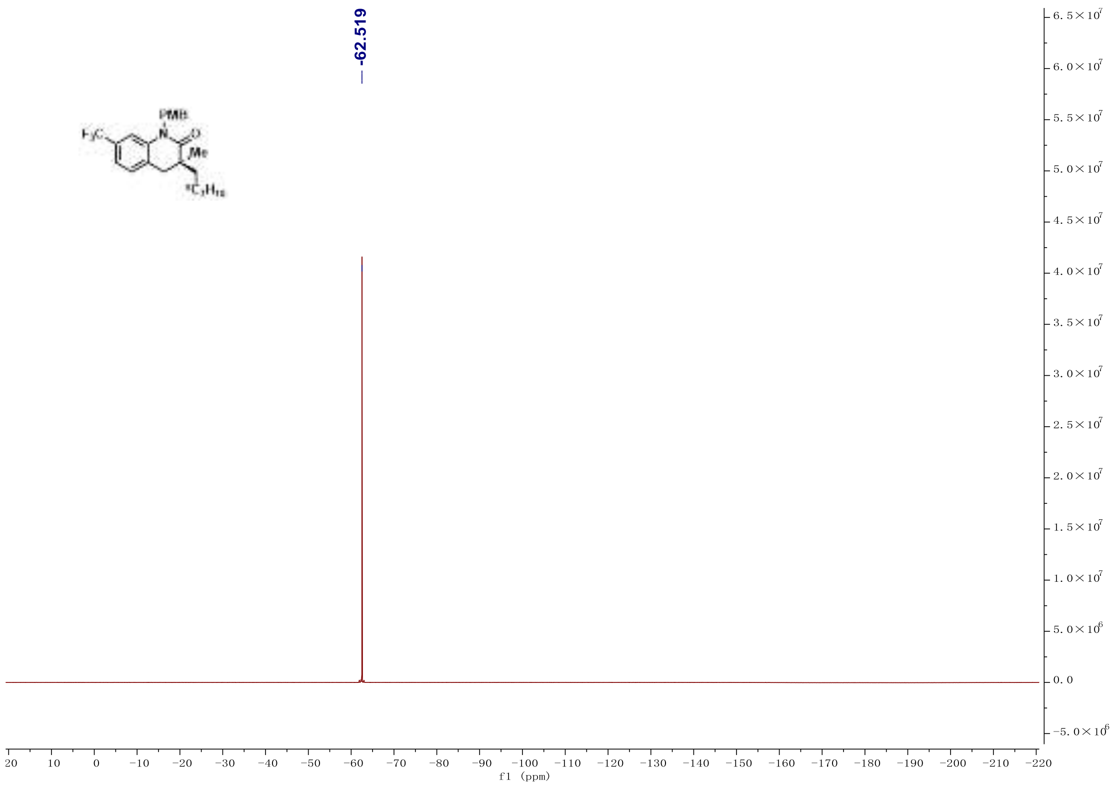

Supplementary Figure 91. ^{19}F NMR-spectrum (376 MHz, $CDCl_3$) of **3e**

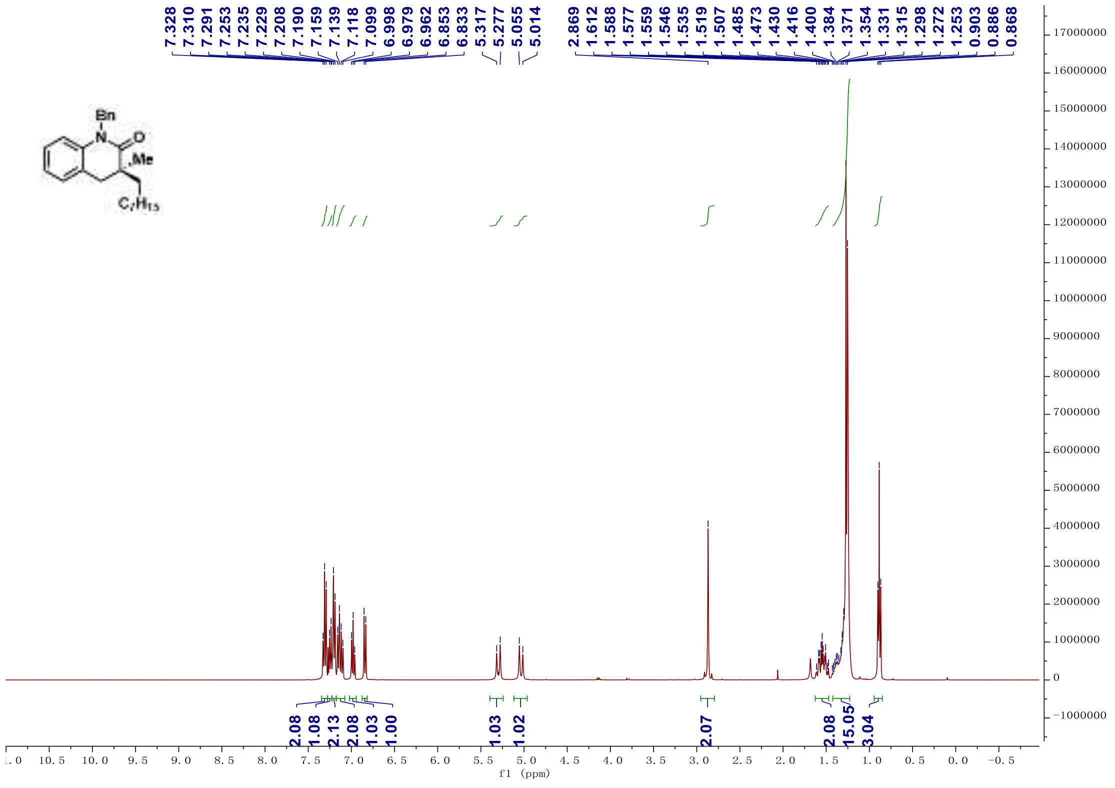

Supplementary Figure 92. ^{1}H NMR-spectrum (400 MHz, $CDCl_3$) of **3f**

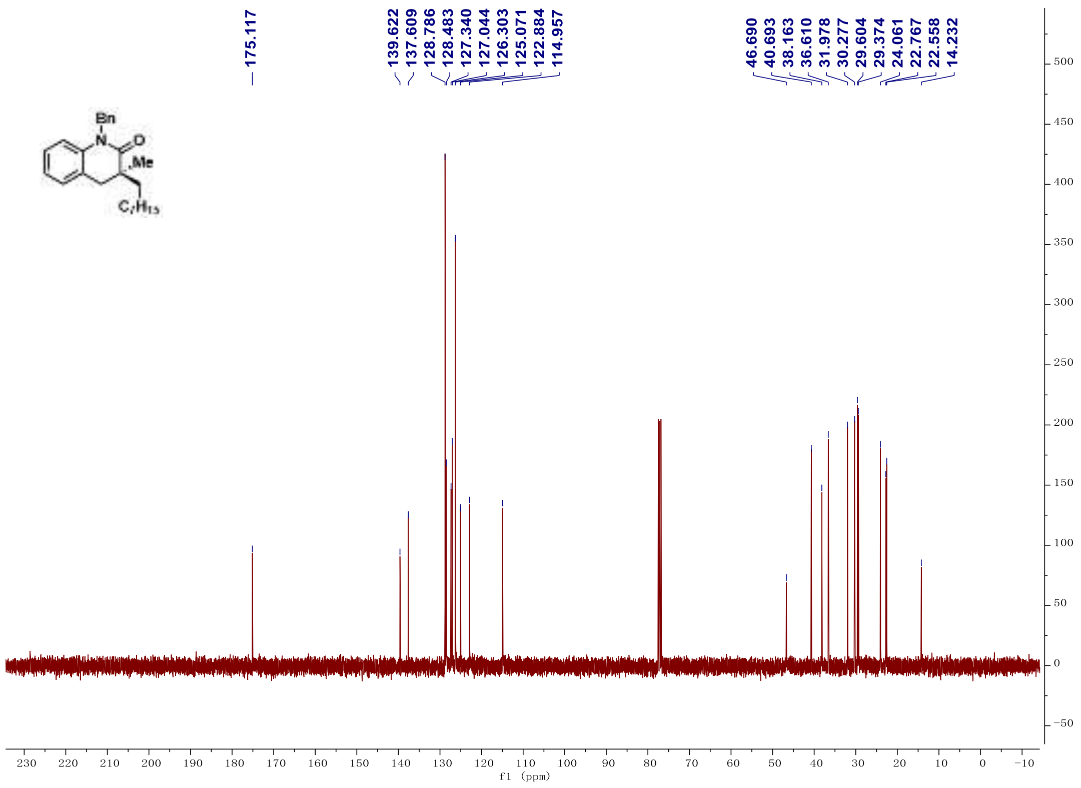

Supplementary Figure 93. ^{13}C NMR-spectrum (100 MHz, $CDCl_3$) of **3f**

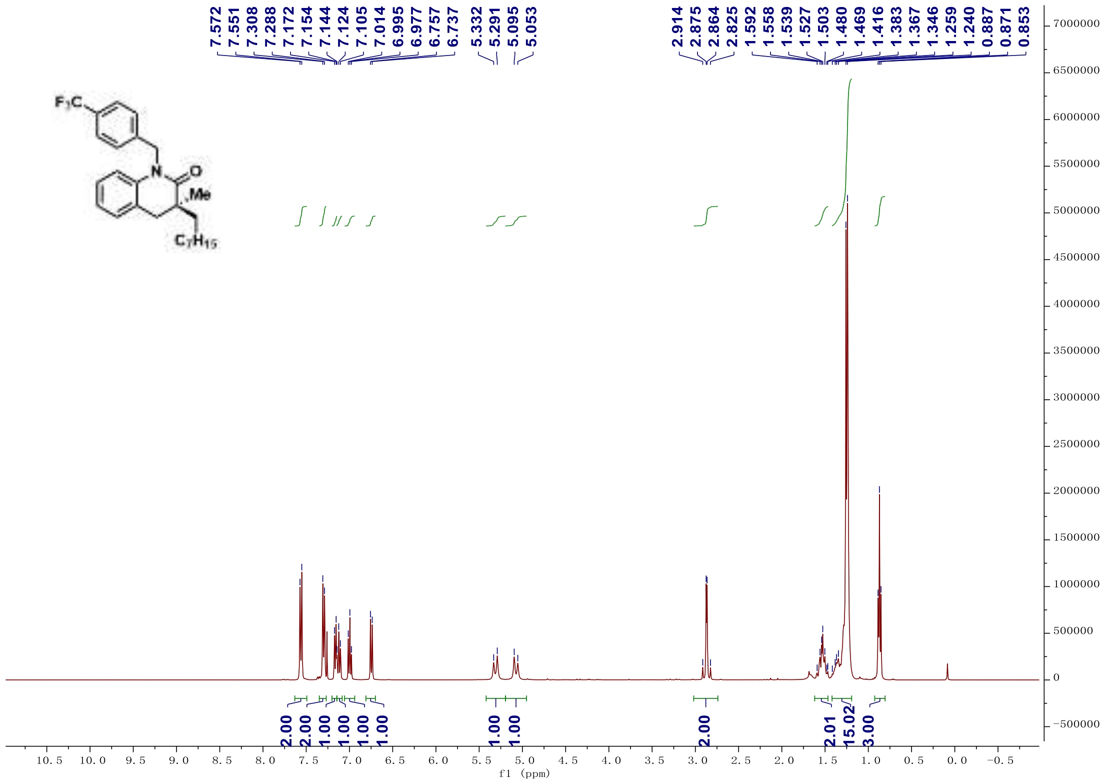

Supplementary Figure 94. ^{1}H NMR-spectrum (400 MHz, $CDCl_3$) of **3g**

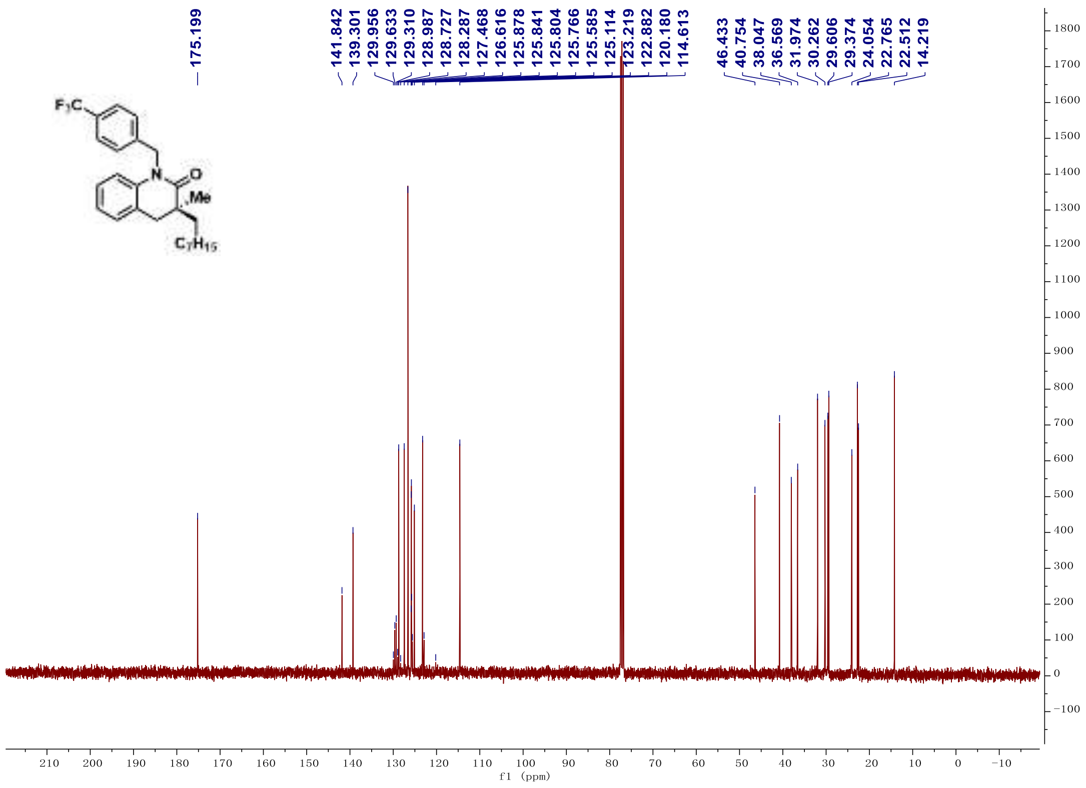

Supplementary Figure 95. ^{13}C NMR-spectrum (100 MHz, $CDCl_3$) of **3g**

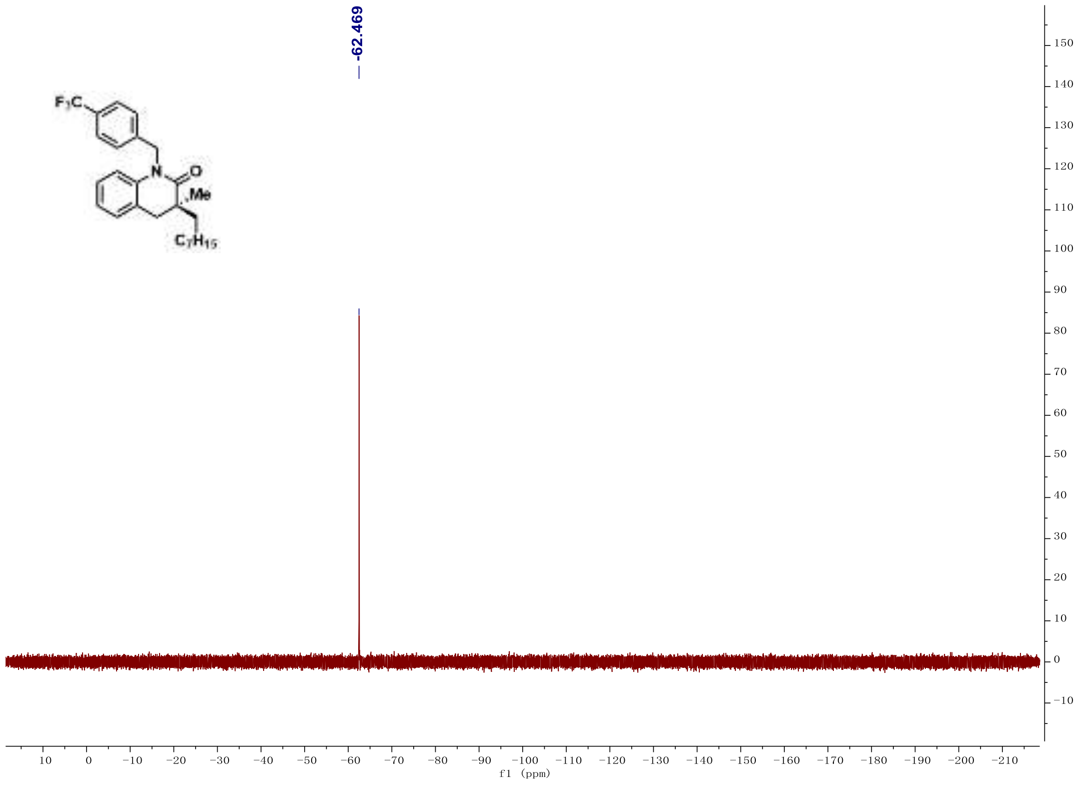

Supplementary Figure 96. ^{19}F NMR-spectrum (376 MHz, $CDCl_3$) of **3g**

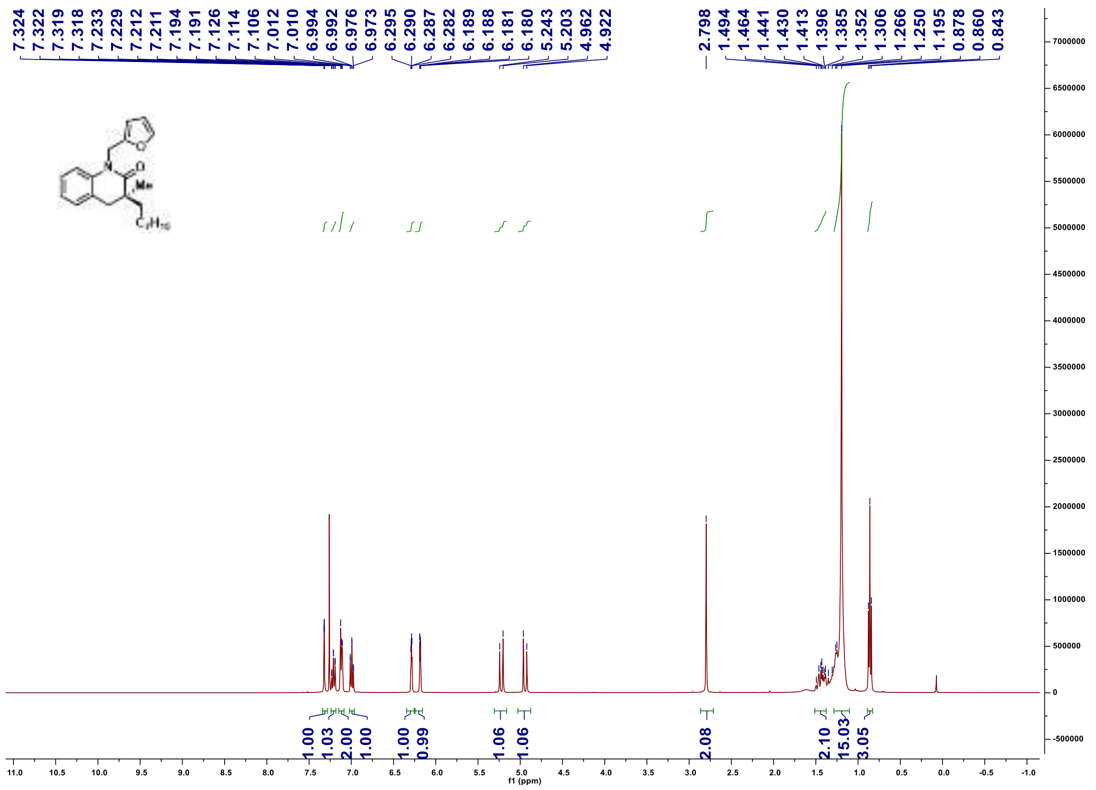

Supplementary Figure 97. ^{1}H NMR-spectrum (400 MHz, $CDCl_3$) of **3h**

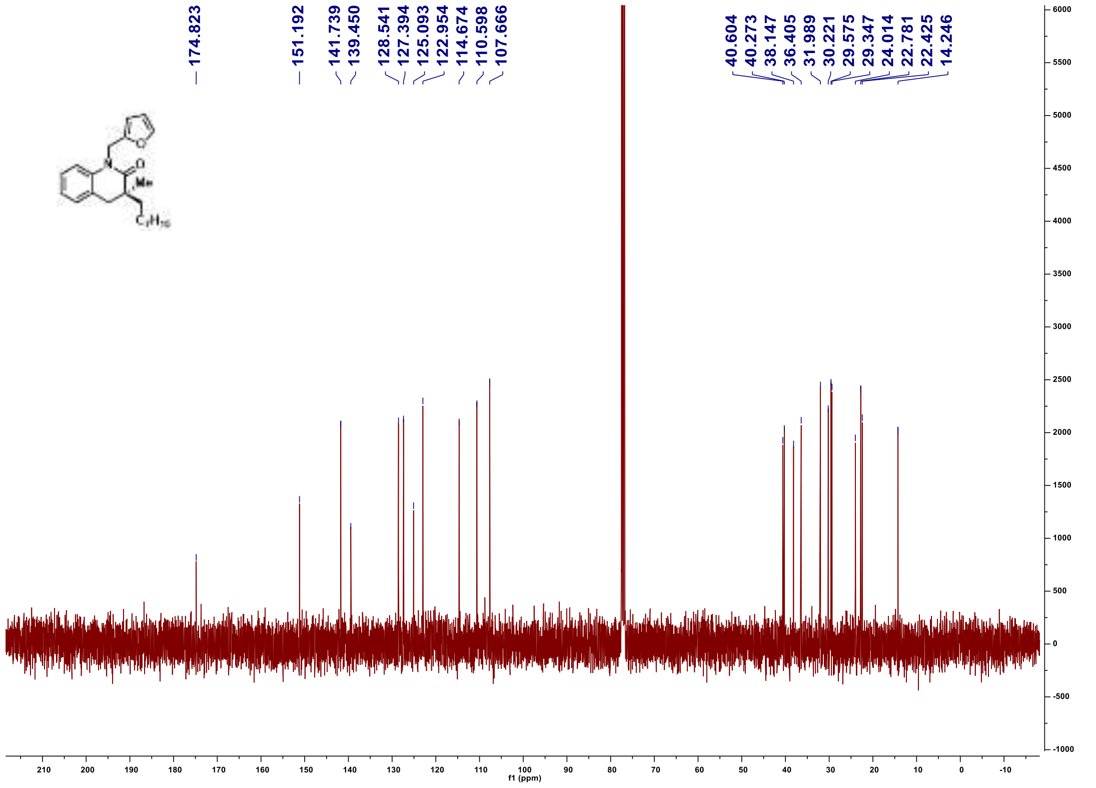

Supplementary Figure 98. ^{13}C NMR-spectrum (100 MHz, $CDCl_3$) of **3h**

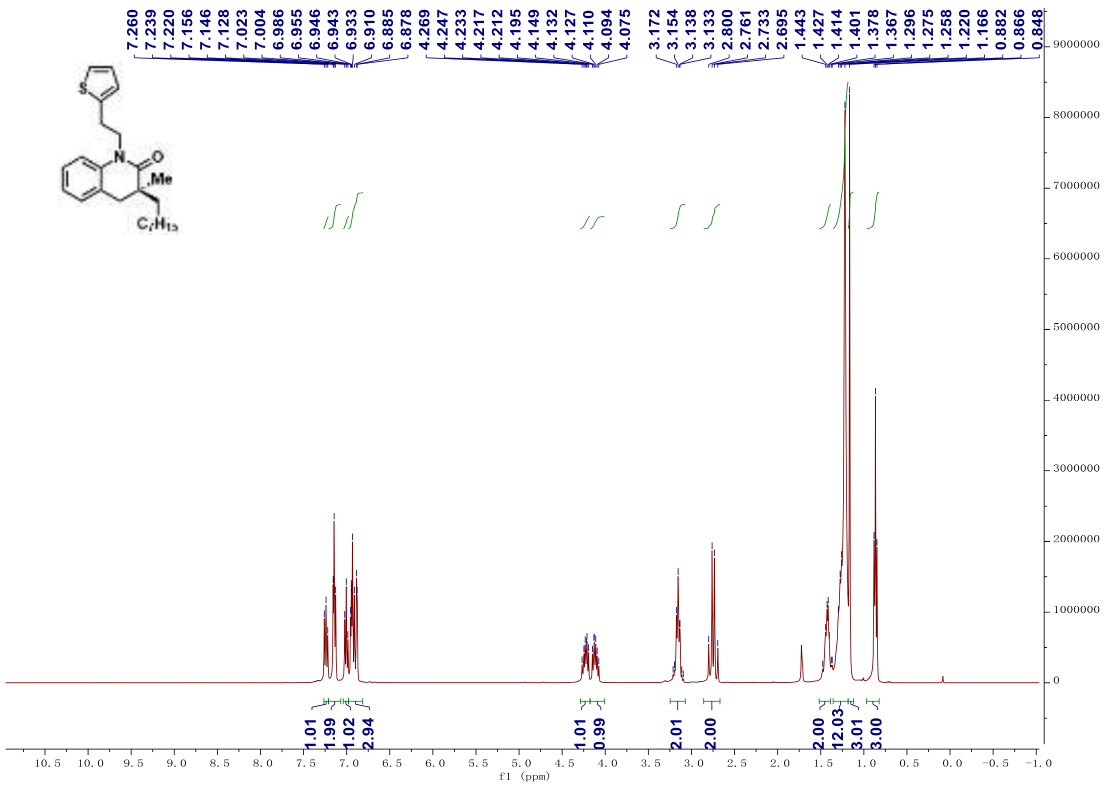

Supplementary Figure 99. ^{1}H NMR-spectrum (400 MHz, $CDCl_3$) of **3i**

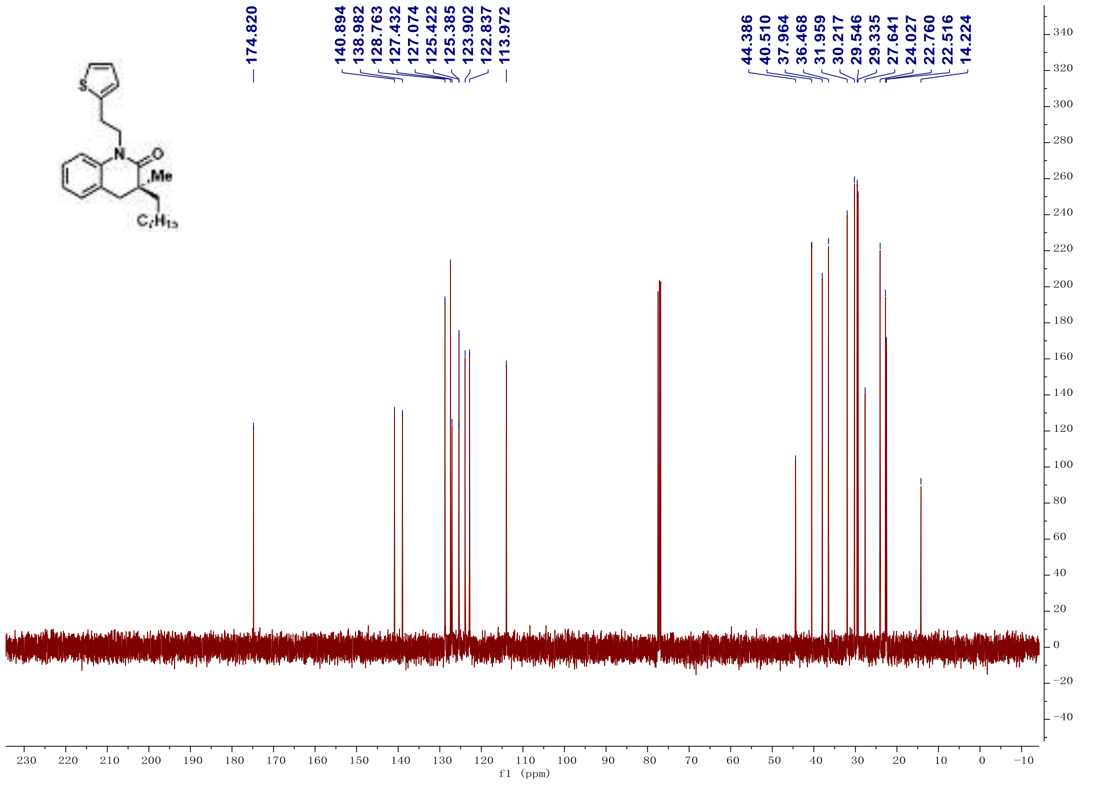

Supplementary Figure 100. ^{13}C NMR-spectrum (100 MHz, $CDCl_3$) of **3i**

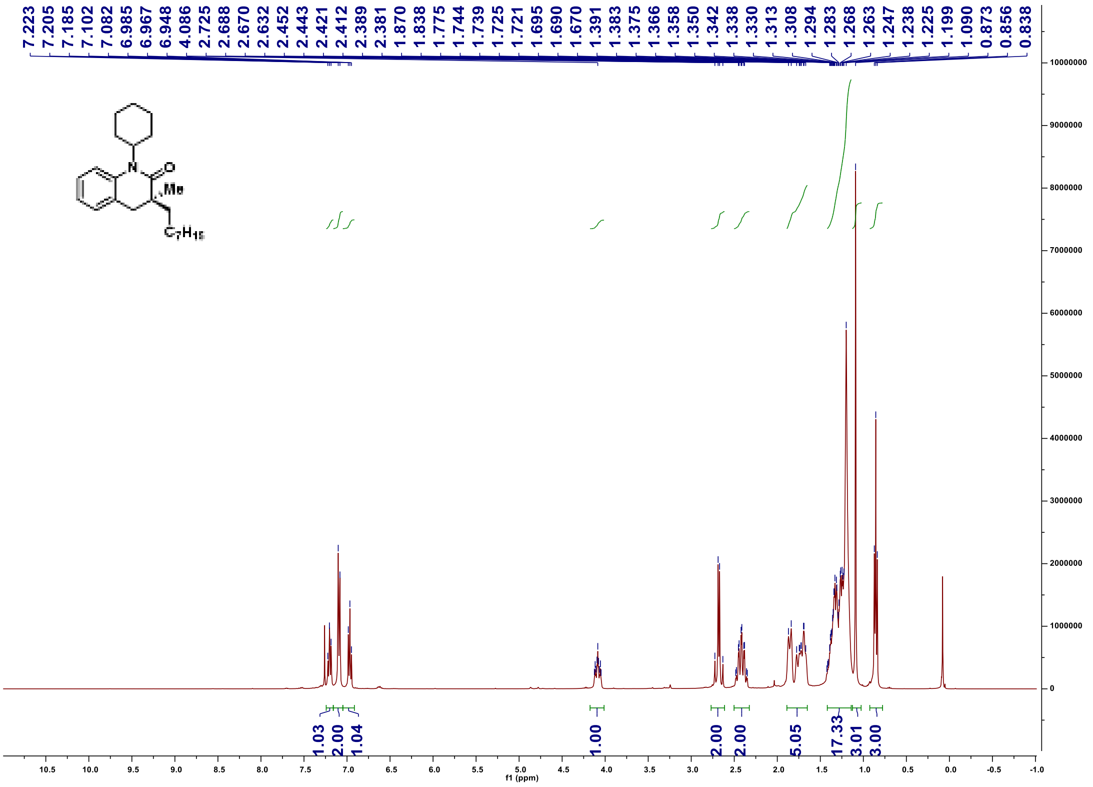

Supplementary Figure 101. ^{1}H NMR-spectrum (400 MHz, $CDCl_3$) of **3j**

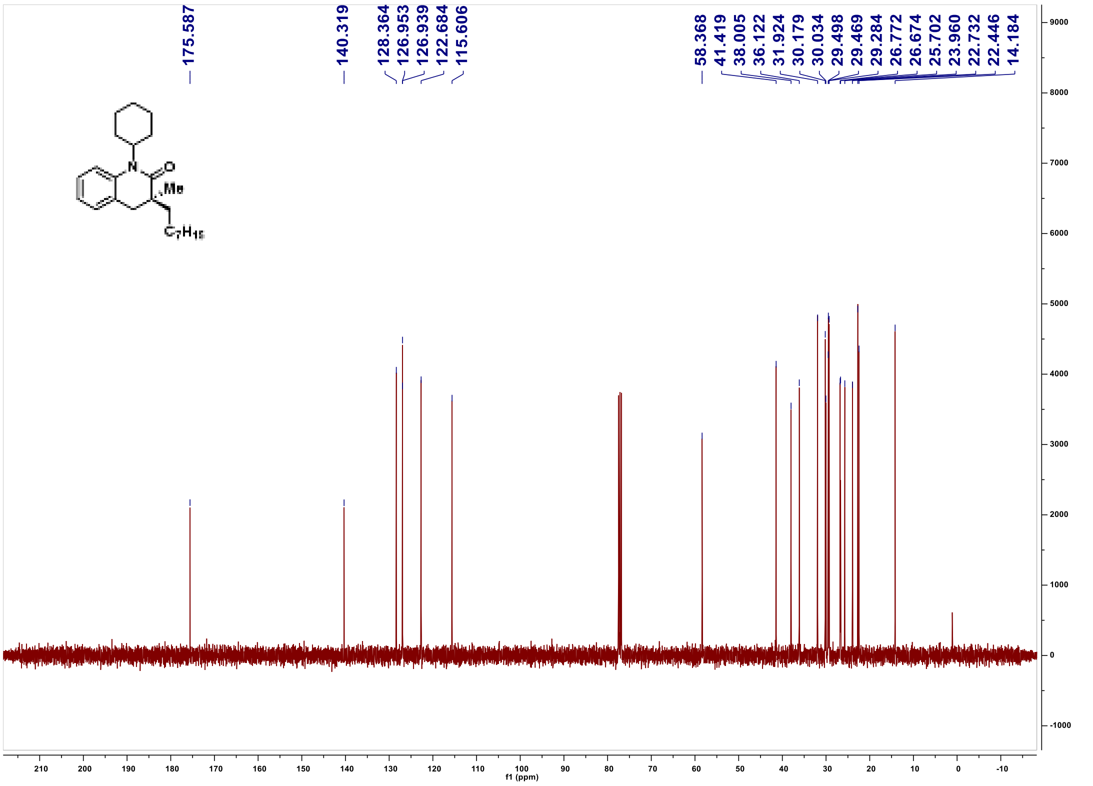

Supplementary Figure 102. ^{13}C NMR-spectrum (100 MHz, $CDCl_3$) of **3j**

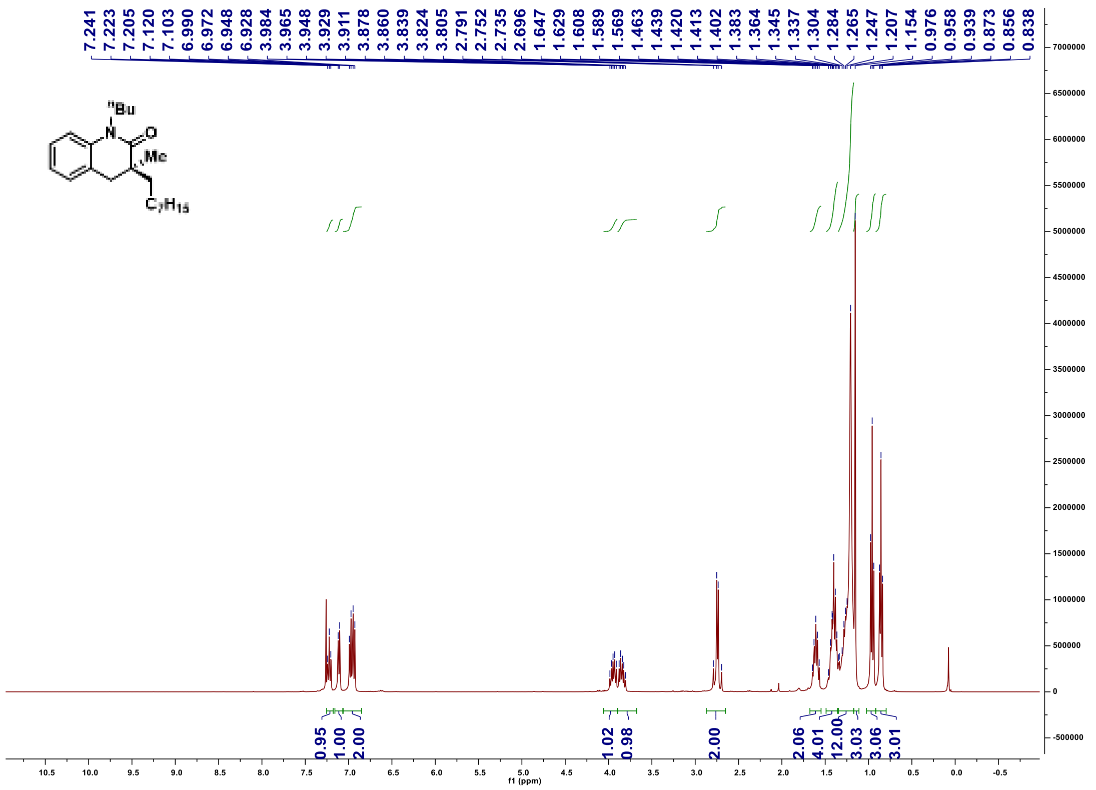

Supplementary Figure 103. ^{1}H NMR-spectrum (400 MHz, $CDCl_3$) of **3k**

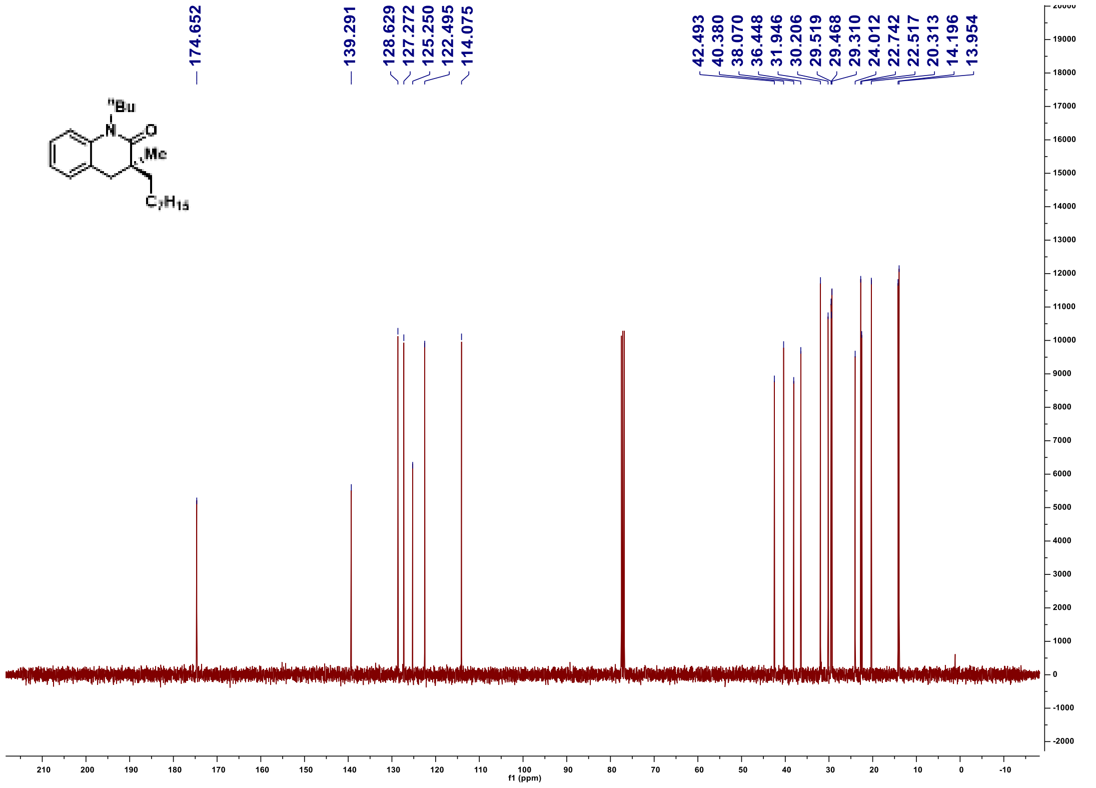

Supplementary Figure 104. ^{13}C NMR-spectrum (100 MHz, $CDCl_3$) of **3k**

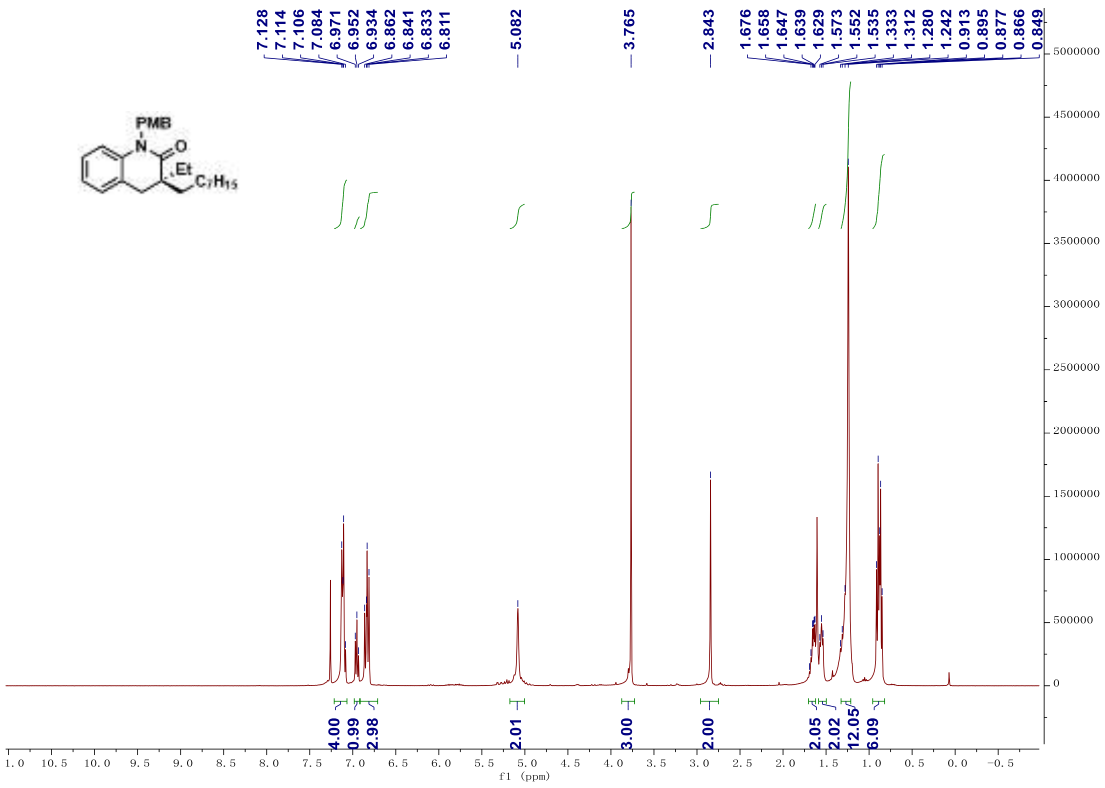

Supplementary Figure 105. ^{1}H NMR-spectrum (400 MHz, $CDCl_3$) of **3l**

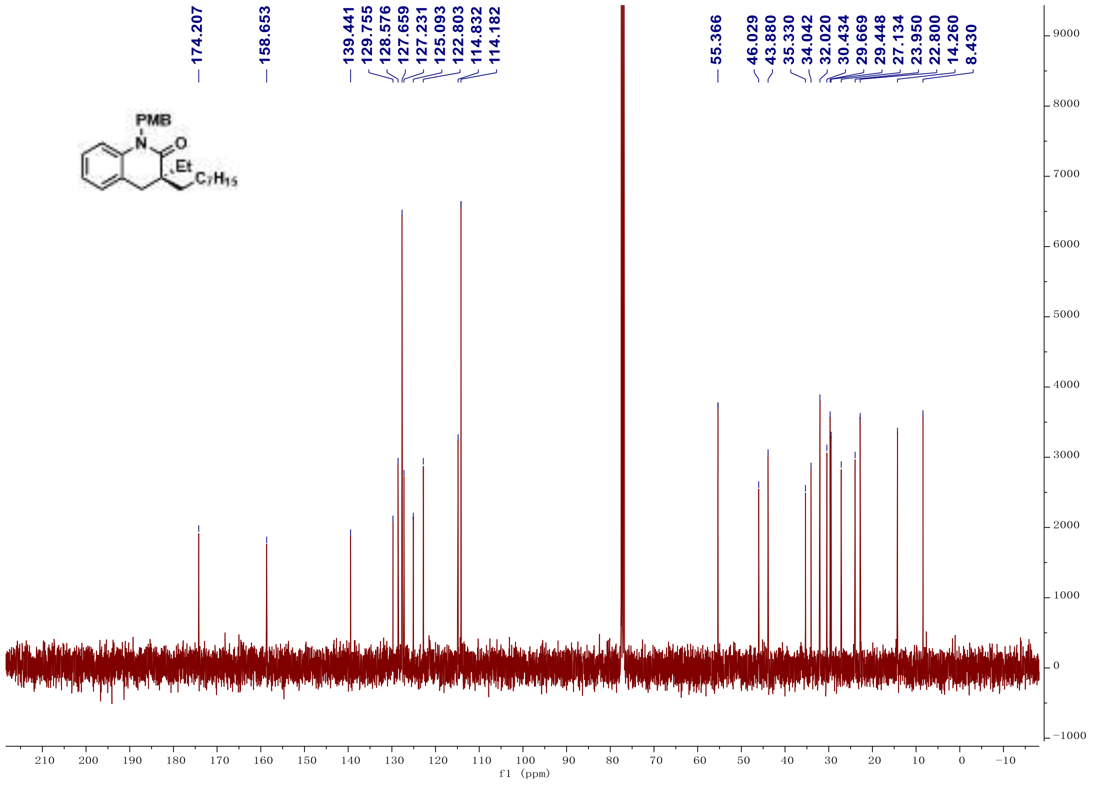

Supplementary Figure 106. ^{13}C NMR-spectrum (100 MHz, $CDCl_3$) of **3l**

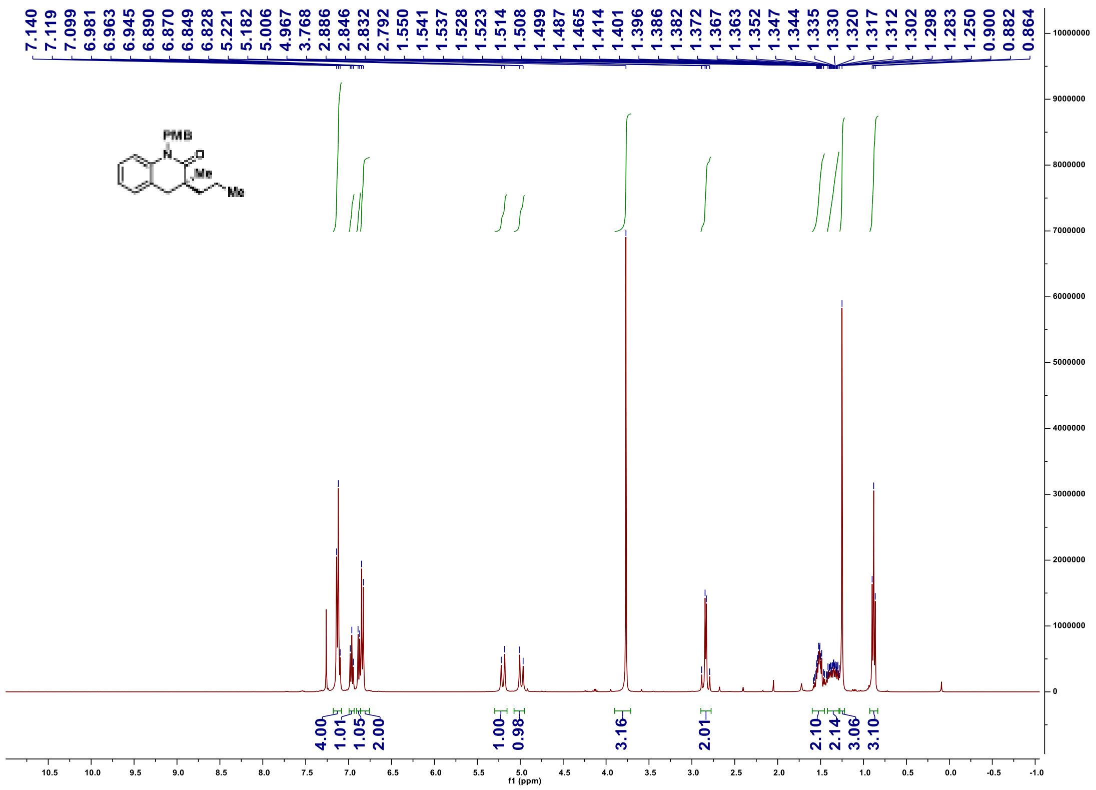

Supplementary Figure 107. ^{1}H NMR-spectrum (400 MHz, $CDCl_3$) of **3n**

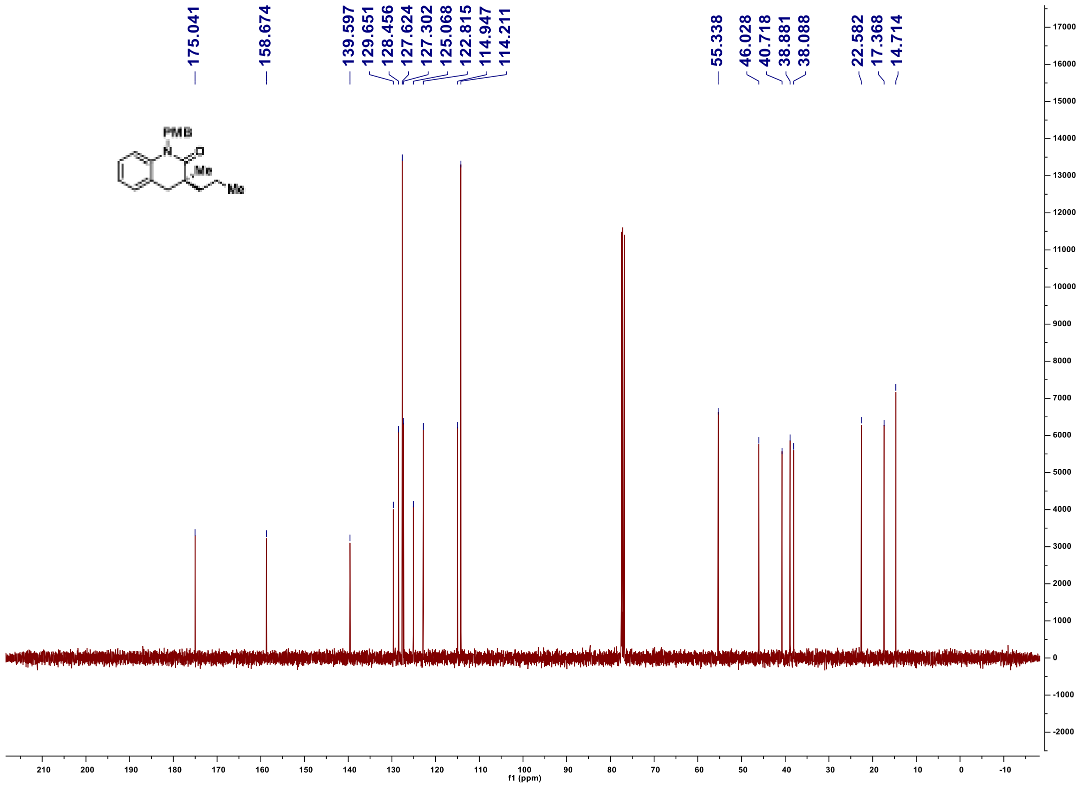

Supplementary Figure 108. ^{13}C NMR-spectrum (100 MHz, $CDCl_3$) of **3n**

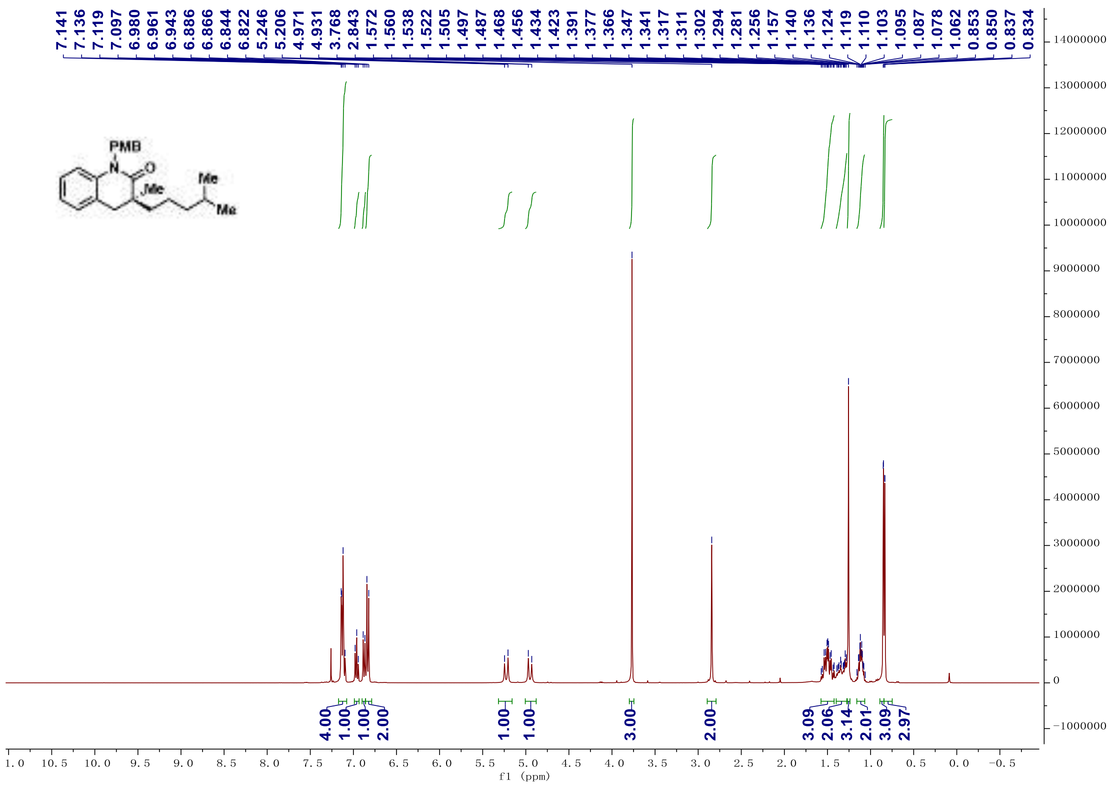

Supplementary Figure 109. ^{1}H NMR-spectrum (400 MHz, $CDCl_3$) of **3o**

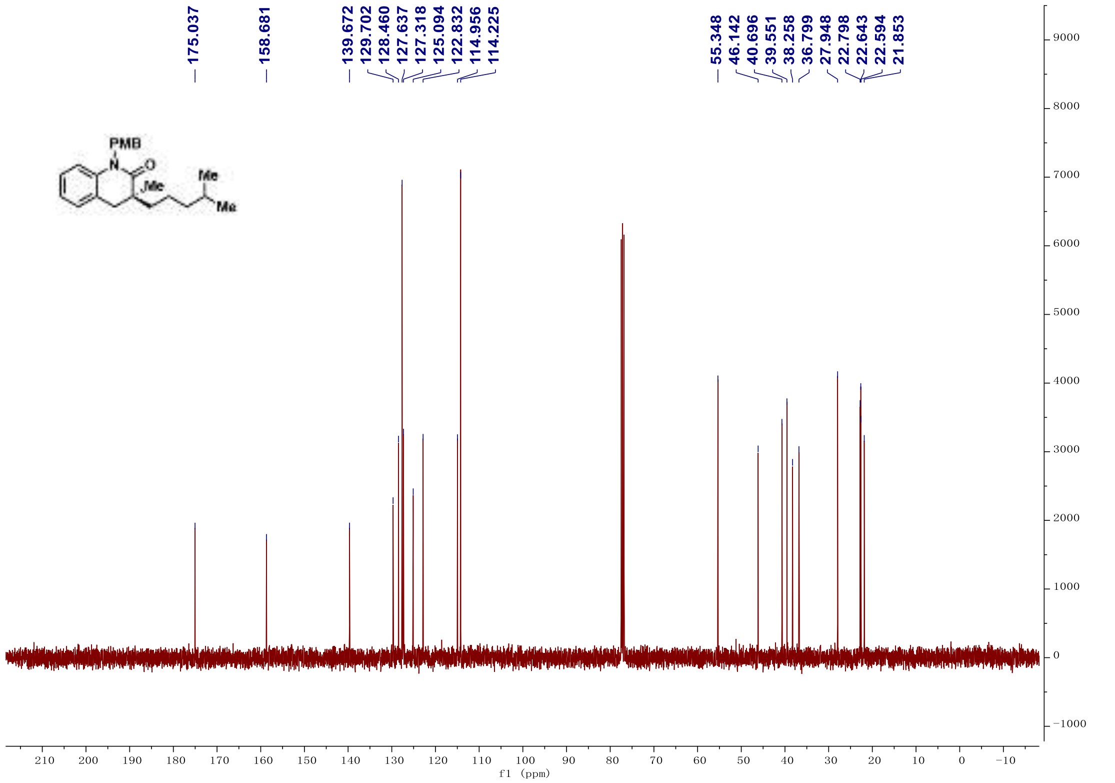

Supplementary Figure 110. ^{13}C NMR-spectrum (100 MHz, $CDCl_3$) of **3o**

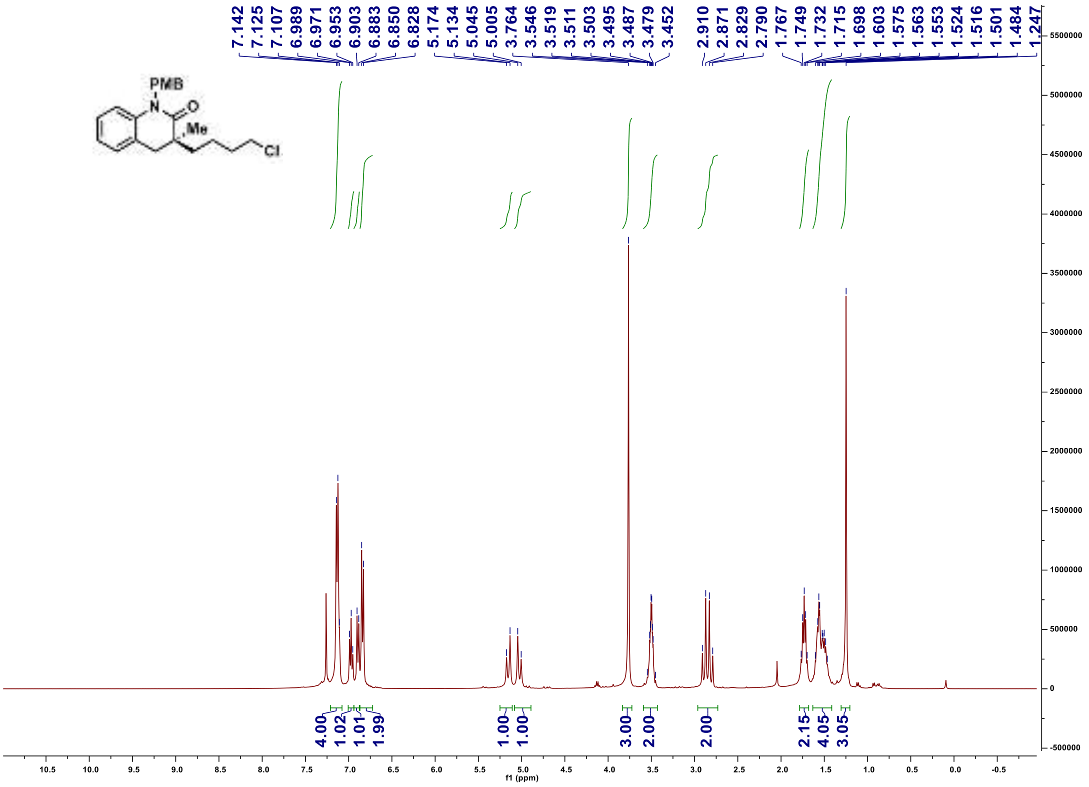

Supplementary Figure 111. ^{1}H NMR-spectrum (400 MHz, $CDCl_3$) of **3p**

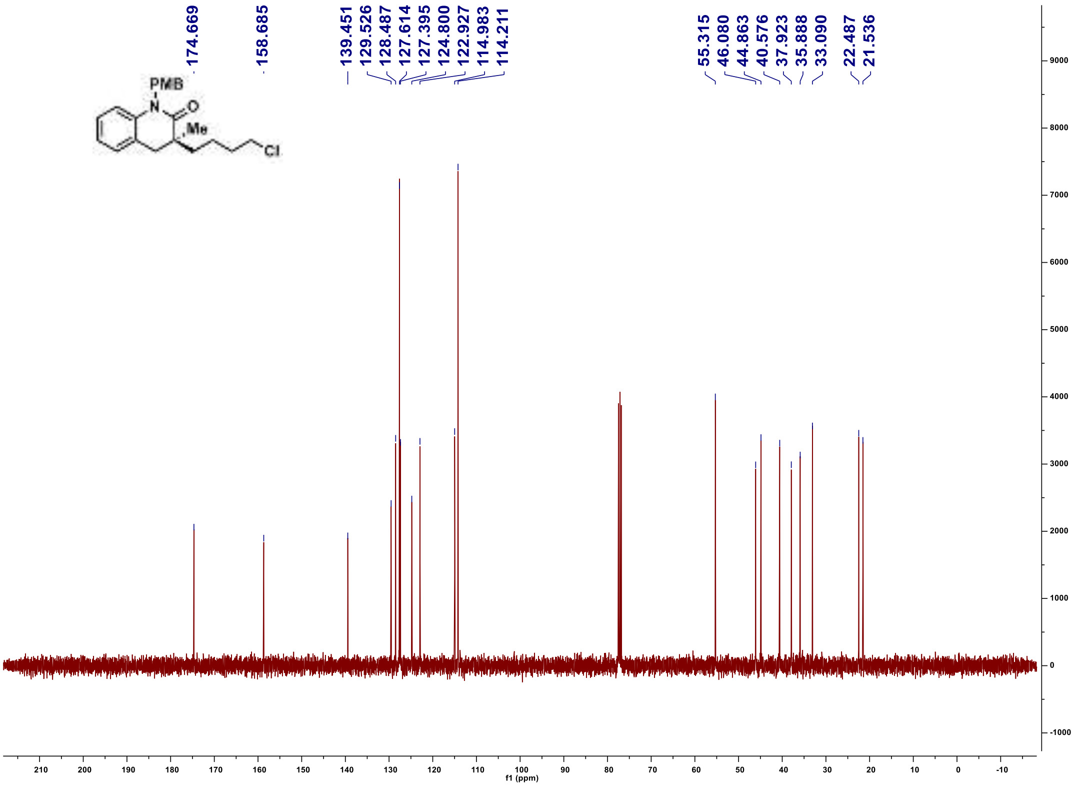

Supplementary Figure 112. ^{13}C NMR-spectrum (100 MHz, $CDCl_3$) of **3p**

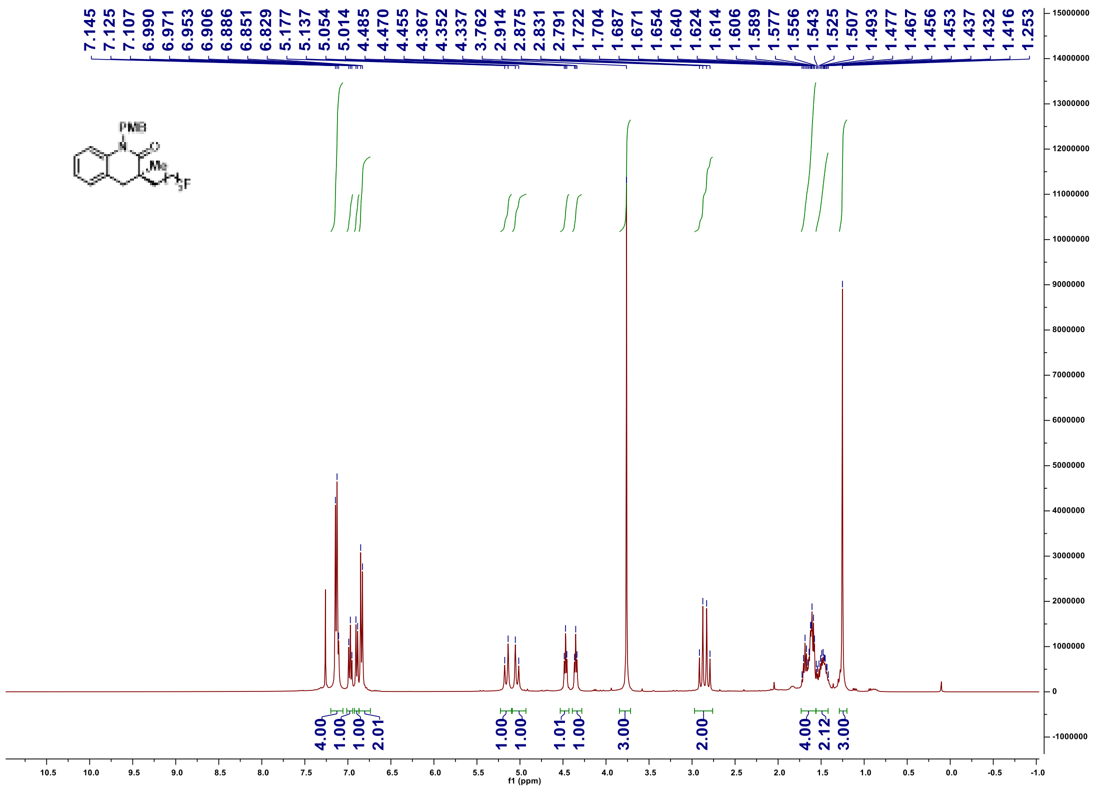

Supplementary Figure 113. ^{1}H NMR-spectrum (400 MHz, $CDCl_3$) of **3q**

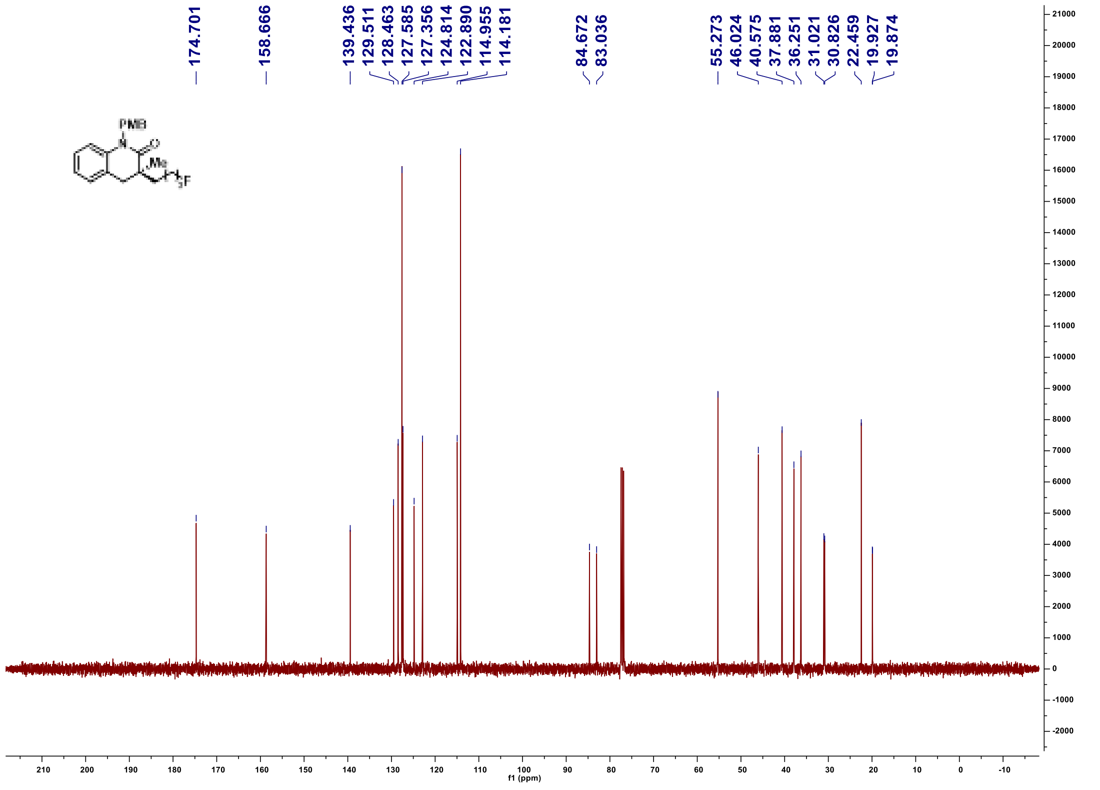

Supplementary Figure 114. ^{13}C NMR-spectrum (100 MHz, $CDCl_3$) of **3q**

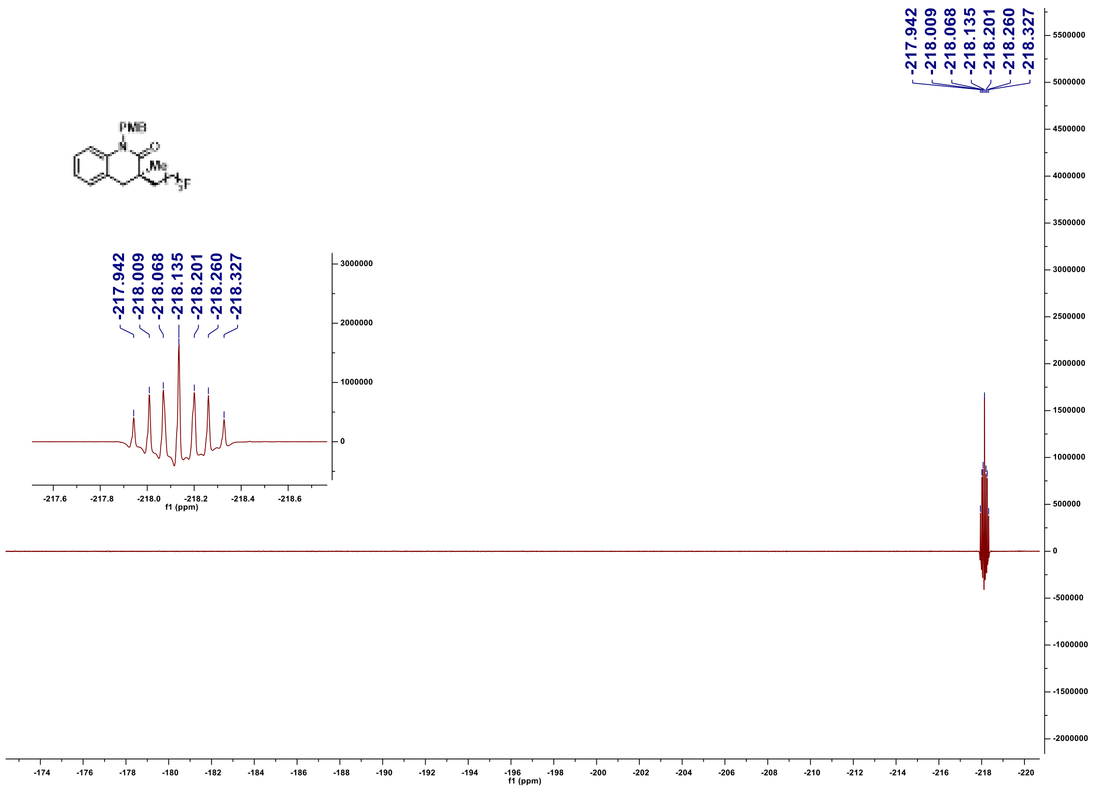

Supplementary Figure 115. ^{19}F NMR-spectrum (376 MHz, $CDCl_3$) of **3q**

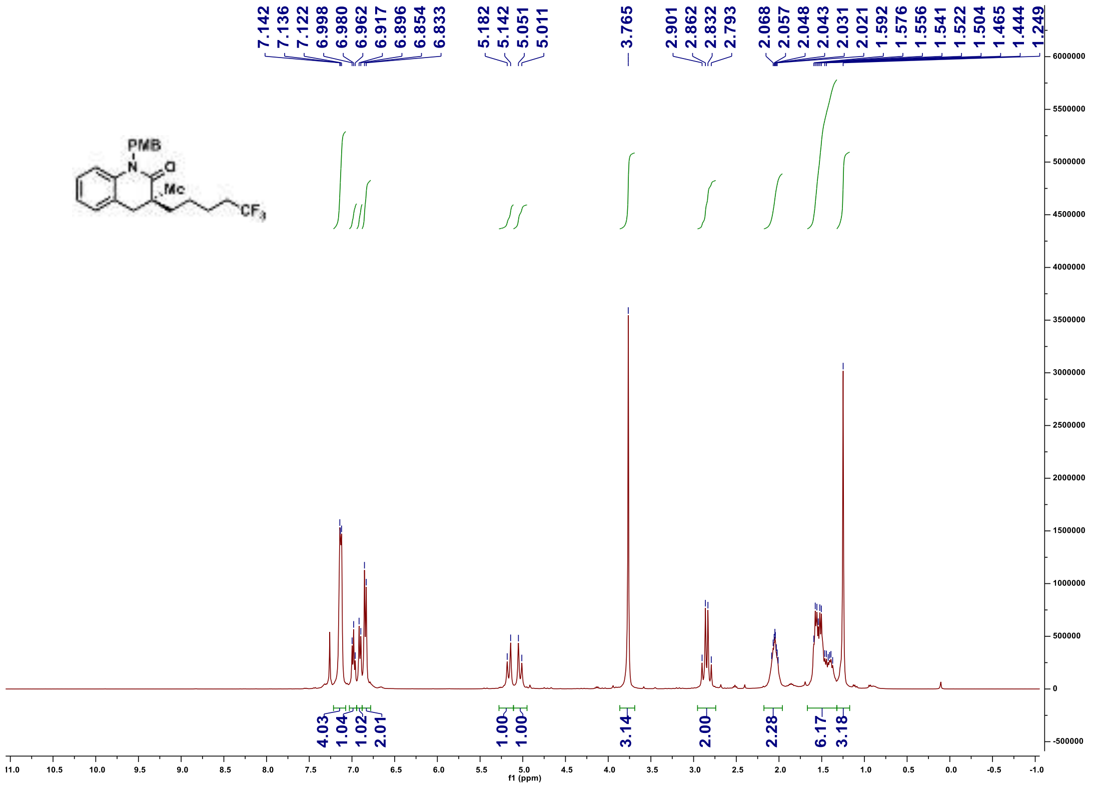

Supplementary Figure 116. ^{1}H NMR-spectrum (400 MHz, $CDCl_3$) of **3r**

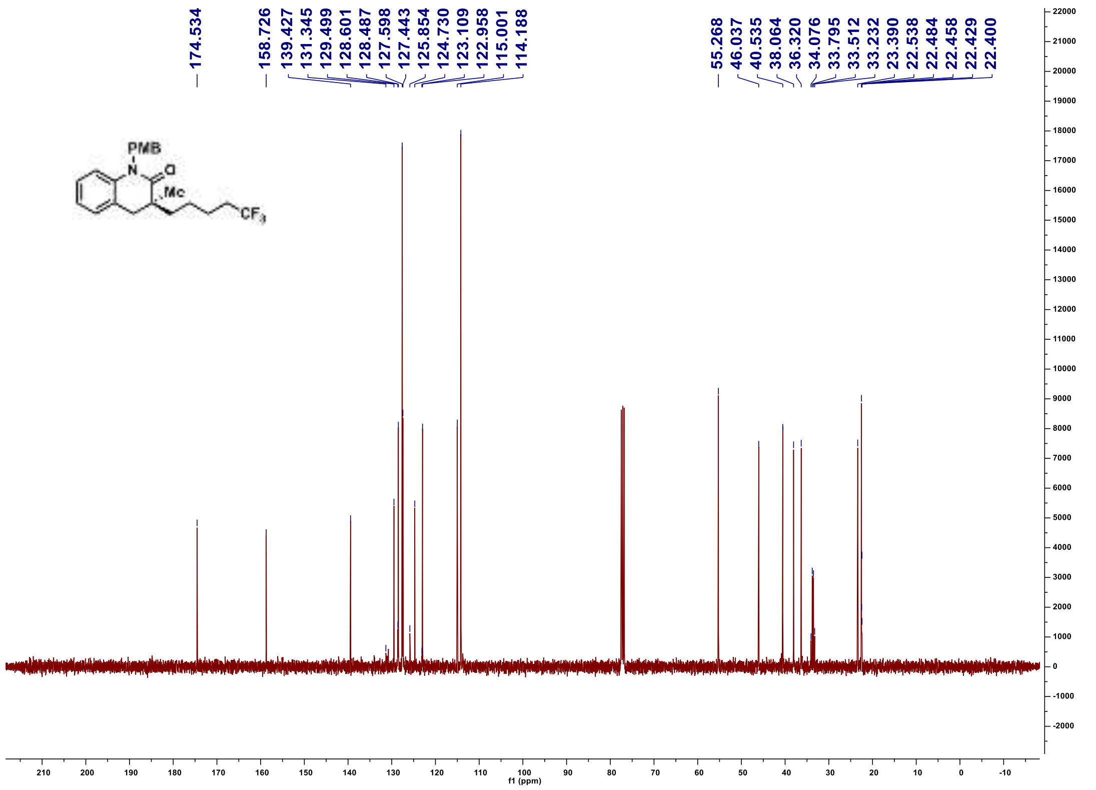

Supplementary Figure 117. ^{13}C NMR-spectrum (100 MHz, $CDCl_3$) of **3r**

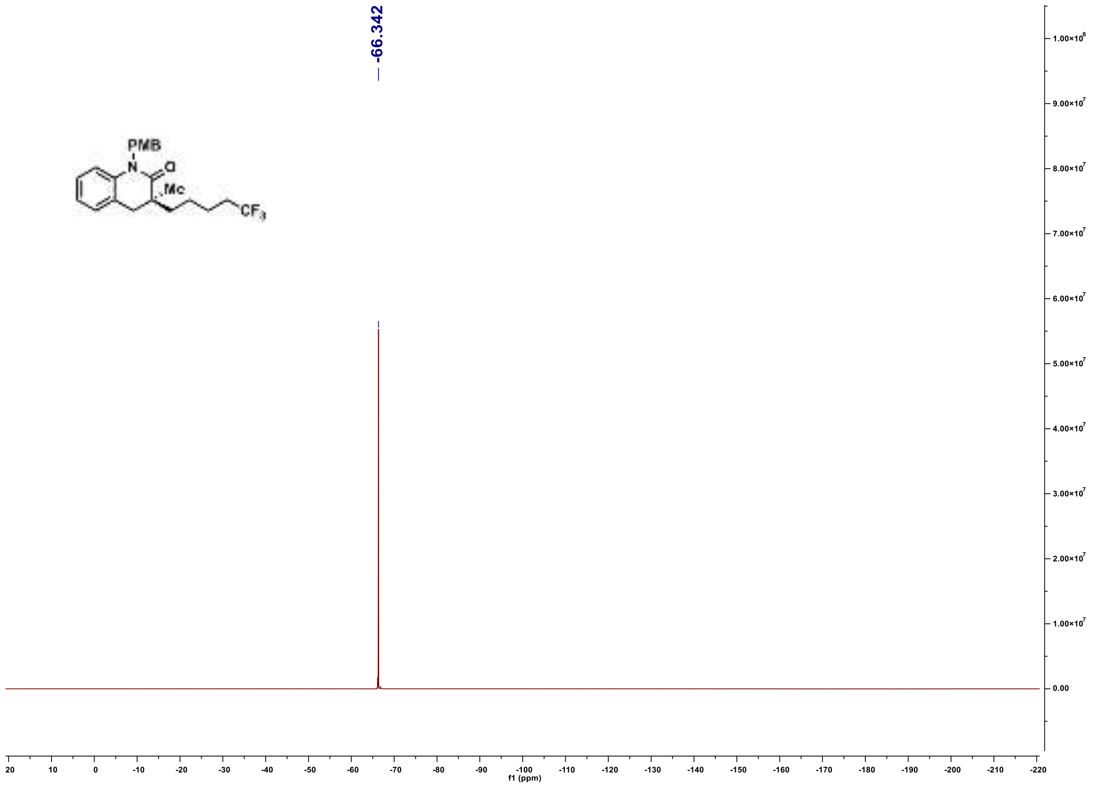

Supplementary Figure 118. ^{19}F NMR-spectrum (376 MHz, $CDCl_3$) of **3r**

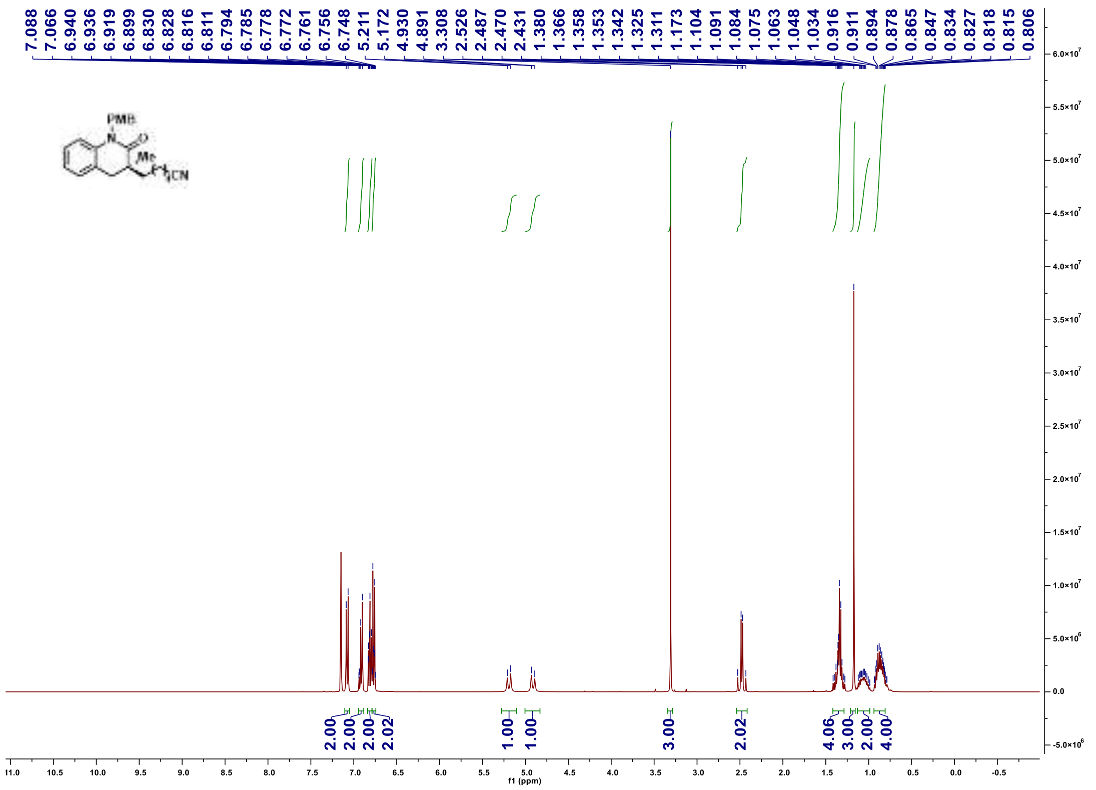

Supplementary Figure 119. ^{1}H NMR-spectrum (400 MHz, C_6D_6) of **3s**

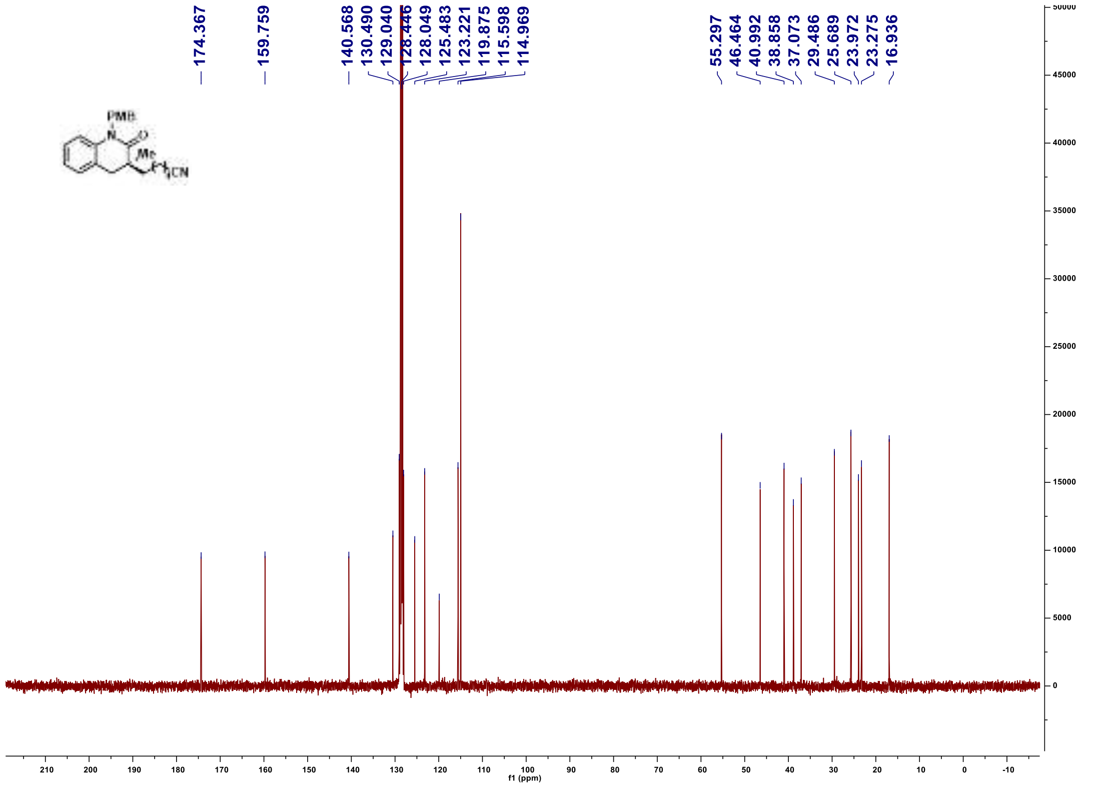

Supplementary Figure 120. ^{13}C NMR-spectrum (100 MHz, C_6D_6) of **3s**

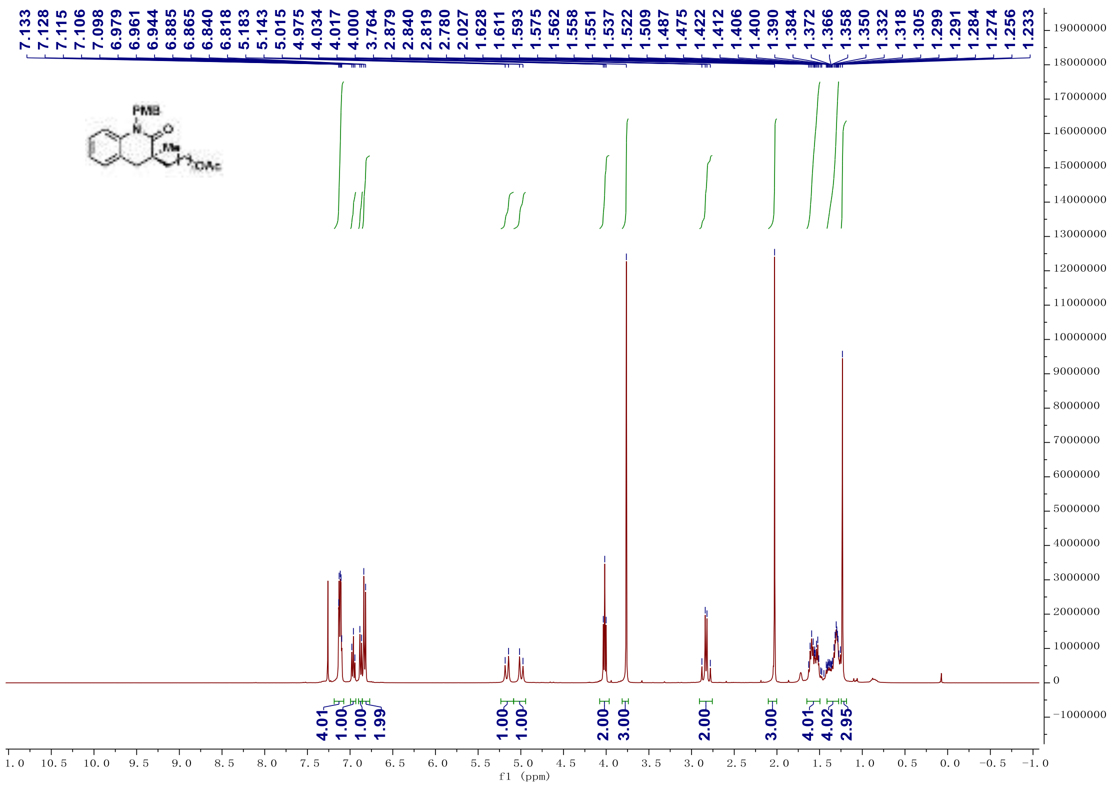

Supplementary Figure 121. ^{1}H NMR-spectrum (400 MHz, $CDCl_3$) of **3t**

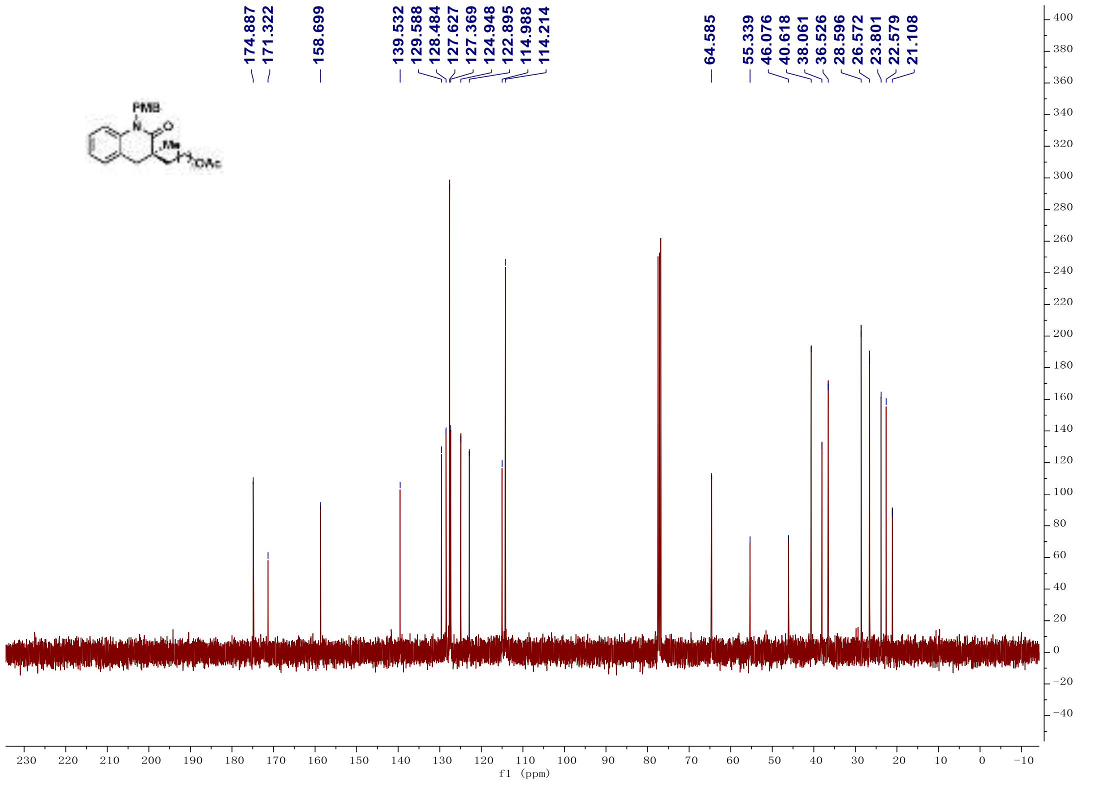

Supplementary Figure 122. ^{13}C NMR-spectrum (100 MHz, $CDCl_3$) of **3t**

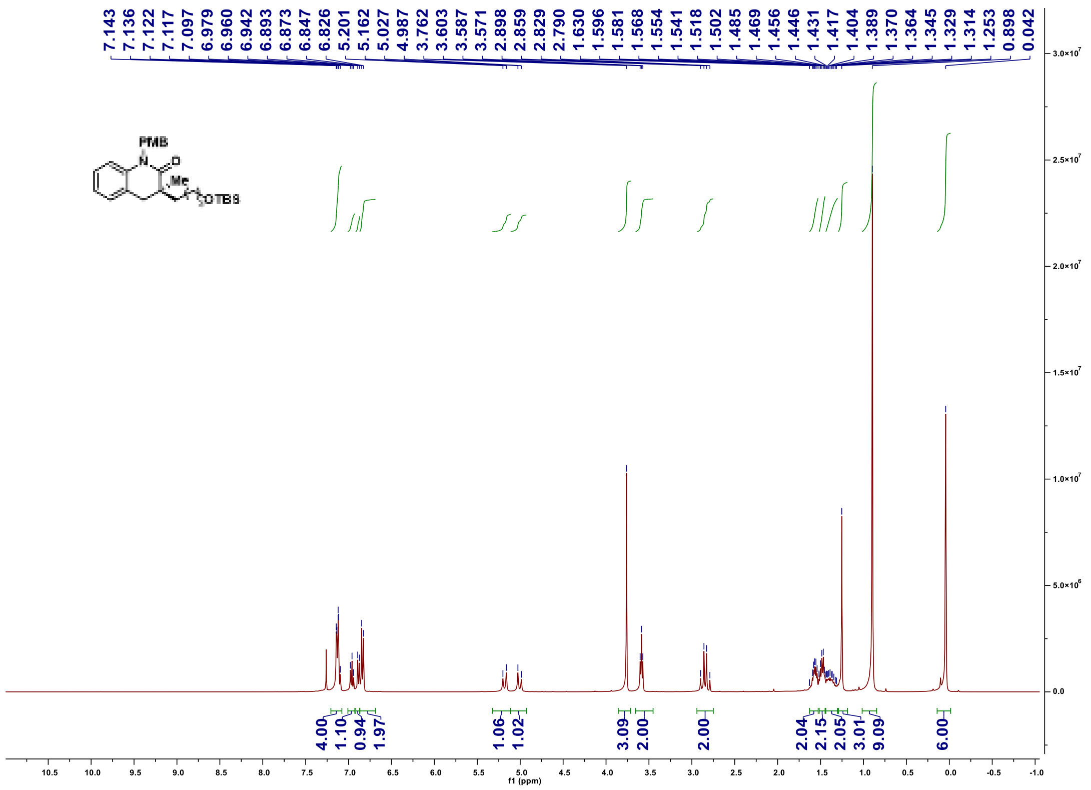

Supplementary Figure 123. ^{1}H NMR-spectrum (400 MHz, $CDCl_3$) of **3u**

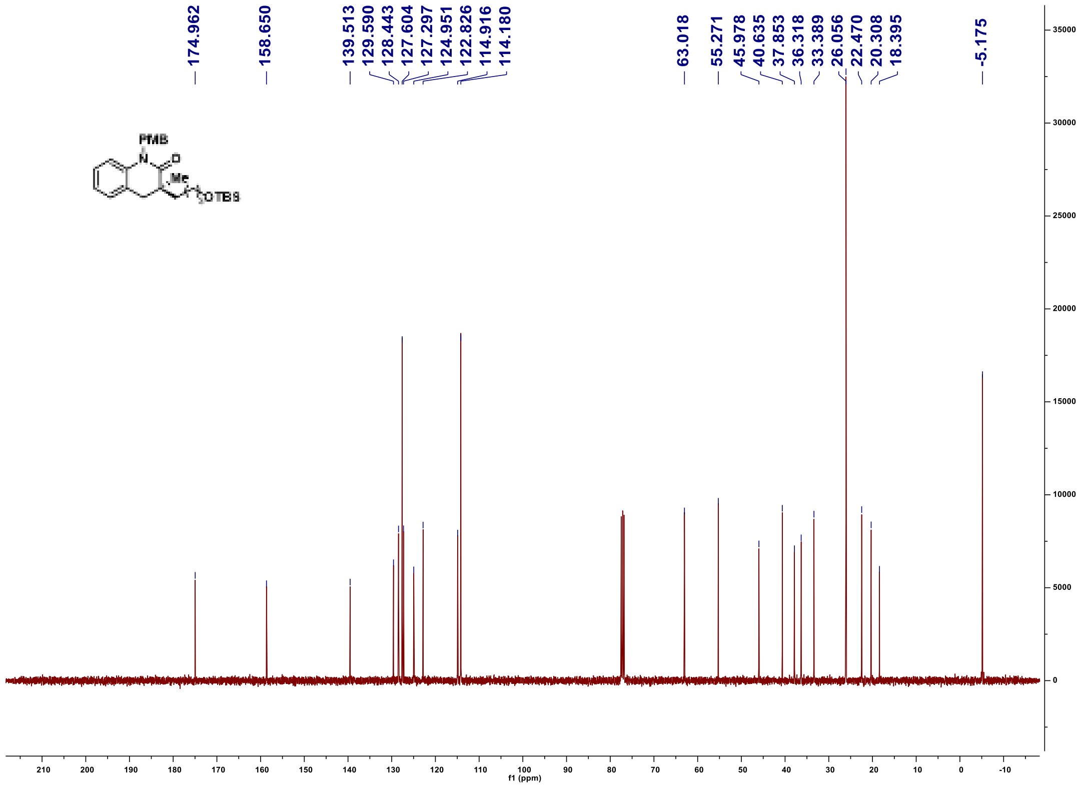

Supplementary Figure 124. ^{13}C NMR-spectrum (100 MHz, $CDCl_3$) of **3u**

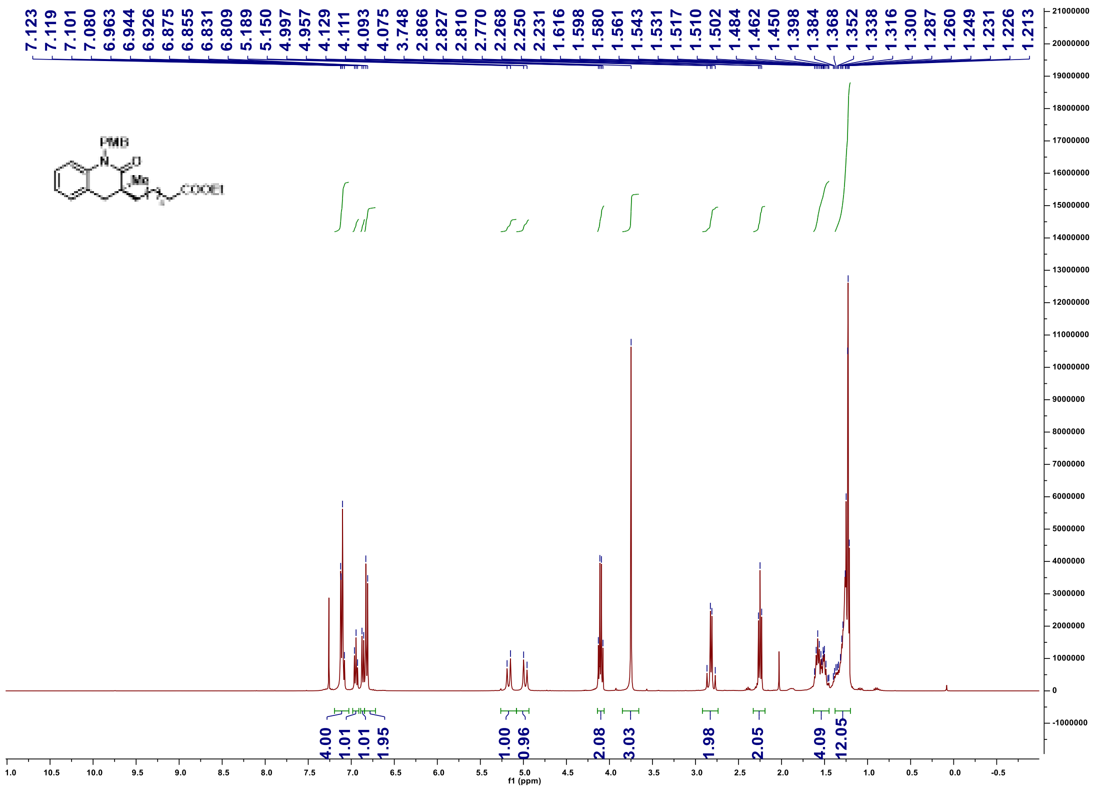

Supplementary Figure 125. ^{1}H NMR-spectrum (400 MHz, $CDCl_3$) of **3v**

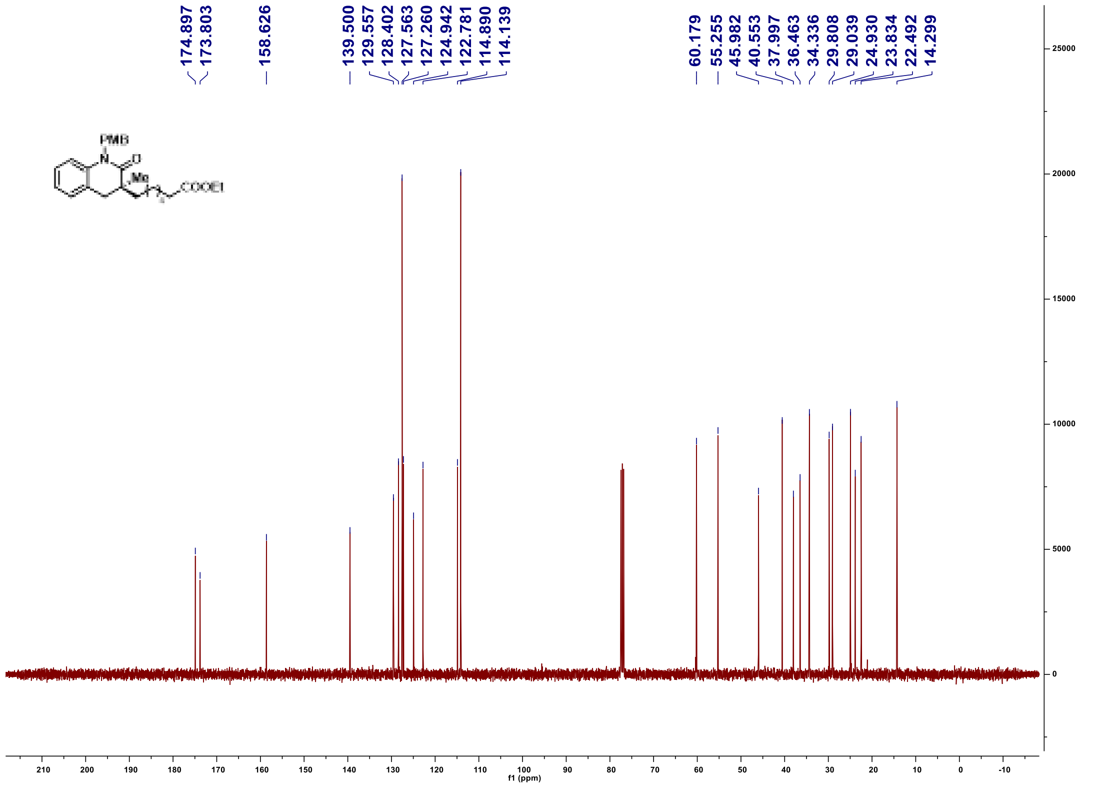

Supplementary Figure 126. ^{13}C NMR-spectrum (100 MHz, $CDCl_3$) of **3v**

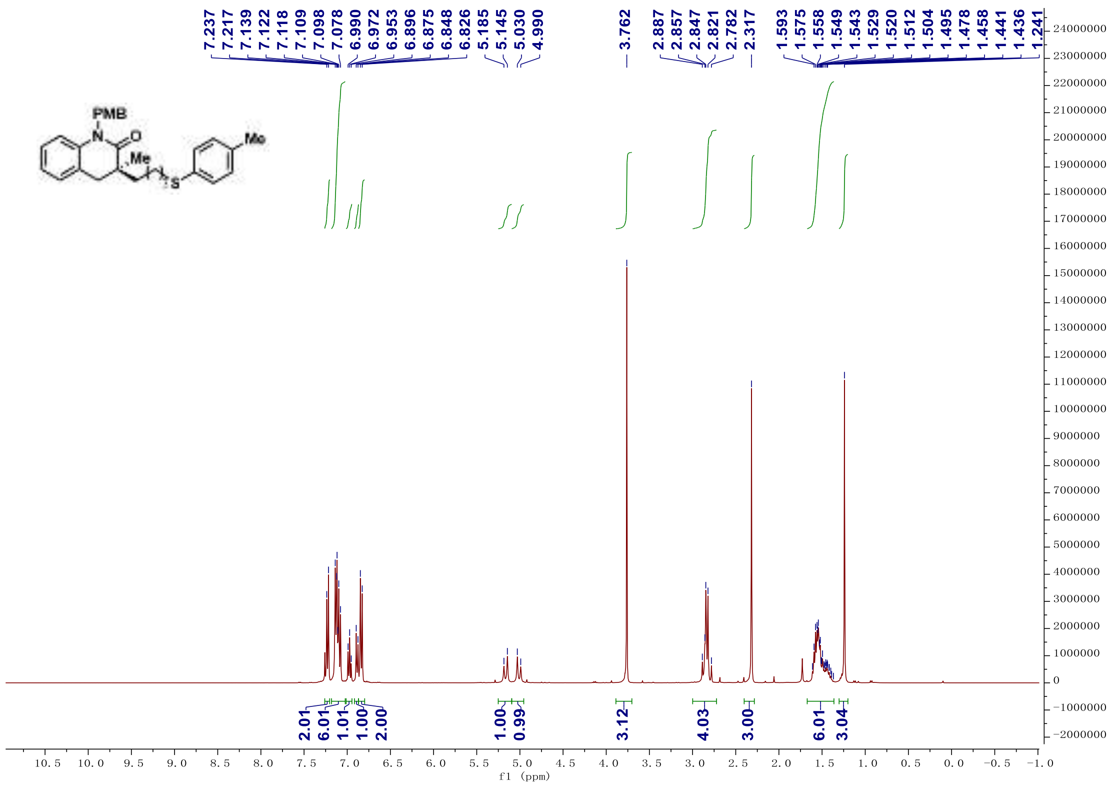

Supplementary Figure 127. ^{1}H NMR-spectrum (400 MHz, $CDCl_3$) of **3w**

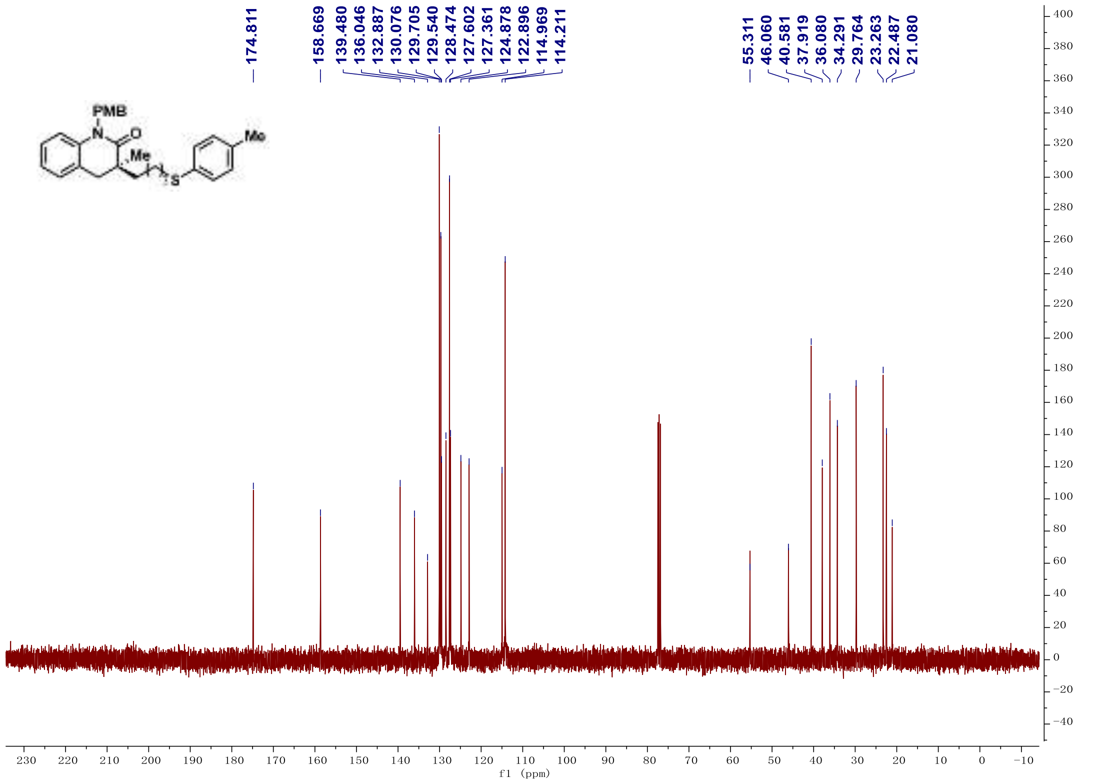

Supplementary Figure 128. ^{13}C NMR-spectrum (100 MHz, $CDCl_3$) of **3w**

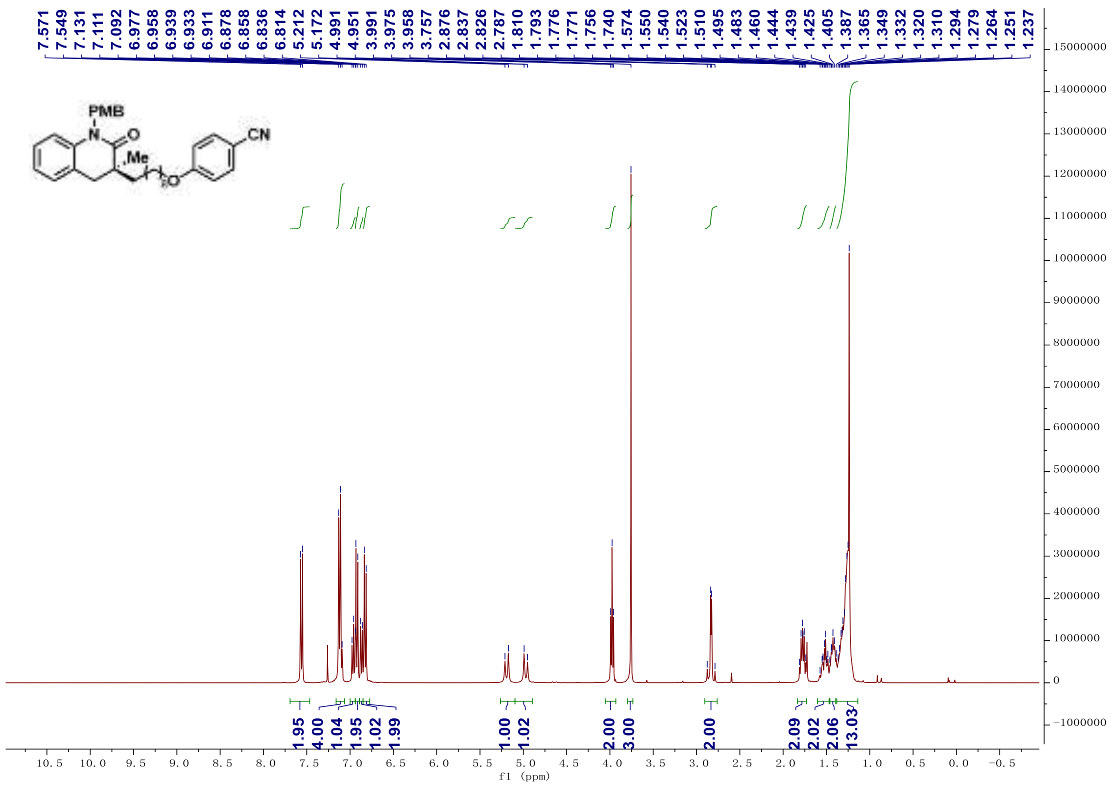

Supplementary Figure 129. ^{1}H NMR-spectrum (400 MHz, $CDCl_3$) of **3x**

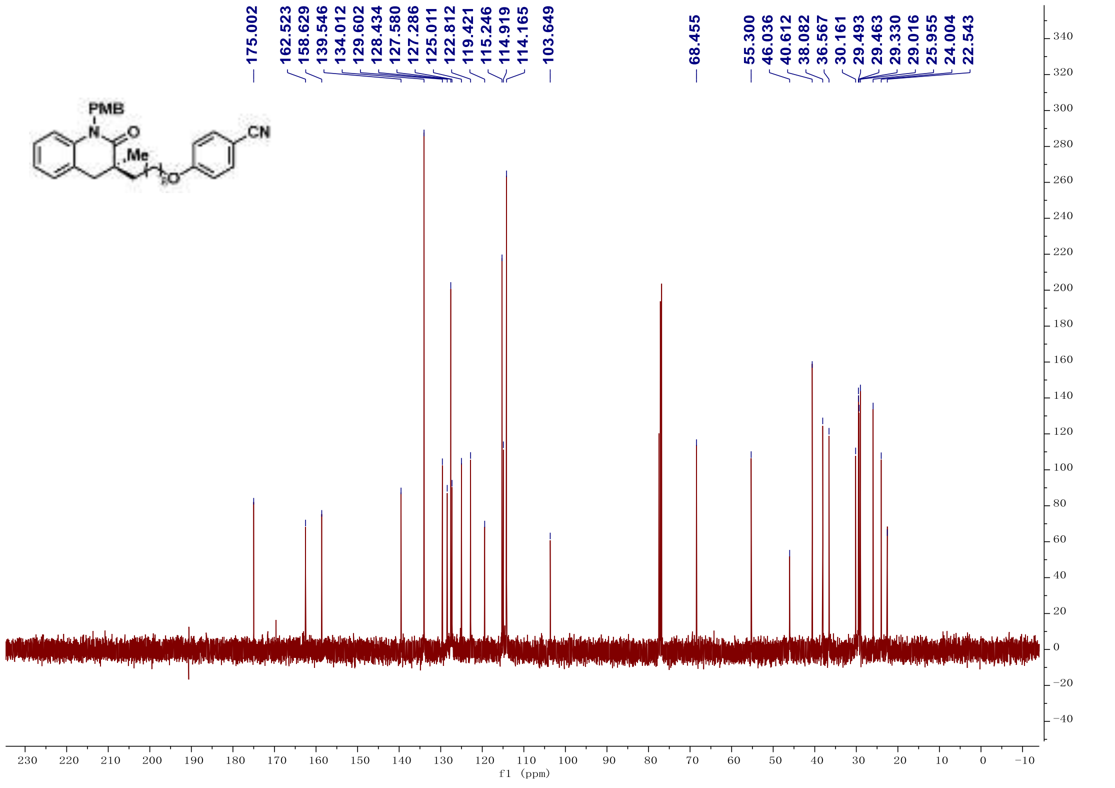

Supplementary Figure 130. ^{13}C NMR-spectrum (100 MHz, $CDCl_3$) of **3x**

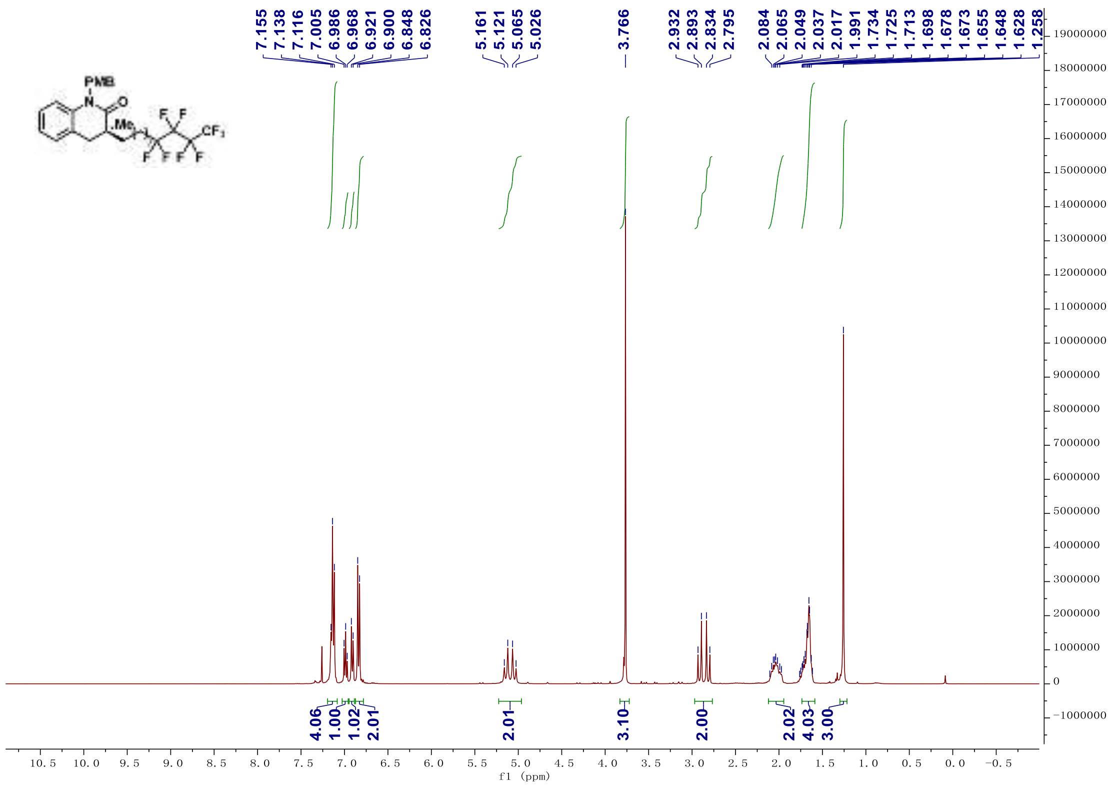

Supplementary Figure 131. ^{1}H NMR-spectrum (400 MHz, $CDCl_3$) of **3y**

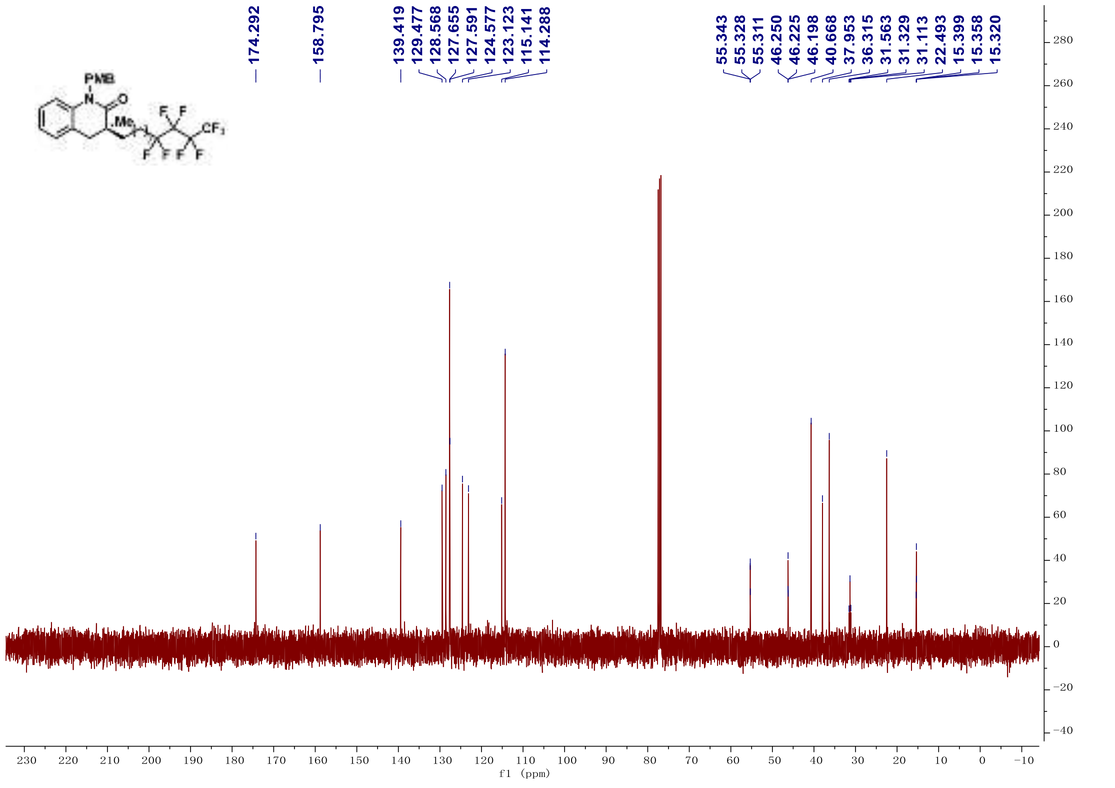

Supplementary Figure 132. ^{13}C NMR-spectrum (100 MHz, $CDCl_3$) of **3y**

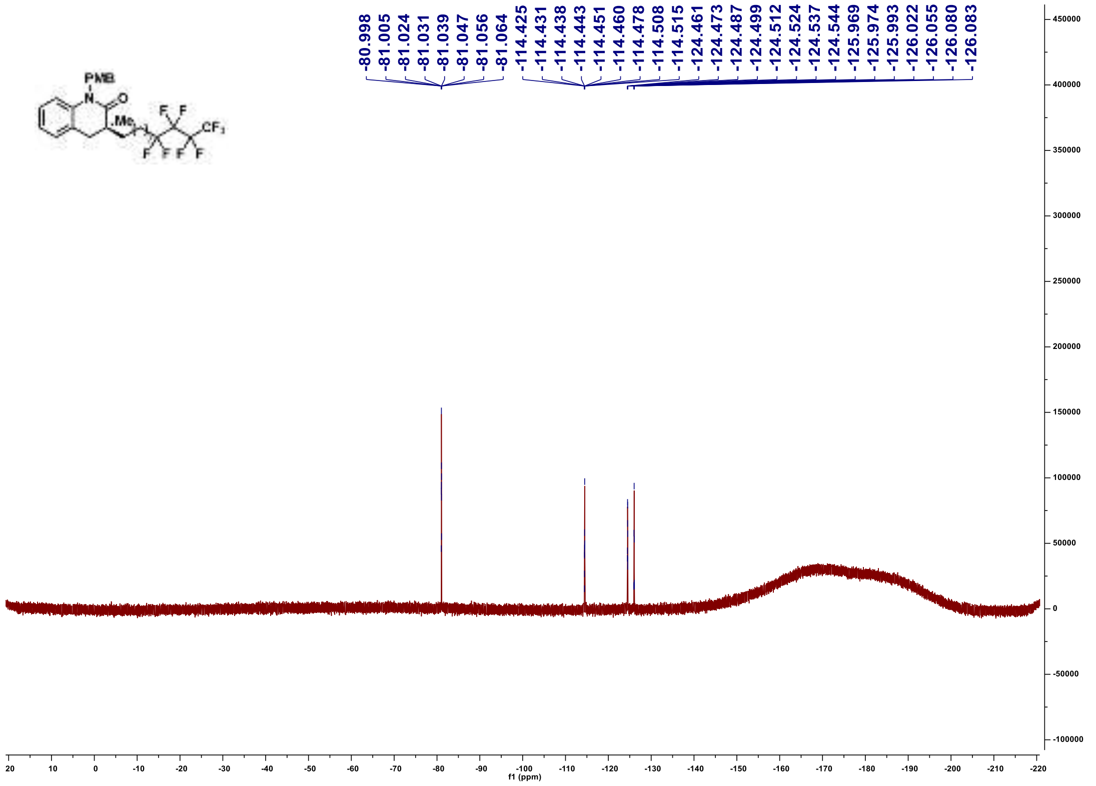

Supplementary Figure 133. ^{19}F NMR-spectrum (376 MHz, $CDCl_3$) of **3y**

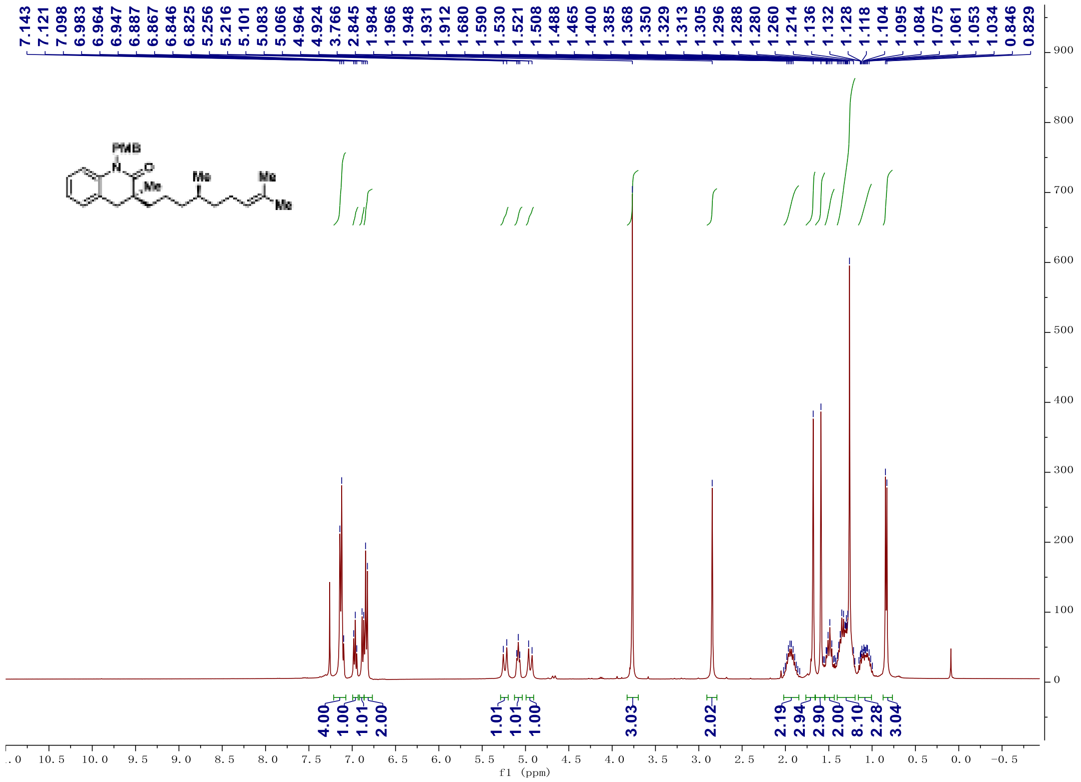

Supplementary Figure 134. ^{1}H NMR-spectrum (400 MHz, $CDCl_3$) of **3z**

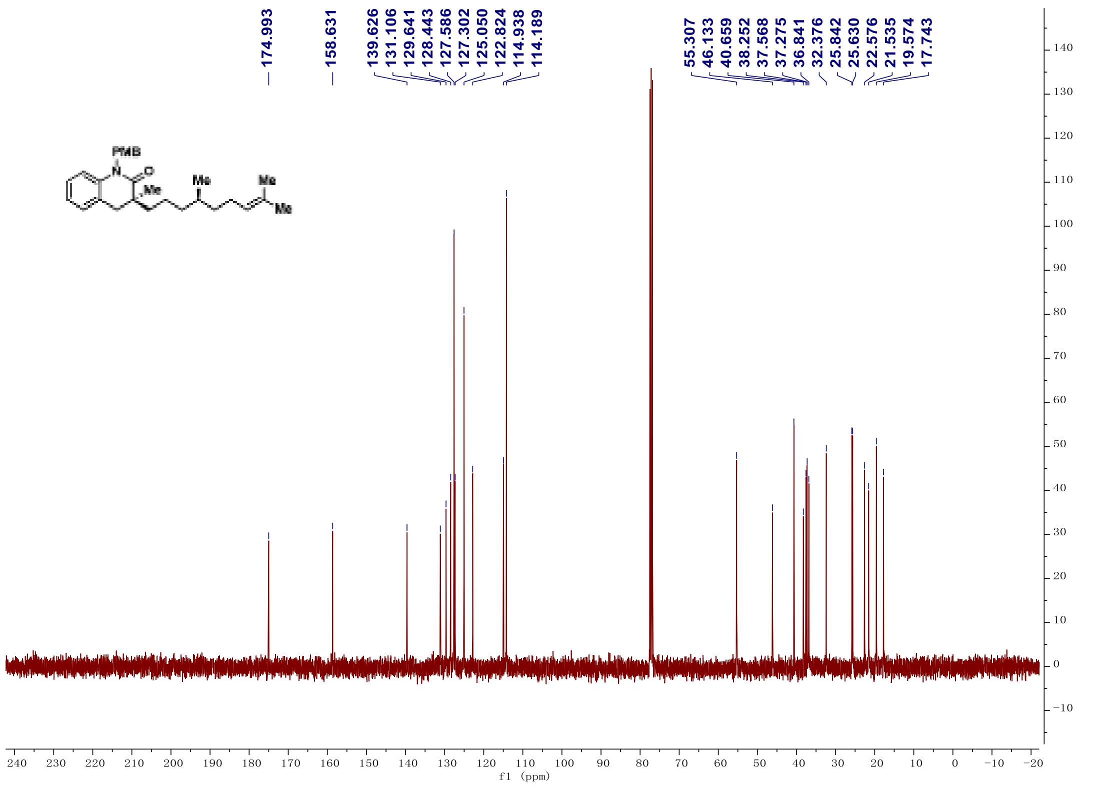

Supplementary Figure 135. ^{13}C NMR-spectrum (100 MHz, $CDCl_3$) of **3z**

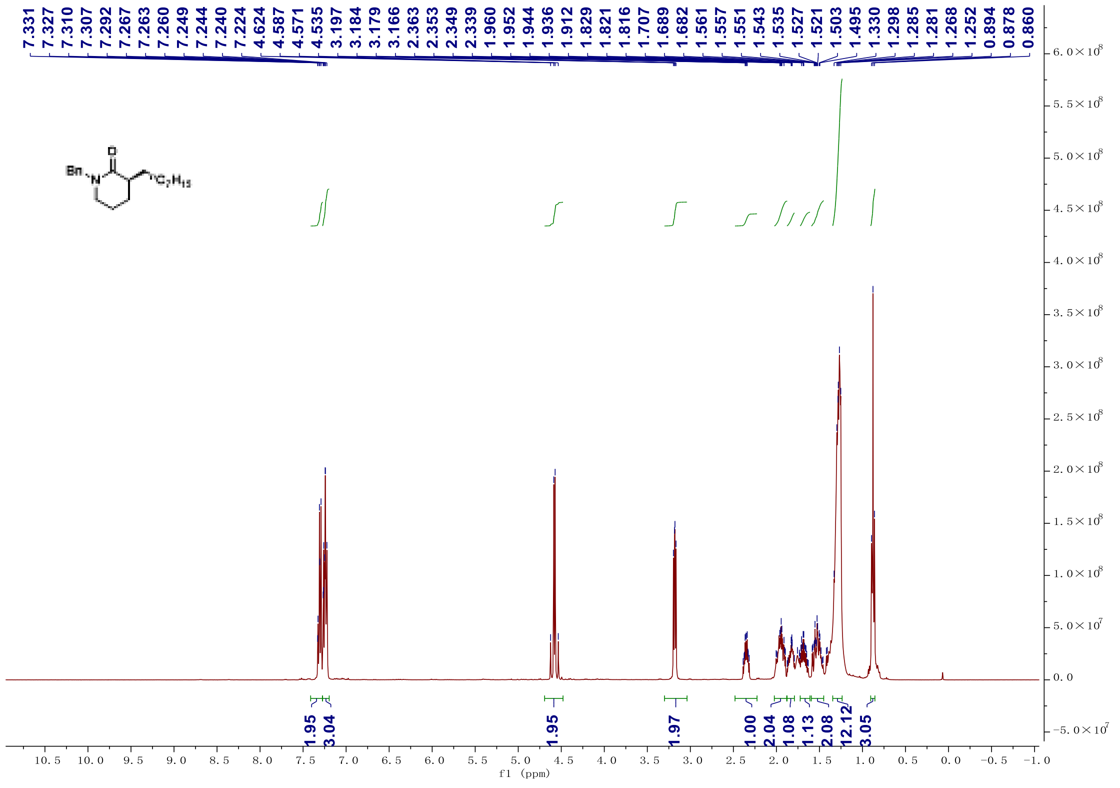

Supplementary Figure 136. ^{1}H NMR-spectrum (400 MHz, $CDCl_3$) of **5a**

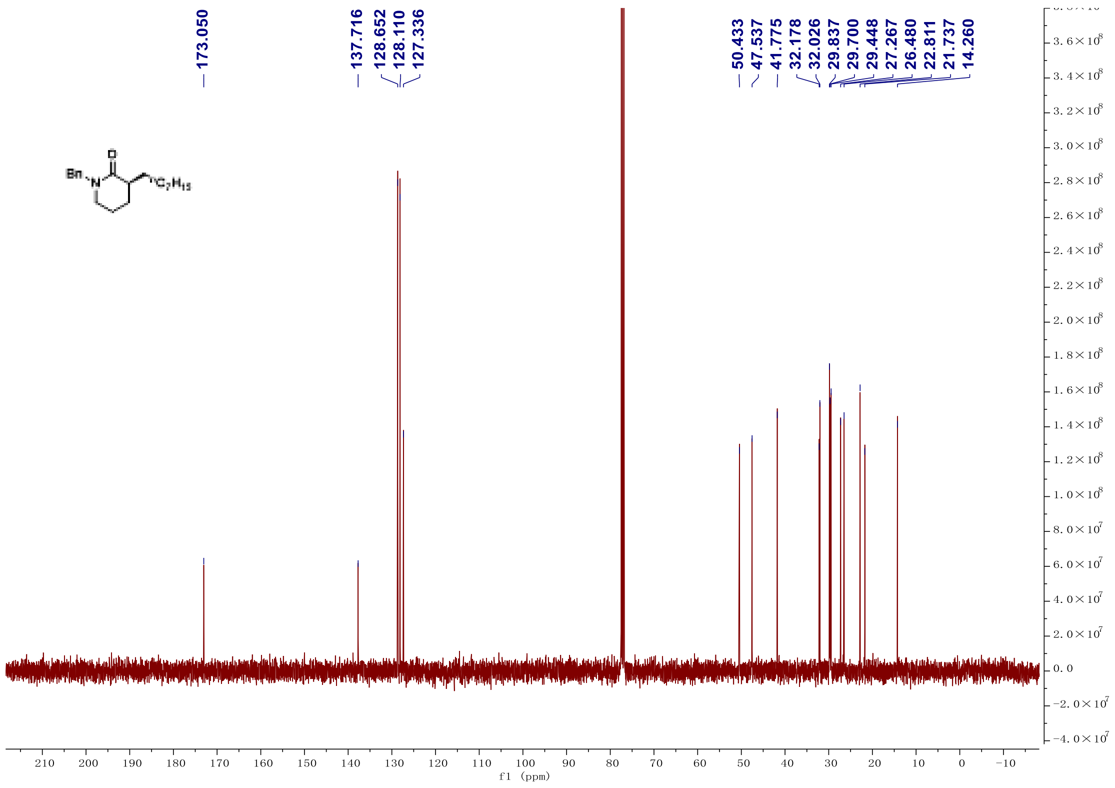

Supplementary Figure 137. ^{13}C NMR-spectrum (100 MHz, $CDCl_3$) of **5a**

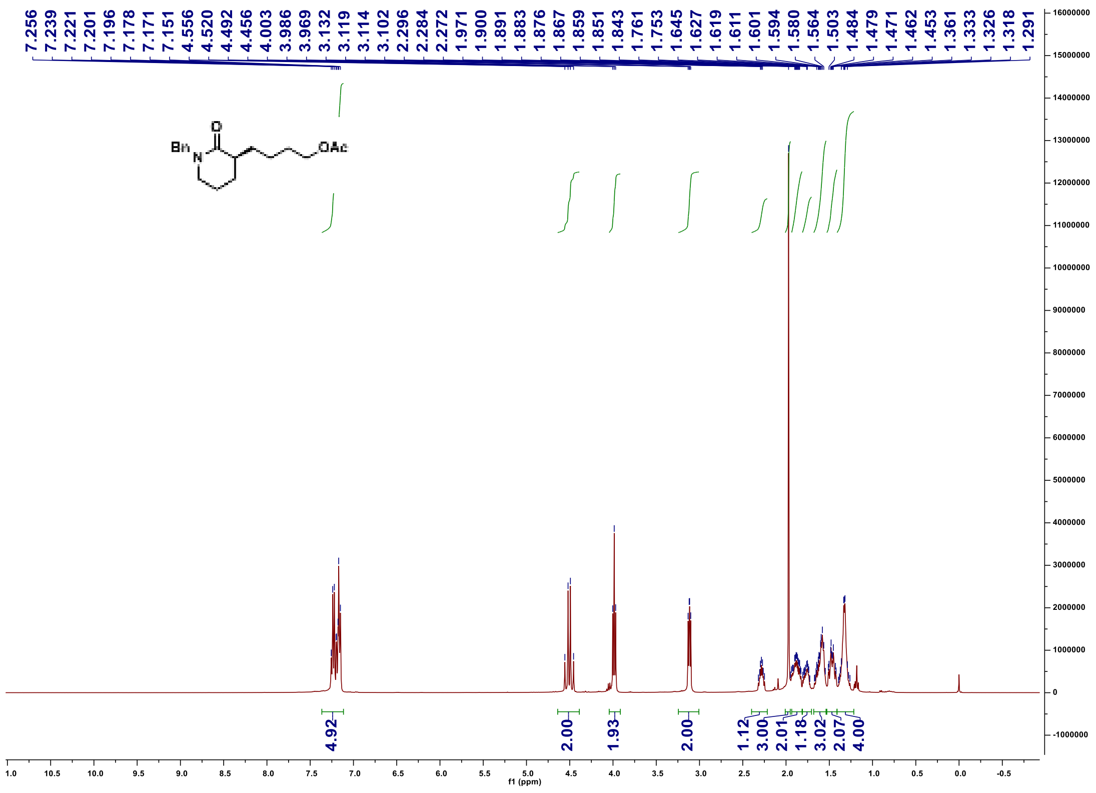

Supplementary Figure 138. ^{1}H NMR-spectrum (400 MHz, $CDCl_3$) of **5b**

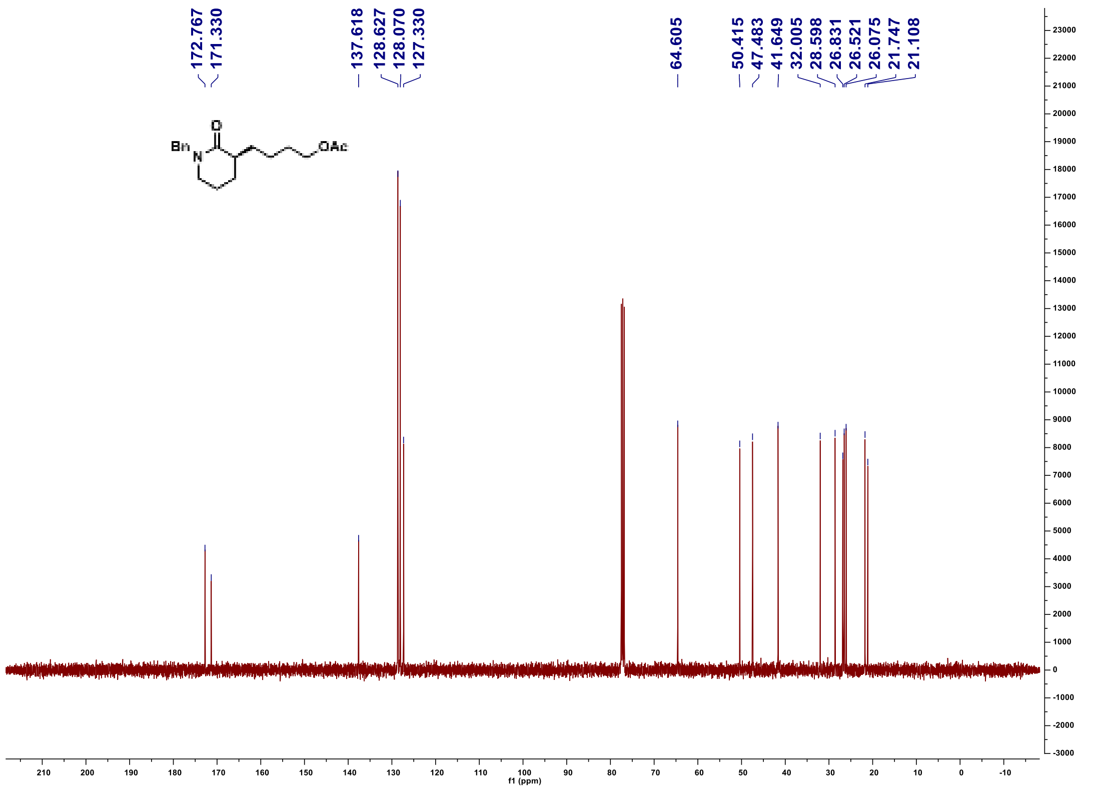

Supplementary Figure 139. ^{13}C NMR-spectrum (100 MHz, $CDCl_3$) of **5b**

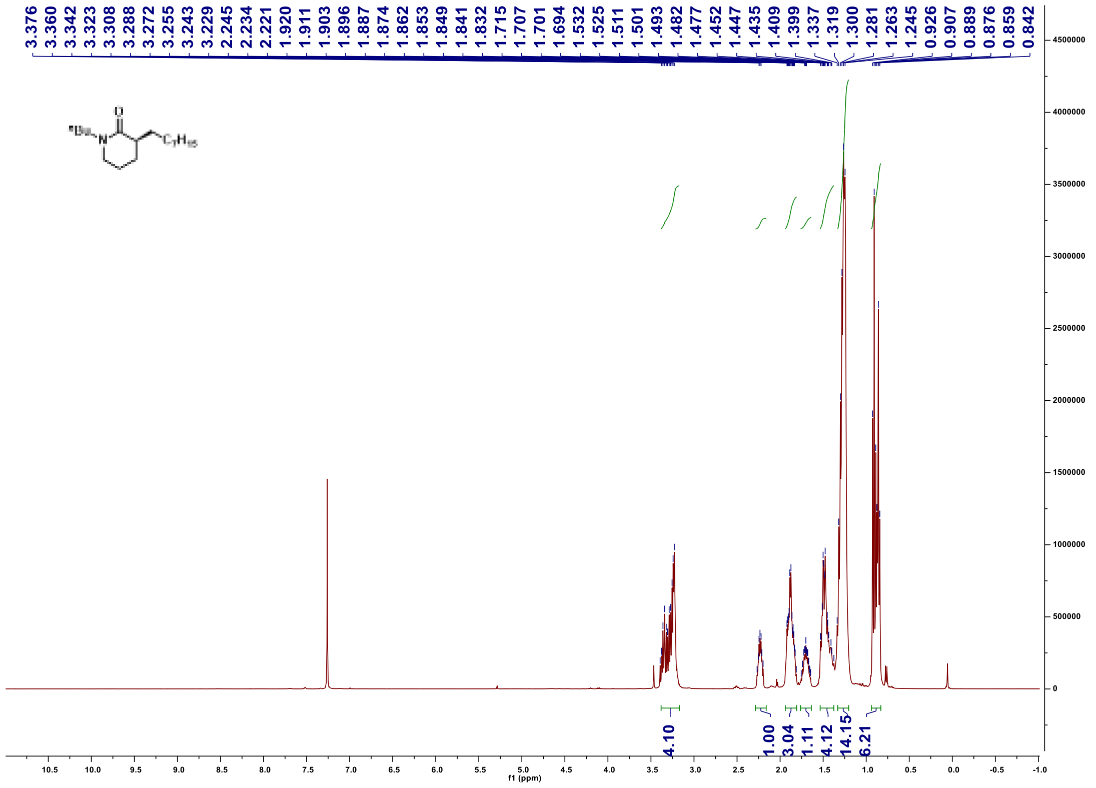

Supplementary Figure 140. ^{1}H NMR-spectrum (400 MHz, $CDCl_3$) of **5c**

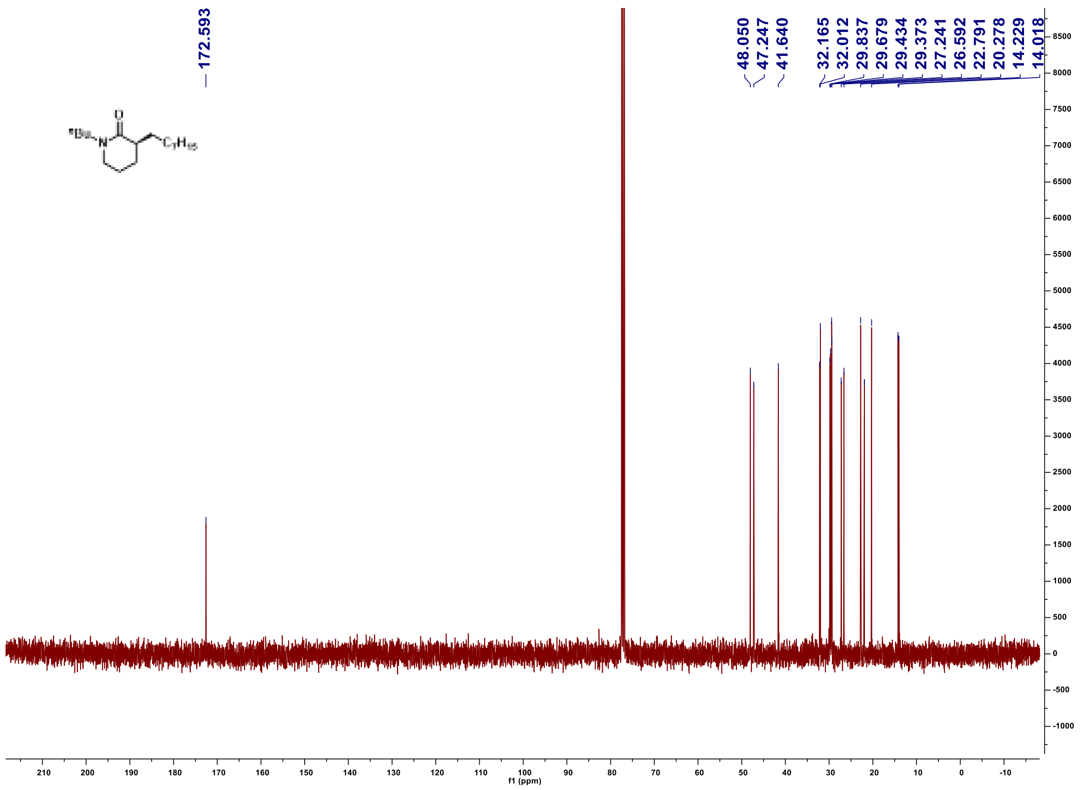

Supplementary Figure 141. ^{13}C NMR-spectrum (100 MHz, $CDCl_3$) of **5c**

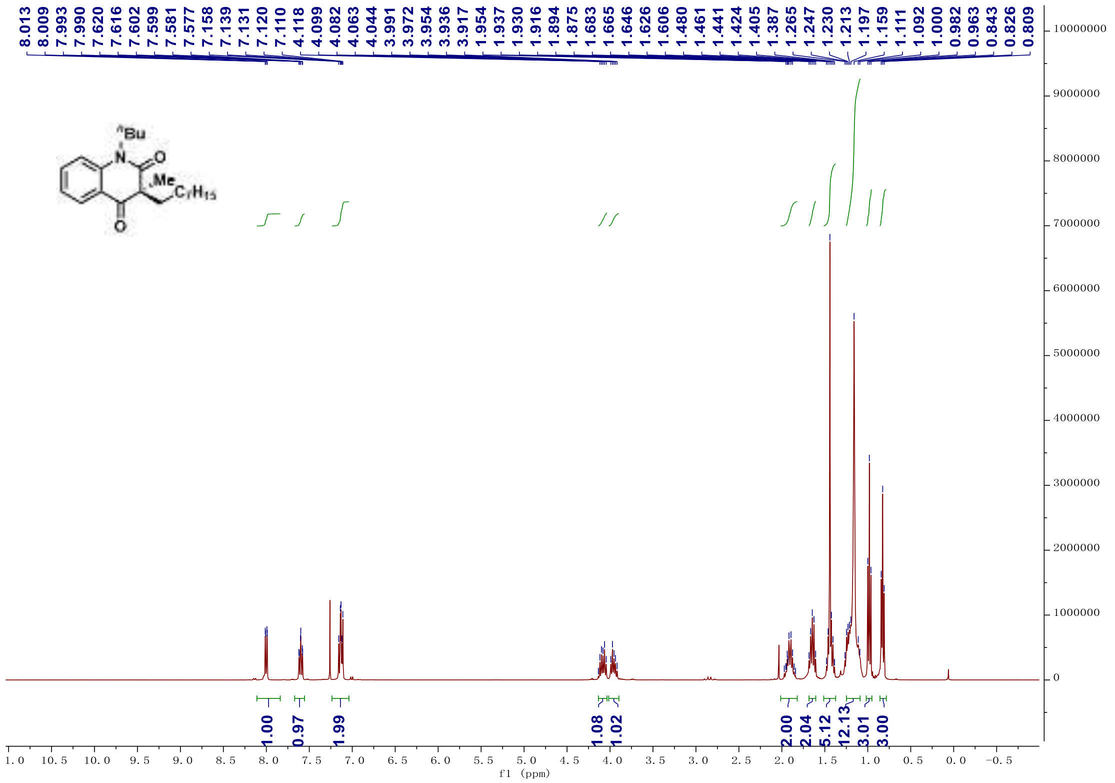

Supplementary Figure 142. ^{1}H NMR-spectrum (400 MHz, $CDCl_3$) of **6**

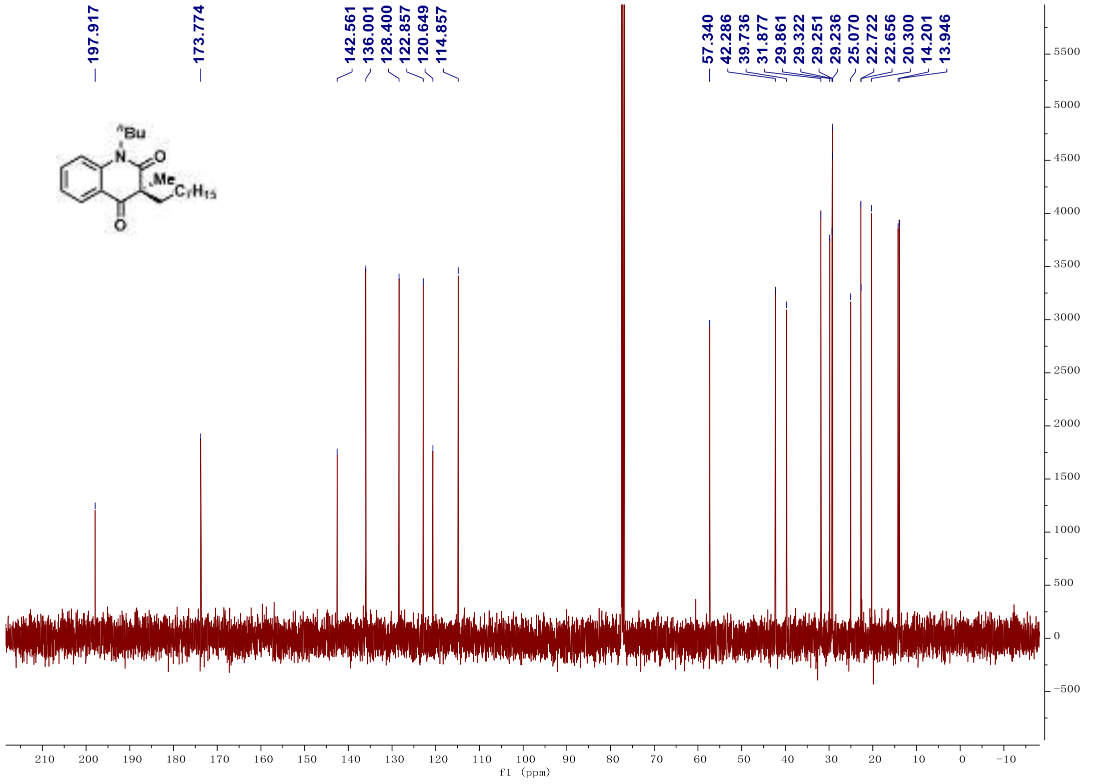

Supplementary Figure 143. ^{13}C NMR-spectrum (100 MHz, $CDCl_3$) of **6**

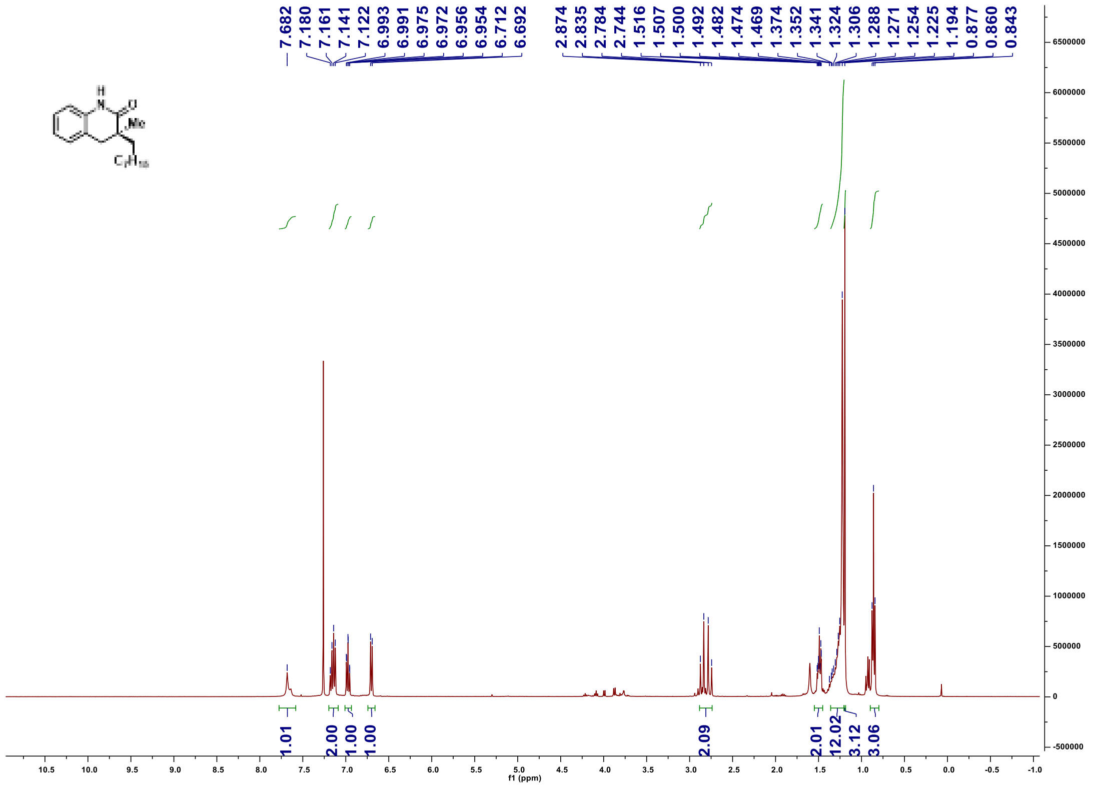

Supplementary Figure 144. ^{1}H NMR-spectrum (400 MHz, $CDCl_3$) of **7**

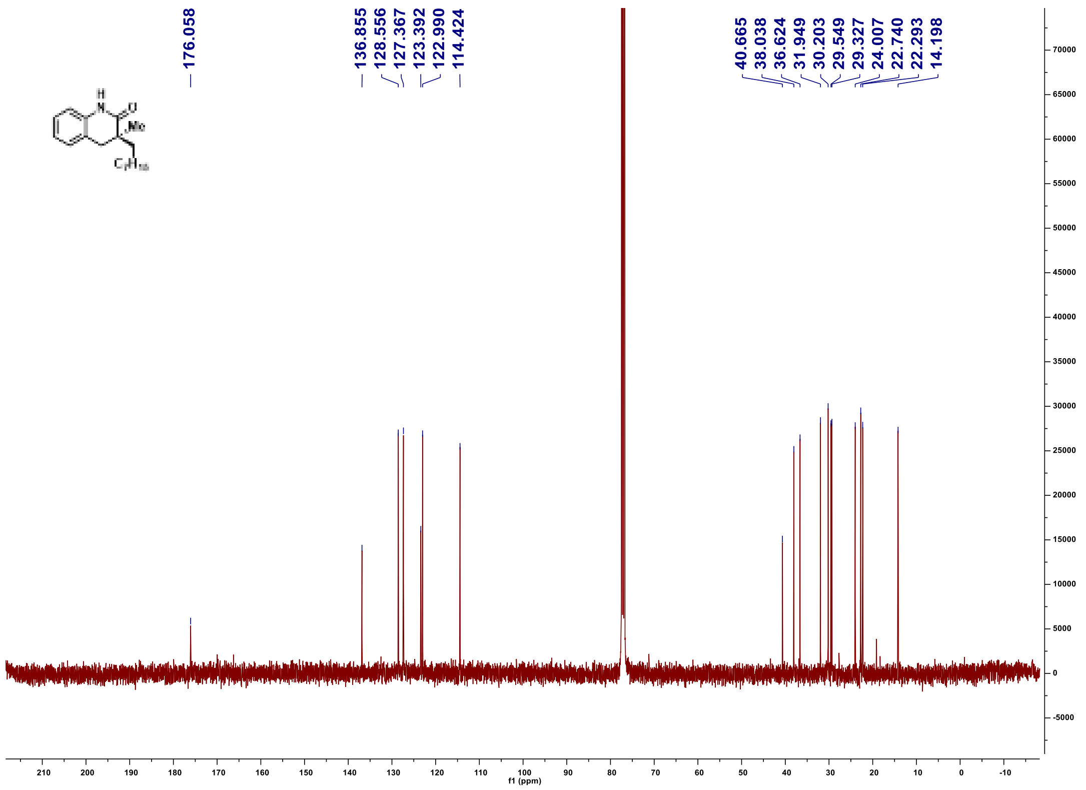

Supplementary Figure 145. ^{13}C NMR-spectrum (100 MHz, $CDCl_3$) of **7**

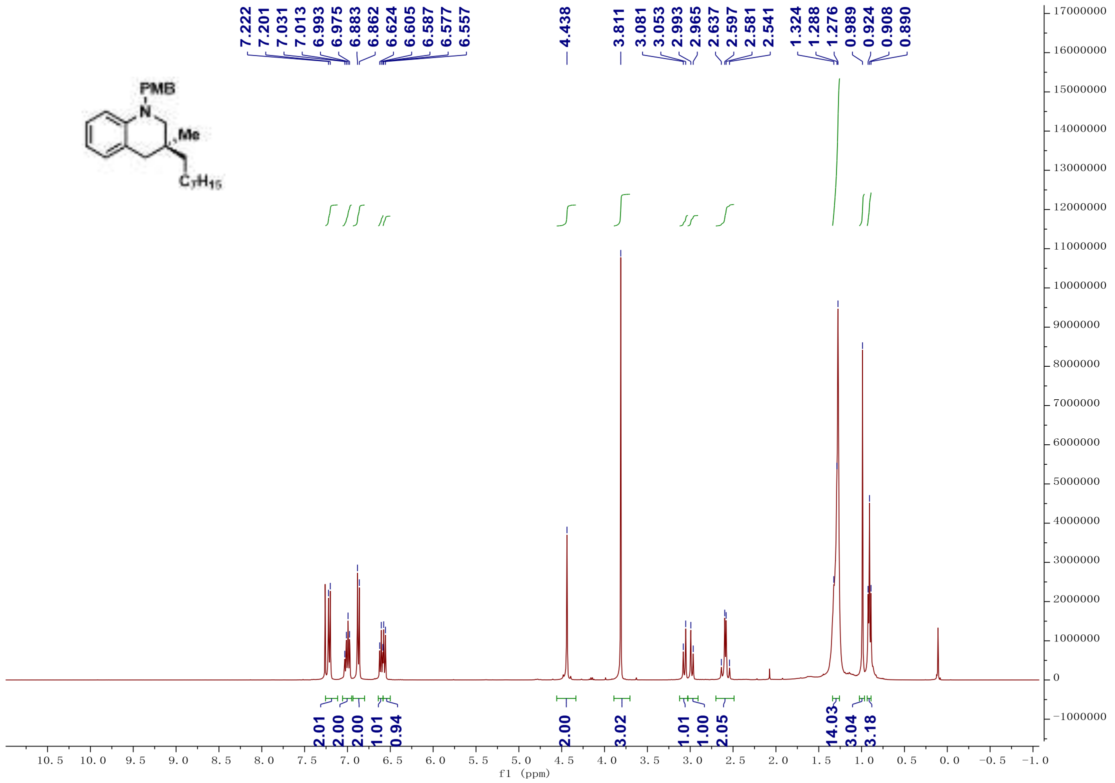

Supplementary Figure 146. ^{1}H NMR-spectrum (400 MHz, $CDCl_3$) of **8**

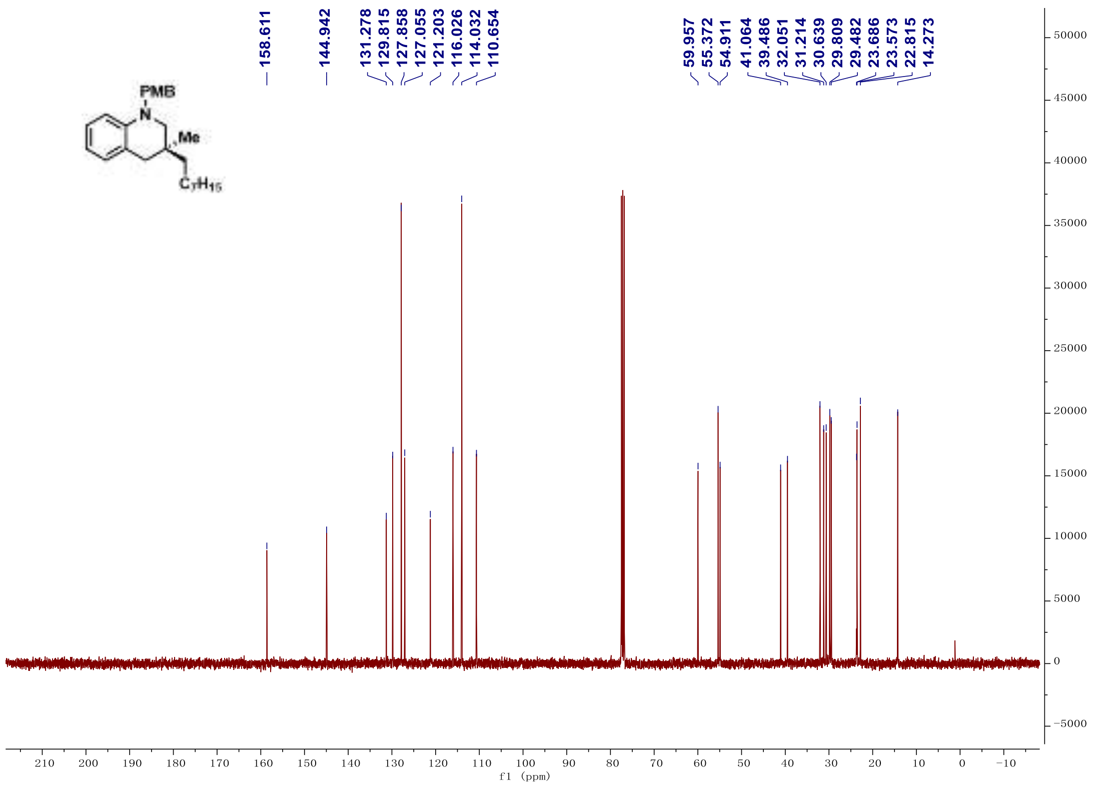

Supplementary Figure 147. ^{13}C NMR-spectrum (100 MHz, $CDCl_3$) of **8**

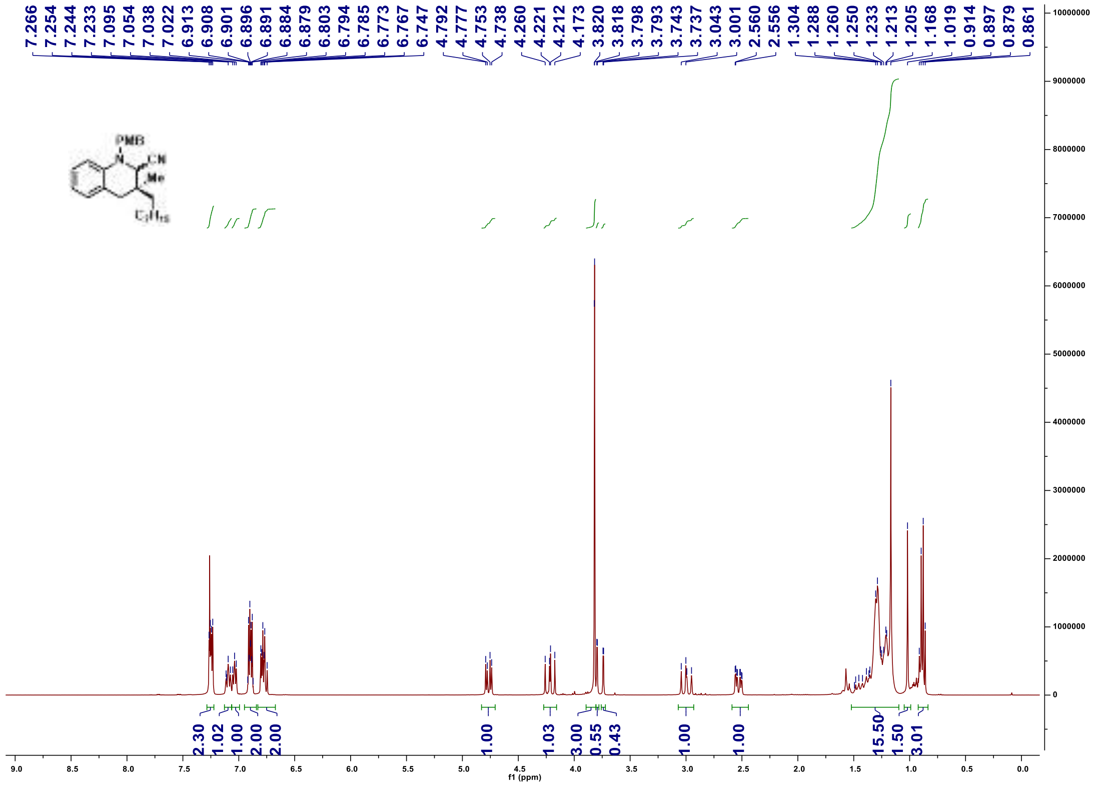

Supplementary Figure 148. ^{1}H NMR-spectrum (400 MHz, $CDCl_3$) of **9**

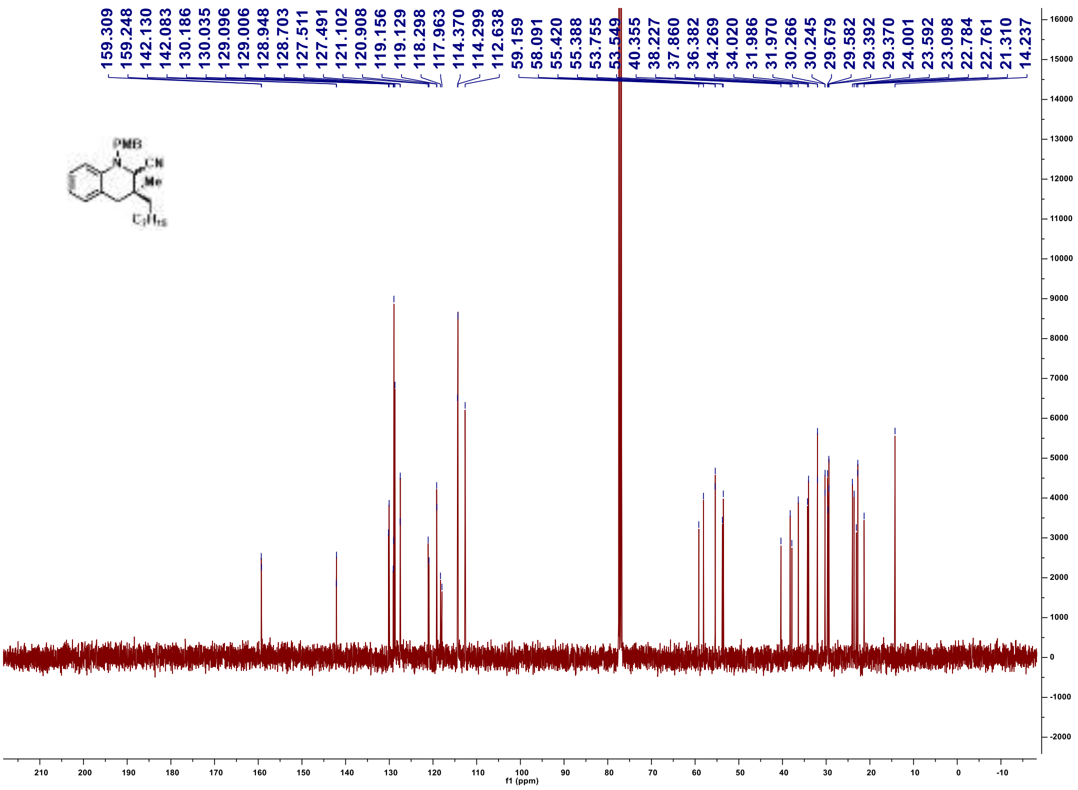

Supplementary Figure 149. ^{13}C NMR-spectrum (100 MHz, $CDCl_3$) of **9**

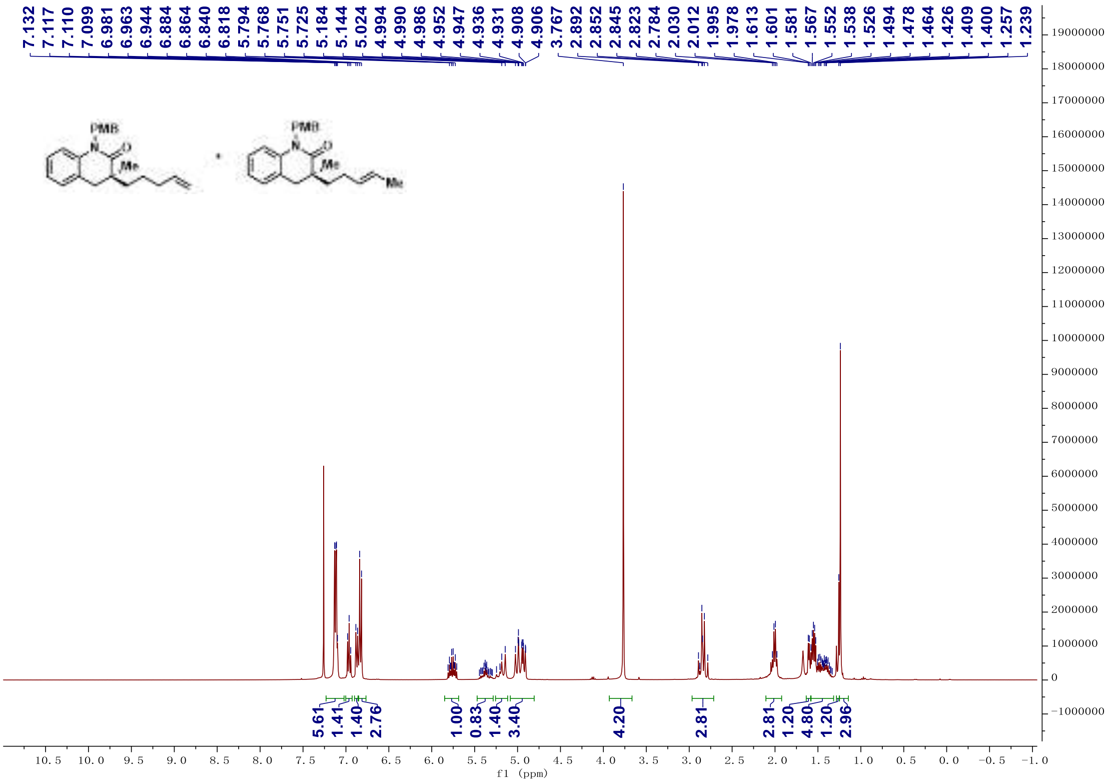

Supplementary Figure 150. ^{1}H NMR-spectrum (400 MHz, $CDCl_3$) of **10** and **11**

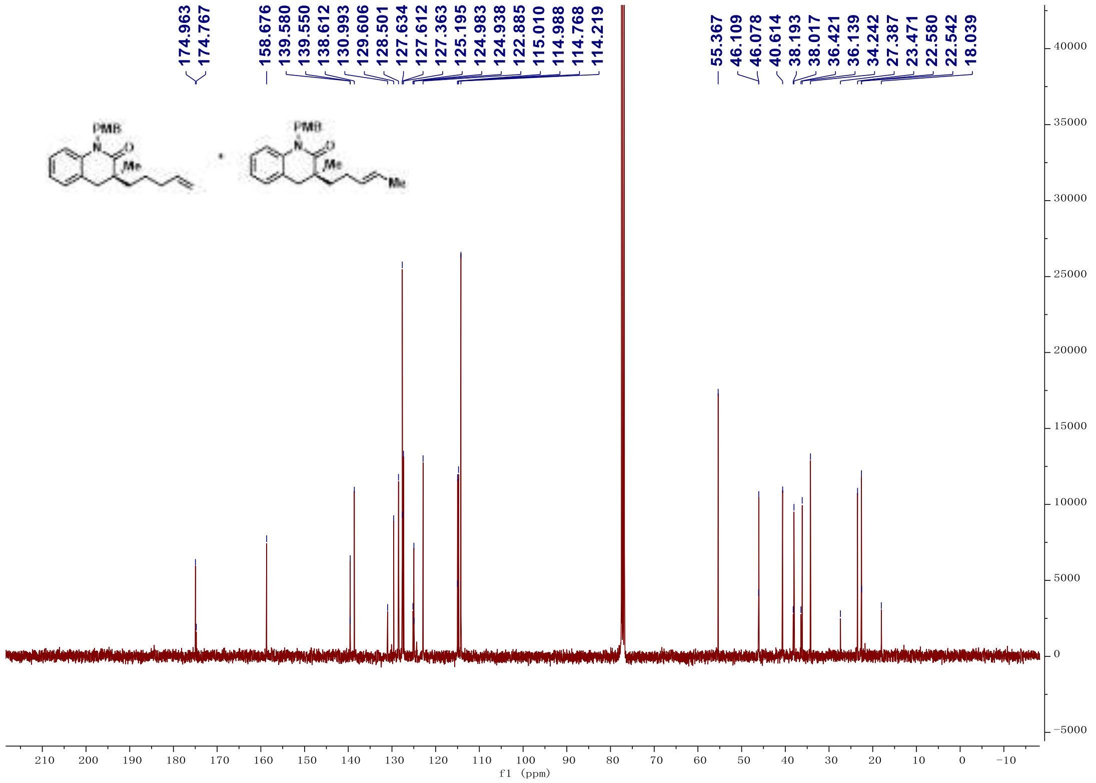

Supplementary Figure 151. ^{13}C NMR-spectrum (100 MHz, $CDCl_3$) of **10** and **11**

2 Supplementary References

1. M. Rauser, R. Eckert, M. Gerbershagen, M. Niggemann, *Angew. Chem. Int. Ed*. **2019**, *58*, 6713; *Angew. Chem*. **2019**, *131*, 6785.
2. S. Nagasawa, Y. Sasano, Y. Iwabuchi, *Angew. Chem. Int. Ed*. **2016**, *55*, 13189; *Angew. Chem*. **2016**, *128*, 13383.
3. X. Wu, J. Qu, Y. Chen, *J. Am. Chem. Soc*. **2020**, *142*, 15654.
4. N. Basar, K. Damodaran, H. Liu, G. A. Morris, H. M. Sirat, E. J.; Thomas, D. P. Curran, *J. Org. Chem*. **2014**, *79*, 7477.
5. E. de Pedro Beato, D. Mazzarella, M. Balletti, P. Melchiorre, *Chem. Sci*. **2020**, *11*, 6312.
6. M. Newcomb, K. A. Weber, *J. Org. Chem.* **1991**, *56*, 1309.
7. I. D. G. Watson, S. Ritter, F. D. Toste, *J. Am. Chem. Soc*. **2009**, *131*, 2056.
8. J. Xiao, Y.-W. Wang, Y. Peng, *Chem. Commun*. **2018**, *54*, 2040.
